# Decarbonylation Enables Chemodivergent Access to Guaiazulene-3-Oxalic and ‑Carboxylic Acid Derivatives

**DOI:** 10.1021/acs.joc.6c00848

**Published:** 2026-06-22

**Authors:** Emma Audi, Carly M. Zack, Esther M. Ntankwa, William Blake Heston, Annika S. Waples, Manana Tsiskarishvili, C. Rose Kennedy, Shuming Chen, J. Patrick Lutz

**Affiliations:** † Department of Chemistry, St. Lawrence University, Canton, New York 13617, United States; ‡ Department of Chemistry and Biochemistry, 6167 Oberlin College, Oberlin, Ohio 44074, United States; § Department of Chemistry, 6927 University of Rochester, Rochester, New York 14627, United States

## Abstract

Guaiazulene derivatives are of interest due to the low cost of guaiazulene compared to azulene while still retaining many of the same unique electronic properties. Syntheses of guaiazulene-3-carboxylic acid and azulene-1-carboxylic acid derivatives have been described, but they typically rely on uncommon or toxic reagents such as oxalyl bromide or phosgene. We describe a new route to these compounds by functionalization with oxalyl chloride, followed by thermal or bromide-induced decarbonylation and quenching with a nucleophile. Combined experimental and computational studies suggest a concerted 1,2-halide shift mechanism for the decarbonylation.

## Graphical abstract

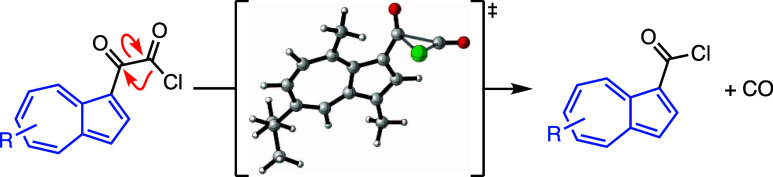

## Introduction

Azulene (**1**), a 10-π-electron hydrocarbon, is an isomer of naphthalene with distinctly different properties that have long intrigued chemists (Figure 1).^1^ Azulene was first synthesized in 1936,^2^ but new insights into its electronic structure continue to the present day.^3^ While naphthalene is a nonpolar, alternant hydrocarbon, azulene is non-alternant due to its fused odd-membered rings. Azulene’s π-system is polarized such that the five-membered ring has the character of a cyclopentadienyl anion and the seven-membered ring has the character of a tropylium cation. Notable properties of azulene include a deep blue color^4^ and a significant dipole moment of 1.08 D.^5^ The electronically polarized ring system facilitates oxidations and reductions of azulene derivatives at low potentials,^6^ which has resulted in much interest toward using azulene-based compounds in materials applications,^7–9^ including organic field-effect transistors,^10^ solar cells,^11^ photoswitches,^12^ chemosensors,^13–16^ and chemodosimeters.^17^ Azulene compounds have also seen some applications in a medicinal chemistry context.^18–20^

**Figure 1:**
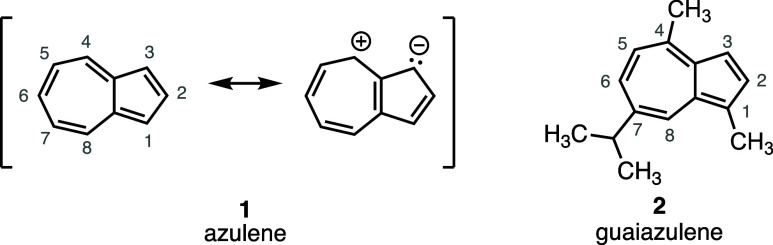
Structures of azulene (**1**) and guaiazulene (**2**).

Despite its utility, azulene remains expensive to purchase (>$130/g), as it can only be prepared via multistep syntheses.^1^ In contrast, the azulene derivative guaiazulene (**2**) can be isolated from renewable sources such as chamomile oil^21^ and is much more affordable (∼$2/g, Figure 1). Because guaiazulene retains comparable optical and electronic properties to azulene, it is possible that guaiazulene derivatives could have similar utility to azulene derivatives at a significantly reduced cost. However, the applications of guaiazulene-based materials are underexplored,^22–24^ perhaps due in part to the lack of synthetic methods described for functionalizing guaiazulene compared to azulene.^25–28^

Many approaches to functionalizing azulene and its derivatives rely on the electronic polarization of the 5,7-ring system. The five-membered ring is partially anionic in nature and tends to react as a nucleophile at positions 1 and 3, whereas the cationic seven-membered ring is more electrophilic.^29,30^ We aimed to develop a synthesis of guaiazulene-3-carboxylic acid derivatives via the reaction of guaiazulene with an electrophilic carbonyl synthon. We ultimately found that oxalyl chloride is an effective reagent for this process (Scheme 1). Depending on the reaction conditions, the functionalization can occur with or without decarbonylation, leading to carboxylic acid or oxalic acid derivatives, respectively. Mechanistic investigations support a concerted 1,2-halide shift pathway for the decarbonylation.

**Scheme 1:**
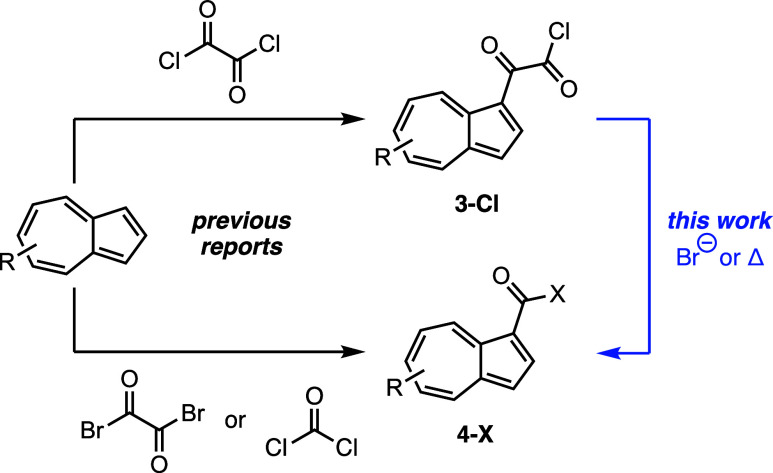
Reactivity of Azulene and Derivatives with Oxalyl Chloride/Bromide and Phosgene

## Results and Discussion

### Method Development

Our initial investigations focused on a two-step sequence wherein electrophilic aromatic substitution of guaiazulene with trichloroacetyl chloride would form the guaiazulene-3-trichloromethyl ketone (**5**), followed by basic hydrolysis to give the corresponding 3-carboxylic acid (**6**, Scheme 2). The first step proceeded smoothly, and thin-layer chromatography indicated consumption of **5** in the basic hydrolysis step to form a highly polar purple compound believed to be the guaiazulene-3-carboxylate ion. However, after acidifying the reaction in an effort to form the neutral carboxylic acid **6**, we instead reisolated guaiazulene **2** in 85% yield. This observation suggested that the guaiazulene-3-carboxylic acid is inherently unstable to decarboxylation and hence would not be a viable precursor to other carboxylic acid derivatives. Interestingly, a previous report described syntheses of the C1, C2, and C4 carboxylic acids of guaiazulene, but not the C3 derivative, possibly due to this instability.^26^ Azulene-1-carboxylic acid is a known compound; presumably the higher basicity of the guaiazulene ring system compared to azulene accelerates a protonation-mediated decarboxylation of **6** (p*K*_a_ = −1.76 and +1.42 for the conjugate acids of azulene and guaiazulene, respectively).^31^

**Scheme 2:**
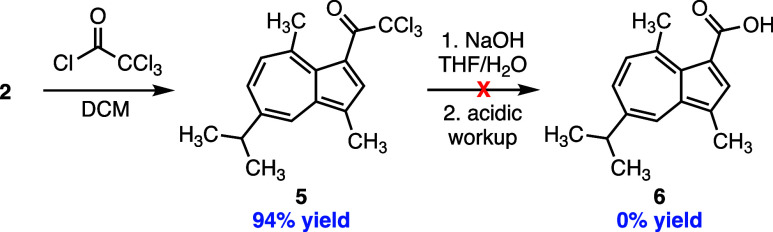
Attempted Synthesis of Guaiazulene-3-Carboxylic Acid

Our next strategy was to employ **5** directly in reactions with alcohols/amines to generate esters/amides via acyl substitution. This approach was successful for 1°, unhindered nucleophiles as previously described.^32^ However, on further testing we found that more sterically demanding reagents led to little or no conversion of **5**, likely due to the trichloromethyl ketone being quite hindered itself. To address this deficiency, we turned our attention toward methods to access the guaiazulene-3-acid chloride instead. Azulene and derivatives thereof are known to react with phosgene to yield acid chloride products^18,19,33–36^ but we sought to avoid such a hazardous reagent. Instead, we looked to examples of azulenes reacting with oxalyl chloride and bromide. Use of oxalyl chloride is reported to afford dicarbonyl chlorides (**3-Cl**),^19,37–40^ while use of oxalyl bromide leads to formation of monocarbonyl bromides (**4-Br**) via a decarbonylation process that has not been investigated in detail (Scheme 1).^26,41–44^ We questioned whether we could identify conditions to access these two classes of product using the more commonly available oxalyl chloride in both cases.

We first sought to confirm the reported reactivity of guaiazulene with oxalyl chloride and bromide. As anticipated, reaction of guaiazulene with oxalyl chloride in THF at 0 °C followed by quenching with isopropanol gave exclusively glyoxylate ester **7k** (Table 1, entry 1). Using oxalyl bromide under otherwise identical conditions facilitated decarbonylation to afford **8k** as the major product, though side-product **9** resulting from addition of THF was also observed in 36% yield (entry 6). This side-product was suppressed by using dioxane in place of THF (entry 7).

**Table 1:**
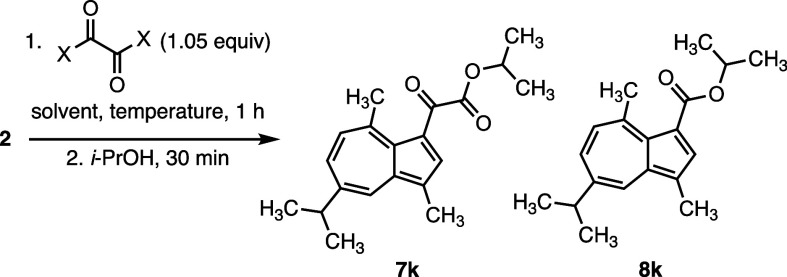
Optimization of Reaction Conditions

**entry**	**X**	**temp. (°C)**	**additive**	**solvent**	**yield (%)** ^b^	
					**7k**	**8k**
1	Cl	0	–	THF	87	0
2	Cl	25	–	THF	74	8
3	Cl	70	–	THF	0	93
4	Cl	25	–	dioxane	67	21
5	Cl	70	–	dioxane	0	93
6[Table-fn t1fn3]	Br	0	–	THF	3	62
7	Br	25	–	dioxane	0	97
8	Cl	25	LiBr (1 equiv)	dioxane	0	87
9	Cl	25	TBABr (1 equiv)	dioxane	0	92

a Yields determined by ^1^H NMR vs durene as an internal standard on 0.25 mmol scale.

b Compound **9**, resulting from reaction of THF with the acid bromide, was also detected in 36% NMR yield.
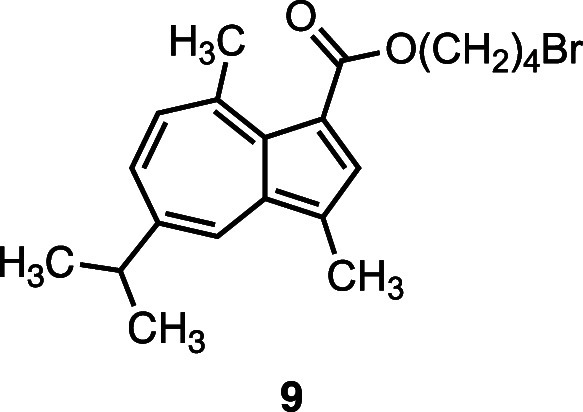

We hypothesized that an increased temperature would facilitate decarbonylation, and indeed, running the reaction with oxalyl chloride at 70 °C formed **8k** in high NMR yield (entries 3 and 5). We also found that exogenous bromide in the form of added LiBr could accelerate decarbonylation at lower temperature (entry 8), presumably via a similar mechanistic pathway to the reaction using oxalyl bromide. Similar results were observed using tetrabutylammonium bromide (TBABr), confirming that this effect results from bromide rather than the Li^+^ counterion (entry 9).

### Substrate Scope

With a divergent set of reaction conditions identified, we next examined the scope of the trapping nucleophile for the reaction of guaiazulene with oxalyl chloride at 0 °C vs 70 °C (Table 2). Using ammonium hydroxide as a nucleophile under the lower-temperature conditions was effective for forming primary amide **7a**, but under the decarbonylative conditions, ammonium hydroxide gave a complex mixture without **8a** evident in the crude ^1^H NMR spectrum. When the reaction was quenched instead with a dioxane solution of ammonia, **8a** was isolated in only 11% yield. We had previously found that **8a** can be prepared more efficiently by reaction of ammonium hydroxide with trichloromethyl ketone **5**.^32^ Both the acylative and decarbonylative reactions worked well when quenched with primary amines (**7b–d**, **8b–d**), including the hindered *tert*-butylamine. Cyclic and acyclic secondary amines were also effective at providing tertiary amide products **7e–g** and **8e–g**.

**Table 2:**
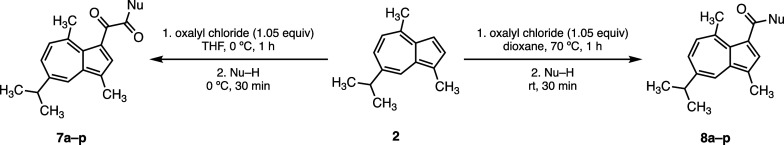

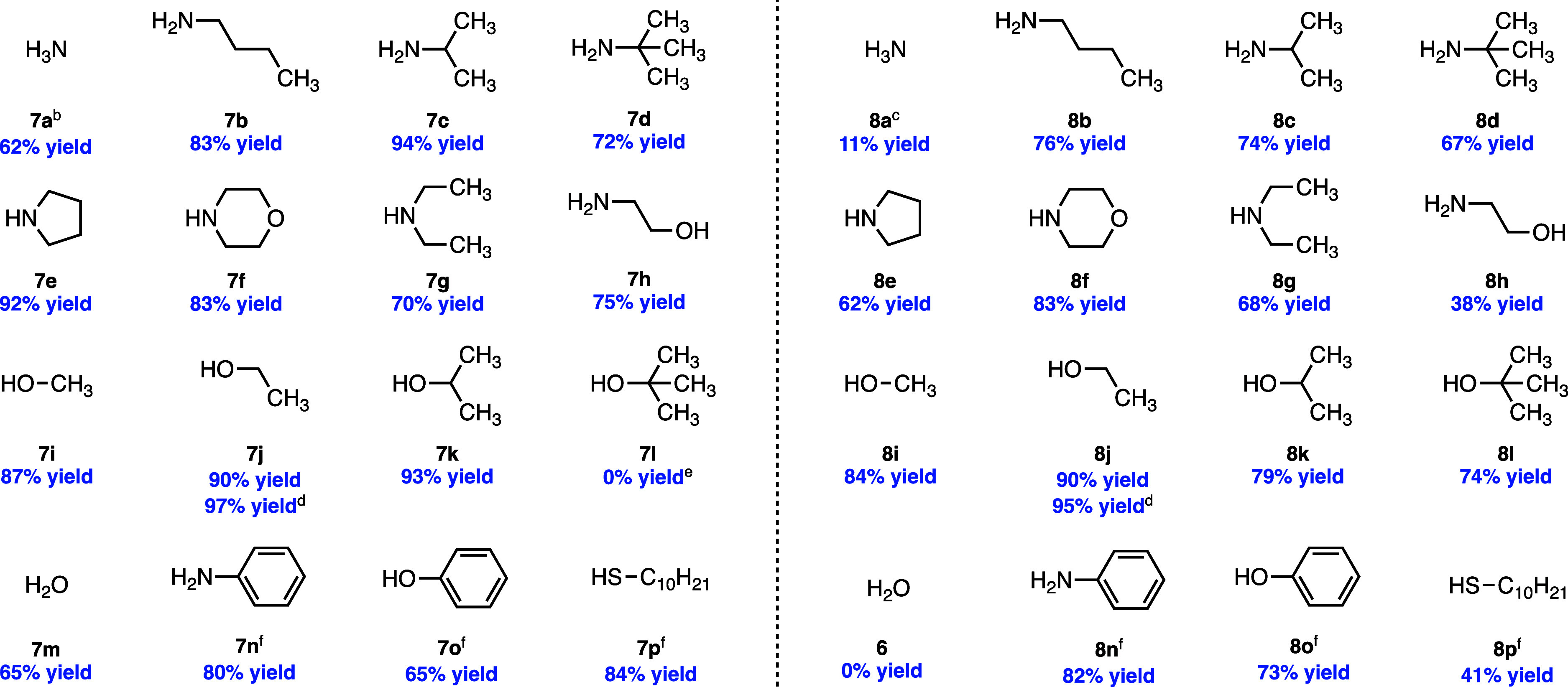
Reaction Scope with Guaiazulene^a^

a Isolated yields on 0.5 mmol scale.

b NH_4_OH (aq) was used as the trapping nucleophile.

c NH_3_ as a solution in dioxane was used as the trapping nucleophile.

d Isolated yield on 5 mmol scale.

e Compound **7m** was isolated in 62% yield.

f NEt_3_ was added as a base along with the trapping nucleophile for this substrate.

Methyl, primary, secondary, and tertiary alcohol nucleophiles gave the corresponding ester products (**7i–k**, **8i–l**), with the exception of *tert*-butanol in the non-decarbonylative reaction. In this case, oxalic acid derivative **7m** was isolated instead in 62% yield, presumably via an acid-mediated dealkylation. Oxalic acid **7m** could also be prepared in similar yield by quenching the reaction with water. On the other hand, when we quenched the decarbonylative reaction with water in an attempt to isolate the corresponding carboxylic acid **6**, we observed primarily the guaiazulene starting material **2**, consistent with our prior studies on hydrolysis of **5** that suggested the guaiazulene-3-carboxylic acid is unstable. In the presence of triethylamine as a base, aniline, phenol, and decanethiol were also competent nucleophiles, yielding the corresponding *N*-phenylamide (**7n**, **8n**), phenyl ester (**7o**, **8o**), and thioester (**7p**, **8p**) products under both sets of reaction conditions.

When we attempted to apply these conditions to functionalizing azulene instead of guaiazulene, we found that the initial electrophilic aromatic substitution and the decarbonylation were both significantly slower (see below). By modifying the reaction times/temperatures, we were able to isolate amide and ester products derived from azulene with or without decarbonylation (**10a–10b**, **11a–11b**, Scheme 3). We anticipate that these modified conditions for azulene are applicable to a wide range of amine and alcohol nucleophiles.

**Scheme 3:**
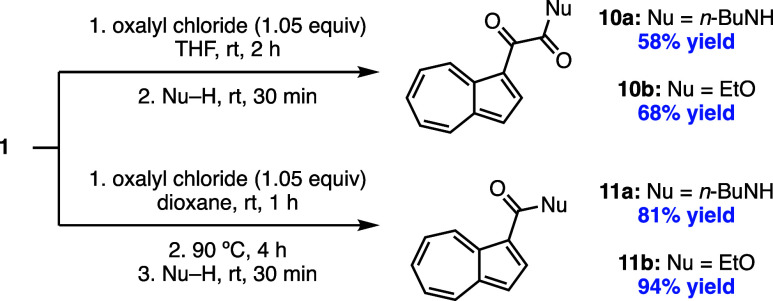
Reaction Scope with Azulene^a^

a Isolated yields on 0.25 mmol scale.

### Mechanistic Studies

Given that bromide accelerates the loss of CO (see Table 1), we initially postulated a halide-mediated pathway for decarbonylation (Scheme 4a). In this mechanism, reaction of **1** or **2** with oxalyl chloride or bromide would first form electrophilic aromatic substitution (EAS) product **3**. Next, rate-determining attack of X^–^ on the ketone of **3** would generate tetrahedral intermediate **12**, which then collapses with loss of CO and X^–^ to form acid halide **4**. If this mechanism held, we would expect the more nucleophilic guaiazulene to react faster than azulene in the initial EAS, consistent with our qualitative observations. However, the alkyl substituents in guaiazulene might be anticipated to make the ketone in **3** less reactive toward nucleophilic attack via hyperconjugative donation to the π-system, which was seemingly inconsistent with the faster decarbonylation observed for guaiazulene.

**Scheme 4:**
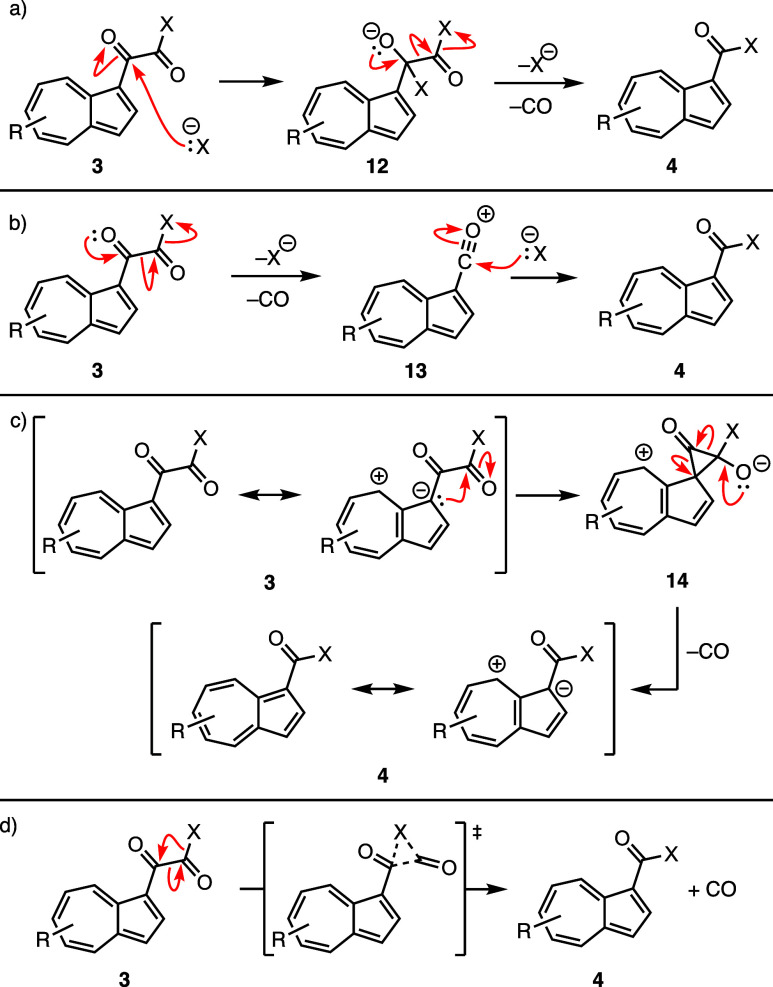
Possible Decarbonylation Mechanisms

To gain insight into the mechanism, we monitored the decarbonylation using no-D ^1^H NMR spectroscopy in dioxane solvent at a variety of temperatures for the reactions of guaiazulene and azulene with oxalyl chloride, as well as the reaction of azulene with oxalyl bromide (Figure 2 and Supporting Information). Note that species are designated as “a” or “g” for reactions with azulene or guaiazulene, respectively. We were also able to obtain no-D ^13^C­{^1^H} NMR spectra for the acylated species (**3g-Cl**, **3a-Cl**, **3a-Br**) and decarbonylation products (**4g-Cl**, **4a-Cl**, **4a-Br**, see Supporting Information for details), confirming the loss of a carbonyl carbon over the course of the reaction. In contrast, the reaction of guaiazulene with oxalyl bromide was too rapid to monitor at or near rt via no-D NMR spectroscopy. However, we were able to obtain kinetic data in dioxane for the decarbonylation of both **3g-Cl** at 35 °C and **3g-Br** at rt using in situ IR spectroscopy (Figure 3a and Supporting Information). We attempted to study the reaction of **3g-Br** at 0 °C in THF using in situ IR spectroscopy, but these measurements were complicated by overlapping C=O stretching signals, presumably arising from the formation of THF addition products like **9** (see Table 1, entry 6).

**Figure 2:**
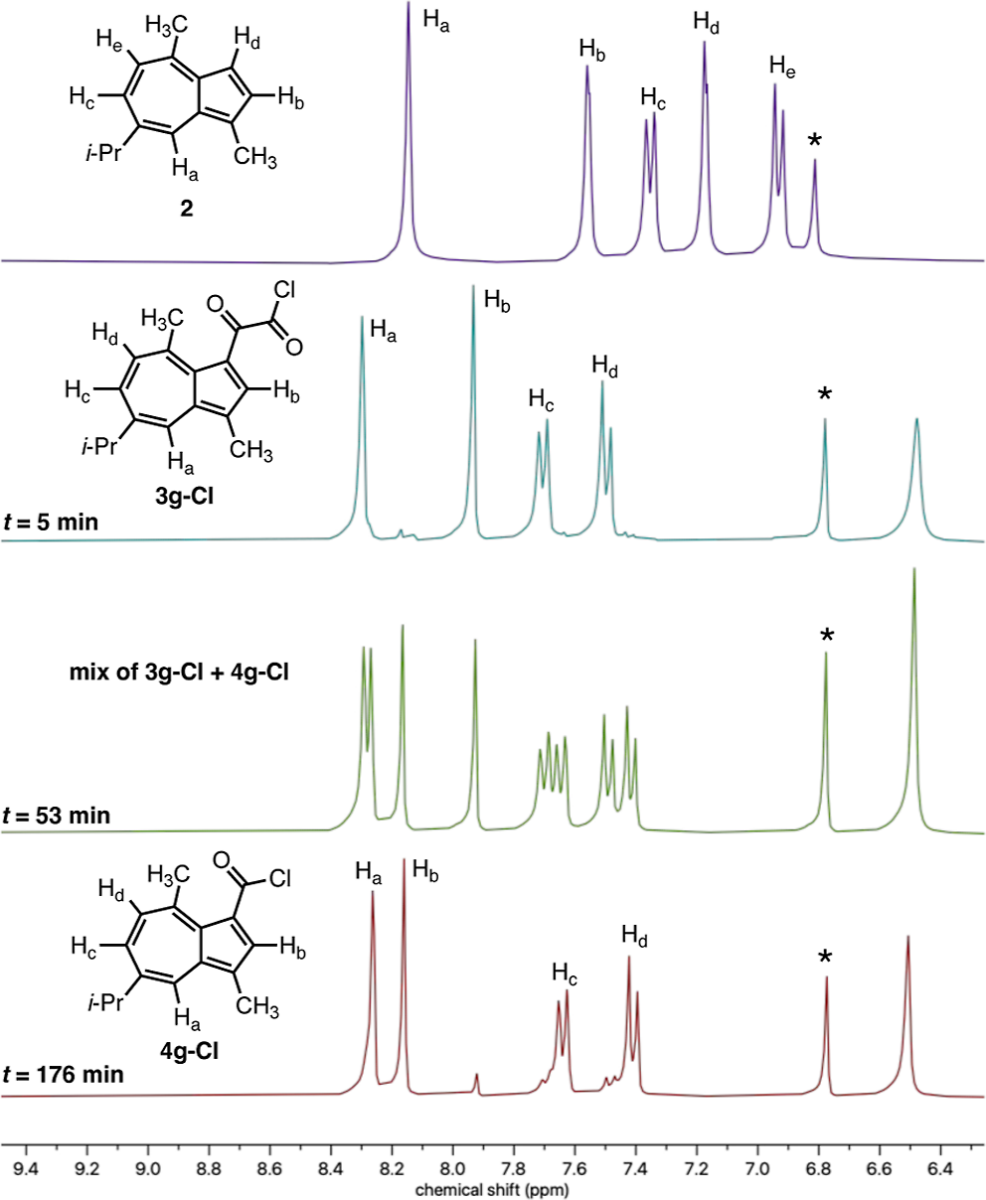
Example no-D ^1^H NMR spectra for reaction of guaiazulene **2** with oxalyl chloride at 35 °C in dioxane. The asterisks mark a signal for the durene internal standard. The signal at δ 6.5 ppm most likely arises from protonated dioxane, formed in the initial EAS reaction. See Supporting Information for spectra of other substrates.

**Figure 3:**
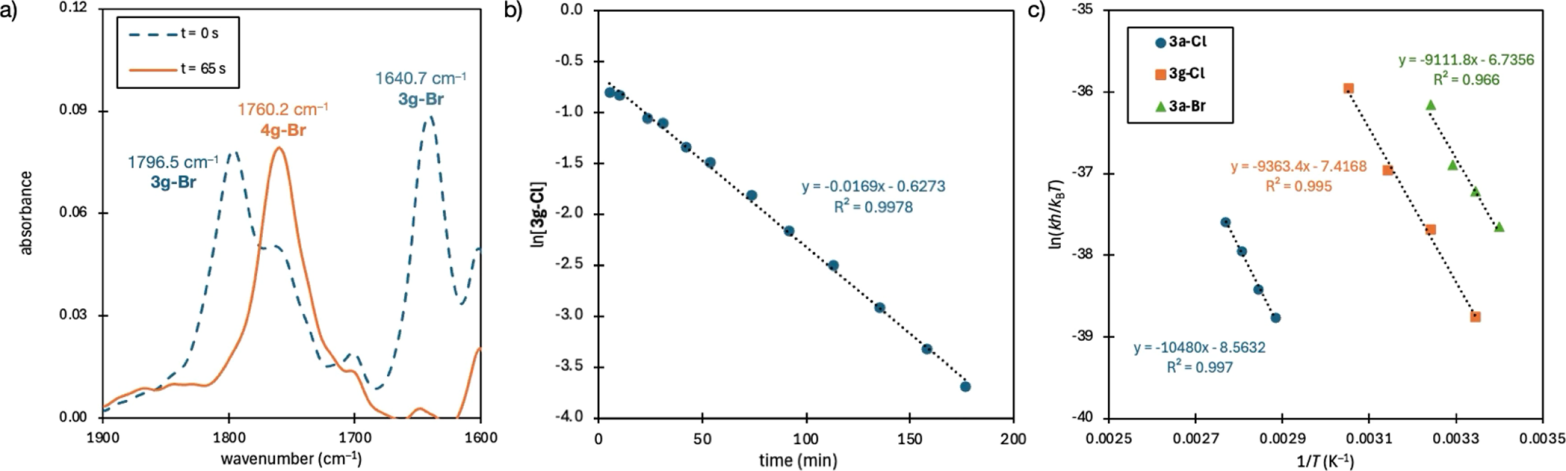
(a) Example in situ IR spectra for decarbonylation of **3g-Br** in dioxane at rt. (b) First-order kinetic plot for decarbonylation of guaiazulene derivative **3g-Cl** at 35 °C based on the no-D ^1^H NMR data in Figure 2. (c) Eyring plot for decarbonylation of **3a-Cl**, **3g-Cl**, and **3a-Br**.

The decarbonylation reactions of **3g-Cl**, **3a-Cl**, **3a-Br**, and **3g-Br** exhibited first-order kinetics for the disappearance of starting material at all temperatures examined (Figure 3b and Supporting Information). Importantly, we measured similar rate constants for the decarbonylation of **3g-Cl** at 35 °C via no-D NMR and in situ IR spectroscopy (0.0169 and 0.0148 min^–1^, respectively), confirming our ability to compare results across these two methods. Consistent with our prior qualitative observations, the decarbonylation was much more rapid for guaiazulene derivative **3g-Cl** (Table 3, entries 5–8) compared to azulene derivative **3a-Cl** (entries 1–4). We also observed a significant rate acceleration for decarbonylation of bromide derivatives **3a-Br** (entries 9–12) and **3g-Br** (entry 13) compared to their chloride analogues.

**Table 3:** Kinetic Data for Decarbonylation Reactions^a^

entry	substrate	temp. (°C)	kobs (min^–1^)	t_1/2_ (min)
1	**3a‑Cl**	75	0.0063	110.0
2	**3a‑Cl**	80	0.0091	76.2
3	**3a‑Cl**	85	0.0147	47.2
4	**3a‑Cl**	90	0.0213	32.5
				
5	**3g‑Cl**	25	0.0055	126.0
6	**3g‑Cl**	35	0.0169	41.5
7	**3g‑Cl**	45	0.0353	19.6
8	**3g‑Cl**	55	0.0996	7.0
				
9	**3a‑Br**	20	0.0163	42.5
10	**3a‑Br**	25	0.0258	26.9
11	**3a‑Br**	30	0.0360	19.3
12	**3a‑Br**	35	0.0763	9.1
				
13^b^	**3g‑Br**	rt	2.4690	0.28

a Determined by no-D ^1^H NMR spectroscopy, except for entry 13.

b Determined by in situ IR spectroscopy.

The observation of first-order kinetics does not rule out a halide-mediated decarbonylation pathway. For the mechanism depicted in Scheme 4a, the concentration of halide should remain constant throughout, leading to pseudo-first-order kinetics. However, no-D NMR experiments showed nearly identical initial rates for the decarbonylation of **3g-Cl** at 25 °C in the absence/presence of 1.1 equiv LiCl (−0.118 vs –0.123 M/h, respectively). Similar initial rates were also observed for the decarbonylation of **3a-Br** at 25 °C in the absence/presence of 1.1 equiv LiBr (−0.89 vs −0.82 M/h, respectively). These results suggest that free halide does not catalyze the decarbonylation reaction and argue against the addition–elimination mechanism in Scheme 4a.^45^

As an alternative, we considered the unimolecular mechanism depicted in Scheme 4b, where rate-determining loss of halide and CO generates acylium ion **13**. Recombination with X^–^ then yields acid halide **4**. In this mechanism, the aromatic π-system could be envisioned as stabilizing the transition state leading to **13**, explaining why the more nucleophilic **3g-Cl** decarbonylates at a higher rate than **3a-Cl**. If this pathway was operative, we would expect to observe a large, positive entropy of activation due to the release of both X^–^ and CO in the rate-determining step. However, an Eyring analysis was inconsistent with this mechanism, instead giving a substantially *negative* Δ*S* ^‡^ for the decarbonylation of all three substrates (Figure 3c, Table 4).^46,47^

**Table 4:** Eyring Parameters for Decarbonylation Reactions

entry	substrate	Δ*H* ^‡^ (kcal/mol)	Δ*S* ^‡^ (cal/mol K)	Δ*G* ^‡^ at 25 °C (kcal/mol)
1	**3a‑Cl**	+20.8	–17.0	+25.9
2	**3g‑Cl**	+18.6	–14.7	+23.0
3	**3a‑Br**	+18.1	–13.4	+22.1

A mechanism that fit all of the data would need to include a step that is unimolecular with a negative Δ*S* ^‡^. These two features are difficult to reconcile together except through some sort of intramolecular rate-determining step. Because our kinetic data would not allow us to distinguish between various intramolecular pathways, we turned to computational chemistry to assess the possibilities.

We first used density functional theory (DFT) calculations to evaluate the mechanisms previously ruled out by our experimental data (Scheme 4a,b). Indeed, our potential energy surface scans showed implausibly high barriers (>60 kcal/mol) with **3a-Cl** for both pathways. As an alternative, we proposed the mechanism shown in Scheme 4c, in which the EAS adduct **3** undergoes intramolecular nucleophilic attack by the π-system on the electrophilic acyl halide, leading to spirocyclic tetrahedral intermediate **14**. Collapse of **14** with release of CO and X^–^ then affords acyl halide **4**. However, our calculations for this proposed pathway indicated a similarly prohibitive barrier of 62.2 kcal/mol (see **TS-S2** in Supporting Information), inconsistent with the experimentally determined Δ*G* ^‡^ of 25.9 kcal/mol for the reaction with **3a-Cl**.

We next considered the concerted 1,2-chloride shift mechanism shown in Scheme 4d. This proposal was inspired by calculations that showed accessible conformations of **3a-Cl** in which the two carbonyl groups become perpendicular rather than coplanar. In this conformation, the C–Cl σ-bond is in parallel alignment with the ketone C=O π* orbital, opening up the possibility of a concerted 1,2-chloride shift with concurrent ejection of CO. Stepwise variations of this mechanism were computationally ruled out, as a tetrahedral intermediate with CO still attached was not an energy minimum on the potential energy surface, instead ejecting CO upon optimization.

The concerted 1,2-chloride shift process (via **TS-1**) was calculated to have an activation barrier of 31.1 kcal/mol for **3a-Cl** (Figure 4). While the calculated absolute value of this barrier is higher than the experimentally determined Δ*G* ^‡^ of 25.9 kcal/mol, no other mechanisms we explored had computed barriers of less than 60 kcal/mol. Later calculations also showed that this mechanism captures the relative reactivity trends well (see below), lending support to its involvement in the reaction. While the predicted sign of Δ*S* ^‡^ for this mechanism was not obvious *a priori*, a negative entropy of activation is plausible due to the reduced conformational degrees of freedom in transition state **TS-1**. For comparison, the hydrolysis of 2-chloroethyl methyl sulfide proceeds via rate-determining, unimolecular episulfonium ion formation with concurrent expulsion of chloride, and this process was reported to have Δ*S* ^‡^ = −20.8 cal/mol•K in 0.10 mole fraction acetone/water solvent.^48^

**Figure 4:**
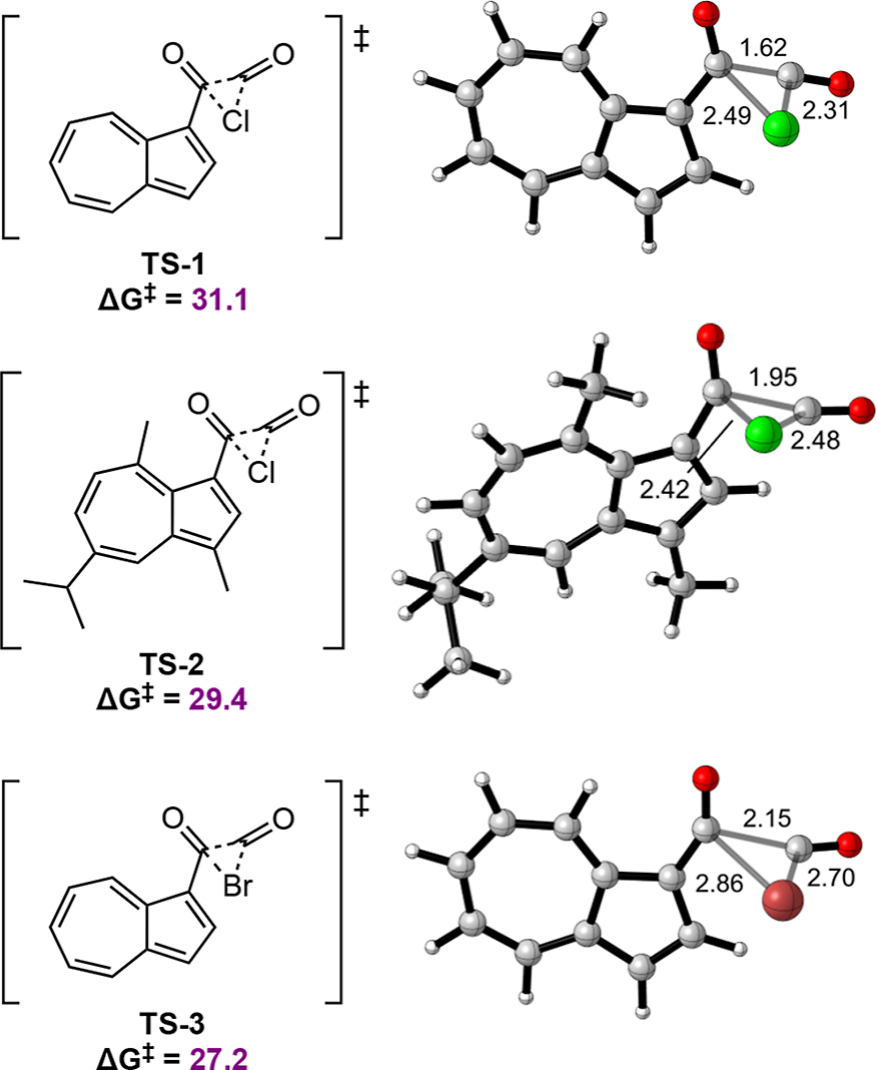
Calculated structures and activation free energies (kcal/mol) of 1,2-halide shift transition states for **3a-Cl**, **3g-Cl**, and **3a-Br** at the M06-2X/def2-TZVPP, SMD­(1,4-dioxane)//M06-2X/def2-SVP, SMD­(1,4-dioxane) level of theory. Interatomic distances are in Å.

A further question is how this concerted 1,2-chloride shift mechanism would account for the faster decarbonylation with **3g-Cl** compared to **3a-Cl**. This trend appeared counterintuitive, as the more electron-rich guaiazulene π-system would be expected to render the ketone in **3g-Cl** less electrophilic. However, our calculations reproduced this experimental trend well by revealing an activation barrier of 29.4 kcal/mol (via **TS-2**) for the 1,2-chloride shift in **3g-Cl**, 1.7 kcal/mol lower than for **3a-Cl**. Calculations also showed that this difference in activation barriers appears to arise not from the electronics of the azulene ring, but rather a conformational effect. In the ground state of **3g-Cl**, the 4-methyl substituent forces the ketone to rotate out of the plane of the ring (C=C–C=O dihedral angles of 3.4° and 18.4° for **3a-Cl** and **3g-Cl**, respectively), which reduces the conjugation of the ketone and presumably renders it more electrophilic.

To obtain further insight, we expanded our computational investigation to the concerted 1,2-shift of other groups in this system. Our calculations predicted that the 1,2-bromide shift in **3a-Br** would proceed with a 27.2 kcal/mol activation barrier, 3.9 kcal/mol lower than the 1,2-chloride shift in **3a-Cl**. This result is in excellent agreement with the experimentally measured 3.8 kcal/mol decrease in Δ*G* ^‡^ between the reactions of **3a-Br** and **3a-Cl** (Table 4). We noted that the calculated C–C bond length between the two carbonyl carbons increases from **TS-1** to **TS-2** to **TS-3** and correlates well with the experimental Δ*S* ^‡^ values for **3a-Cl**, **3g-Cl**, and **3a-Br** (see Figure S49 in the Supporting Information). This C–C bond length can be taken to represent the progress of the decarbonylation and an indication of which substrates exhibit a relatively early or late transition state. It makes sense that **3a-Br**, with the longest intercarbonyl bond length and hence the latest transition state in **TS-3**, has the least negative Δ*S* ^‡^, as the release of CO is anticipated to be the main positive contributor to the entropy change for this process.

Additional calculations indicated that 1,2-migrations of other groups, including NH_2_ and OH, would have prohibitive barriers >60 kcal/mol (see Supporting Information), consistent with our ability to isolate these and other amides/esters without observing decarbonylation. Overall, the combined experimental and computational evidence provides the strongest support for a concerted 1,2-halide shift mechanism.

## Conclusion

To summarize, we investigated the functionalization of guaiazulene with oxalyl chloride and the reactivity of the resulting EAS adduct. We found that at 0 °C, quenching with a nucleophile yields guaiazulene-3-oxalic acid derivatives, while heat or added bromide can induce decarbonylation, enabling synthesis of guaiazulene-3-carboxylic acid derivatives. Experimental and computational investigations are most consistent with an unexpected 1,2-chloride shift mechanism for the decarbonylation. The reaction conditions we describe can be modified for use with the parent azulene, suggesting that oxalyl chloride is a suitable replacement for phosgene and oxalyl bromide in the functionalization of a range of azulene derivatives.

## Supplementary Material





## Data Availability

The data underlying this study are available in the published article and its Supporting Information.

## Notes

Text file of all computed molecule Cartesian coordinates in a format for convenient visualization (XYZ)
Experimental procedures, characterization data, kinetics plots, theoretical calculations, and NMR spectra (PDF)

## ∥

Department of Chemistry, Carnegie Mellon University, Pittsburgh, PA, 15213, United States
