# Supplementary material for: Decarbonylation Enables Chemodivergent Access to Guaiazulene-3-Oxalic and ‑Carboxylic Acid Derivatives
Source: J Org Chem. 2026 Jun 22;91(26):9134–41. doi: 10.1021/acs.joc.6c00848 (PMC13339644; doi:10.1021/acs.joc.6c00848)
Supplement: Supplementary file 2 [file jo6c00848_si_002.pdf]

Supporting Information

Decarbonylation Enables Chemodivergent Access to Guaiazulene-3-Oxalic and -Carboxylic Acid Derivatives

Emma Audi,[a] Carly M. Zack,[a] Esther M. Ntankwa,[a] William Blake Heston,[a] Annika S. Waples,[b] Manana Tsiskarishvili,[c] C. Rose Kennedy,[c] Shuming Chen,*,[b] and J. Patrick Lutz*,[a]

[a] Department of Chemistry, St. Lawrence University, Canton, NY, 13617, United States

[b] Department of Chemistry and Biochemistry, Oberlin College, Oberlin, Ohio 44074, United States

[c] Department of Chemistry, University of Rochester, Rochester, NY, 14627, United States

*E-mail: schen3@oberlin.edu, jlutz@stlawu.edu

I. General Information S-2
II. Experimental Section S-4
  a. General Synthetic Procedures S-4
  b. Characterization of Compounds S-6
  c. General Kinetics Procedures S-38
  d. Kinetics Data for Guaiazulene Reactions with Oxalyl Chloride S-43
  e. Kinetics Data for Azulene Reactions with Oxalyl Chloride S-55
  f. Kinetics Data for Azulene Reactions with Oxalyl Bromide S-64
  g. NMR Data for Acyl Halides S-76
  h. *In Situ* IR Studies S-79
III. Computational Section S-87
  a. Computational Results S-87
  b. Calculated Energies S-91
  c. Cartesian Coordinates of Calculated Structures S-92
IV. References S-97
V. NMR Spectra S-98

I. General Information

Materials and methods. Commercial reagents were purchased from Sigma Aldrich, Oakwood, Ambeed, or Combi-Blocks, and used as received. Solvents were dried over activated 3 Å molecular sieves. Reactions were run in closed vials under an air atmosphere.

Instrumentation. Nuclear magnetic resonance spectra were recorded on a JEOL ECZS 400 MHz NMR spectrometer. Proton chemical shifts are reported in parts per million downfield from tetramethylsilane. Chemical shifts for conventional ^{1}H NMR spectra are referenced to residual protium in the NMR solvent ($CDCl_3$ = δ 7.26 ppm, C_6D_6 = δ 7.16 ppm). Chemical shifts for conventional ^{13}C NMR spectra are reported in parts per million downfield from tetramethylsilane and are referenced to the carbon resonances of the solvent residual peak ($CDCl_3$ = δ 77.16 ppm). No-D NMR spectra were recorded in dioxane and are left unreferenced. NMR data are represented as follows: chemical shift (δ ppm), multiplicity (s = singlet, d = doublet, t = triplet, q = quartet, sept = septet, m = multiplet), coupling constant in Hertz (Hz), integration. Some structural assignments were made with additional information from gCOSY, gHSQC, and gHMQC experiments. For kinetic measurements, the NMR probe temperature was calibrated with ethylene glycol to give a best-fit line of [corrected temp] = 0.96[set temp] + 1.95 ºC.[1]

Mass spectra were acquired at Cornell University on a DART-SVP (Direct Analysis in Real Time) ion source (IonSense, Saugus, MA) coupled to an Exactive Orbitrap mass spectrometer (Thermo Scientific, Bremen, Germany).

Fourier-tranform infrared (FT-IR) spectra were recorded on a Nicolet 4700 IR spectrometer with a Pike MIRacle ATR attachment and are reported in terms of frequency of absorption (cm^{-1}).

In situ infrared spectra were recorded using a ReactIR 700 spectrometer (SN: C135474708) equipped with a liquid nitrogen–cooled MCT detector. Apodization was applied using the **Norton-**Beer Medium function. The probe employed was a SiComp (Silicon) probe, SN: C137588709, interfaced via a 6 mm x 1.5 m AgX fiber (Silver Halide). Spectra were collected over a wavenumber range of 3000–650 cm^{-1} with a spectral resolution of 8 cm^{-1}. Scanning was performed using the AutoSelect option, and the detector gain was set to low. Kinetic absorbance–time data were analyzed in MATLAB (R2023b, MathWorks, Natick, MA, USA). Linear least-squares regression (polyfit) was applied to zero-, first, and second-order integrated rate law plots ([A] vs t, ln[A] vs t, and 1/[A] vs t). Rate constants were extracted from the fitted slopes. Coefficients of determination (R^2) were calculated from residual sum-of-squares analysis, and

half-lives were determined using standard integrated rate expressions. The best-fit kinetic order was assigned based on the highest R^2 value.

Computational methods. All density functional theory (DFT) computations were performed using Gaussian 16, Revision A.03.[2] Molecular geometries were optimized using the M06-2X[3] functional, using the def2-SVP basis set for all atoms. Frequency calculations were performed at the same level of theory as that used for geometry optimization to confirm the stationary points as either minima (no imaginary frequencies) or first-order saddle points (one imaginary frequency) on the potential energy surface. Intrinsic Reaction Coordinate (IRC) calculations were performed to confirm the first-order saddle points as actual transition states connecting the expected reactants and products. Thermal contributions to free energies were calculated from vibrational frequencies using the quasi-rigid rotor-harmonic oscillator (RRHO) approach of Grimme[4] implemented through Paton's GoodVibes[5] Python script with the temperature set to 343 K. Single-point energies (SPEs) were calculated with the M06-2X functional, using the def2-TZVPP basis set for all atoms. Solvation effects were incorporated into single-point energy calculations using Truhlar's SMD[6] model at both the optimization/frequency analysis level and the single-point energy refinement level of theory, with 1,4-dioxane as the solvent. Visualizations of molecular structures were obtained using CYLview.[7] Reiterative Monte Carlo conformational searches were performed with the Merck molecular force field (MMFF) implemented in Spartan ’24.[8] All conformers within 10.0 kcal/mol of the lowest-energy conformer through Monte Carlo searches were reoptimized and evaluated for free energy in Gaussian using the DFT methods described above.

Safety warning: The decarbonylative functionalization involves generation of carbon monoxide as a byproduct, with significant quantities being produced on scale-up. Carbon monoxide is a highly toxic gas that is extremely harmful if inhaled. Therefore, all of these reactions MUST be performed in a well-ventilated fume hood.

II. Experimental Section

II.a. General Synthetic Procedures

General Procedure A: Non-Decarbonylative Functionalization of Guaiazulene

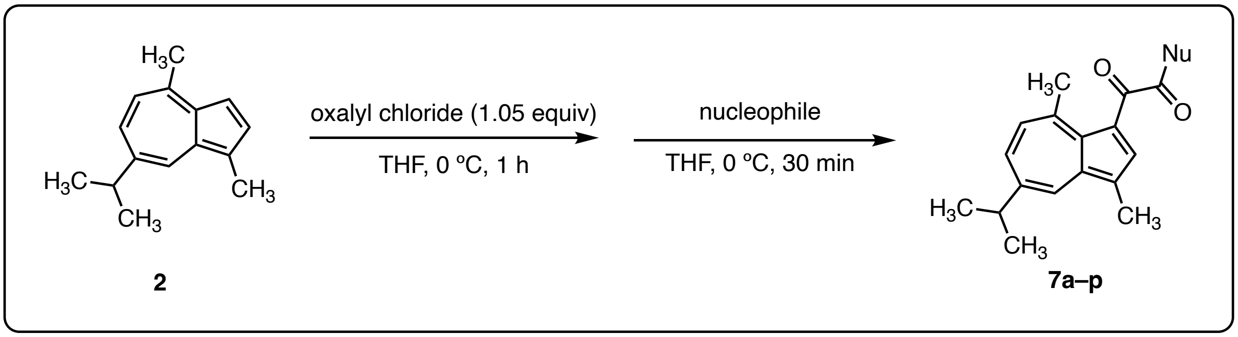

A 1-dram vial was charged with a stir bar, guaiazulene (100. mg, 0.504 mmol, 1.00 equiv), and THF (1 mL, dried over sieves). The vial was cooled to 0 °C in an ice/water bath, oxalyl chloride (45.0 µL, 0.532 mmol, 1.05 equiv) was added *via* syringe, and the reaction was stirred at 0 °C for 1 h. The chosen nucleophile was added was added (typically 4.0 equiv for amines or 0.5 mL for alcohols), and the reaction was stirred for an additional 30 min at 0 °C. The contents of the reaction vial were transferred to a separatory funnel with water (30 mL) and ethyl acetate (30 mL). The layers were separated, then the aqueous layer was extracted with additional ethyl acetate (2 x 30 mL). The combined organic extracts were dried over $MgSO_4$, filtered, and concentrated on the rotary evaporator. The resulting crude material was purified *via* column chromatography on silica to give the target compound.

General Procedure B: Decarbonylative Functionalization of Guaiazulene

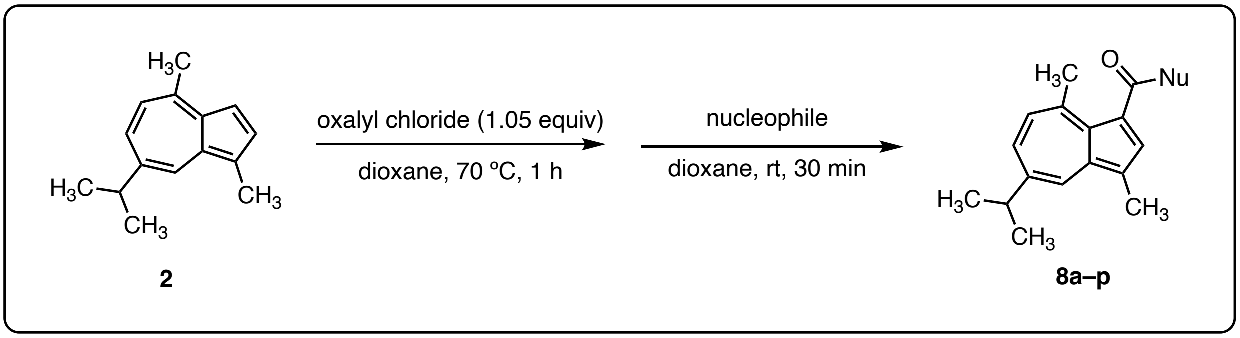

A 1-dram vial was charged with a stir bar, guaiazulene (100. mg, 0.504 mmol, 1.00 equiv), and dioxane (1 mL, dried over sieves). At rt, oxalyl chloride (45.0 µL, 0.532 mmol, 1.05 equiv) was

added *via* syringe, then the vial was transferred to an oil bath at 70 °C and stirred at this temperature for 1 h. The vial was removed from the oil bath and transferred to an ice/water bath, then the chosen nucleophile was added was added (typically 4.0 equiv for amines or 0.5 mL for alcohols). The vial was removed from the cold bath and stirred at rt for 30 min. The contents of the reaction vial were transferred to a separatory funnel with water (30 mL) and ethyl acetate (30 mL). The layers were separated, then the aqueous layer was extracted with additional ethyl acetate (2 x 30 mL). The combined organic extracts were dried over $MgSO_4$, filtered, and concentrated on the rotary evaporator. The resulting crude material was purified *via* column chromatography on to give the target compound.

II.b Characterization of Compounds

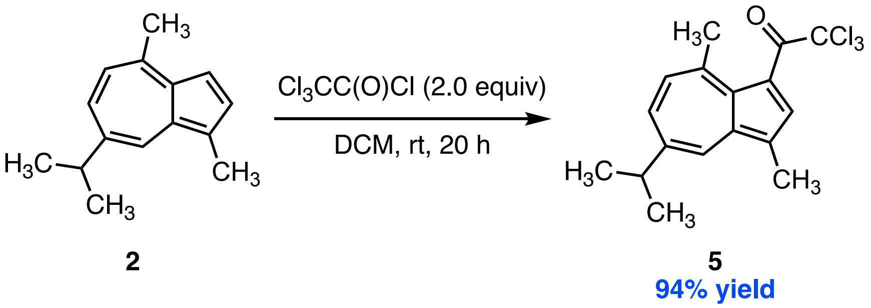

2,2,2-Trichloro-1-(5-isopropyl-3,8-dimethylazulen-1-yl)ethan-1-one (5). A 100-mL round-bottomed flask was charged with a stir bar, guaiazulene (1.50 g, 7.56 mmol, 1.00 equiv), DCM (15 mL), and trichloroacetyl chloride (1.70 mL, 15.2 mmol, 2.01 equiv). The reaction was stirred at rt for 20 h, then quenched with sat. aq. $NaHCO_3$ (20 mL). The mixture was extracted with DCM (3 x 15 mL), and the combined organic extracts were dried over $MgSO_4$, filtered, and concentrated on the rotary evaporator to give a brown oil. The crude material was purified *via* column chromatography (silica, 5% EtOAc in hexanes) to afford **5** as a dark brown solid (2.18 g, 7.15 mmol, 94% yield).

R_f: 0.41 in 5% EtOAc in hexanes.

^{1}H NMR (400 MHz, $CDCl_3$): δ 8.31 (s, 1H), 8.30 (d, J = 2.3 Hz, 1H), 7.66 (dd, J = 10.9, 2.3 Hz, 1H), 7.45 (d, J = 10.9 Hz, 1H), 3.17 (hept, J = 6.9 Hz, 1H), 2.82 (s, 3H), 2.61 (s, 3H), 1.39 (d, J = 7.0 Hz, 6H) ppm.

^{13}C{^{1}H} NMR (125 MHz, $CDCl_3$): δ 180.3, 148.8, 147.1, 142.7, 141.2, 140.2, 136.8, 134.6, 133.5, 124.6, 116.7, 97.6, 38.3, 28.6, 24.7, 13.1 ppm.

HRMS: (DART/ORBITRAP) calculated for $C_{17}H_{18}Cl_3O^+$ ($[M+H]^+$): 343.0418, found: 343.0419.

FTIR (ATR, cm^{-1}): 2958 (w), 2925 (w), 2865 (w), 1668 (m).

mp: 87–90 ºC

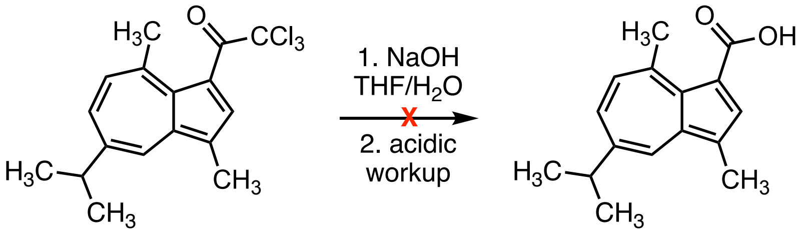

5 6

5-Isopropyl-3,8-dimethylazulene-1-carboxylic acid (6). A 1-dram vial was charged with a stir bar, trichloromethyl ketone **5** (50.0 mg, 0.145 mmol, 1.00 equiv), THF (0.5 mL), and 5% aq. NaOH (1 mL). The reaction was stirred at 60 ºC for 1 h, at which point TLC indicated complete conversion of **5** and formation of a new purple spot at R_f = 0 in 5% EtOAc in hexanes. The reaction was acidified by dropwise addition of 1 M HCl until the pH read 2–3 by pH paper. The mixture was transferred to a separatory funnel with water (15 mL) and EtOAc (15 mL). The layers were separated, then the aqueous layer was extracted with additional EtOAc (2 x 15 mL). The combined organic fractions were dried over $MgSO_4$, filtered, and concentrated on the rotary evaporator to give a dark blue solid. The crude material was purified *via* column chromatography (silica, 100% hexanes) to afford a blue oil that was identified by ^1H NMR as guaiazulene **2** (24.3 mg, 0.123 mmol, 85% yield).

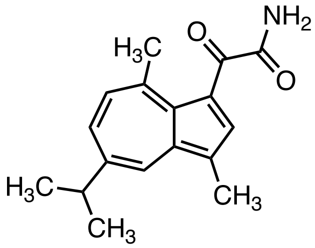

7a

2-(5-Isopropyl-3,8-dimethylazulen-1-yl)-2-oxoacetamide (7a). Prepared according to **General Procedure A** using ~30% aq. NH_4OH (0.15 mL, ~1.8 mmol) as the nucleophile. The crude product was purified *via* column chromatography (silica, 50% EtOAc in hexanes) to afford **7a** as a black/dark blue solid (83.8 mg, 0.311 mmol, 62% yield).

***R_f*:** 0.17 in 30% EtOAc in hexanes; 0.52 in 70% EtOAc in hexanes.

^{1}H NMR (400 MHz, CDCl$_3$): δ 8.35 (s, 1H), 8.27 (d, *J* = 2.1 Hz, 1H), 7.64 (dd, *J* = 11.0, 2.1 Hz, 1H), 7.45 (d, *J* = 11.0 Hz, 1H), 7.22 (broad s, 1H), 5.80 (broad s, 1H), 3.16 (hept, *J* = 6.9 Hz, 1H), 2.90 (s, 3H), 2.57 (s, 3H), 1.39 (d, *J* = 6.9 Hz, 6H) ppm.

^{13}C{^{1}H} NMR (101 MHz, CDCl$_3$): δ 181.5, 166.1, 149.4, 147.4, 144.1, 144.0, 140.7, 136.7, 134.4, 133.8, 125.6, 121.7, 38.3, 28.8, 24.6, 13.0 ppm.

HRMS: (DART/ORBITRAP) calculated for $C_{17}H_{20}NO_2^+$ ([M+H]$^+$): 270.1489, found: 270.1489.

FTIR (ATR, cm^{-1}): 3434 (w), 3391 (m), 3282 (w), 3188 (w), 2956 (w), 2925 (w), 2864 (w), 1692 (m), 1620 (m).

mp: 172–174 °C

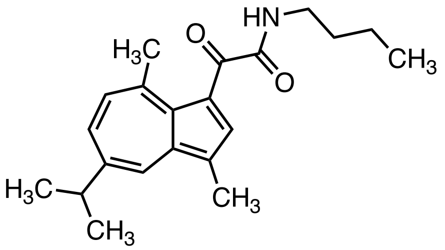

7b

***N*-Butyl-2-(5-isopropyl-3,8-dimethylazulen-1-yl)-2-oxoacetamide (7b).** Prepared according to **General Procedure A** using *n*-butylamine (0.20 mL, 2.0 mmol, 4.0 equiv) as the nucleophile. The crude product was purified *via* column chromatography (silica, 10% EtOAc in hexanes) to afford **7b** as a brown oil that solidified upon standing (136.7 mg, 0.420 mmol, 83% yield).

***R*$_f$:** 0.58 in 30% EtOAc in hexanes.

^{1}H NMR (400 MHz, CDCl$_3$): δ 8.39 (s, 1H), 8.26 (d, *J* = 2.1 Hz, 1H), 7.63 (dd, *J* = 11.0, 2.1 Hz, 1H), 7.43 (d, *J* = 11.0 Hz, 1H), 7.36 (t, *J* = 5.9 Hz, 1H), 3.42 (q, 2H), 3.15 (hept, *J* = 6.9 Hz, 1H), 2.88 (s, 3H), 2.56 (s, 3H), 1.67 – 1.55 (m, 2H), 1.51 – 1.40 (m, 2H), 1.38 (d, *J* = 6.9 Hz, 6H), 0.97 (t, *J* = 7.3 Hz, 3H) ppm.

^{13}C{^{1}H} NMR (101 MHz, CDCl$_3$): δ 182.2, 163.7, 149.2, 147.1, 144.4, 143.8, 140.6, 136.5, 134.3, 133.6, 125.4, 122.1, 39.4, 38.3, 31.6, 28.7, 24.6, 20.3, 13.9, 12.0 ppm.

HRMS: (DART/ORBITRAP) calculated for $C_{21}H_{28}NO_2^+$ ([M+H]$^+$): 326.2115, found: 326.2114.

FTIR (ATR, cm^{-1}): 3339 (w), 2957 (m), 2929 (m), 2868 (w), 1672 (w), 1638 (s).

mp: 72–76 °C

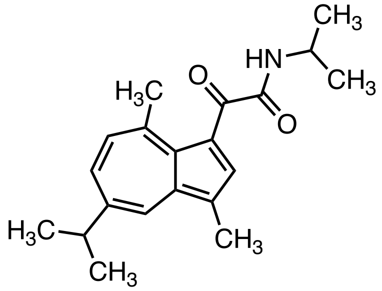

7c

***N*-Isopropyl-2-(5-isopropyl-3,8-dimethylazulen-1-yl)-2-oxoacetamide (7c).** Prepared according to **General Procedure A** using isopropylamine (0.17 mL, 2.0 mmol, 4.0 equiv) as the nucleophile. The crude product was purified *via* column chromatography (silica, 30% EtOAc in hexanes) to afford **7c** as a brown oil that solidified upon standing (147.9 mg, 0.475 mmol, 94% yield).

***R*$_f$:** 0.50 in 30% EtOAc in hexanes; 0.69 in 70% EtOAc in hexanes.

^{1}H NMR (400 MHz, CDCl$_3$): δ 8.39 (s, 1H), 8.26 (d, *J* = 2.1 Hz, 1H), 7.62 (dd, *J* = 11.0, 2.1 Hz, 1H), 7.42 (d, *J* = 11.0 Hz, 1H), 7.19 (d, *J* = 8.3 Hz, 1H), 4.29 – 4.12 (m, 1H), 3.15 (hept, *J* = 6.9 Hz, 1H), 2.88 (s, 3H), 2.56 (s, 3H), 1.38 (d, *J* = 6.9 Hz, 6H), 1.29 (d, *J* = 6.6 Hz, 6H) ppm.

^{13}C{^{1}H} NMR (101 MHz, CDCl$_3$): δ 182.5, 162.8, 149.2, 147.0, 144.4, 143.8, 140.6, 136.5, 134.2, 133.5, 125.4, 122.1, 41.8, 38.3, 28.7, 24.6, 22.7, 13.0 ppm.

HRMS: (DART/ORBITRAP) calculated for $C_{20}H_{26}NO_2^+$ ([M+H]$^+$): 312.1958, found: 312.1959.

FTIR (ATR, cm^{-1}): 3228 (m), 3060 (w), 2959 (m), 2929 (m), 2868 (m), 1628 (s), 1532 (m).

mp: 108–110 °C

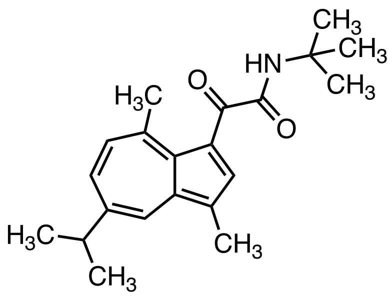

7d

***N*-(*tert*-Butyl)-2-(5-isopropyl-3,8-dimethylazulen-1-yl)-2-oxoacetamide (7d).** Prepared according to **General Procedure A** using *tert*-butylamine (0.21 mL, 2.0 mmol, 4.0 equiv) as the nucleophile. The crude product was purified *via* column chromatography (silica, 10% EtOAc in hexanes) to afford **7d** as a brown oil that solidified upon standing (123.3 mg, 0.379 mmol, 72% yield).

***R*$_f$:** 0.61 in 30% EtOAc in hexanes.

^{1}H NMR (400 MHz, $CDCl_3$): δ 8.36 (s, 1H), 8.25 (d, *J* = 2.1 Hz, 1H), 7.61 (dd, *J* = 11.0, 2.1 Hz, 1H), 7.41 (d, *J* = 11.0 Hz, 1H), 7.25 (s, 1H), 3.15 (hept, *J* = 6.9 Hz, 1H), 2.87 (s, 3H), 2.56 (s, 3H), 1.49 (s, 9H), 1.38 (d, *J* = 7.0 Hz, 6H) ppm.

^{13}C{^{1}H} NMR (101 MHz, $CDCl_3$): δ 183.5, 162.9, 149.0, 146.8, 144.3, 143.5, 140.6, 136.4, 134.2, 133.3, 125.3, 122.0, 51.4, 38.3, 28.8, 28.6, 24.6, 12.9 ppm.

HRMS: (DART/ORBITRAP) calculated for $C_{21}H_{28}NO_2^+$ ([M+H]$^+$): 326.2115, found: 326.2115.

FTIR (ATR, cm^{-1}): 3362 (m), 3317 (m), 2961 (m), 2927 (w), 2867 (w), 1670 (s), 1618 (s).

mp: 61–65 °C

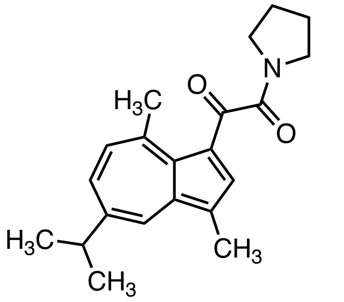

7e

1-(5-Isopropyl-3,8-dimethylazulen-1-yl)-2-(pyrrolidin-1-yl)ethane-1,2-dione (7e). Prepared according to **General Procedure A** using pyrrolidine (0.18 mL, 2.2 mmol, 4.4 equiv) as the

nucleophile. The crude product was purified *via* column chromatography (silica, 70% EtOAc in hexanes) to afford **7e** as a brown solid (149.6 mg, 0.463 mmol, 92% yield).

***R*<u>f</u>:** 0.36 in 50% EtOAc in hexanes.

^{1}H NMR (400 MHz, $CDCl_3$): δ 8.26 (d, *J* = 2.2 Hz, 1H), 7.93 (s, 1H), 7.63 (dd, *J* = 11.0, 2.2 Hz, 1H), 7.47 (d, *J* = 11.0 Hz, 1H), 3.69 (t, *J* = 6.7 Hz, 2H), 3.52 (t, *J* = 6.4 Hz, 2H), 3.16 (hept, *J* = 6.9 Hz, 1H), 3.09 (s, 3H), 2.55 (d, *J* = 0.7 Hz, 3H), 1.95 (dtd, *J* = 9.6, 7.0, 4.9 Hz, 4H), 1.38 (d, *J* = 6.9 Hz, 6H) ppm.

^{13}C{^{1}H} NMR (101 MHz, $CDCl_3$): 186.6, 167.7, 150.5, 147.1, 144.0, 143.4, 139.5, 137.0, 134.7, 134.0, 125.5, 121.6, 47.1, 45.5, 38.3, 29.0, 26.2, 24.6, 24.3, 13.0 ppm.

HRMS: (DART/ORBITRAP) calculated for $C_{21}H_{26}NO_2^+$ ($[M+H]^+$): 324.1958, found: 324.1958.

FTIR (ATR, cm^{-1}): 2959 (m), 2927 (m), 2872 (w), 1642 (s), 1622 (s).

mp: 104–109 °C

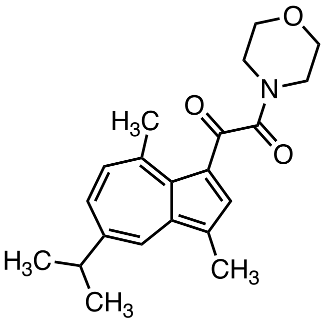

7f

1-(5-Isopropyl-3,8-dimethylazulen-1-yl)-2-morpholinoethane-1,2-dione (7f). Prepared according to **General Procedure A** using morpholine (0.19 mL, 2.2 mmol, 4.4 equiv) as the nucleophile. The crude product was purified *via* column chromatography (silica, 70% EtOAc in hexanes) to afford **7f** as a brown solid (142.0 mg, 0.418 mmol, 83% yield).

***R*<u>f</u>:** 0.37 in 70% EtOAc in hexanes.

^{1}H NMR (400 MHz, $CDCl_3$): δ 8.27 (d, *J* = 2.1 Hz, 1H), 7.87 (s, 1H), 7.66 (dd, *J* = 11.1, 2.1 Hz, 1H), 7.51 (d, *J* = 11.0 Hz, 1H), 3.83 (s, 4H), 3.71 – 3.64 (m, 2H), 3.48 (t, *J* = 4.7 Hz, 2H), 3.22 – 3.13 (m, 1H), 3.12 (s, 3H), 2.55 (s, 3H), 1.38 (d, *J* = 5.8 Hz, 6H) ppm.

^{13}C{^{1}H} NMR (101 MHz, CDCl$_3$): δ 185.2, 168.2, 151.0, 147.7, 144.3, 143.1, 139.6, 137.3, 135.0, 134.5, 125.7, 121.4, 67.1, 67.0, 46.8, 41.9, 38.3, 29.2, 24.6, 13.1 ppm.

HRMS: (DART/ORBITRAP) calculated for $C_{21}H_{26}NO_3^+$ ([M+H]$^+$): 340.1907, found: 340.1908.

FTIR (ATR, cm^{-1}): 2957 (w), 2925 (w), 2863 (w), 1631 (s), 1536 (w).

mp: 98–101 °C

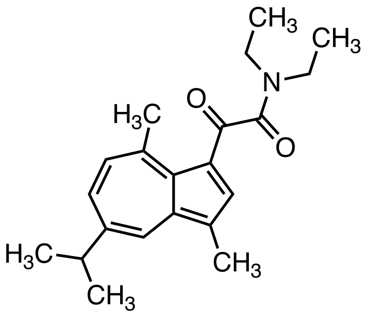

7g

***N*,*N*-Diethyl-2-(5-isopropyl-3,8-dimethylazulen-1-yl)-2-oxoacetamide (7g).** Prepared according to **General Procedure A** using *N*,*N*-diethylamine (0.21 mL, 2.0 mmol, 4.0 equiv) as the nucleophile. The crude product was purified *via* column chromatography (silica, 30% EtOAc in hexanes) to afford **7g** as a brown oil that solidified upon standing (114.1 mg, 0.351 mmol, 70% yield).

***R*$_f$:** 0.29 in 30% EtOAc in hexanes.

^{1}H NMR (400 MHz, CDCl$_3$): δ 8.26 (d, *J* = 2.1 Hz, 1H), 7.85 (s, 1H), 7.63 (dd, *J* = 11.0, 2.2 Hz, 1H), 7.47 (d, *J* = 11.1 Hz, 1H), 3.58 (q, *J* = 7.2 Hz, 2H), 3.35 (q, *J* = 7.1 Hz, 2H), 3.20 – 3.10 (m, 4H), 2.54 (s, 3H), 1.37 (d, *J* = 6.9 Hz, 6H), 1.31 (t, *J* = 7.2 Hz, 3H), 1.17 (t, *J* = 7.1 Hz, 3H) ppm.

^{13}C{^{1}H} NMR (101 MHz, CDCl$_3$): δ 186.6, 169.3, 150.7, 147.1, 143.9, 143.2, 139.2, 137.0, 134.7, 134.0, 125.4, 121.7, 42.5, 38.9, 38.2, 29.0, 24.6, 14.3, 13.0, 13.0 ppm.

HRMS: (DART/ORBITRAP) calculated for $C_{21}H_{28}NO_2^+$ ([M+H]$^+$): 326.2115, found: 326.2117.

FTIR (ATR, cm^{-1}): 2973 (w), 2931 (w), 2870 (w), 1638 (s), 1617 (s).

mp: 82–85 °C

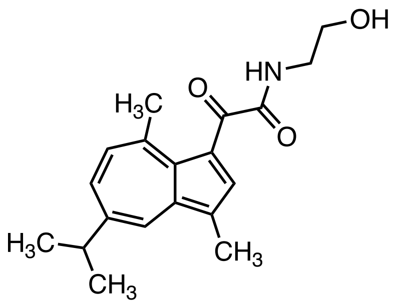

7h

***N*-(2-Hydroxyethyl)-2-(5-isopropyl-3,8-dimethylazulen-1-yl)-2-oxoacetamide (7h).** Prepared according to **General Procedure A** using ethanolamine (0.50 mL, 8.3 mmol, 1.6 equiv) as the nucleophile. The crude product was purified *via* column chromatography (silica, 70% EtOAc in hexanes) to afford **7h** as a brown oil (118.8 mg, 0.379 mmol, 75% yield). Note that a higher amount of amine nucleophile was used in this case to minimize formation of a side product resulting from functionalizing the alcohol group with a second equivalent of the guaiazulene compound.

R_f: 0.23 in 70% EtOAc in hexanes; 0.40 in 100% EtOAc.

^{1}H NMR (400 MHz, $CDCl_3$): δ 8.35 (s, 1H), 8.26 (d, *J* = 2.1 Hz, 1H), 7.78 (t, *J* = 6.1 Hz, 1H), 7.63 (dd, *J* = 11.1, 2.2 Hz, 1H), 7.43 (d, *J* = 11.0 Hz, 1H), 3.84 (t, *J* = 5.0 Hz, 2H), 3.59 (td, *J* = 5.8, 4.5 Hz, 2H), 3.15 (hept, *J* = 6.9 Hz, 1H), 2.93 (s, 1H), 2.87 (s, 3H), 2.55 (s, 3H), 1.38 (d, *J* = 6.9 Hz, 6H) ppm.

^{13}C{^{1}H} NMR (101 MHz, $CDCl_3$): δ 181.6, 164.9, 149.4, 147.4, 144.2, 144.1, 140.8, 136.6, 134.3, 133.8, 125.6, 121.8, 62.3, 42.7, 38.3, 28.7, 24.6, 13.0 ppm.

HRMS: (DART/ORBITRAP) calculated for $C_{19}H_{24}NO_3^+$ ($[M+H]^+$): 314.1751, found: 314.1751.

FTIR (ATR, cm^{-1}): 3329 (w, broad), 2959 (w), 2927 (w), 2868 (w), 1619 (m), 1513 (m).

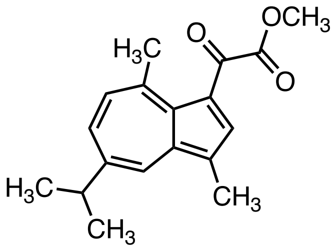

7i

Methyl 2-(5-isopropyl-3,8-dimethylazulen-1-yl)-2-oxoacetate (7i). Prepared according to **General Procedure A** using methanol (0.5 mL) as the nucleophile. The crude product was purified *via* column chromatography (silica, 10 → 30% EtOAc in hexanes) to afford **7i** as a brown solid (124.5 mg, 0.438 mmol, 87% yield).

R_f: 0.48 in 30% EtOAc in hexanes.

^{1}H NMR (400 MHz, CDCl$_3$δ 8.28 (d, *J* = 2.1 Hz, 1H), 8.00 (s, 1H), 7.66 (dd, *J* = 11.0, 2.1 Hz, 1H), 7.49 (d, *J* = 11.0 Hz, 1H), 3.98 (s, 3H), 3.16 (hept, *J* = 6.9 Hz, 1H), 3.00 (s, 3H), 2.56 (d, *J* = 0.8 Hz, 3H), 1.39 (d, *J* = 6.9 Hz, 6H) ppm.

^{13}C{^{1}H} NMR (101 MHz, CDCl$_3$): δ 179.9, 166.1, 150.5, 147.7, 144.2, 143.0, 140.0, 137.2, 134.8, 134.3, 125.6, 121.2, 52.7, 38.3, 29.0, 24.6, 13.0 ppm.

HRMS: (DART/ORBITRAP) calculated for $C_{18}H_{21}O_3^+$ ([M+H]$^+$): 285.1485, found: 285.1485.

FTIR (ATR, cm^{-1}): 2966 (w), 2928 (w), 2870.(w), 1720 (s), 1632 (s).

mp: 86–90 °C

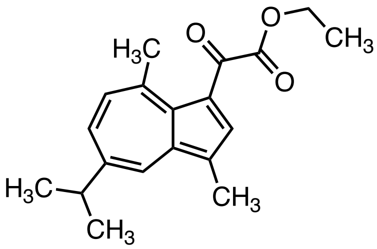

7j

Ethyl 2-(5-isopropyl-3,8-dimethylazulen-1-yl)-2-oxoacetate (7j). Prepared according to **General Procedure A** using ethanol (0.5 mL) as the nucleophile. The crude product was purified

via column chromatography (silica, 5 → 30% EtOAc in hexanes) to afford **7j** as a brown solid (135.7 mg, 0.455 mmol, 90% yield).

R_f: 0.12 in 5% EtOAc in hexanes.

^{1}H NMR (400 MHz, $CDCl_3$): δ 8.28 (d, *J* = 2.1 Hz, 1H), 7.99 (s, 1H), 7.65 (dd, *J* = 11.0, 2.2 Hz, 1H), 7.48 (d, *J* = 11.1 Hz, 1H), 4.45 (q, *J* = 7.1 Hz, 2H), 3.16 (hept, *J* = 6.9 Hz, 1H), 3.01 (s, 3H), 2.56 (d, *J* = 0.8 Hz, 3H), 1.45 (t, *J* = 7.1 Hz, 3H), 1.38 (d, *J* = 6.9 Hz, 6H) ppm.

^{13}C{^{1}H} NMR (101 MHz, $CDCl_3$): δ 180.6, 165.8, 150.5, 147.5, 144.1, 143.0, 139.8, 137.1, 134.8, 134.2, 125.5, 121.2, 62.0, 38.3, 29.0, 24.6, 14.4, 13.0 ppm.

HRMS: (DART/ORBITRAP) calculated for $C_{19}H_{23}O_3^+$ ($[M+H]^+$): 299.1642, found: 299.1642.

FTIR (ATR, cm^{-1}): 2960 (m), 2869 (w), 1721 (s), 1622 (s).

mp: 78–80 °C

7j was also prepared on 1 g scale according to the following procedure. A 100-mL round-bottomed flask was charged with a stir bar, guaiazulene **2** (1.00 g, 5.04 mmol, 1.00 equiv), and THF (10 mL). The flask was cooled to 0 ºC before oxalyl chloride (0.450 mL, 5.32 mmol, 1.05 equiv). The reaction was stirred at 0 ºC for 1 h, then EtOH (5 mL) was added. The reaction was stirred at 0 ºC for an additional 30 minutes, then the reaction was quenched with water (50 mL), extracted with EtOAc (3 x 50 mL), dried over $MgSO_4$, filtered, and rotovapped to give a dark brown oil. The crude material was purified *via* column chromatography (silica, 5 → 20% EtOAc in hexanes) to give **7j** as a dark brown solid (1.46 g, 4.89 mmol, 97% yield).

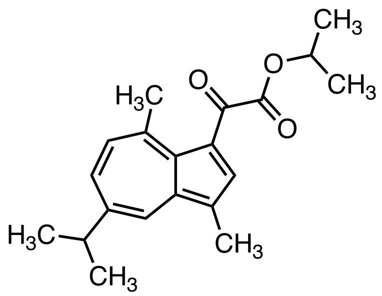

7k

Isopropyl 2-(5-isopropyl-3,8-dimethylazulen-1-yl)-2-oxoacetate (7k). Prepared according to **General Procedure A** using isopropanol (0.5 mL) as the nucleophile. The crude product was

purified *via* column chromatography (silica, 5 → 30% EtOAc in hexanes) to afford **7k** as a brown solid (145.9 mg, 0.467 mmol, 93% yield).

***R*<u>f</u>:** 0.13 in 5% EtOAc in hexanes.

^{1}H NMR (400 MHz, CDCl$_3$): δ 8.27 (d, *J* = 2.1 Hz, 1H), 7.96 (s, 1H), 7.65 (dd, *J* = 11.1, 2.2 Hz, 1H), 7.47 (d, *J* = 11.0 Hz, 1H), 5.32 (hept, *J* = 6.3 Hz, 1H), 3.16 (hept, *J* = 6.9 Hz, 1H), 3.02 (s, 3H), 2.56 (s, 3H), 1.43 (d, *J* = 6.3 Hz, 6H), 1.38 (d, *J* = 6.9 Hz, 6H) ppm.

^{13}C{^{1}H} NMR (101 MHz, CDCl$_3$): δ 181.1, 165.6, 150.5, 147.4, 144.0, 142.9, 139.7, 137.1, 134.8, 134.2, 125.5, 121.2, 69.9, 38.3, 29.0, 24.60, 22.0, 13.1 ppm.

HRMS: (DART/ORBITRAP) calculated for $C_{20}H_{25}O_3^+$ ([M+H]$^+$): 313.1798, found: 313.1796.

FTIR (ATR, cm^{-1}): 2978 (w), 2955 (m), 2865 (w), 1716 (s), 1637 (s).

mp: 68–70 °C

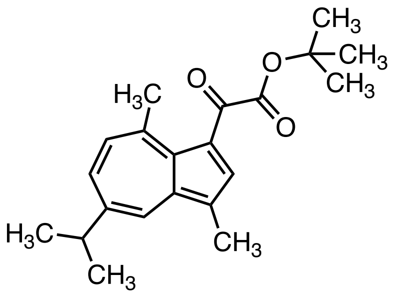

7l

***tert*-Butyl 2-(5-isopropyl-3,8-dimethylazulen-1-yl)-2-oxoacetate (7l).** Preparation attempted according to **General Procedure A** using *tert*-butanol (0.5 mL) as the nucleophile. The crude product was purified via column chromatography (silica, 5 → 30 → 100% EtOAc in hexanes) but no **7l** was isolated or apparent in a crude ^{1}H NMR spectrum. Instead, the major product was **7m** (85.0 mg, 0.314 mmol, 62% yield of **7m**).

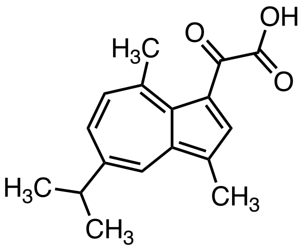

7m

2-(5-Isopropyl-3,8-dimethylazulen-1-yl)-2-oxoacetic acid (7m). Prepared according to **General Procedure A** using water (0.5 mL) as the nucleophile. The crude product was purified via column chromatography (silica, 100% EtOAc in hexanes) to afford **7m** as a red/brown solid (89.3 mg, 0.330 mmol, 65% yield).

***R*$_f$:** 0.05 in 100% EtOAc in hexanes.

^{1}H NMR (400 MHz, CDCl$_3$): δ 8.64 (s, 1H), 8.31 (d, *J* = 2.1 Hz, 1H), 7.73 (dd, *J* = 11.1, 2.2 Hz, 1H), 7.58 (d, *J* = 11.0 Hz, 1H), 3.20 (hept, *J* = 6.9 Hz, 1H), 2.93 (s, 3H), 2.56 (s, 3H), 1.41 (d, *J* = 6.9 Hz, 6H) ppm.

^{13}C{^{1}H} NMR (101 MHz, CDCl$_3$): δ 175.0, 162.1, 150.6, 149.8, 146.1, 145.2, 142.7, 137.4, 135.7, 134.7, 126.9, 119.8, 38.5, 28.9, 24.6, 13.0 ppm.

HRMS: (DART/ORBITRAP) calculated for $C_{17}H_{19}O_3^+$ ([M+H]$^+$): 271.1329, found: 271.1330.

FTIR (ATR, cm^{-1}): 3181 (w, broad), 2971 (w), 2959 (w), 2924 (w), 2862 (w), 1730 (s), 1617 (s).

mp: 115–117 °C

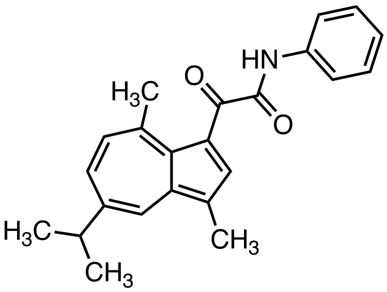

7n

2-(5-Isopropyl-3,8-dimethylazulen-1-yl)-2-oxo-*N*-phenylacetamide (7n). Prepared according to **General Procedure A** using aniline (0.10 mL, 1.1 mmol, 2.2 equiv) as the nucleophile as well

as triethylamine (0.15 mL, 1.1 mmol, 2.2 equiv) as an added base. The crude product was purified via column chromatography (silica, 10% EtOAc in hexanes) to afford **7n** as a brown solid (139.3 mg, 0.422 mmol, 80% yield).

***R*$_f$:** 0.48 in 30% EtOAc in hexanes; 0.88 in 70% EtOAc in hexanes.

^{1}H NMR (400 MHz, $CDCl_3$): δ 9.31 (broad s, 1H), 8.49 (s, 1H), 8.30 (d, *J* = 2.1 Hz, 1H), 7.81 – 7.73 (m, 2H), 7.67 (dd, *J* = 11.0, 2.1 Hz, 1H), 7.49 (d, *J* = 11.0 Hz, 1H), 7.45 – 7.35 (m, 2H), 7.22 – 7.13 (m, 1H), 3.18 (hept, *J* = 6.9 Hz, 1H), 2.92 (s, 3H), 2.58 (s, 3H), 1.40 (d, *J* = 6.9 Hz, 6H) ppm.

^{13}C{^{1}H} NMR (101 MHz, $CDCl_3$): δ 181.2, 161.1, 149.4, 147.7, 144.5, 144.2, 141.2, 137.5, 136.7, 134.4, 134.0, 129.3, 125.8, 124.9, 121.6, 119.8, 38.4, 28.8, 24.7, 13.0 ppm.

HRMS: (DART/ORBITRAP) calculated for $C_{23}H_{24}NO_2^+$ ([M+H]$^+$): 346.1802, found: 346.1802.

FTIR (ATR, cm^{-1}): 3334 (w), 3061 (w), 2963 (w), 2928 (w), 2869 (w), 1682 (s), 1607 (s), 1589 (s).

mp: 154–156 °C

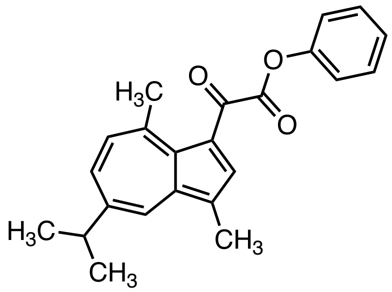

7o

Phenyl 2-(5-isopropyl-3,8-dimethylazulen-1-yl)-2-oxoacetate (7o). Prepared according to **General Procedure A** using phenol (102 mg, 1.1 mmol, 2.2 equiv) as the nucleophile as well as triethylamine (0.15 mL, 1.1 mmol, 2.2 equiv) as an added base. The crude product was purified via column chromatography (silica, 10% EtOAc in hexanes) to afford **7o** as a brown oil that solidified upon standing (114.2 mg, 0.330 mmol, 65% yield).

***R*$_f$:** 0.13 in 5% EtOAc in hexanes.

^{1}H NMR (400 MHz, CDCl$_3$): δ 8.31 (d, *J* = 2.1 Hz, 1H), 8.12 (s, 1H), 7.70 (dd, *J* = 11.0, 2.2 Hz, 1H), 7.54 (d, *J* = 11.1 Hz, 1H), 7.50 – 7.40 (m, 2H), 7.37 – 7.27 (m, 3H), 3.19 (hept, *J* = 6.9 Hz, 1H), 3.08 (s, 3H), 2.58 (s, 3H), 1.40 (d, *J* = 6.9 Hz, 6H) ppm.

^{13}C{^{1}H} NMR (101 MHz, CDCl$_3$): δ 179.0, 163.9, 150.8, 150.8, 148.1, 144.5, 143.0, 140.3, 137.3, 135.0, 134.7, 129.7, 126.4, 125.9, 121.6, 120.8, 38.3, 29.1, 24.6, 13.1 ppm.

HRMS: (DART/ORBITRAP) calculated for $C_{23}H_{23}O_3^+$ ([M+H]$^+$): 347.1642, found: 347.1641.

FTIR (ATR, cm^{-1}): 2958 (w), 2923 (w), 2853 (w), 1738 (m), 1636 (m).

mp: 114–115 °C

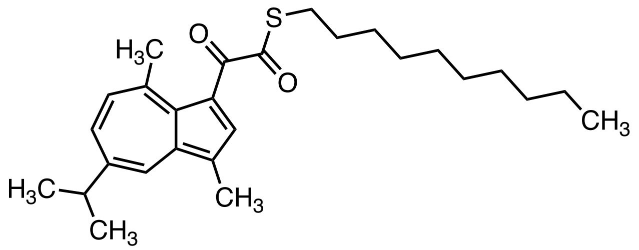

7p

***S*-Decyl 2-(5-isopropyl-3,8-dimethylazulen-1-yl)-2-oxoethanethioate (7p).** Prepared according to **General Procedure A** using 1-decanethiol (0.22 mL, 1.1 mmol, 2.1 equiv) as the nucleophile as well as triethylamine (0.15 mL, 1.1 mmol, 2.2 equiv) as an added base. The crude product was purified via column chromatography (silica, 0 → 2 → 5% EtOAc in hexanes) to afford **7p** as a brown oil (181.4 mg, 0.425 mmol, 84% yield).

R_f: 0.20 in 5% EtOAc in hexanes.

^{1}H NMR (400 MHz, CDCl$_3$): δ 8.27 (d, *J* = 2.1 Hz, 1H), 8.10 (s, 1H), 7.66 (dd, *J* = 11.0, 2.1 Hz, 1H), 7.48 (d, *J* = 11.0 Hz, 1H), 3.16 (hept, *J* = 6.9 Hz, 1H), 3.03 (t, *J* = 7.4 Hz, 2H), 2.94 (s, 3H), 2.56 (s, 3H), 1.70 (p, *J* = 7.4 Hz, 2H), 1.51 – 1.40 (m, 2H), 1.38 (d, *J* = 6.9 Hz, 6H), 1.32 – 1.24 (m, 12H), 0.92 – 0.84 (m, 3H) ppm.

^{13}C{^{1}H} NMR (101 MHz, CDCl$_3$): δ 195.5, 180.5, 150.2, 147.8, 144.4, 143.3, 140.9, 137.0, 134.6, 134.3, 125.9, 119.6, 38.3, 32.0, 29.69, 29.65, 29.5, 29.4, 29.31, 29.26, 29.1, 28.9, 24.6, 22.8, 14.3, 13.0 ppm.

HRMS: (DART/ORBITRAP) calculated for $C_{27}H_{39}O_2S^+$ ([M+H]$^+$): 427.2665, found: 427.2665.

FTIR (ATR, cm^{-1}): 2957 (w), 2923 (m), 2852 (m), 1672 (m), 1628 (m).

mp: 114–115 °C

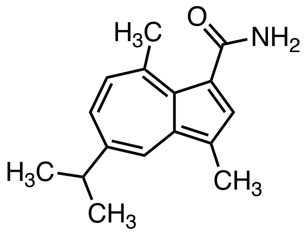

8a

5-Isopropyl-3,8-dimethylazulene-1-carboxamide (8a). Prepared according to **General Procedure B** using a 25-mL round-bottomed flask instead of a 1-dram vial with NH_3 (4.0 mL of a 0.50 M solution in dioxane, 2.0 mmol, 4.0 equiv) as the nucleophile. The crude product was purified via column chromatography (silica, 30 → 70 → 100% EtOAc in hexanes) to afford **8a** as a brown solid that appeared purple in solution (12.8 mg, 0.053 mmol, 11% yield).

***R*$_f$:** 0.12 in 30% EtOAc in hexanes.

^{1}H NMR (400 MHz, $CDCl_3$): δ 8.23 (d, J = 2.1 Hz, 1H), 7.72 (s, 1H), 7.50 (dd, J = 10.8, 2.1 Hz, 1H), 7.17 (d, J = 10.9 Hz, 1H), 5.85 (broad s, 2H), 3.10 (hept, J = 7.0 Hz, 1H), 2.97 (s, 3H), 2.60 (s, 3H), 1.36 (d, J = 6.8 Hz, 6H) ppm.

^{13}C{^{1}H} NMR (101 MHz, $CDCl_3$): δ 171.8, 147.1, 142.3, 138.6, 137.4, 136.1, 134.7, 133.9, 129.3, 123.9, 121.2, 38.1, 26.8, 24.8, 12.9 ppm.

HRMS: (DART/ORBITRAP) calculated for $C_{16}H_{20}NO^+$ ($[M+H]^+$): 242.1539, found: 242.1539.

FTIR (ATR, cm^{-1}): 3386 (w), 3174 (w), 2962 (w), 2924 (w), 2855 (w), 1706 (w), 1637 (s).

mp: 172–174 °C

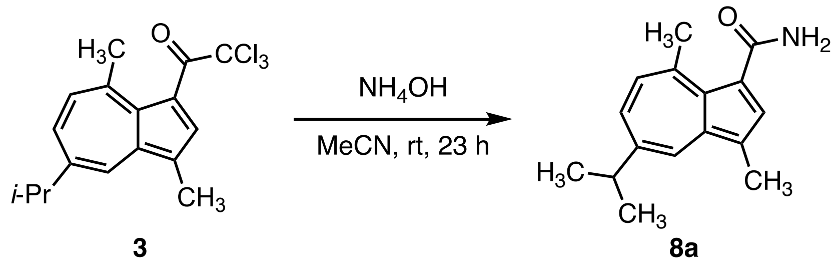

Compound **8a** was also prepared more efficiently by another procedure. A 1-dram vial was charged with a stir bar, **3** (50 mg, 0.15 mmol, 1.0 equiv), MeCN (1 mL), and ~30% aq. NH_4OH (1 mL). The reaction was stirred at rt for 23 h, then diluted with EtOAc (2 mL). The organic layer was pipetted off, filtered through $MgSO_4$, and concentrated on the rotary evaporator to give a brown solid. The crude material was purified via a pipette column (silica, 5 → 30 → 100% EtOAc in hexanes) to afford **8a** as a purple solid (34.9 mg, 0.15 mmol, 99% yield).

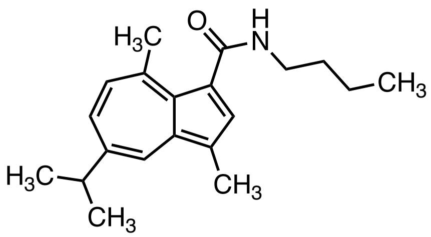

8b

***N*-Butyl-5-isopropyl-3,8-dimethylazulene-1-carboxamide (8b).** Prepared according to **General Procedure B** using *n*-butylamine (0.20 mL, 2.0 mmol, 4.0 equiv) as the nucleophile. The crude product was purified via column chromatography (silica, 30 → 70% EtOAc in hexanes) to afford **8b** as a purple solid (114.4 mg, 0.385 mmol, 76% yield).

R_f: 0.41 in 30% EtOAc in hexanes; 0.53 in 70% EtOAc in hexanes.

^{1}H NMR (400 MHz, $CDCl_3$): δ 8.21 (d, *J* = 2.1 Hz, 1H), 7.63 (s, 1H), 7.46 (dd, *J* = 10.8, 2.1 Hz, 1H), 7.11 (d, *J* = 10.8 Hz, 1H), 5.82 (t, *J* = 6.0 Hz, 1H), 3.49 (q, *J* = 6.6 Hz, 2H), 3.10 (h, *J* = 6.9 Hz, 1H), 2.91 (s, 3H), 2.60 (s, 3H), 1.68 – 1.55 (m, 2H), 1.50 – 1.40 (m, 2H), 1.36 (d, *J* = 6.5 Hz, 6H), 0.97 (t, *J* = 7.3 Hz, 3H) ppm.

^{13}C{^{1}H} NMR (101 MHz, $CDCl_3$): δ 170.4, 146.6, 141.7, 137.9, 137.0, 135.9, 134.6, 133.2, 128.5, 124.0, 123.1, 40.1, 38.1, 31.9, 26.3, 24.8, 20.4, 14.0, 12.9 ppm.

HRMS: (DART/ORBITRAP) calculated for $C_{20}H_{28}NO^+$ ($[M+H]^+$): 298.2165, found: 298.2165.

FTIR (ATR, cm^{-1}): 3238 (w), 2956 (m), 2921 (m), 2857 (m), 1615 (s), 1532 (s).

mp: 131–134 °C

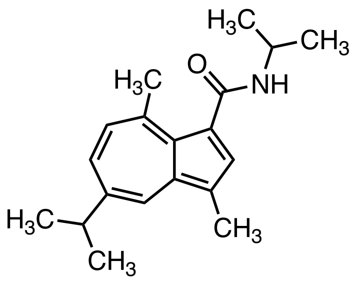

8c

***N*,5-Diisopropyl-3,8-dimethylazulene-1-carboxamide (8c).** Prepared according to **General Procedure B** using isopropylamine (0.17 mL, 2.0 mmol, 4.0 equiv) as the nucleophile. The crude product was purified via column chromatography (silica, 30 → 100% EtOAc in hexanes) to afford **8c** as a purple solid (105.8 mg, 0.373 mmol, 74% yield).

R_f: 0.22 in 30% EtOAc in hexanes; 0.56 in 70% EtOAc in hexanes.

^{1}H NMR (400 MHz, CDCl$_3$): δ 8.21 (d, *J* = 2.1 Hz, 1H), 7.63 (s, 1H), 7.45 (dd, *J* = 10.8, 2.1 Hz, 1H), 7.10 (d, *J* = 10.8 Hz, 1H), 5.65 (d, *J* = 8.1 Hz, 1H), 4.42 – 4.25 (m, 1H), 3.14 – 3.03 (m, 1H), 2.91 (s, 3H), 2.60 (s, 3H), 1.36 (d, *J* = 6.9 Hz, 6H), 1.28 (d, *J* = 6.5 Hz, 6H) ppm.

^{13}C{^{1}H} NMR (101 MHz, CDCl$_3$): δ 169.7, 146.5, 141.6, 137.9, 137.1, 135.8, 134.6, 133.1, 128.4, 124.0, 123.3, 42.0, 38.1, 26.3, 24.8, 23.0, 12.9 ppm.

HRMS: (DART/ORBITRAP) calculated for $C_{19}H_{26}NO^+$ ([M+H]$^+$): 284.2009, found: 284.2008.

FTIR (ATR, cm^{-1}): 3294 (m), 2962 (m), 2927 (w), 2869 (w), 1620 (s), 1526 (s), 1516 (s).

mp: 138–142 °C

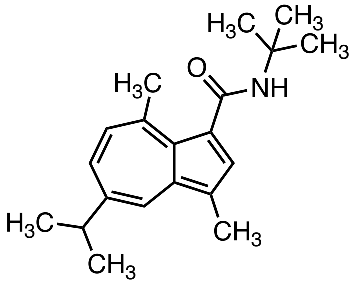

8d

***N*-(*tert*-Butyl)-5-isopropyl-3,8-dimethylazulene-1-carboxamide (8d).** Prepared according to **General Procedure B** using *tert*-butylamine (0.21 mL, 2.0 mmol, 4.0 equiv) as the nucleophile. The crude product was purified via column chromatography (silica, 5 → 10% EtOAc in hexanes) to afford **8d** as a purple solid (100.9 mg, 0.339 mmol, 67% yield).

R_f: 0.48 in 30% EtOAc in hexanes.

^{1}H NMR (400 MHz, CDCl$_3$): δ 8.21 (d, *J* = 2.1 Hz, 1H), 7.63 (s, 1H), 7.45 (dd, *J* = 10.8, 2.1 Hz, 1H), 7.09 (d, *J* = 10.8 Hz, 1H), 5.68 (s, 1H), 3.09 (hept, *J* = 6.9 Hz, 1H), 2.94 (s, 3H), 2.61 (d, *J* = 0.7 Hz, 3H), 1.50 (s, 9H), 1.36 (d, *J* = 6.9 Hz, 6H) ppm.

^{13}C{^{1}H} NMR (101 MHz, CDCl$_3$): δ 170.0, 146.5, 141.3, 137.5, 137.0, 135.7, 134.5, 132.6, 128.1, 124.6, 123.9, 51.7, 38.1, 289.0, 26.3, 24.8, 12.9 ppm.

HRMS: (DART/ORBITRAP) calculated for $C_{20}H_{28}NO^+$ ([M+H]$^+$): 298.2165, found: 298.2164.

FTIR (ATR, cm^{-1}): 3267 (m), 2963 (m), 2923 (w), 2865 (w), 1620 (s), 1537 (s).

mp: 138–142 °C

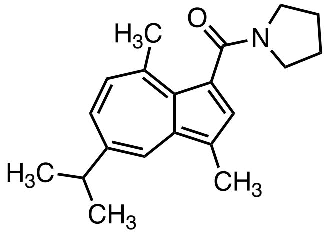

8e

(5-Isopropyl-3,8-dimethylazulen-1-yl)(pyrrolidin-1-yl)methanone (8e). Prepared according to **General Procedure B** using pyrrolidine (0.18 mL, 2.2 mmol, 4.3 equiv) as the nucleophile. The

crude product was purified via column chromatography (silica, 70% EtOAc in hexanes) to afford **8e** as a blue oil that solidified upon standing (92.5 mg, 0.313 mmol, 62% yield).

***R*f:** 0.25 in 50% EtOAc in hexanes.

^{1}H NMR (400 MHz, $CDCl_3$): δ 8.18 (d, *J* = 2.1 Hz, 1H), 7.54 (s, 1H), 7.41 (dd, *J* = 10.7, 2.1 Hz, 1H), 7.03 (d, *J* = 10.7 Hz, 1H), 3.71 (t, *J* = 7.0 Hz, 2H), 3.15 – 3.00 (m, 3H), 2.78 (s, 3H), 2.64 – 2.59 (m, 3H), 1.98 (p, *J* = 6.9 Hz, 2H), 1.83 (p, *J* = 6.8 Hz, 2H), 1.36 (d, *J* = 6.9 Hz, 6H) ppm.

^{13}C{^{1}H} NMR (101 MHz, $CDCl_3$): δ 170.8, 145.3, 141.0, 137.4, 135.7, 135.5, 134.4, 131.9, 127.4, 124.8, 123.2, 49.3, 45.8, 38.11, 25.9, 25.1, 25.0, 24.8, 13.0 ppm.

HRMS: (DART/ORBITRAP) calculated for $C_{20}H_{26}NO^+$ ($[M+H]^+$): 296.2009, found: 296.2009.

FTIR (ATR, cm^{-1}): 2957 (w), 2923 (w), 2863 (w), 1626 (s).

mp: 124–126 °C

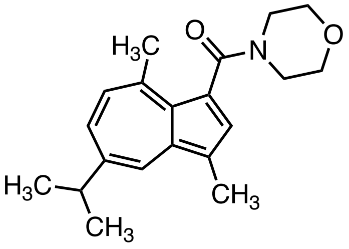

8f

(5-Isopropyl-3,8-dimethylazulen-1-yl)(morpholino)methanone (8f). Prepared according to **General Procedure B** using morpholine (0.19 mL, 2.2 mmol, 4.3 equiv) as the nucleophile. The crude product was purified via column chromatography (silica, 70% EtOAc in hexanes) to afford **8f** as a blue oil (130.2 mg, 0.418 mmol, 83% yield).

***R*f:** 0.22 in 50% EtOAc in hexanes.

^{1}H NMR (400 MHz, $CDCl_3$): δ 8.20 (d, *J* = 2.0 Hz, 1H), 7.48 (s, 1H), 7.44 (dd, *J* = 10.7, 2.1 Hz, 1H), 7.06 (d, *J* = 10.7 Hz, 1H), 4.06 – 3.93 (m, 1H), 3.85 – 3.76 (m, 3H), 3.56 (t, *J* = 4.9 Hz, 2H),

3.31 – 3.22 (m, 2H), 3.08 (hept, J = 6.9 Hz, 1H), 2.81 (s, 3H), 2.62 (s, 3H), 1.36 (d, J = 6.9 Hz, 6H) ppm.

$^{13}C\{^1H\}$ NMR (101 MHz, $CDCl_3$): δ 171.0, 145.6, 141.5, 137.7, 135.8, 135.7, 134.6, 132.7, 127.7, 124.9, 120.4, 67.0, 66.8, 48.1, 42.4, 38.1, 25.5, 24.8, 13.0 ppm.

HRMS: (DART/ORBITRAP) calculated for $C_{20}H_{26}NO_2^+$ ($[M+H]^+$): 312.1958, found: 312.1958.

FTIR (ATR, cm^{-1}): 3420 (w, broad), 2958 (m), 2923 (m), 2854 (m), 1622 (s).

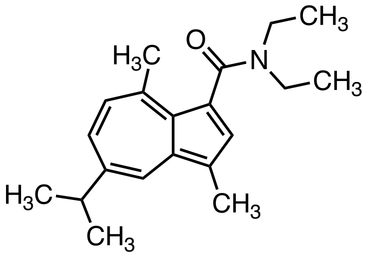

8g

***N*,*N*-Diethyl-5-isopropyl-3,8-dimethylazulene-1-carboxamide (8g).** Prepared according to **General Procedure B** using *N*,*N*-diethylamine (0.21 mL, 2.0 mmol, 4.0 equiv) as the nucleophile. The crude product was purified via column chromatography (silica, 30% EtOAc in hexanes) to afford **8g** as a blue oil (102.7 mg, 0.345 mmol, 68% yield).

R_f: 0.20 in 30% EtOAc in hexanes.

^1H NMR (400 MHz, $CDCl_3$): δ 8.18 (d, J = 2.1 Hz, 1H), 7.50 (s, 1H), 7.40 (dd, J = 10.7, 2.1 Hz, 1H), 7.01 (d, J = 10.7 Hz, 1H), 3.90 (s, 1H), 3.40 (s, 1H), 3.31 – 3.12 (m, 2H), 3.07 (hept, J = 6.9 Hz, 1H), 2.79 (s, 3H), 2.63 (s, 3H), 1.36 (d, J = 6.8 Hz, 6H), 1.29 (t, J = 7.1 Hz, 3H), 1.02 (t, J = 7.1 Hz, 3H) ppm.

$^{13}C\{^1H\}$ NMR (101 MHz, $CDCl_3$): δ 171.8, 145.6, 140.8, 137.3, 135.6, 135.5, 134.3, 132.1, 127.2, 124.7, 122.4, 43.3, 39.0, 38.1, 25.3, 24.7, 13.9, 12.9, 12.4 ppm.

HRMS: (DART/ORBITRAP) calculated for $C_{20}H_{28}NO^+$ ($[M+H]^+$): 298.2165, found: 298.2166.

FTIR (ATR, cm^{-1}): 3413 (w, broad), 2960 (m), 2931 (m), 2868 (m), 1618 (s).

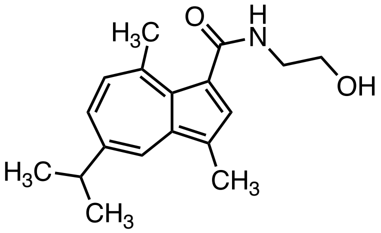

8h

***N*-(2-Hydroxyethyl)-5-isopropyl-3,8-dimethylazulene-1-carboxamide (8h).** Prepared according to **General Procedure B** using ethanolamine (0.5 mL, 8.3 mmol, 1.6 equiv) as the nucleophile. The crude product was purified via column chromatography (silica, 70 → 100% EtOAc in hexanes) to afford **8h** as a blue solid (54.9 mg, 0.192 mmol, 38% yield). Note that a higher amount of amine nucleophile was used in this case to lessen formation of a side product resulting from functionalizing the alcohol group with a second equivalent of the guaiazulene compound.

R_f: 0.27 in 70% EtOAc in hexanes.

^{1}H NMR (400 MHz, $CDCl_3$): δ 8.21 (d, *J* = 2.1 Hz, 1H), 7.65 (s, 1H), 7.47 (dd, *J* = 10.8, 2.1 Hz, 1H), 7.12 (d, *J* = 10.8 Hz, 1H), 6.36 (t, *J* = 6.1 Hz, 1H), 3.83 (t, *J* = 5.0 Hz, 2H), 3.66 – 3.58 (m, 2H), 3.17 – 3.02 (m, 2H), 2.89 (s, 3H), 2.59 (s, 3H), 1.36 (d, *J* = 6.9 Hz, 6H) ppm.

^{13}C{^{1}H} NMR (101 MHz, $CDCl_3$): δ 171.7, 146.7, 142.1, 138.3, 137.2, 136.0, 134.7, 133.6, 129.0, 124.0, 121.9, 63.1, 43.5, 38.1, 26.5, 24.8, 12.9 ppm.

HRMS: (DART/ORBITRAP) calculated for $C_{18}H_{24}NO_2^+$ ($[M+H]^+$): 286.1802, found: 286.1801.

FTIR (ATR, cm^{-1}): 3359 (w), 3299 (m), 2953 (m), 2951 (w), 2860 (w), 1607 (s), 1543 (s).

mp: 157–159 °C

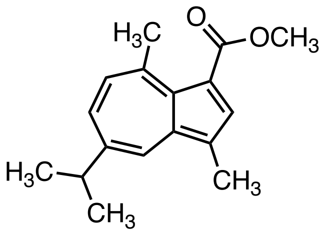

8i

Methyl 5-isopropyl-3,8-dimethylazulene-1-carboxylate (8i). Prepared according to **General Procedure B** using methanol (0.5 mL) as the nucleophile. The crude product was purified via column chromatography (silica, 5% EtOAc in hexanes) to afford **8i** as a purple oil (108.5 mg, 0.423 mmol, 84% yield).

***R_f*:** 0.28 in 5% EtOAc in hexanes; 0.67 in 70% EtOAc in hexanes.

^{1}H NMR (400 MHz, $CDCl_3$): δ 8.25 (d, *J* = 2.1 Hz, 1H), 7.95 (s, 1H), 7.53 (dd, *J* = 10.8, 2.1 Hz, 1H), 7.27 (d, *J* = 10.3 Hz, 1H), 3.91 (s, 3H), 3.12 (hept, *J* = 6.9 Hz, 1H), 2.97 (s, 3H), 2.59 (s, 3H), 1.37 (d, *J* = 6.9 Hz, 6H) ppm.

^{13}C{^{1}H} NMR (101 MHz, $CDCl_3$): δ 167.7, 147.4, 143.7, 140.5, 140.4, 136.3, 136.0, 134.5, 130.7, 124.1, 116.6, 51.8, 38.1, 27.9, 24.7, 12.9 ppm.

HRMS: (DART/ORBITRAP) calculated for $C_{17}H_{21}O_2^+$ ($[M+H]^+$): 257.1536, found: 257.1536.

FTIR (ATR, cm^{-1}): 2957 (m), 2866 (w), 1697 (s).

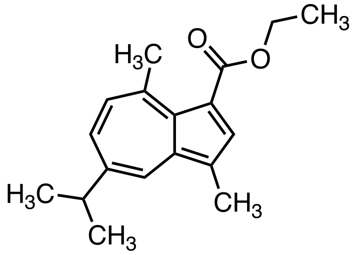

8j

Ethyl 5-isopropyl-3,8-dimethylazulene-1-carboxylate (8j). Prepared according to **General Procedure B** using ethanol (0.5 mL) as the nucleophile. The crude product was purified via column chromatography (silica, 5% EtOAc in hexanes) to afford **8j** as a purple oil (122.6 mg, 0.453 mmol, 90% yield).

***R*f:** 0.21 in 5% EtOAc in hexanes.

^{1}H NMR (400 MHz, $CDCl_3$): δ 8.25 (s, 1H), 7.96 (s, 1H), 7.52 (d, *J* = 10.9 Hz, 1H), 7.26 (d, *J* = 11.0 Hz, 1H), 4.39 (qd, *J* = 7.1, 1.6 Hz, 2H), 3.12 (hept, *J* = 6.9 Hz, 1H), 2.98 (s, 3H), 2.60 (s, 3H), 1.43 (t, *J* = 7.1 Hz, 3H), 1.38 (d, *J* = 7.0 Hz, 6H) ppm.

$^{13}C\{^1H\}$ NMR (101 MHz, $CDCl_3$): δ 167.4, 147.3, 143.5, 140.4, 140.3, 136.1, 135.9, 134.5, 130.5, 124.1, 117.3, 60.5, 38.1, 27.9, 24.7, 14.6, 12.9 ppm.

HRMS: (DART/ORBITRAP) calculated for $C_{18}H_{23}O_2^+$ ($[M+H]^+$): 271.1693, found: 271.1694.

FTIR (ATR, cm^{-1}): 2959 (m), 2929 (w), 2868 (w), 1696 (s).

8j was also prepared on 1 g scale according to the following procedure. A 100-mL round-bottomed flask was charged with a stir bar, guaiazulene **2** (1.00 g, 5.04 mmol, 1.00 equiv), dioxane (10 mL), and oxalyl chloride (0.450 mL, 5.32 mmol, 1.05 equiv). The reaction was heated to 70 ºC and stirred at this temperature for 1 h. The reaction was cooled in an ice/water bath while EtOH (5 mL) was added. The reaction was removed from the ice bath and stirred at rt for an additional 30 minutes. The reaction was quenched with water (50 mL), extracted with EtOAc (3 x 50 mL), dried over $MgSO_4$, filtered, and rotovapped to give a purple oil. The crude material was purified *via* column chromatography (silica, 0 → 5 % EtOAc in hexanes) to give **8j** as a purple oil (1.29 g, 4.77 mmol, 95% yield).

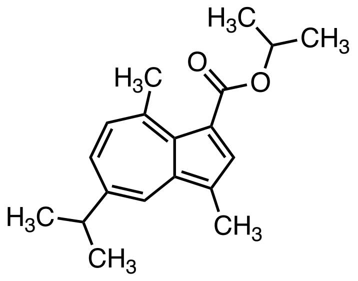

8k

Isopropyl 5-isopropyl-3,8-dimethylazulene-1-carboxylate (8k). Prepared according to **General Procedure B** using isopropanol (0.5 mL) as the nucleophile. The crude product was purified via column chromatography (silica, 5% EtOAc in hexanes) to afford **8k** as a purple oil (111.1 mg, 0.391 mmol, 79% yield).

***R*f:** 0.17 in 5% EtOAc in hexanes.

^{1}H NMR (400 MHz, CDCl$_3$): δ 8.23 (d, *J* = 2.1 Hz, 1H), 7.92 (s, 1H), 7.52 (dd, *J* = 10.8, 2.1 Hz, 1H), 7.25 (d, *J* = 11.4 Hz, 1H), 5.26 (hept, *J* = 6.3 Hz, 1H), 3.12 (hept, *J* = 6.9 Hz, 1H), 2.97 (s, 3H), 2.59 (d, *J* = 0.7 Hz, 3H), 1.40 (d, *J* = 6.3 Hz, 6H), 1.37 (d, *J* = 6.9 Hz, 6H) ppm.

^{13}C{^{1}H} NMR (101 MHz, CDCl$_3$):) δ 167.0, 147.2, 143.4, 140.3, 140.2, 135.9, 135.8, 134.5, 130.4, 124.0, 117.9, 67.7, 38.1, 27.9, 24.1, 22.2, 12.9 ppm.

HRMS: (DART/ORBITRAP) calculated for $C_{19}H_{25}O_2^+$ ([M+H]$^+$): 285.1849, found: 285.1848.

FTIR (ATR, cm^{-1}): 2955 (m), 2926 (w), 2862 (w), 1696 (s).

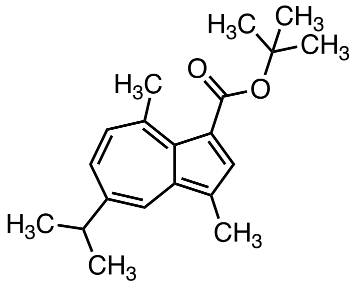

8l

***tert*-Butyl 5-isopropyl-3,8-dimethylazulene-1-carboxylate (8l).** Prepared according to **General Procedure B** using *tert*-butanol (0.5 mL) as the nucleophile. The crude product was purified via column chromatography (silica, 5% EtOAc in hexanes) to afford **8l** as a purple oil (111.8 mg, 0.375 mmol, 74% yield).

***R*$_f$:** 0.22 in 5% EtOAc in hexanes.

^{1}H NMR (400 MHz, CDCl$_3$): δ 8.23 (d, *J* = 2.1 Hz, 1H), 7.87 (s, 1H), 7.49 (dd, *J* = 10.8, 2.1 Hz, 1H), 7.21 (d, *J* = 10.8 Hz, 1H), 3.11 (hept, *J* = 6.9 Hz, 1H), 2.98 (s, 3H), 2.60 (s, 3H), 1.64 (s, 9H), 1.37 (d, *J* = 6.9 Hz, 6H) ppm.

^{13}C{^{1}H} NMR (101 MHz, CDCl$_3$): δ 167.3, 147.0, 143.0, 140.1, 139.9, 135.7, 135.2, 134.4, 129.9, 123.9, 119.6, 80.2, 38.1, 28.5, 27.8, 24.7, 12.9 ppm.

HRMS: (DART/ORBITRAP) calculated for $C_{20}H_{27}O_2^+$ ([M+H]$^+$): 299.2006, found: 299.2007.

FTIR (ATR, cm^{-1}): 2960 (m), 2929 (w), 2867 (m), 1694 (m).

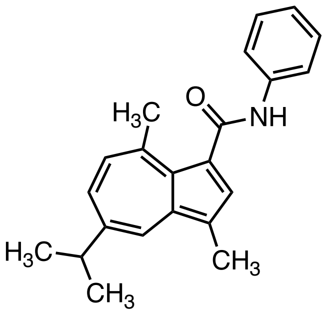

8n

5-Isopropyl-3,8-dimethyl-*N*-phenylazulene-1-carboxamide (8n). Prepared according to **General Procedure B** using aniline (0.10 mL, 1.1 mmol, 2.2 equiv) as the nucleophile as well as triethylamine (0.15 mL, 1.1 mmol, 2.2 equiv) as an added base. The crude product was purified via column chromatography (silica, 10% EtOAc in hexanes) to afford **8n** as a dark blue solid (130.9 mg, 0.412 mmol, 82% yield).

R_f: 0.42 in 30% EtOAc in hexanes; 0.74 in 70% EtOAc in hexanes.

^{1}H NMR (400 MHz, $CDCl_3$): δ 8.26 (d, *J* = 2.1 Hz, 1H), 7.77 (s, 1H), 7.67 – 7.61 (m, 3H), 7.51 (dd, *J* = 10.8, 2.1 Hz, 1H), 7.36 (t, *J* = 7.8 Hz, 2H), 7.17 (d, *J* = 10.8 Hz, 1H), 7.15 – 7.10 (m, 1H), 3.12 (hept, *J* = 6.9 Hz, 1H), 2.94 (s, 3H), 2.63 (s, 3H), 1.39 (d, *J* = 6.9 Hz, 6H) ppm.

^{13}C{^{1}H} NMR (101 MHz, $CDCl_3$): δ 167.9, 146.9, 142.5, 138.9, 138.5, 137.2, 136.2, 134.8, 133.9, 129.24, 129.21, 124.23, 124.15, 122.7, 119.7, 38.2, 26.8, 24.8, 13.0 ppm.

HRMS: (DART/ORBITRAP) calculated for $C_{22}H_{24}NO^+$ ($[M+H]^+$): 318.1852, found: 318.1855.

FTIR (ATR, cm^{-1}): 3321 (m), 3045 (w), 2957 (m), 2925 (w), 2864 (w), 1637 (m), 1595 (m).

mp: 172–175 °C

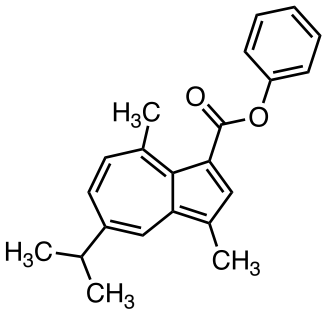

8o

Phenyl 5-isopropyl-3,8-dimethylazulene-1-carboxylate (8o). Prepared according to **General Procedure B** using phenol (102 mg, 1.1 mmol, 2.2 equiv) as the nucleophile as well as triethylamine (0.15 mL, 1.1 mmol, 2.2 equiv) as an added base. The crude product was purified via column chromatography (silica, 10% EtOAc in hexanes) to afford **8o** as a purple oil that solidified upon standing (116.4 mg, 0.366 mmol, 73% yield).

***R_f*:** 0.30 in 5% EtOAc in hexanes.

^{1}H NMR (400 MHz, $CDCl_3$): δ 8.30 (d, *J* = 2.1 Hz, 1H), 8.21 (s, 1H), 7.59 (dd, *J* = 10.9, 2.2 Hz, 1H), 7.50 – 7.40 (m, 2H), 7.36 (d, *J* = 10.9 Hz, 1H), 7.32 – 7.22 (m, 3H), 3.16 (hept, *J* = 6.9 Hz, 1H), 3.07 (s, 3H), 2.63 (s, 3H), 1.39 (d, *J* = 6.9 Hz, 6H) ppm.

^{13}C{^{1}H} NMR (101 MHz, $CDCl_3$): δ 164.6, 151.8, 148.3, 144.7, 141.6, 141.4, 137.9, 136.3, 134.7, 131.8, 129.5, 125.4, 124.4, 122.1, 115.3, 38.1, 28.5, 24.7, 13.0 ppm.

HRMS: (DART/ORBITRAP) calculated for $C_{22}H_{23}O_2^+$ ($[M+H]^+$): 319.1693, found: 319.1694.

FTIR (ATR, cm^{-1}): 2974 (w), 2955 (w), 2925 (w), 2863 (w), 1717 (s).

mp: 55–57 °C

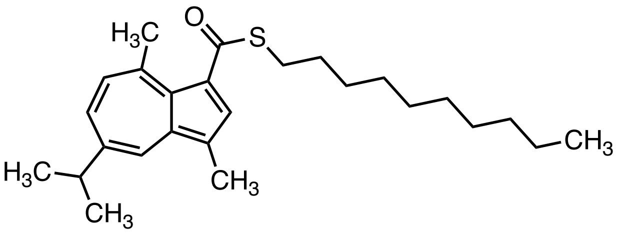

8p

***S*-Decyl 5-isopropyl-3,8-dimethylazulene-1-carbothioate (8p).** Prepared according to **General Procedure B** using 1-decanethiol (0.15 mL, 0.72 mmol, 1.4 equiv) as the nucleophile as well as triethylamine (0.15 mL, 1.1 mmol, 2.2 equiv) as an added base. The crude product was purified via column chromatography (silica, 10% EtOAc in hexanes) to afford **8p** as a purple oil (82.7 mg, 0.207 mmol, 41% yield). Note that a lower amount of the nucleophile was used in this example compared to the usual conditions because residual 1-decanethiol in the reaction mixture proved challenging to separate from **8p** *via* column chromatography. This change contributes to the lower yield reported for this substrate.

R_f: 0.44 in 5% EtOAc in hexanes.

^{1}H NMR (400 MHz, $CDCl_3$): δ 8.25 (d, *J* = 2.1 Hz, 1H), 8.01 (s, 1H), 7.54 (dd, *J* = 10.9, 2.2 Hz, 1H), 7.26 (s, 1H), 3.19 – 3.03 (m, 3H), 2.87 (s, 3H), 2.60 (s, 3H), 1.71 (p, *J* = 7.3 Hz, 2H), 1.52 – 1.41 (m, 2H), 1.37 (d, *J* = 6.9 Hz, 6H), 1.33 – 1.26 (m, 12H), 0.93 – 0.85 (m, 3H) ppm.

^{13}C{^{1}H} NMR (101 MHz, $CDCl_3$): δ 190.4, 148.1, 144.0, 140.7, 139.9, 136.4, 134.8, 134.5, 131.2, 125.6, 124.2, 38.2, 32.1, 30.2, 30.0, 29.7, 29.5, 29.4, 29.1, 27.9, 24.7, 22.8, 14.3, 13.0 ppm. Note that the peak at δ 29.7 ppm represents two overlapping carbon signals, as determined by quantitative ^{13}C NMR spectroscopy.

HRMS: (DART/ORBITRAP) calculated for $C_{26}H_{39}OS^{+}$ ($[M+H]^{+}$): 399.2716, found: 399.2715.

FTIR (ATR, cm^{-1}): 2957 (w), 2922 (m), 2852 (m), 1648 (m).

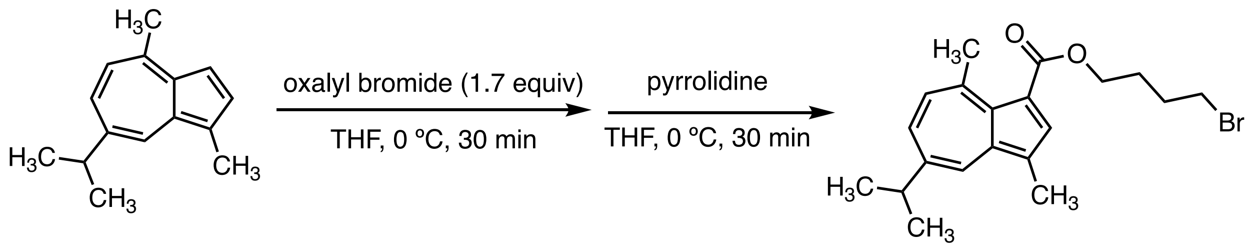

2 **9**

4-Bromobutyl 2-(azulen-1-yl)-2-oxoacetate (9). A 1-dram vial was charged with a stir bar, guaiazulene (100. mg, 0.504 mmol, 1.00 equiv), and THF (1 mL, dried over sieves). The vial was cooled to 0 °C in an ice/water bath, oxalyl bromide (0.12 mL, 0.85 mmol, 1.7 equiv) was added *via* syringe, and the reaction was stirred at 0 °C for 30 min. Pyrrolidine (0.5 mL) was added, and the reaction was stirred for an additional 30 min at 0 °C. The contents of the reaction vial were transferred to a separatory funnel with water (40 mL) and ethyl acetate (30 mL). The layers were separated, then the aqueous layer was extracted with additional ethyl acetate (2 x 30 mL). The combined organic extracts were dried over $MgSO_4$, filtered, and concentrated on the rotary evaporator. The resulting crude material was purified *via* column chromatography (silica, 30 → 50 → 70 → 100% EtOAc in hexanes) to afford **9** as a purple oil (116 mg, 0.307 mmol, 61% yield).

***R_f*:** 0.86 in 50% EtOAc in hexanes.

^{1}H NMR (400 MHz, $CDCl_3$): δ 8.25 (d, *J* = 2.2 Hz, 1H), 7.94 (s, 1H), 7.53 (dd, *J* = 10.9, 2.1 Hz, 1H), 7.26 (s, 1H), 4.35 (t, *J* = 6.3 Hz, 2H), 3.50 (t, *J* = 6.6 Hz, 2H), 3.11 (hept, *J* = 6.8 Hz, 1H), 2.97 (s, 3H), 2.59 (s, 3H), 2.14 – 2.01 (m, 2H), 2.00 – 1.90 (m, 2H), 1.37 (d, *J* = 6.9 Hz, 6H) ppm.

^{13}C{^{1}H} NMR (125 MHz, $CDCl_3$): δ 167.0, 147.5, 143.8, 140.6, 140.4, 136.4, 136.0, 134.6, 130.8, 124.1, 116.7, 63.5, 38.1, 33.5, 29.7, 28.1, 27.8, 24.7, 13.0 ppm.

HRMS: (DART/ORBITRAP) calculated for $C_{20}H_{26}BrO_2^+$ ($[M+H]^+$): 377.1111, found: 377.1107.

FTIR (ATR, cm^{-1}): 2957 (w), 2925 (w), 2867 (w), 1696 (m), 1186 (s).

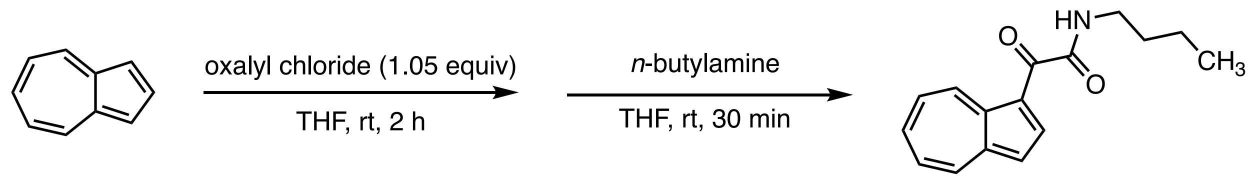

1 **10a**

2-(Azulen-1-yl)-*N*-butyl-2-oxoacetamide (10a). A 1-dram vial was charged with a stir bar, azulene **1** (32.0 mg, 0.250 mmol, 1.00 equiv), THF (0.5 mL), and oxalyl chloride (22.0 µL, 0.260 mmol, 1.05 equiv). The reaction was stirred at rt for 2 h. *n*-Butylamine (0.10 mL, 1.0 mmol, 4.0 equiv) was added, and the reaction was stirred at rt for an additional 30 min. The contents of the reaction vial were transferred to a separatory funnel with water (30 mL) and ethyl acetate (30 mL). The layers were separated, then the aqueous layer was extracted with additional ethyl acetate (2 x 30 mL). The combined organic extracts were dried over $MgSO_4$, filtered, and concentrated on the rotary evaporator to give a dark red oil. The resulting crude material was purified via column chromatography (silica, 20% EtOAc in hexanes) to afford **10a** as a dark red oil (37.3 mg, 0.146 mmol, 58% yield).

***R*f:** 0.56 in 50% EtOAc in hexanes.

^{1}H NMR (400 MHz, $CDCl_3$): δ 9.84 (d, *J* = 9.8 Hz, 1H), 9.23 (d, *J* = 4.3 Hz, 1H), 8.51 (d, *J* = 8.6 Hz, 1H), 7.88 (t, *J* = 9.8 Hz, 1H), 7.71 (t, *J* = 9.9 Hz, 1H), 7.59 (t, *J* = 9.2 Hz, 1H), 7.44 (broad s, 1H), 7.31 (d, *J* = 4.3 Hz, 1H), 3.43 (td, *J* = 7.1, 6.0 Hz, 2H), 1.69 – 1.56 (m, 2H), 1.44 (ddt, *J* = 14.5, 9.7, 7.3 Hz, 2H), 0.97 (t, *J* = 7.3 Hz, 3H) ppm.

^{13}C{^{1}H} NMR (101 MHz, $CDCl_3$): δ 182.5, 163.7, 147.1, 145.1, 144.0, 139.7, 139.2, 138.5, 130.8, 129.4, 121.3, 119.8, 39.2, 31.6, 20.3, 13.9 ppm.

HRMS: (DART/ORBITRAP) calculated for $C_{16}H_{18}NO_2^+$ ($[M+H]^+$): 256.1332, found: 256.1333.

FTIR (ATR, cm^{-1}): 3342 (w), 2956 (w), 2929 (w), 2870.2 (w), 1666.(m), 1611 (m).

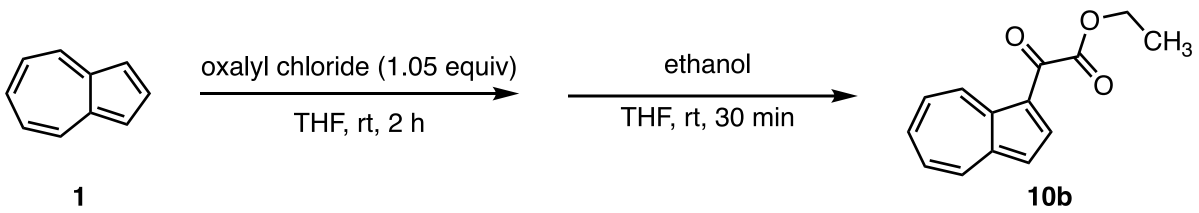

Ethyl 2-(azulen-1-yl)-2-oxoacetate (10b). A 1-dram vial was charged with a stir bar, azulene **1** (32.0 mg, 0.250 mmol, 1.00 equiv), THF (0.5 mL), and oxalyl chloride (22.0 µL, 0.26.0 mmol, 1.05 equiv). The reaction was stirred at rt for 2 h. Ethanol (0.25 mL) was added, and the reaction was stirred at rt for an additional 30 min. The contents of the reaction vial were transferred to a separatory funnel with water (30 mL) and ethyl acetate (30 mL). The layers were separated, then the aqueous layer was extracted with additional ethyl acetate (2 x 30 mL). The combined organic extracts were dried over $MgSO_4$, filtered, and concentrated on the rotary evaporator to give a red oil. The resulting crude material was purified via column chromatography (silica, 20% EtOAc in hexanes) to afford **10b** as a red oil (38.5 mg, 0.169 mmol, 68% yield).

***R_f*:** 0.45 in 50% EtOAc in hexanes.

^{1}H NMR (400 MHz, $CDCl_3$): δ 9.86 (d, *J* = 9.8 Hz, 1H), 8.52 (d, *J* = 9.7 Hz, 1H), 8.43 (d, *J* = 4.3 Hz, 1H), 7.91 (t, *J* = 9.7 Hz, 1H), 7.73 (t, *J* = 9.9 Hz, 1H), 7.61 (t, *J* = 9.8 Hz, 1H), 7.29 (d, *J* = 4.3 Hz, 1H), 4.46 (q, *J* = 7.1 Hz, 2H), 1.45 (t, *J* = 7.1 Hz, 3H) ppm.

^{13}C{^{1}H} NMR (101 MHz, $CDCl_3$): δ 181.4, 164.7, 147.3, 142.9, 142.9, 140.3, 139.8, 139.1, 131.2, 129.6, 120.7, 119.4, 62.0, 14.3 ppm.

HRMS: (DART/ORBITRAP) calculated for $C_{14}H_{13}O_3^+$ ($[M+H]^+$): 229.0859, found: 229.0861.

FTIR (ATR, cm^{-1}): 2981 (w), 2935 (w), 1725 (m), 1623 (s).

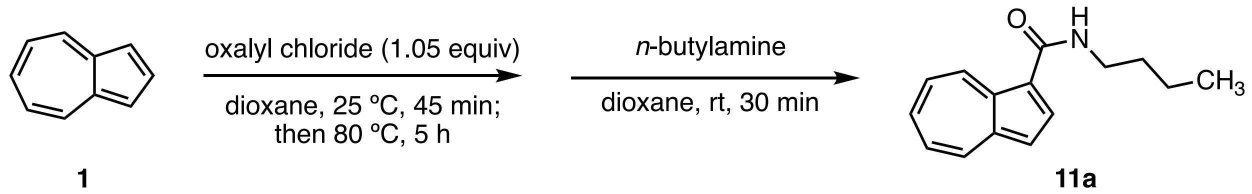

N-Butylazulene-1-carboxamide (11a). A 1-dram vial was charged with a stir bar, azulene **1** (32 mg, 0.25 mmol, 1.0 equiv), dioxane (0.5 mL), and oxalyl chloride (22.0 µL, 0.260 mmol, 1.05 equiv). The reaction was allowed to stir at rt for 1 h, then at 90 ºC for 4 h. The reaction was cooled to rt, then butylamine (0.10 mL, 1.0 mmol, 4.0 equiv) was added. The reaction was stirred for an additional 30 minutes, and the reaction mixture was transferred to a separatory funnel with water (20 mL) and EtOAc (20 mL). They layers were separated and the aqueous layer was extracted with additional EtOAc (2 x 20 mL). The combined organic extracts were dried over $MgSO_4$, filtered, and concentrated on the rotary evaporator to provide a purple solid. The resulting crude material was purified via column chromatography (silica, 20% EtOAc in hexanes) to afford **11a** as a purple solid (46 mg, 2.0 mmol, 81% yield).

***R_f*:** 0.53 in 50% EtOAc in hexanes.

^{1}H NMR (400 MHz, $CDCl_3$): δ 9.70 (d, *J* = 9.9 Hz, 1H), 8.40 (d, *J* = 10.6 Hz, 1H), 8.01 (d, *J* = 4.1 Hz, 1H), 7.73 (t, *J* = 9.9 Hz, 1H), 7.43 (t, *J* = 9.9 Hz, 1H), 7.33 (t, *J* = 9.7 Hz, 1H), 7.25 (d, *J* = 4.0 Hz, 1H), 6.24 (broad s, 1H), 3.50 (td, *J* = 7.2, 5.7 Hz, 2H), 1.63 (p, *J* = 7.7 Hz, 2H), 1.43 (h, *J* = 7.3 Hz, 2H), 0.96 (t, *J* = 7.3 Hz, 3H) ppm.

^{13}C{^{1}H} NMR (101 MHz, $CDCl_3$): δ 166.4, 143.4, 139.3, 139.2, 138.7, 138.3, 135.1, 126.7, 125.6, 121.1, 116.9, 39.5, 32.2, 20.4, 14.0 ppm.

HRMS: (DART/ORBITRAP) calculated for $C_{15}H_{18}NO^+$ ($[M+H]^+$): 228.1383, found: 228.1383.

FTIR (ATR, cm^{-1}): 3283 (m), 2959 (m), 2924 (m), 2857 (m), 1684 (w), 1616 (m).

mp: 65–67 °C

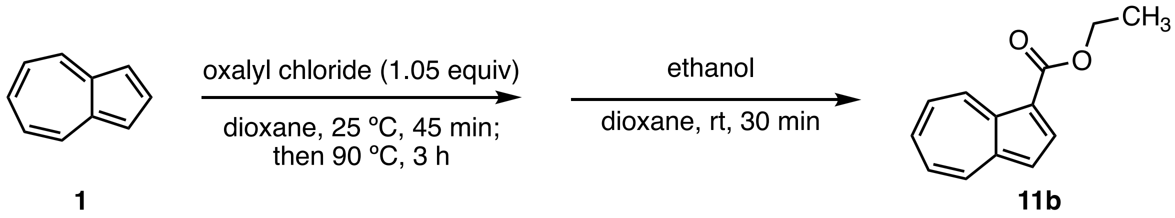

Ethyl azulene-1-carboxylate (11b). A 1-dram vial was charged with a stir bar, azulene **1** (32 mg, 0.25 mmol, 1.0 equiv), dioxane (0.5 mL), and oxalyl chloride (22.0 μL, 0.260 mmol, 1.05 equiv). The reaction was allowed to stir at rt for 1 h, then at 90 ºC for 4 h. The reaction was cooled to rt, then ethanol (0.25 mL) was added. The reaction was stirred for an additional 30 minutes, and the reaction mixture was transferred to a separatory funnel with water (20 mL) and EtOAc (20 mL). They layers were separated and the aqueous layer was extracted with additional EtOAc (2 x 20 mL). The combined organic extracts were dried over $MgSO_4$, filtered, and concentrated on the rotary evaporator to provide a purple solid. The resulting crude material was purified via column chromatography (silica, 10% EtOAc in hexanes) to afford **11b** as a purple solid (47 mg, 0.24 mmol, 94% yield).

***R*~f~:** 0.48 in 20% EtOAc in hexanes.

^{1}H NMR (400 MHz, $CDCl_3$): δ 9.66 (d, *J* = 9.9 Hz, 1H), 8.44 (d, *J* = 8.5 Hz, 1H), 8.39 (d, *J* = 4.1 Hz, 1H), 7.79 (t, *J* = 9.8 Hz, 1H), 7.54 (t, *J* = 9.9 Hz, 1H), 7.43 (t, *J* = 9.7 Hz, 1H), 7.29 (d, *J* = 4.1 Hz, 1H), 4.44 (q, *J* = 7.1 Hz, 2H), 1.45 (t, *J* = 7.1 Hz, 3H) ppm.

^{13}C{^{1}H} NMR (101 MHz, $CDCl_3$): δ 165.6, 144.8, 140.8, 140.3, 139.0, 138.3, 137.9, 127.7, 126.7, 117.7, 117.2, 59.9, 14.7 ppm.

HRMS: (DART/ORBITRAP) calculated for $C_{13}H_{13}O_2^+$ ($[M+H]^+$): 201.0910, found: 201.0910.

FTIR (ATR, cm^{-1}): 2979 (w), 1682 (s).

II.c. General Kinetics Procedures

General Procedure C: No-D NMR Kinetics for Guaiazulene + Oxalyl Chloride

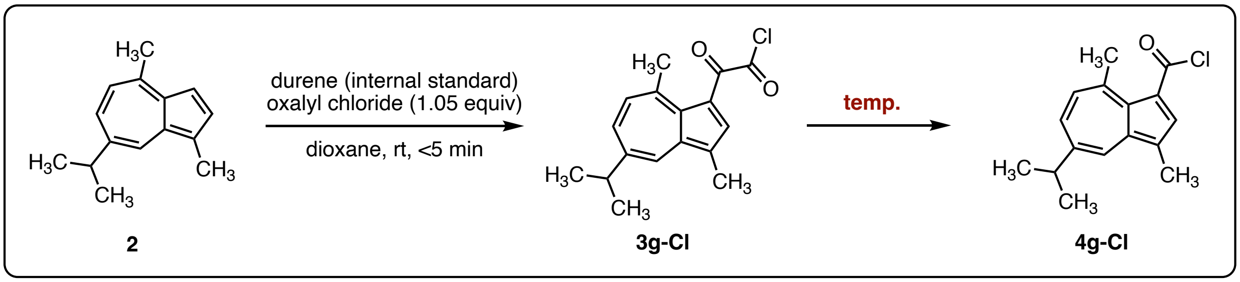

A screw-cap NMR tube was charged with guaiazulene **2** (49.0 mg, 0.247 mmol, 1.00 equiv) and durene (6–8 mg) as an internal standard. Dry dioxane (0.5 mL) was added, and the tube was capped and inverted several times to dissolve the solids. A preliminary no-D ^{1}H NMR spectrum was acquired at rt to quantify the initial compound ratio based on the integration of guaiazulene **2** (δ 8.1 ppm) relative to durene (δ 6.8 ppm). The tube was removed from the NMR, oxalyl chloride (22.0 µL, 0.260 mmol, 1.05 equiv) was added via syringe, and the tube was inverted several times to mix. Time *t* = 0 was defined as the time of oxalyl chloride addition. The tube was returned to the NMR and heated to the target temperature (25–55 ºC), and no-D ^{1}H NMR spectra were acquired periodically until ~90% conversion was reached. In each case, the initial conversion of **2** to **3g-Cl** was complete by the time the first spectrum was acquired (typically ~5 min). The integration of **3g-Cl** (δ 7.9 ppm) and **4g-Cl** (δ 8.2 ppm) relative to durene (δ 6.8 ppm) was used to quantify concentration of starting material and product over the course of the reaction.

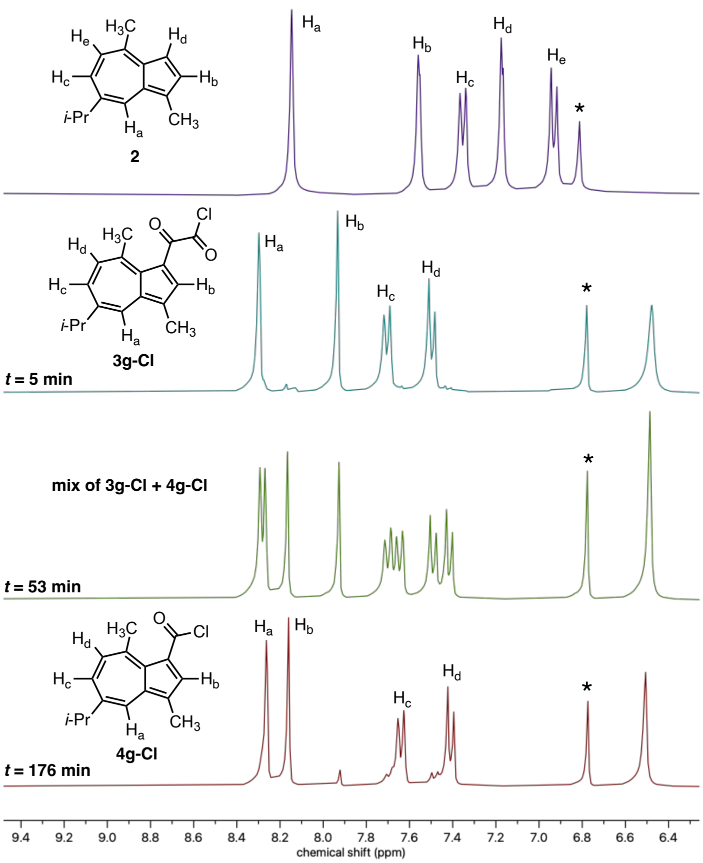

Figure S1. Example no-D ^{1}H NMR spectra with peak assignments for reaction of **2** with oxalyl chloride at 35 ºC. The signal marked with the asterisk represents the durene internal standard.

General Procedure D: No-D NMR Kinetics for Azulene + Oxalyl Chloride

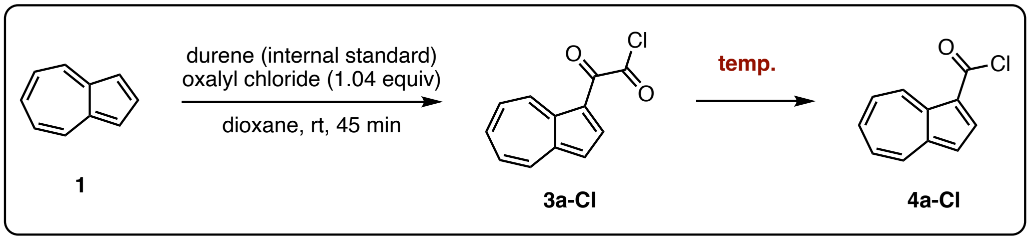

A screw-cap NMR tube was charged with azulene **1** (32.0 mg, 0.250 mmol, 1.0 equiv) and durene (6–8 mg) as an internal standard. Dry dioxane (0.5 mL) was added, and the tube was capped and inverted several times to dissolve the solids. A preliminary no-D ^{1}H NMR spectrum was acquired at rt to quantify the initial compound ratio based on the integration of azulene **1** (δ 7.9 ppm) relative to durene (δ 6.8 ppm). The tube was removed from the NMR, oxalyl chloride (22.0 µL,

0.260 mmol, 1.04 equiv) was added via syringe, and the tube was inverted several times to mix. The tube was returned to the NMR and set at 25 ºC for 45–50 min, at which point no-D ^{1}H NMR indicated conversion of **1** to **3a-Cl** but no formation of **4a-Cl**. The sample was heated to the target temperature (75–90 ºC), and this point was defined as time *t* = 0. No-D ^{1}H NMR spectra were acquired periodically until ~90% conversion was reached. The integration of **3a-Cl** (δ 9.7 ppm) and **4a-Cl** (δ 9.3 ppm) relative to durene (δ 6.8 ppm) was used to quantify concentration of starting material and product over the course of the reaction.

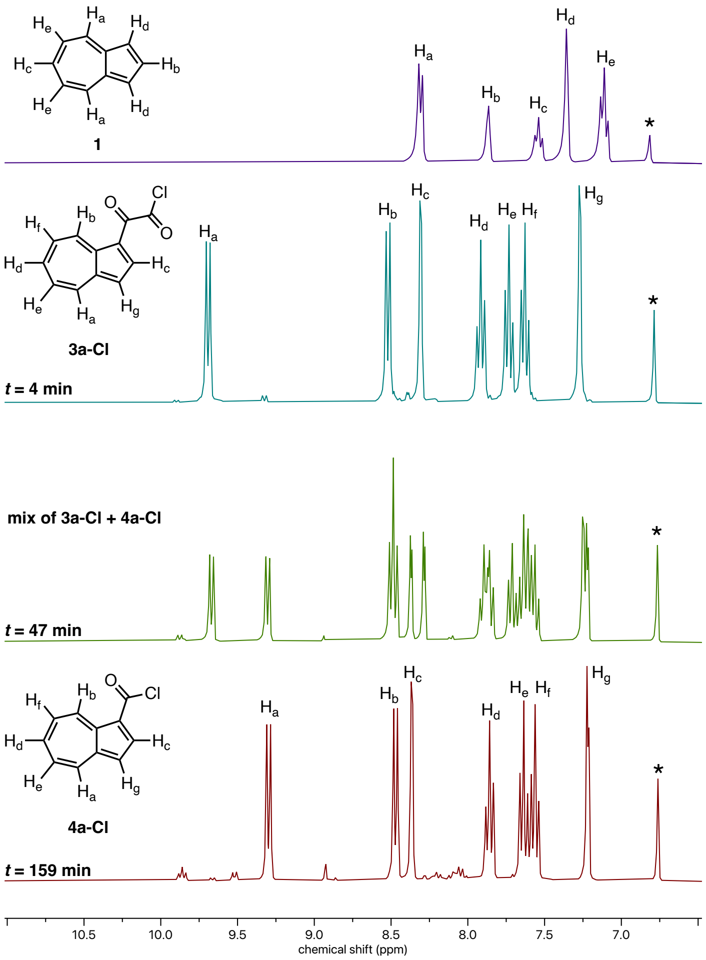

Figure S2. Example no-D ^{1}H NMR spectra with peak assignments for reaction of **1** with oxalyl chloride at 85 ºC. The signal marked with the asterisk represents the durene internal standard.

General Procedure E: No-D NMR Kinetics for Azulene + Oxalyl Bromide

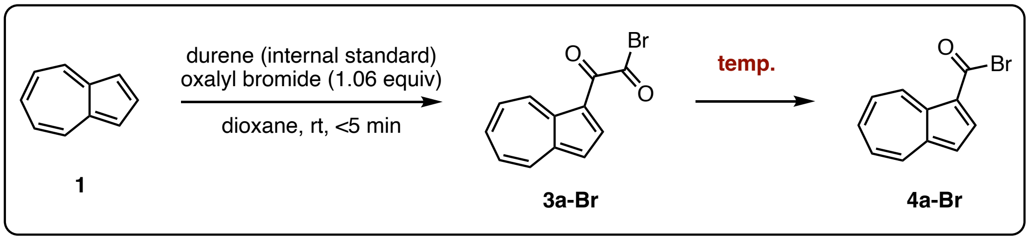

A screw-cap NMR tube was charged with azulene **1** (32.0 mg, 0.250 mmol, 1.0 equiv) and durene (6–8 mg) as an internal standard. Dry dioxane (0.5 mL) was added, and the tube was capped and inverted several times to dissolve the solids. A preliminary no-D ^{1}H NMR spectrum was acquired at rt to quantify the initial compound ratio based on the integration of azulene **1** (δ 7.9 ppm) relative to durene (δ 6.8 ppm). The tube was removed from the NMR, oxalyl bromide (25.0 µL, 0.266 mmol, 1.06 equiv) was added via syringe, and the tube was inverted several times to mix. Time *t* = 0 was defined as the time of oxalyl bromide addition. The tube was returned to the NMR and heated to the target temperature (20–35 ºC), and no-D ^{1}H NMR spectra were acquired periodically until ~90% conversion was reached. In each case, the initial conversion of **1** to **3a-Br** was complete by the time the first spectrum was acquired (typically ~5 min). The integration of **3a-Br** (δ 9.6 ppm) and **4a-Br** (δ 9.2 ppm) relative to durene (δ 6.8 ppm) was used to quantify concentration of starting material and product over the course of the reaction.

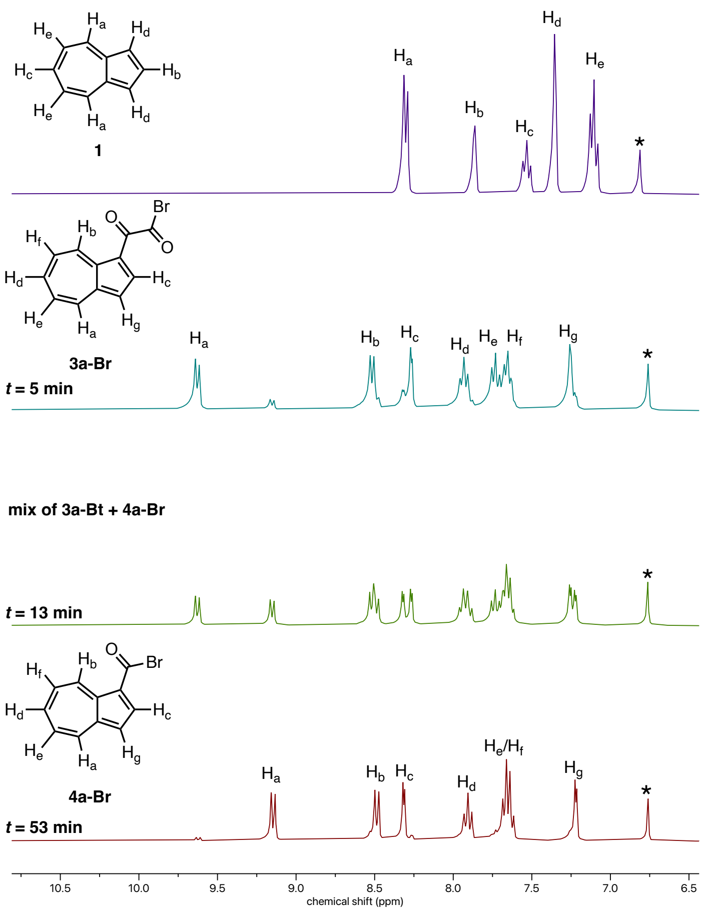

Figure S3. Example no-D ^{1}H NMR spectra with peak assignments for reaction of **1** with oxalyl bromide at 30 ºC. The signal marked with the asterisk represents the durene internal standard.

II.d. Kinetics Data for Guaiazulene Reactions with Oxalyl Chloride

Table S1. Kinetic data for reaction of **3g-Cl** at 25 ºC.

ID	time (min)	[3g-Cl] (M)	ln[3g-Cl]	$[3g\text{-}Cl]^{-1}$ (M^{-1})	[4g-Cl] (M)
A	4.9	0.439	-0.82	2.3	0.016
B	12.3	0.429	-0.85	2.3	0.031
C	24.0	0.413	-0.88	2.4	0.058
D	34.9	0.389	-0.94	2.6	0.083
E	50.0	0.350	-1.05	2.9	0.109
F	76.4	0.311	-1.17	3.2	0.159
G	102	0.264	-1.33	3.8	0.203
H	138	0.219	-1.52	4.6	0.247
I	174	0.177	-1.73	5.6	0.279
J	215	0.141	-1.96	7.1	0.313
K	248	0.117	-2.15	8.5	0.336
L	346	0.069	-2.67	14.5	0.371
M	502	0.029	-3.54	34.5	0.418

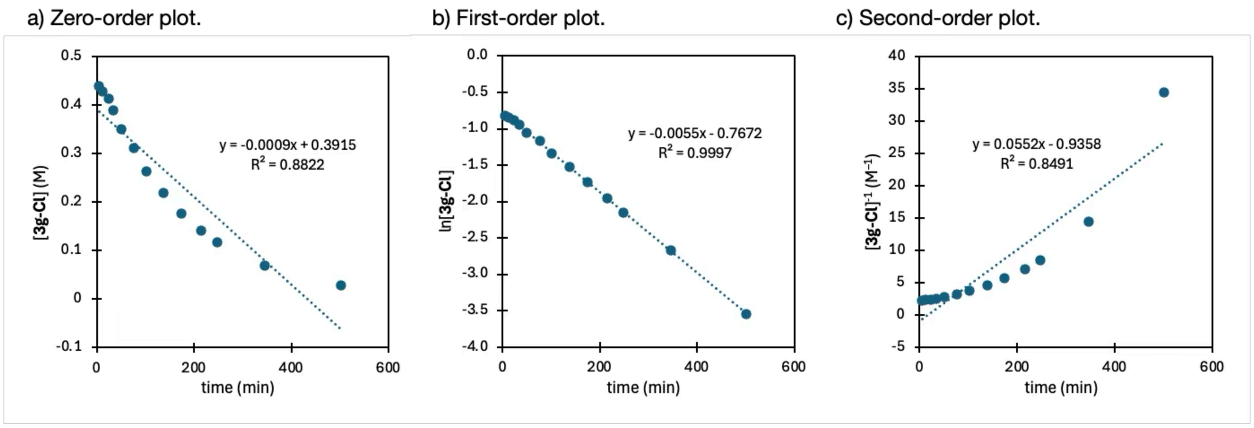

Figure S4. Kinetic plots for reaction of **3g-Cl** at 25 ºC.

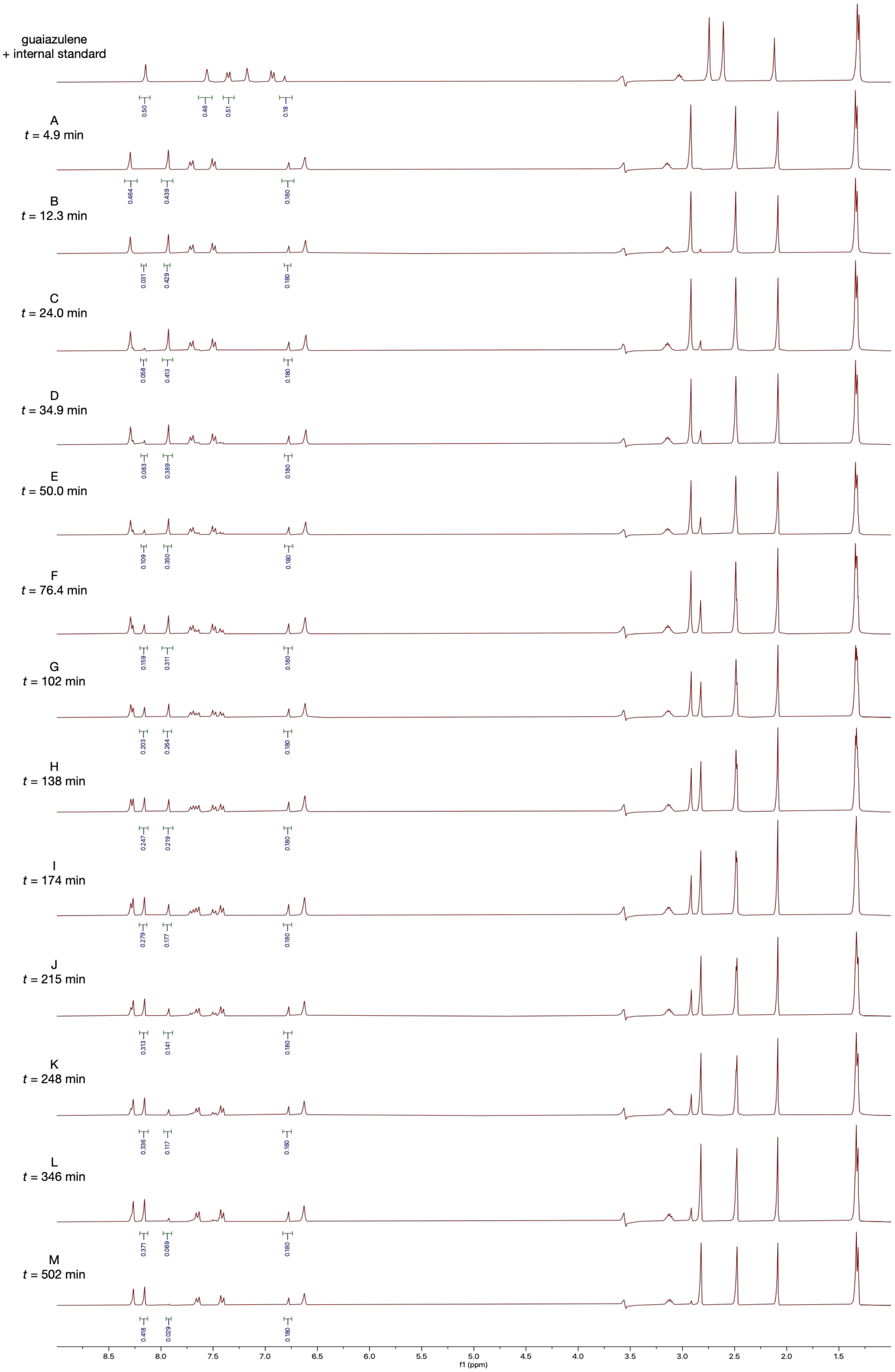

Figure S5. No-D ^{1}H NMR data for reaction of **3g-Cl** at 25 ºC.

Table S2. Kinetic data for reaction of **3g-Cl** at 35 ºC.

ID	time (min)	[3g-Cl] (M)	ln[3g-Cl]	$[3g\text{-}Cl]^{-1}$ (M^{-1})	[4g-Cl] (M)
A	5.3	0.449	-0.80	2.2	0.008
B	9.9	0.435	-0.83	2.3	0.031
C	23.7	0.349	-1.05	2.9	0.109
D	31.2	0.333	-1.10	3.0	0.160
E	42.3	0.262	-1.34	3.8	0.200
F	53.6	0.225	-1.49	4.4	0.242
G	73.5	0.163	-1.81	6.1	0.309
H	91.6	0.115	-2.16	8.7	0.347
I	113	0.082	-2.50	12.2	0.361
J	135	0.054	-2.92	18.5	0.418
K	158	0.036	-3.32	27.8	0.434
L	176	0.025	-3.69	40.0	0.428

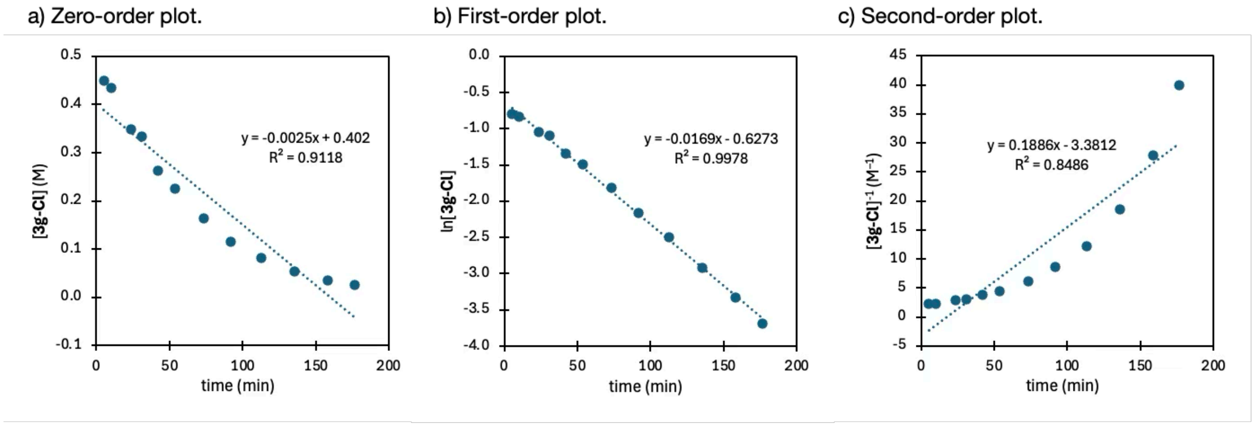

Figure S6. Kinetic plots for reaction of **3g-Cl** at 35 ºC.

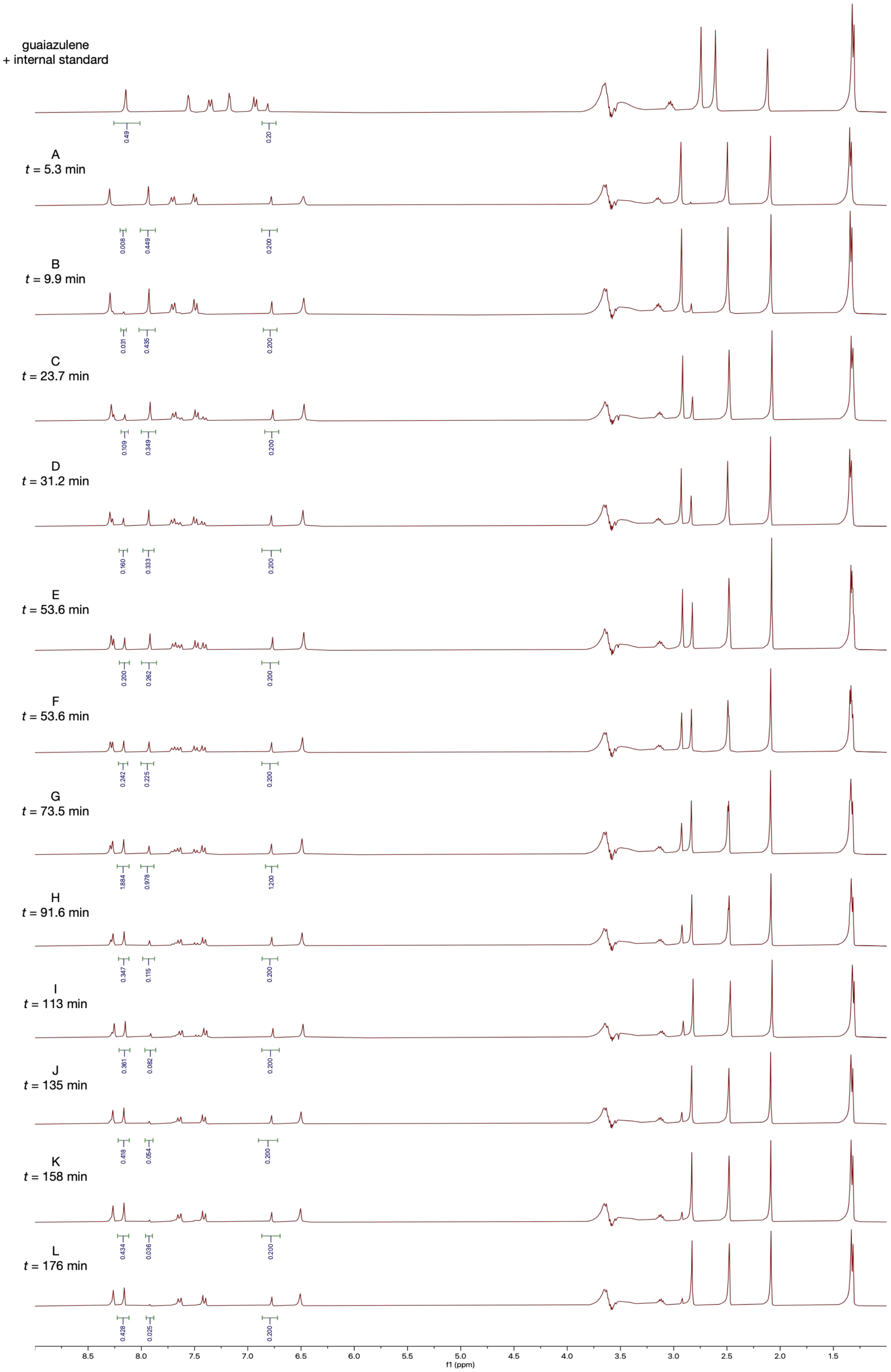

Figure S7. No-D ^{1}H NMR data for reaction of **3g-Cl** at 35 ºC.

Table S3. Kinetic data for reaction of **3g-Cl** at 45 ºC.

ID	time (min)	[3g-Cl] (M)	ln[3g-Cl]	$[3g\text{-}Cl]^{-1}$ (M^{-1})	[4g-Cl] (M)
A	5.1	0.409	-0.89	2.4	0.047
B	9.6	0.369	-1.00	2.7	0.111
C	14.1	0.311	-1.17	3.2	0.168
D	18.7	0.268	-1.32	3.7	0.218
E	23.2	0.219	-1.52	4.6	0.249
F	27.7	0.188	-1.67	5.3	0.289
G	32.4	0.153	-1.88	6.5	0.305
H	37.0	0.133	-2.02	7.5	0.332
I	41.5	0.111	-2.20	9.0	0.349
J	59.4	0.061	-2.80	16.4	0.389
K	68.3	0.043	-3.15	23.3	0.407
L	80.8	0.029	-3.54	34.5	0.423
M	98.7	0.016	-4.14	62.5	0.432

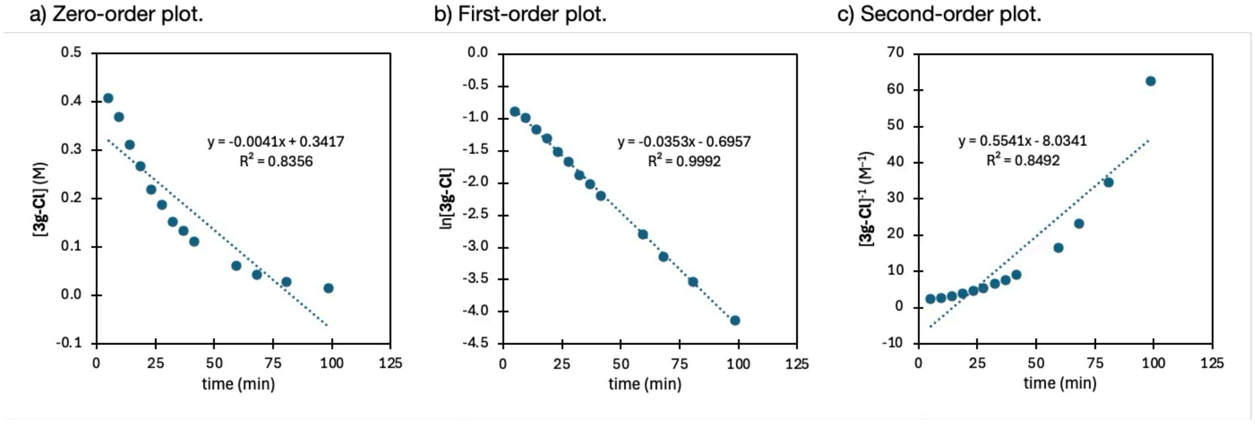

Figure S8. Kinetic plots for reaction of **3g-Cl** at 45 ºC.

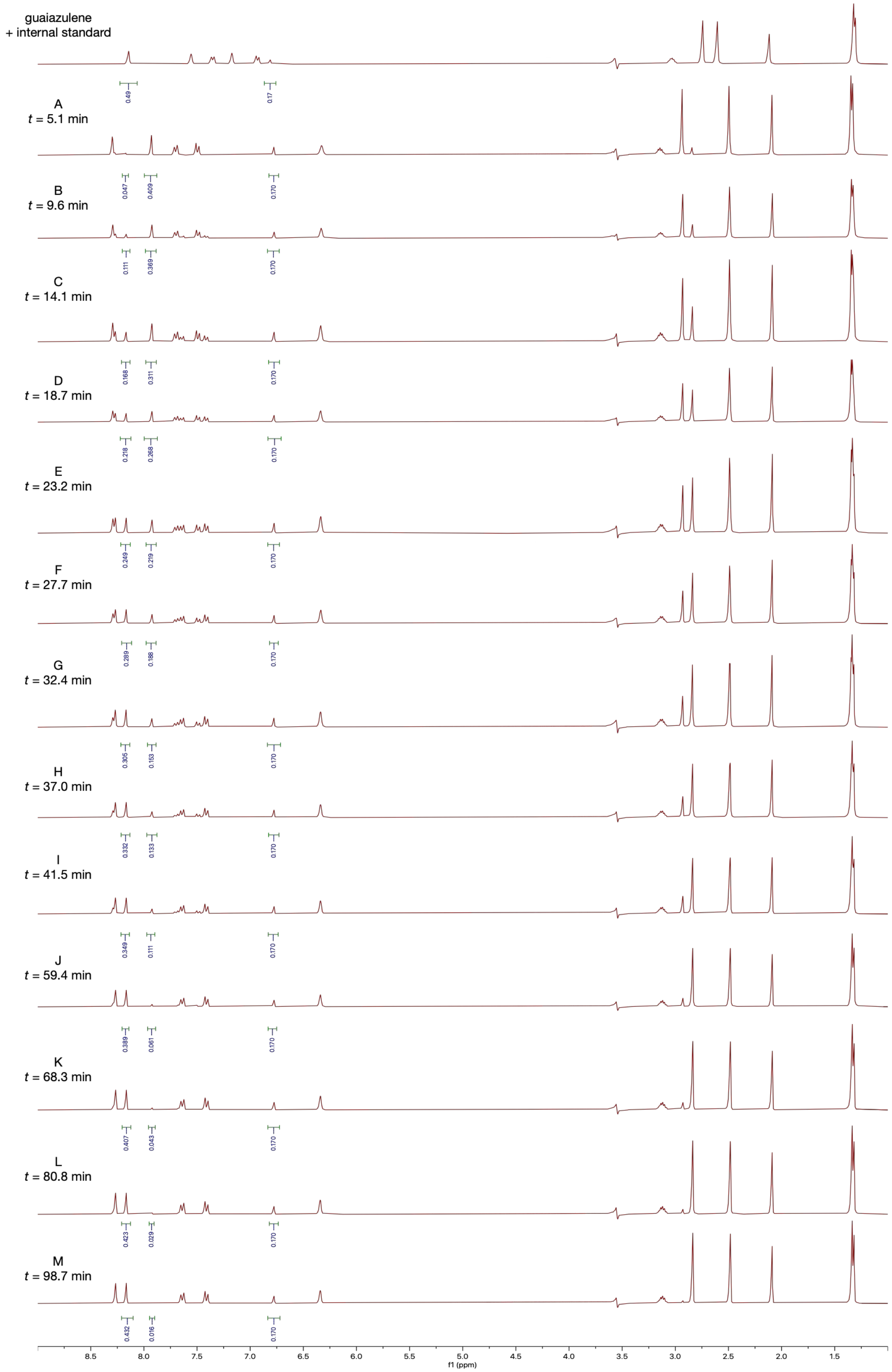

Figure S9. No-D ^{1}H NMR data for reaction of **3g-Cl** at 45 ºC.

Table S4. Kinetic data for reaction of **3g-Cl** at 55 ºC.

ID	time (min)	[3g-Cl] (M)	ln[3g-Cl]	$[3g\text{-}Cl]^{-1}$ (M^{-1})	[4g-Cl] (M)
A	5.2	0.409	-0.98	2.7	0.106
B	9.8	0.369	-1.47	4.3	0.217
C	14.2	0.311	-1.87	6.5	0.293
D	18.6	0.268	-2.34	10.4	0.343
E	23.2	0.219	-2.78	16.1	0.354
F	27.8	0.188	-3.19	24.4	0.372
G	32.8	0.153	-3.77	43.5	0.410

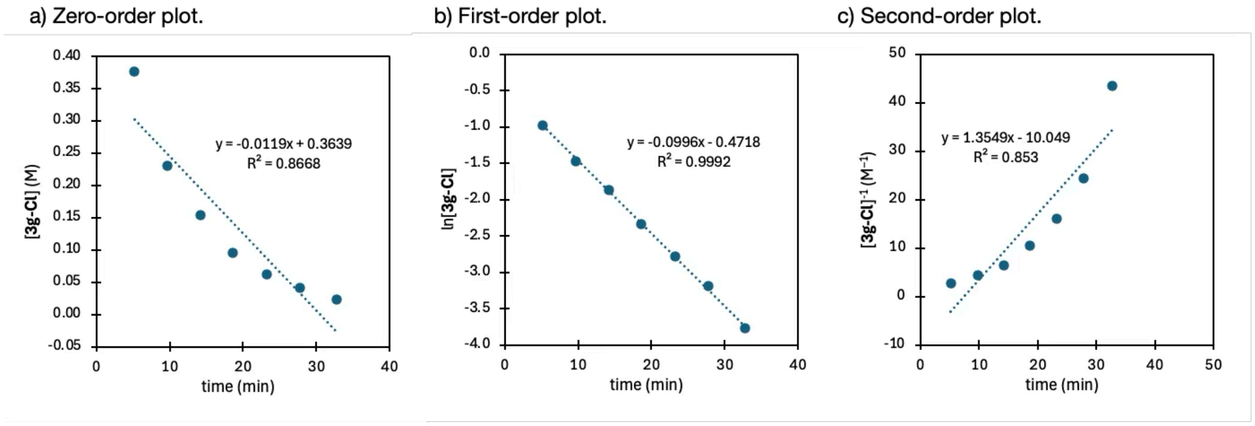

Figure S10. Kinetic plots for reaction of **3g-Cl** at 55 ºC.

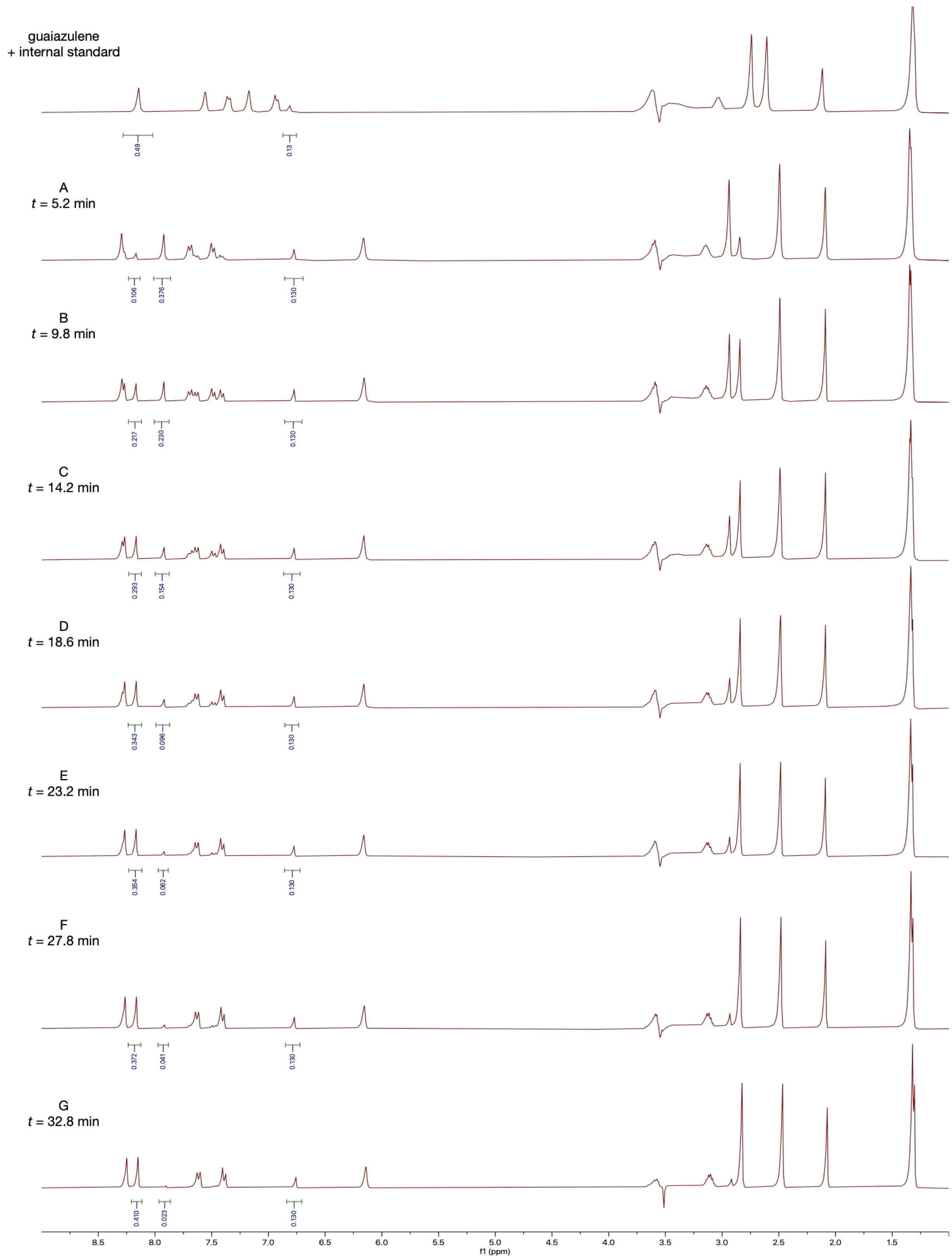

Figure S11. No-D ^{1}H NMR data for reaction of **3g-Cl** at 45 ºC.

Table S5. Rate constants for reaction of **3g-Cl** at various temperatures.

Set temp (°C)	corrected temp (°C)	corrected temp (K)	1/corrected temp (1/K)	k_{obs} (min^{-1})	$t_{1/2}$ (min)
25.0	25.8	299.0	0.003345	0.0055	126.0
35.0	35.4	308.5	0.003241	0.0267	41.5
45.0	44.9	318.1	0.003144	0.0353	19.6
55.0	54.5	327.6	0.003052	0.0996	7.0

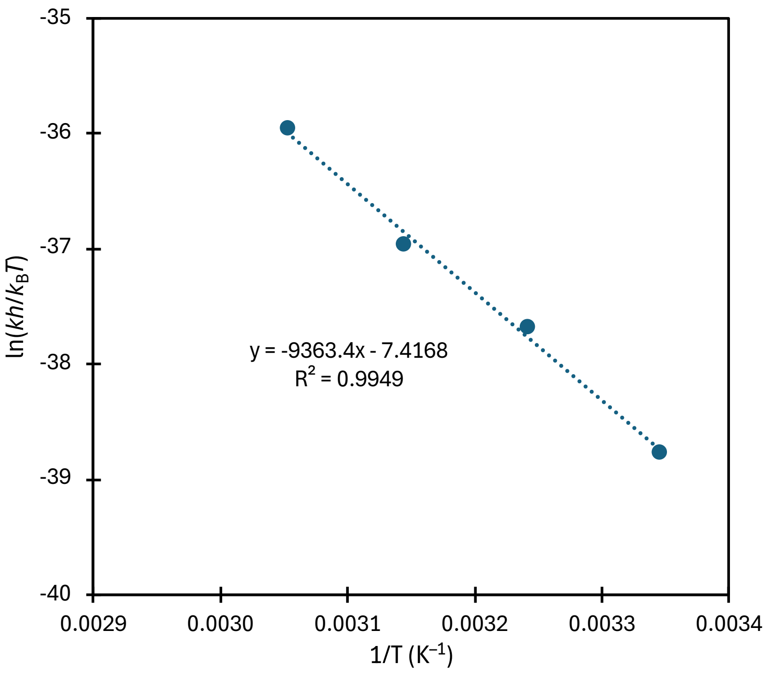

Figure S12. Eyring plot for reaction of **3g-Cl**.

To evaluate the role of halide in the reaction of **3g-Cl**, a kinetic run was set up according to **General Procedure C** with the addition of LiCl (11.7 mg, 0.276 mmol, 1.12 equiv) along with guaiazulene and durene. In this case, there was signal overlap with the durene resonance at δ 6.8 ppm, so the durene signal at δ 2.1 ppm was used as a reference instead.

Table S6. Kinetic data for reaction of **3g-Cl** at 25 ºC with the addition of LiCl.

ID	time (min)	[3g-Cl] (M)	ln[3g-Cl]	$[3g\text{-}Cl]^{-1}$ (M^{-1})	[4g-Cl] (M)
A	5.1	0.464	-0.77	2.2	0.046
B	10.9	0.451	-0.80	2.2	0.044
C	20.4	0.442	-0.82	2.3	0.067
D	29.7	0.420	-0.87	2.4	0.086
E	43.2	0.384	-0.96	2.6	0.114
F	67.0	0.324	-1.13	3.1	0.153
G	101	0.252	-1.38	4.0	0.207
H	136	0.196	-1.63	5.1	0.264
I	166	0.155	-1.86	6.5	0.289
J	189	0.134	-2.01	7.5	0.315
K	296	0.068	-2.69	14.7	0.375
L	396	0.040	-3.22	25.0	0.396

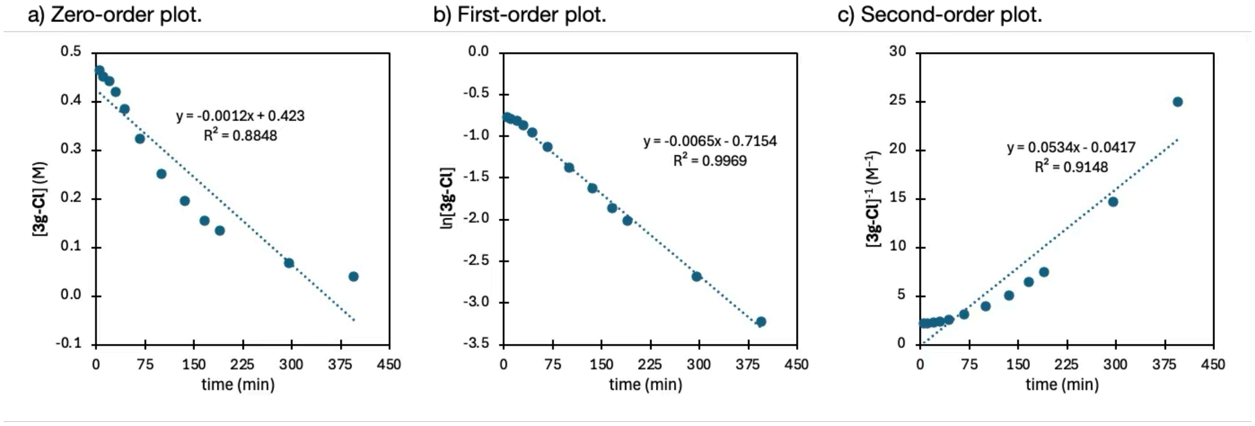

Figure S13. Kinetic plots for reaction of **3g-Cl** at 25 ºC with the addition of LiCl.

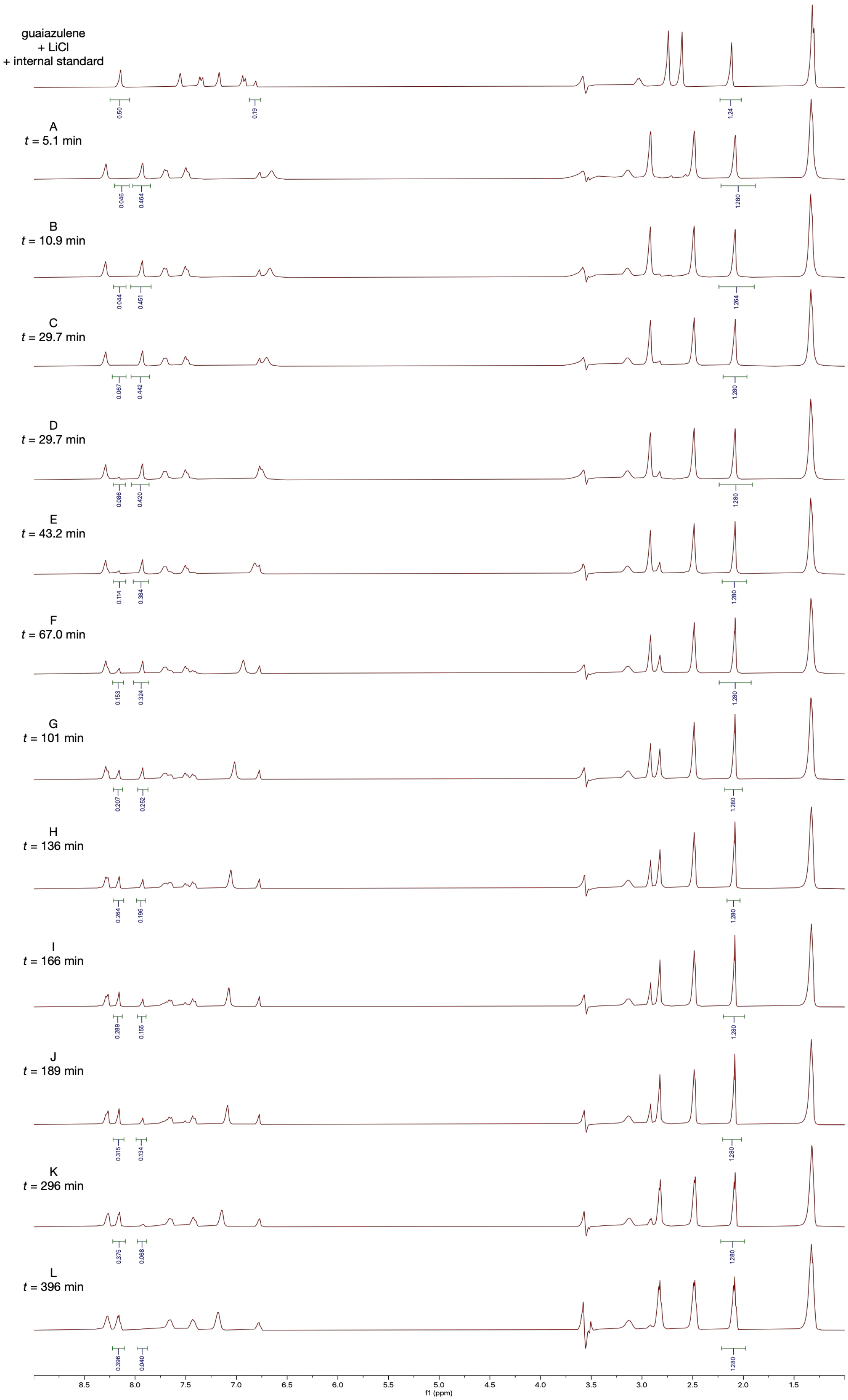

Figure S14. No-D ^{1}H NMR data for reaction of **3g-Cl** at 25 ºC with the addition of LiCl.

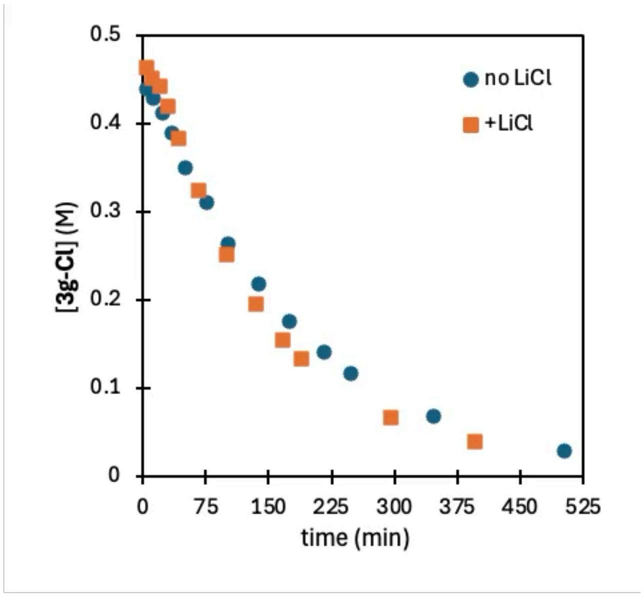

Figure S15. Reaction kinetics for conversion of **3g-Cl** in the absence/presence of exogenous LiCl at 25 ºC. Initial rates were measured as –0.118 M/h (no LiCl) and –0.123 M/h (+ LiCl).

II.e. Kinetics Data for Azulene Reactions with Oxalyl Chloride

Table S7. Kinetic data for reaction of **3a-Cl** at 75 ºC.

ID	time (min)	[3a-Cl] (M)	ln[3a-Cl]	$[3a\text{-}Cl]^{-1}$ (M^{-1})	[4a-Cl] (M)
A	3.9	0.406	-0.90	2.46	0.007
B	10.6	0.440	-0.82	2.27	0.024
C	17.6	0.378	-0.97	2.65	0.040
D	27.2	0.355	-1.04	2.82	0.061
E	43.3	0.327	-1.12	3.06	0.094
F	60.2	0.289	-1.24	3.46	0.125
G	85.1	0.227	-1.48	4.41	0.151
H	111	0.202	-1.60	4.95	0.192
I	131	0.181	-1.71	5.52	0.219
J	175	0.138	-1.98	7.25	0.260
K	227	0.102	-2.28	9.80	0.303
L	266	0.082	-2.50	12.20	0.333
M	312	0.061	-2.80	16.39	0.346

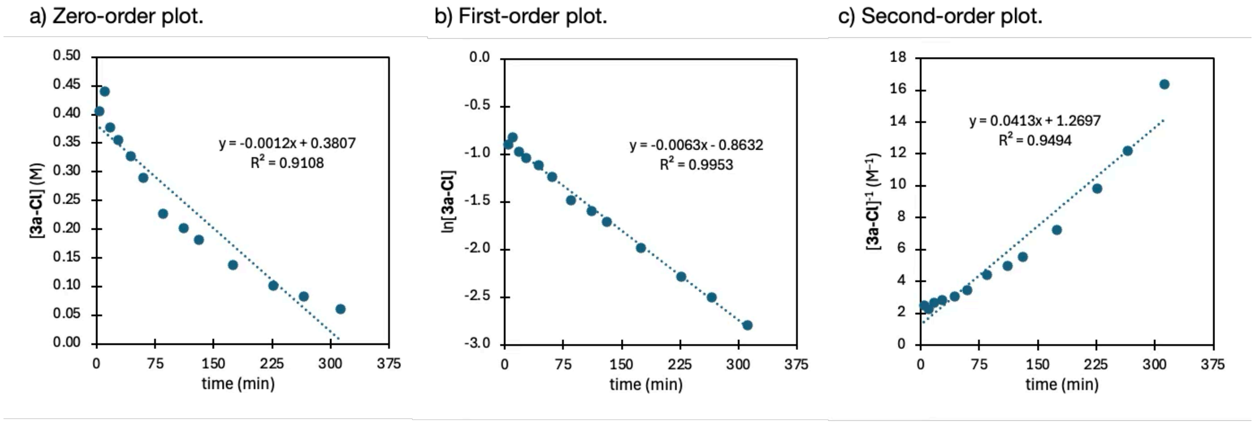

Figure S16. Kinetic plots for reaction of **3a-Cl** at 75 ºC.

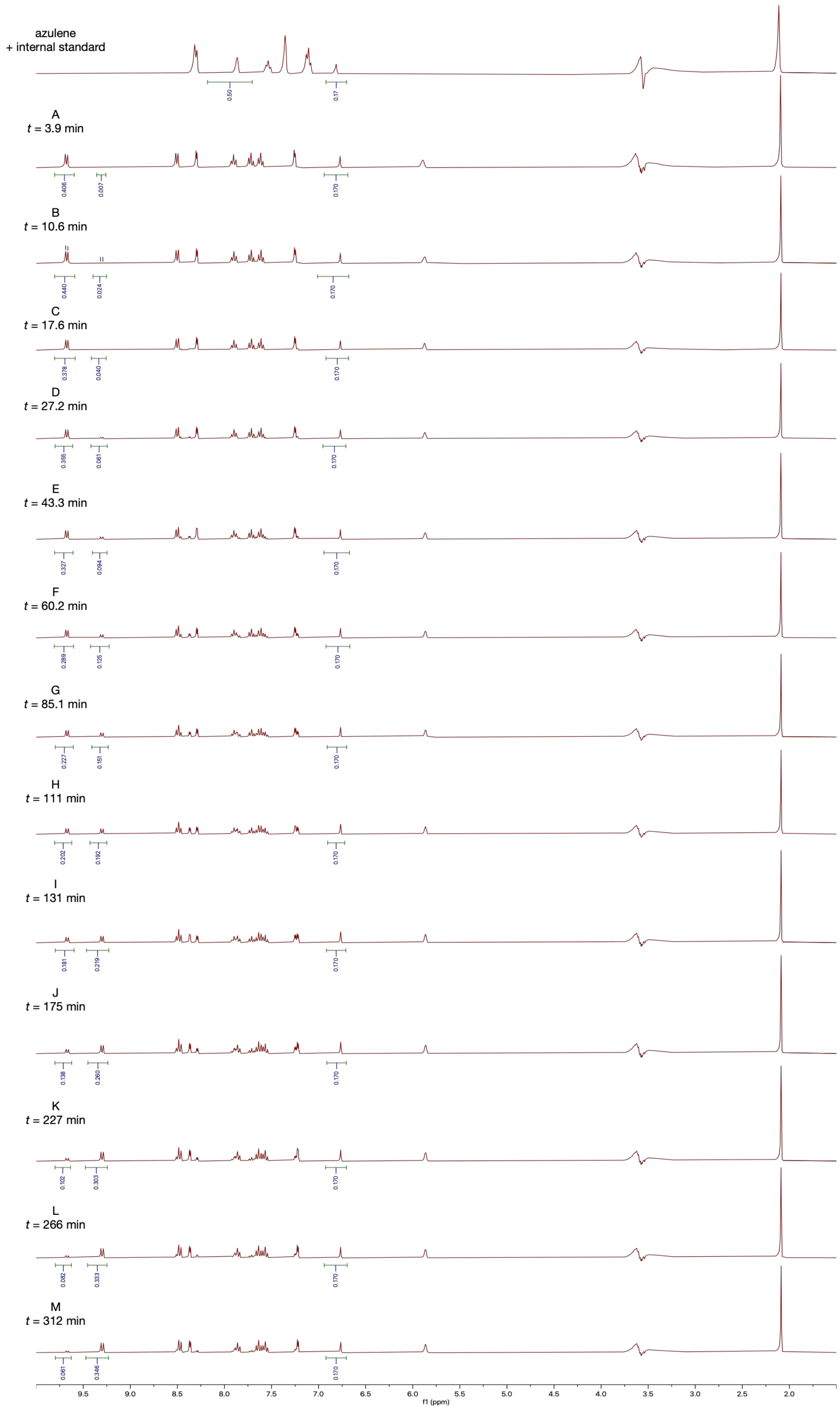

Figure S17. No-D ^{1}H NMR data for reaction of **3a-Cl** at 75 ºC.

Table S8. Kinetic data for reaction of **3a-Cl** at 80 ºC.

ID	time (min)	[3a-Cl] (M)	ln[3a-Cl]	$[3a\text{-}Cl]^{-1}$ (M^{-1})	[4a-Cl] (M)
A	4.2	0.581	-0.54	1.72	0.020
B	10.4	0.566	-0.57	1.77	0.054
C	20.9	0.538	-0.62	1.86	0.117
D	31.7	0.493	-0.71	2.03	0.181
E	46.8	0.425	-0.86	2.35	0.248
F	61.0	0.340	-1.08	2.94	0.288
G	84.5	0.266	-1.32	3.76	0.360
H	112	0.206	-1.58	4.85	0.421
I	140	0.169	-1.78	5.92	0.482
J	172	0.126	-2.07	7.94	0.511
K	209	0.090	-2.41	11.11	0.539
L	250	0.061	-2.80	16.39	0.549
M	301	0.043	-3.15	23.26	0.593

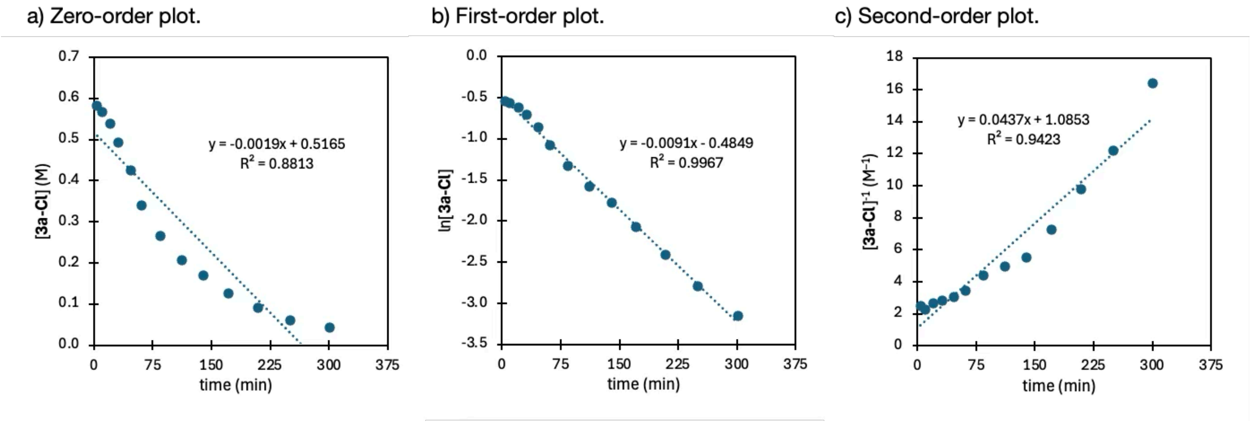

Figure S18. Kinetic plots for reaction of **3a-Cl** at 80 ºC.

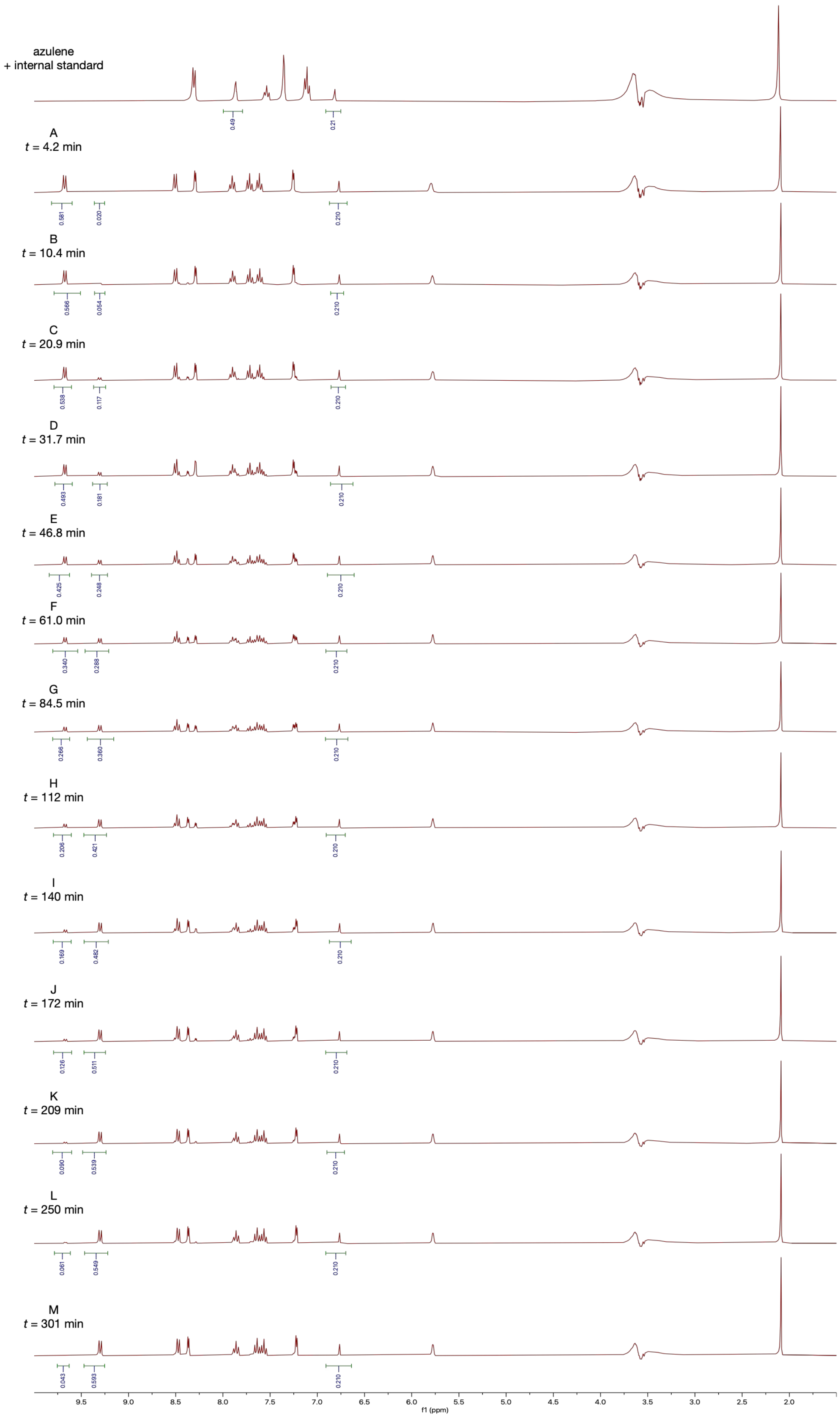

Figure S19. No-D ^{1}H NMR data for reaction of **3a-Cl** at 80 ºC.

Table S9. Kinetic data for reaction of **3a-Cl** at 85 ºC.

ID	time (min)	[3a-Cl] (M)	ln[3a-Cl]	$[3a\text{-}Cl]^{-1}$ (M^{-1})	[4a-Cl] (M)
A	3.7	0.565	-0.57	1.77	0.025
B	8.3	0.573	-0.56	1.75	0.067
C	12.9	0.522	-0.65	1.92	0.103
D	17.5	0.500	-0.69	2.00	0.141
E	22.0	0.453	-0.79	2.21	0.167
F	26.6	0.427	-0.85	2.34	0.199
G	31.1	0.378	-0.97	2.65	0.215
H	36.4	0.355	-1.04	2.82	0.249
I	46.6	0.302	-1.20	3.31	0.294
J	58.8	0.250	-1.39	4.00	0.340
K	70.6	0.208	-1.57	4.81	0.377
L	87.1	0.170	-1.77	5.88	0.429
M	119	0.102	-2.28	9.80	0.478
N	159	0.060	-2.81	16.67	0.505
O	220	0.025	-3.69	40.00	0.532

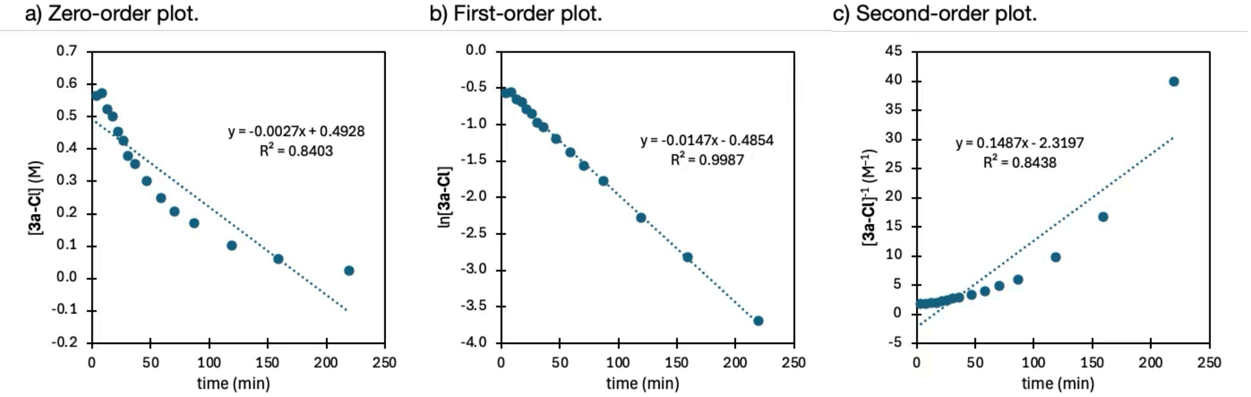

Figure S20. Kinetic plots for reaction of **3a-Cl** at 85 ºC.

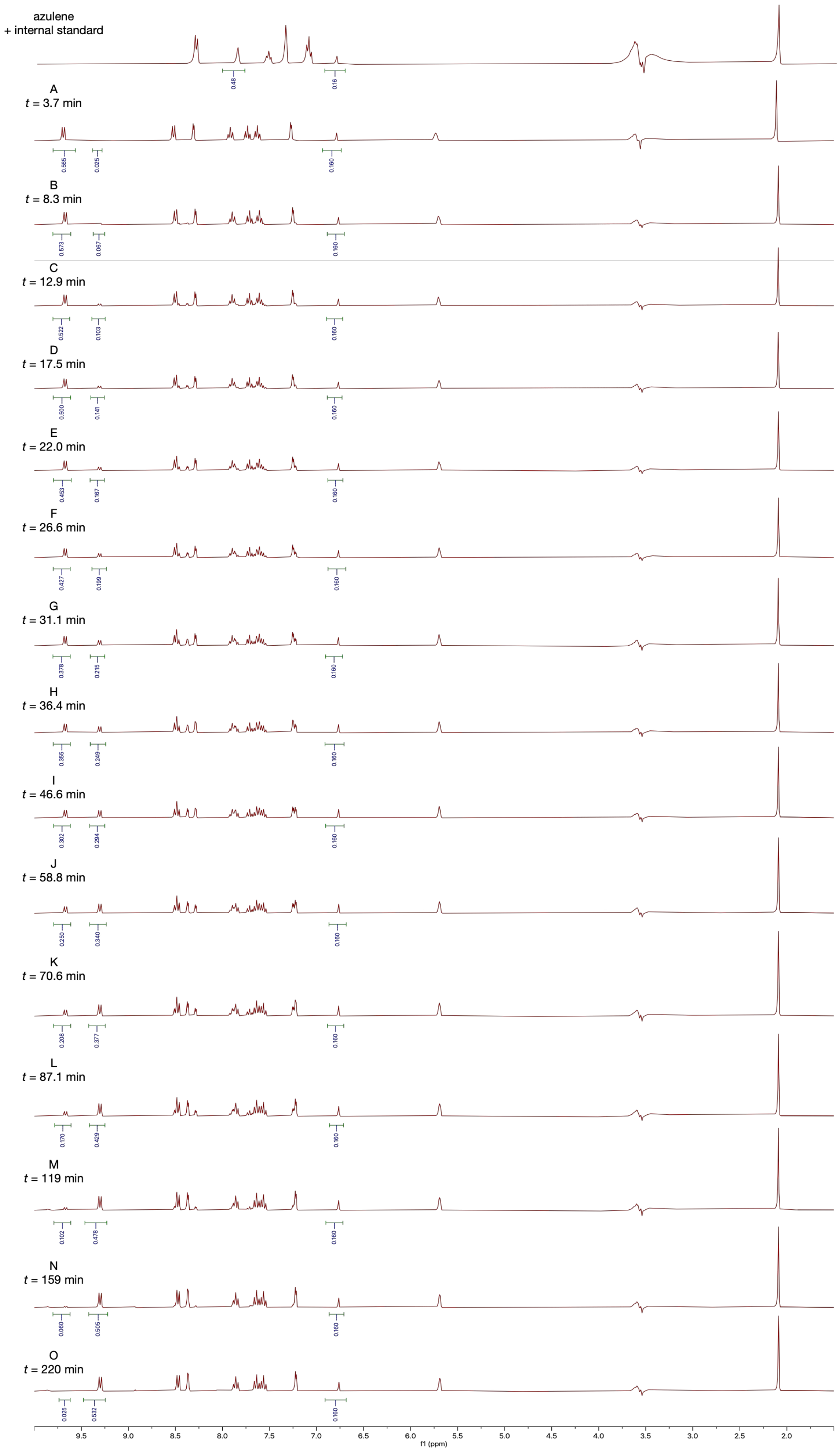

Figure S21. No-D ^{1}H NMR data for reaction of **3a-Cl** at 85 ºC.

Table S10. Kinetic data for reaction of **3a-Cl** at 90 ºC.

ID	time (min)	[3a-Cl] (M)	ln[3a-Cl]	$[3a\text{-}Cl]^{-1}$ (M^{-1})	[4a-Cl] (M)
A	3.9	0.438	-0.83	2.28	0.023
B	8.4	0.425	-0.86	2.35	0.079
C	12.9	0.378	-0.97	2.65	0.115
D	17.4	0.339	-1.08	2.95	0.149
E	21.8	0.316	-1.15	3.16	0.191
F	26.2	0.281	-1.27	3.56	0.211
G	33.4	0.246	-1.40	4.07	0.262
H	45.0	0.195	-1.63	5.13	0.314
I	61.9	0.132	-2.02	7.58	0.340
J	80.3	0.093	-2.38	10.75	0.392
K	105	0.051	-2.98	19.61	0.428

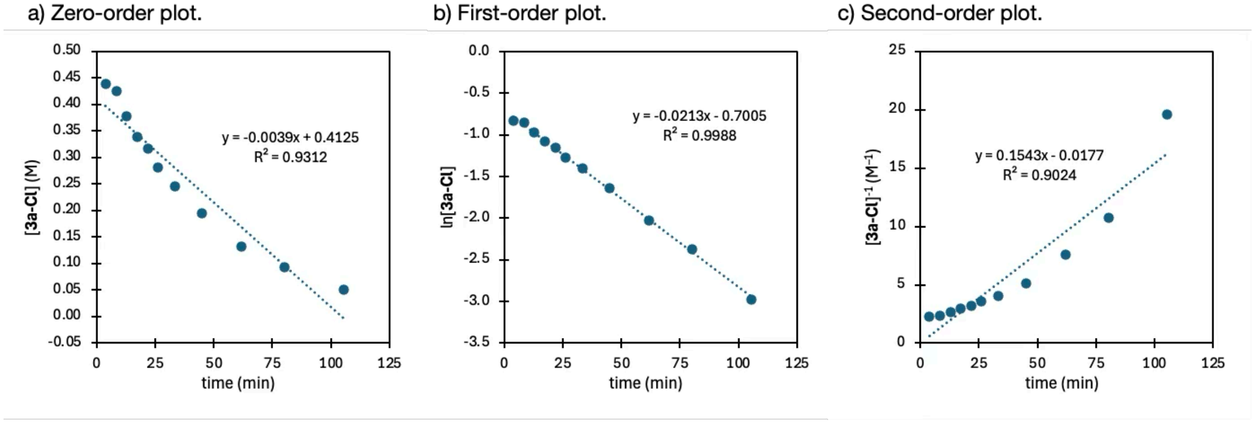

Figure S22. Kinetic plots for reaction of **3a-Cl** at 90 ºC.

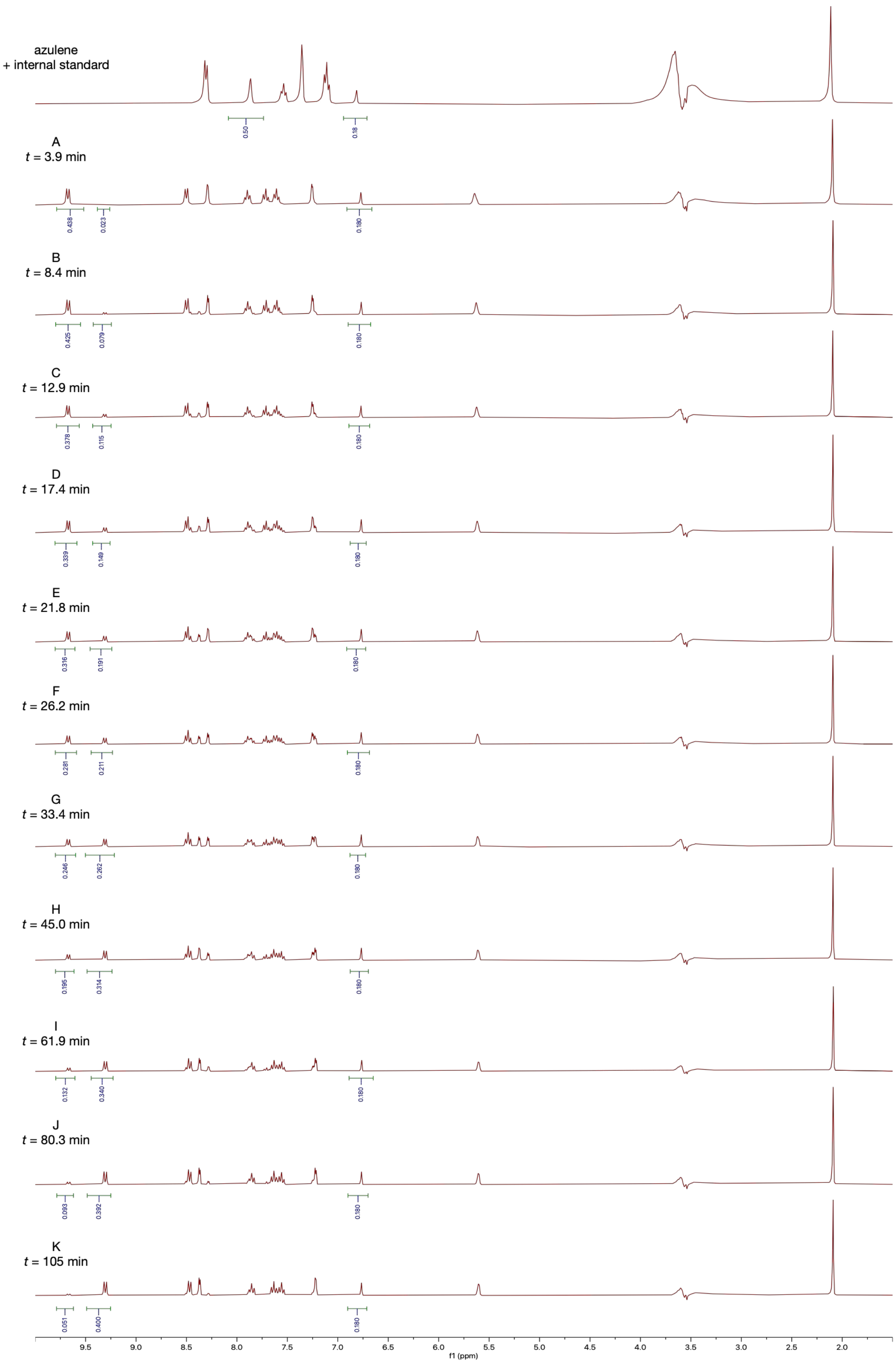

Figure S23. No-D ^{1}H NMR data for reaction of **3a-Cl** at 90 ºC.

Table S11. Rate constants for reaction of **3a-Cl** at various temperatures.

Set temp (°C)	corrected temp (°C)	corrected temp (K)	1/corrected temp (1/K)	k_{obs} (min^{-1})	$t_{1/2}$ (min)
75.0	73.6	346.8	0.002884	0.0063	110.0
80.0	78.4	351.5	0.002845	0.0091	76.2
85.0	83.2	356.3	0.002807	0.0147	47.2
90.0	87.9	361.1	0.002769	0.0213	32.5

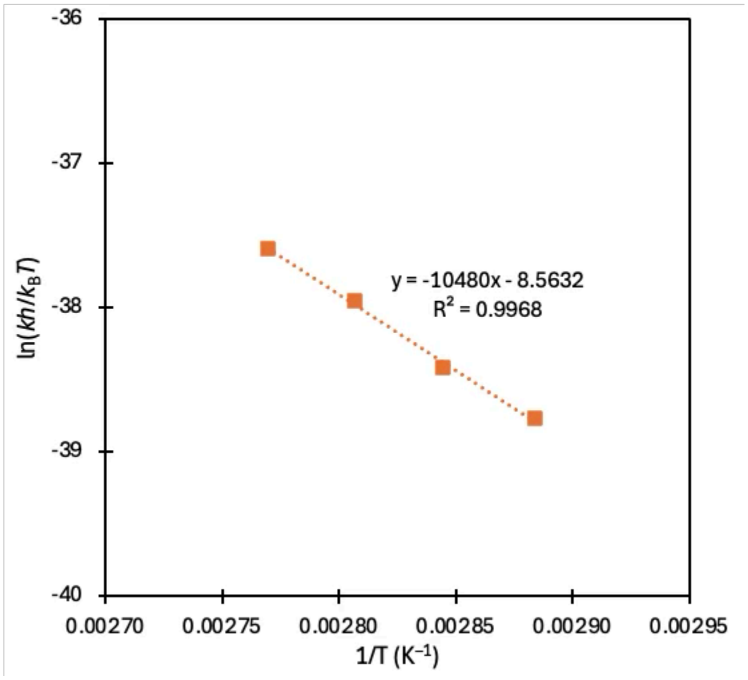

Figure S24. Eyring plot for reaction of **3a-Cl**.

II.f. Kinetics Data for Azulene Reactions with Oxalyl Bromide

Table S12. Kinetic data for reaction of **3a-Br** at 20 ºC.

ID	time (min)	[3a-Br] (M)	ln[3a-Br]	$[3a\text{-}Br]^{-1}$ (M^{-1})	[4a-Br] (M)
A	4.8	0.474	-0.75	2.11	0.047
B	9.3	0.458	-0.78	2.18	0.080
C	13.8	0.418	-0.87	2.39	0.104
D	18.5	0.394	-0.93	2.54	0.130
E	27.3	0.342	-1.07	2.92	0.174
F	37.3	0.289	-1.24	3.46	0.223
G	51.5	0.233	-1.46	4.29	0.278
H	62.6	0.203	-1.59	4.93	0.323
I	77.1	0.154	-1.87	6.49	0.362
J	91.3	0.125	-2.08	8.00	0.380
K	109	0.091	-2.40	10.99	0.396
L	125	0.072	-2.63	13.89	0.404
M	147	0.049	-3.02	20.41	0.426
N	165	0.034	-3.38	29.41	0.413

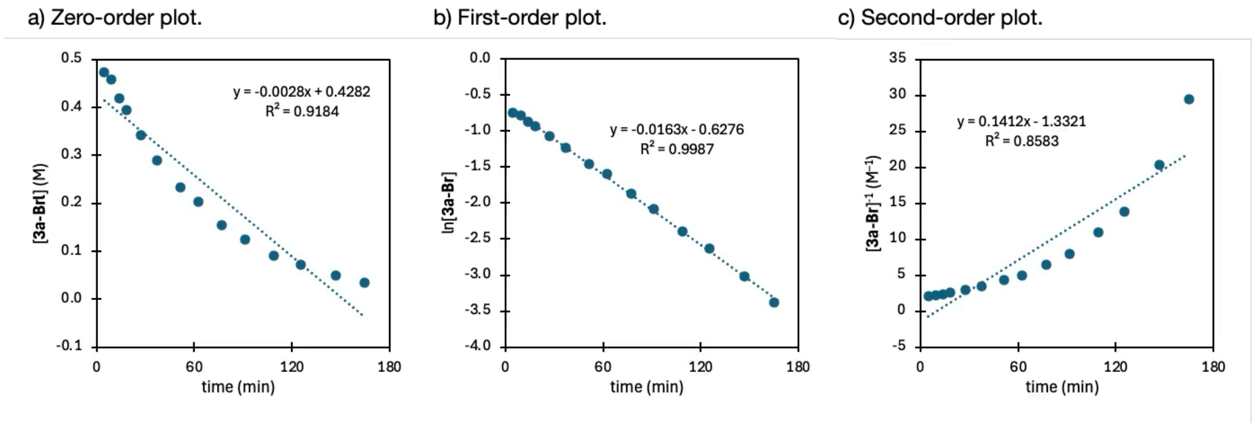

Figure S25. Kinetic plots for reaction of **3a-Br** at 20 ºC.

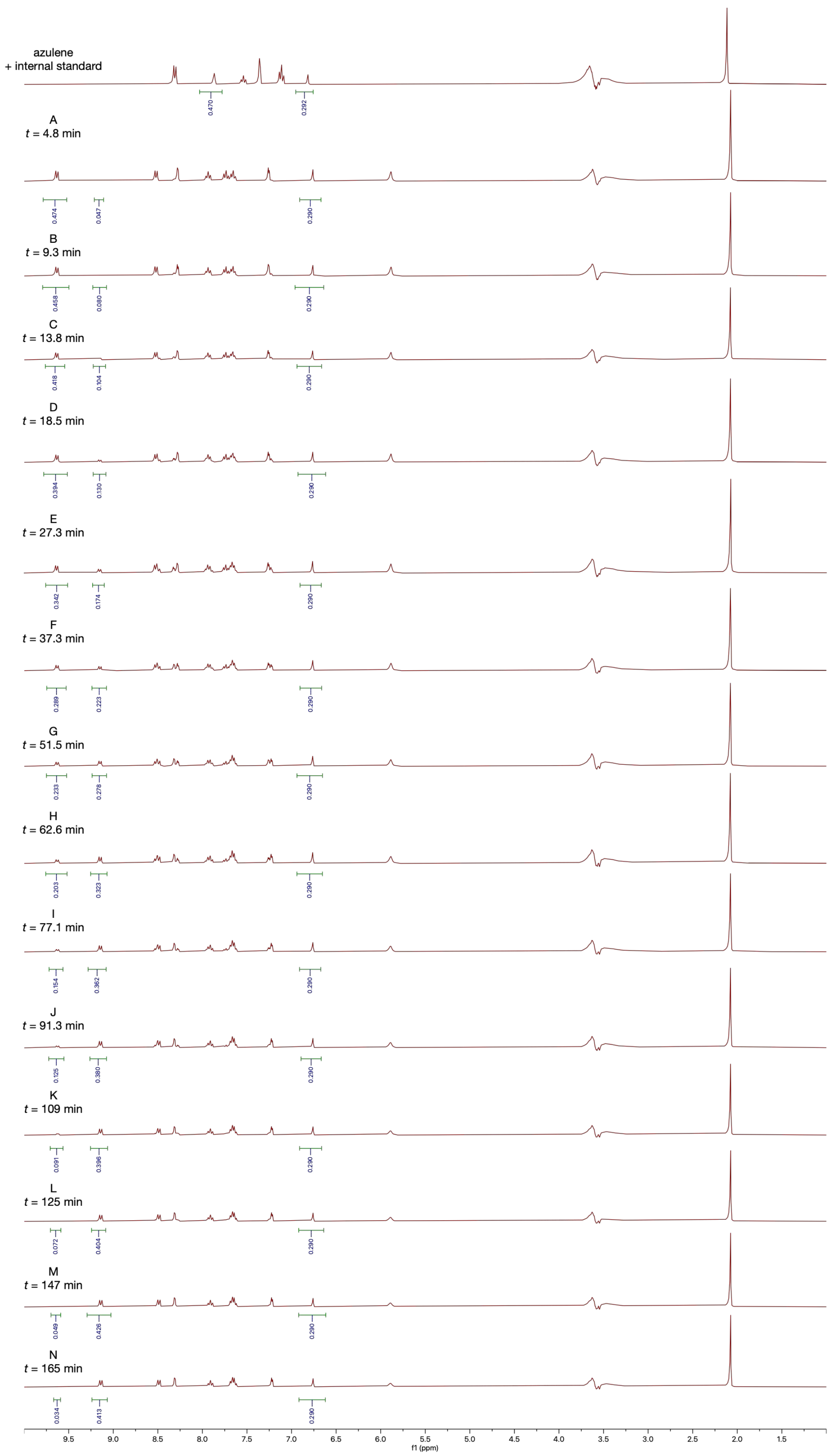

Figure S26. No-D ^{1}H NMR data for reaction of **3a-Br** at 20 ºC.

Table S13. Kinetic data for reaction of **3a-Br** at 25 ºC.

ID	time (min)	[3a-Br] (M)	ln[3a-Br]	$[3a\text{-}Br]^{-1}$ (M^{-1})	[4a-Br] (M)
A	5.3	0.595	-0.52	1.68	0.089
B	9.7	0.528	-0.64	1.89	0.146
C	14.1	0.465	-0.77	2.15	0.203
D	18.5	0.417	-0.87	2.40	0.261
E	22.9	0.364	-1.01	2.75	0.297
F	27.4	0.326	-1.12	3.07	0.344
G	36.7	0.258	-1.35	3.88	0.406
H	45.3	0.213	-1.55	4.69	0.464
I	55.3	0.163	-1.81	6.13	0.495
J	69.4	0.118	-2.14	8.47	0.539
K	82.9	0.074	-2.60	13.51	0.545
L	97.3	0.056	-2.88	17.86	0.560

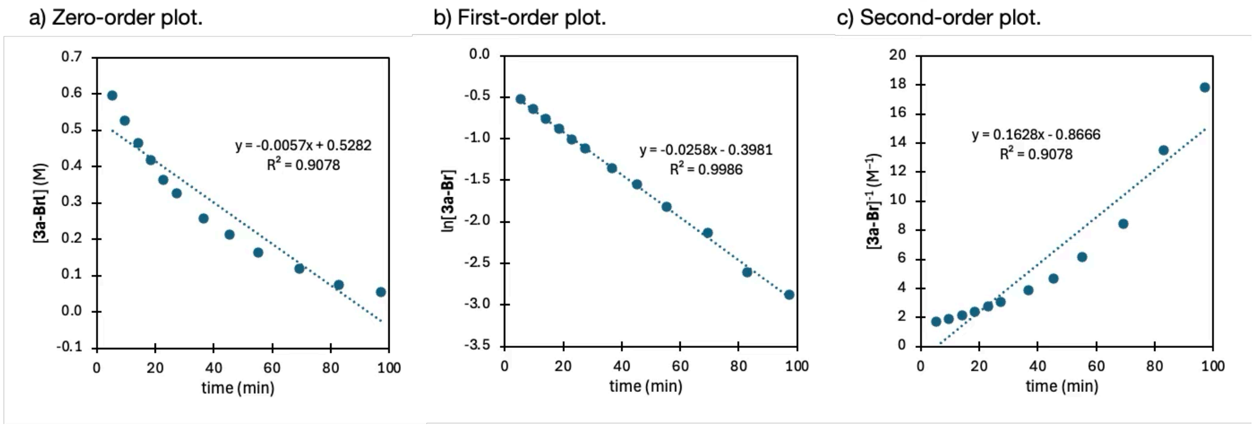

Figure S27. Kinetic plots for reaction of **3a-Br** at 25 ºC.

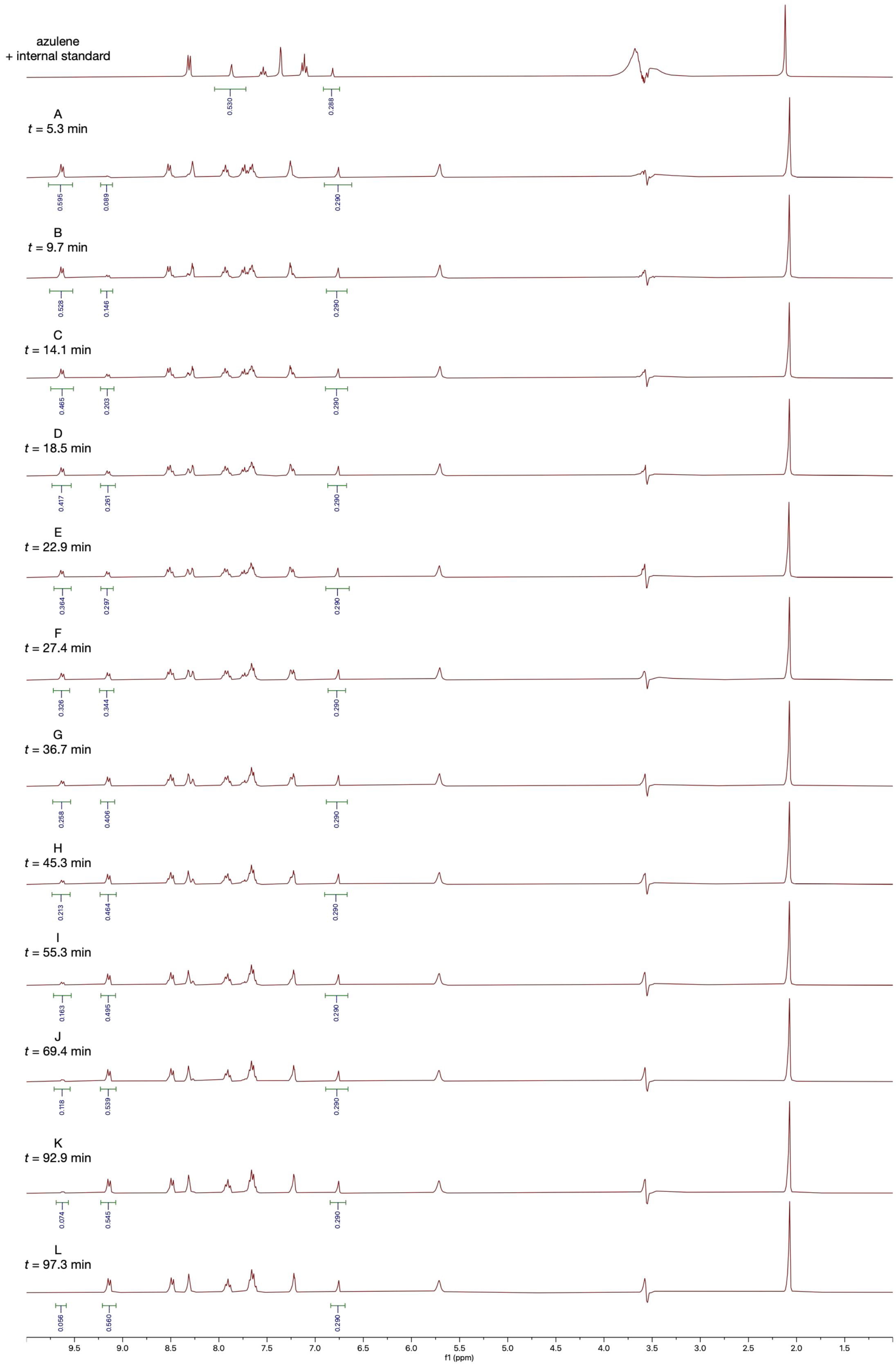

Figure S28. No-D ^{1}H NMR data for reaction of **3a-Br** at 25 ºC.

Table S14. Kinetic data for reaction of **3a-Br** at 30 ºC.

ID	time (min)	[3a-Br] (M)	ln[3a-Br]	$[3a\text{-}Br]^{-1}$ (M^{-1})	[4a-Br] (M)
A	4.9	0.477	-0.74	2.10	0.082
B	9.5	0.417	-0.87	2.40	0.147
C	14.1	0.353	-1.04	2.83	0.207
D	18.7	0.292	-1.23	3.42	0.249
E	23.2	0.258	-1.35	3.88	0.307
F	29.4	0.195	-1.63	5.13	0.339
G	35.8	0.158	-1.85	6.33	0.383
H	43.7	0.116	-2.15	8.62	0.418
I	51.5	0.080	-2.53	12.50	0.451
J	58.6	0.067	-2.70	14.93	0.455
K	69.2	0.048	-3.04	20.83	0.481
L	78.7	0.037	-3.30	27.03	0.481

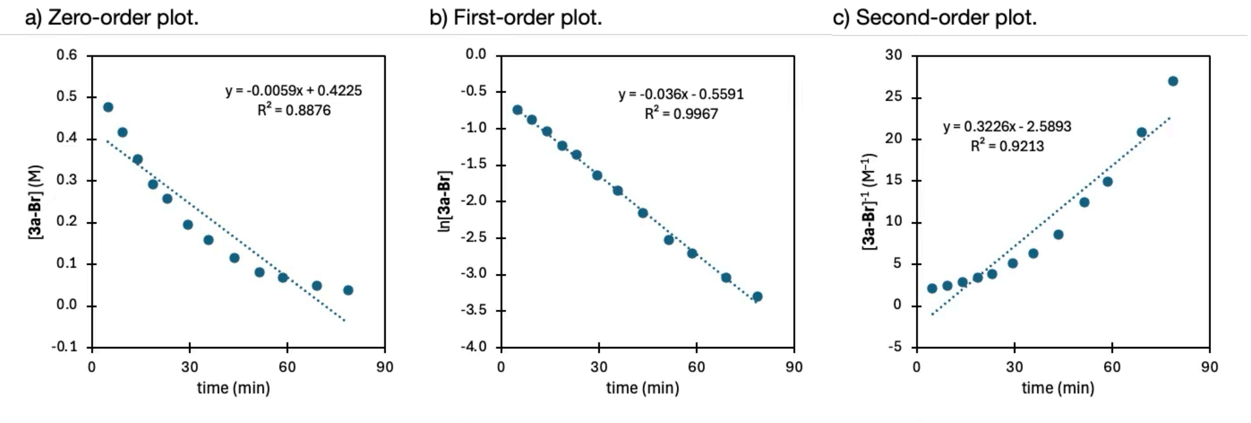

Figure S29. Kinetic plots for reaction of **3a-Br** at 30 ºC.

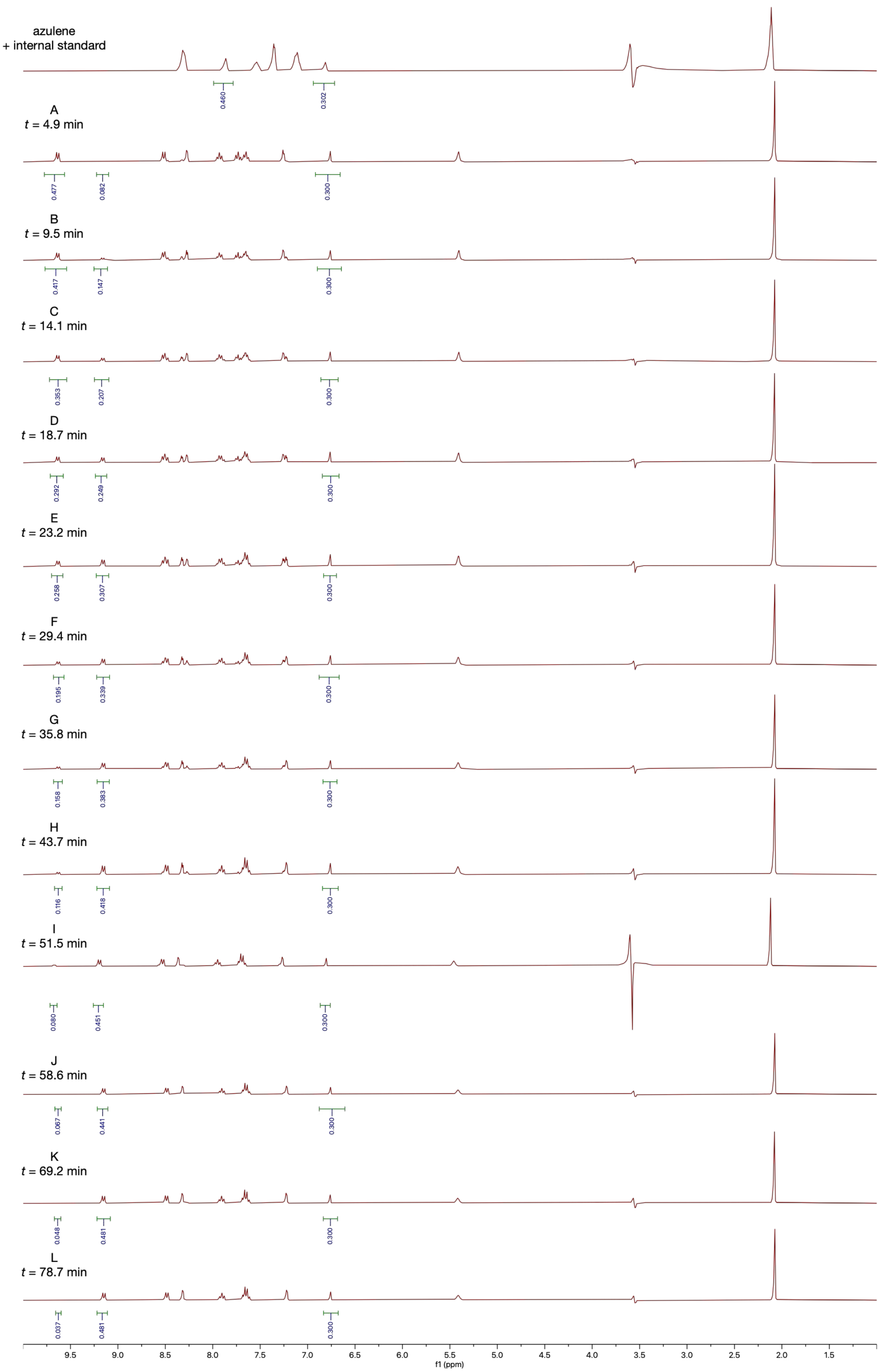

Figure S30. No-D ^{1}H NMR data for reaction of **3a-Br** at 30 ºC.

Table S15. Kinetic data for reaction of **3a-Br** at 35 ºC.

ID	time (min)	[3a-Br] (M)	ln[3a-Br]	$[3a\text{-}Br]^{-1}$ (M^{-1})	[4a-Br] (M)
A	4.6	0.376	-0.98	2.66	0.107
B	9.0	0.317	-1.15	3.15	0.260
C	13.4	0.211	-1.56	4.74	0.333
D	17.7	0.143	-1.94	6.99	0.363
E	22.1	0.107	-2.23	9.35	0.411
F	26.4	0.079	-2.54	12.66	0.440
G	30.7	0.057	-2.86	17.54	0.453
H	35.1	0.037	-3.30	27.03	0.443
I	39.4	0.029	-3.54	34.48	0.452

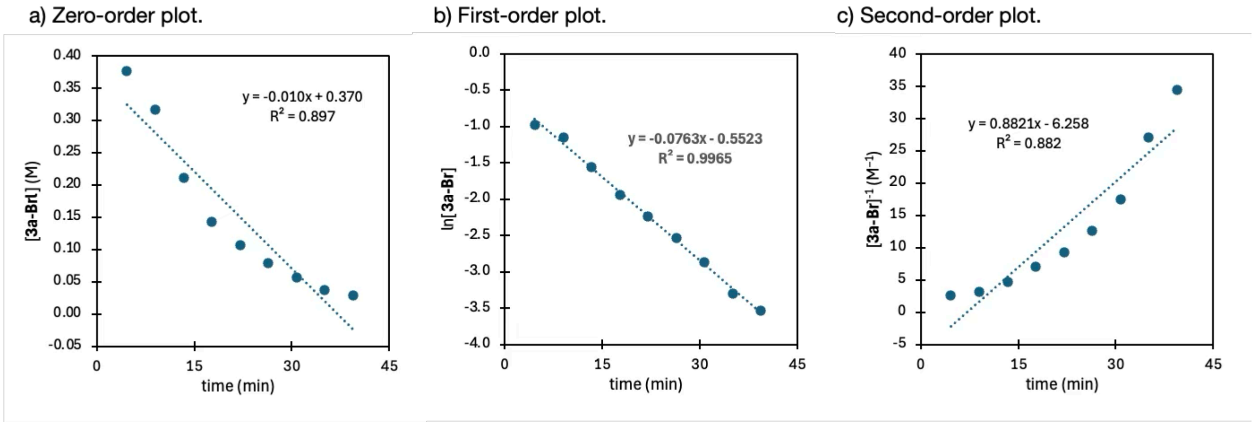

Figure S31. Kinetic plots for reaction of **3a-Br** at 35 ºC.

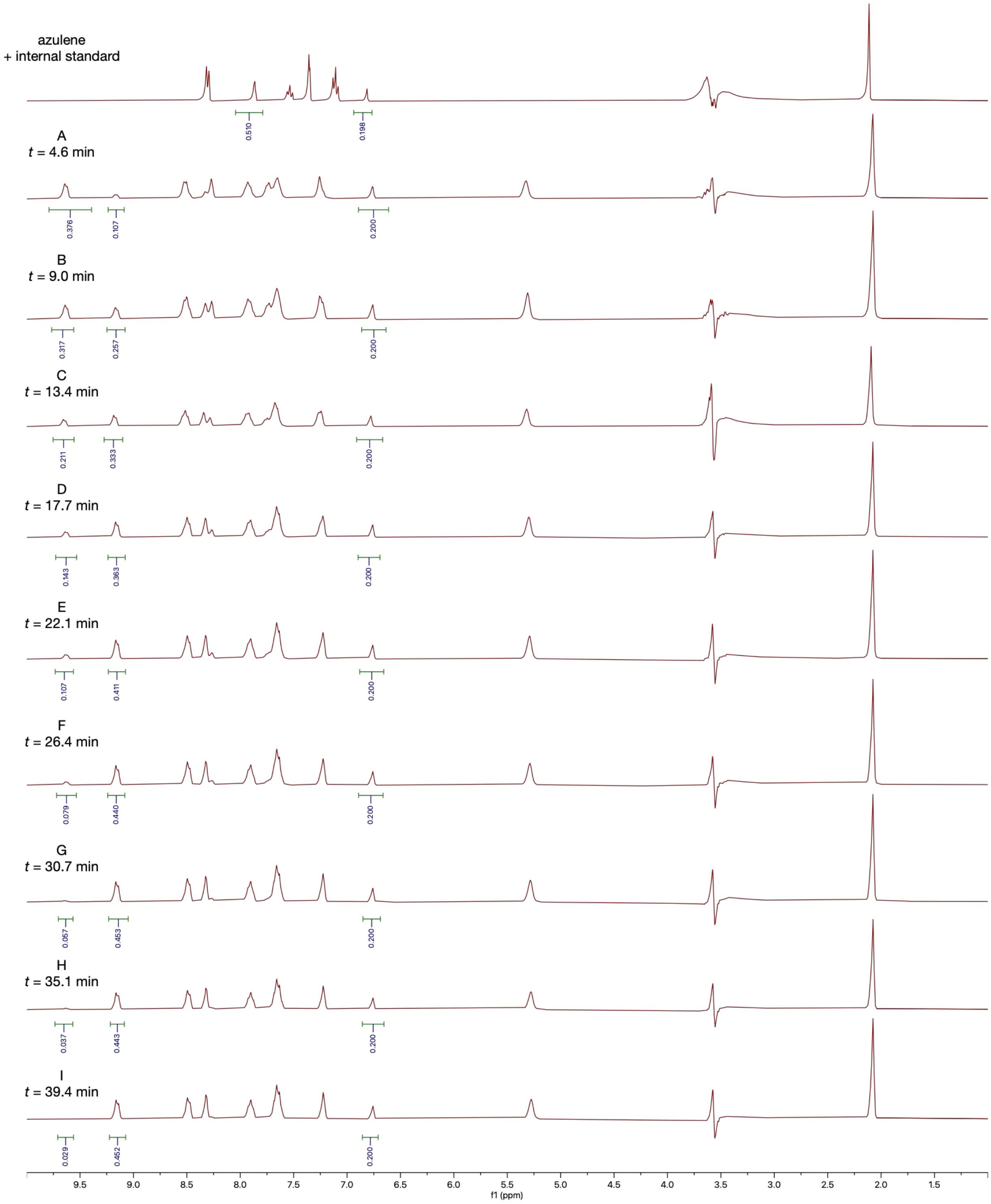

Figure S32. No-D ^{1}H NMR data for reaction of **3a-Br** at 35 ºC.

Table S16. Rate constants for reaction of **3a-Br** at various temperatures.

set temp (°C)	corrected temp (°C)	corrected temp (K)	1/corrected temp (1/K)	k_{obs} (min^{-1})	$t_{1/2}$ (min)
20.0	21.1	294.2	0.003399	0.0163	42.5
25.0	25.8	299.0	0.003345	0.0258	26.9
30.0	30.6	303.8	0.003292	0.0360	19.3
35.0	35.4	308.5	0.003241	0.0763	9.1

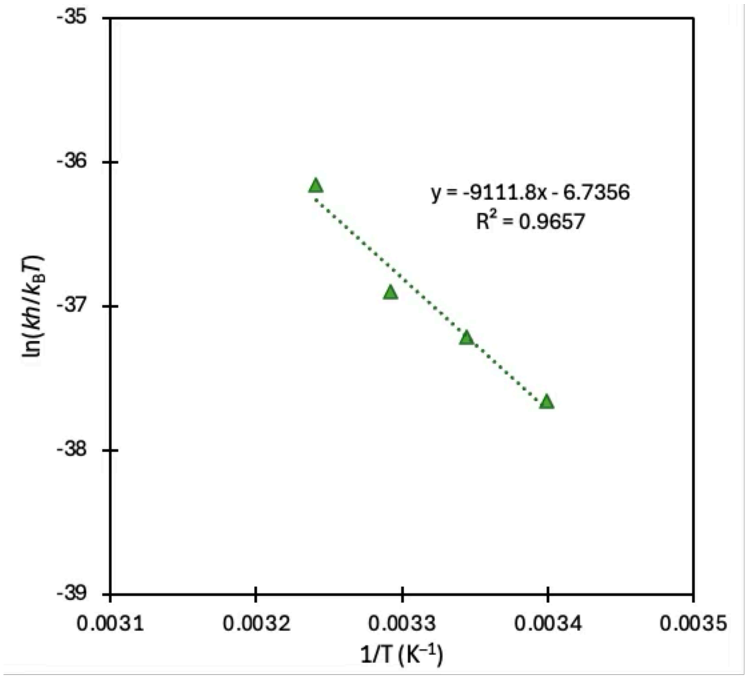

Figure S33. Eyring plot for reaction of **3a-Br**.

To evaluate the role of halide in the reaction of **3a-Br**, a kinetic run was set up according to **General Procedure E** with the addition of LiBr (23.9 mg, 0.275 mmol, 1.10 equiv) along with azulene and durene.

Table S17. Kinetic data for reaction of **3a-Br** at 25 ºC with the addition of LiBr.

ID	time (min)	[3a-Br] (M)	ln[3a-Br]	$[3a\text{-}Br]^{-1}$ (M^{-1})	[4a-Br] (M)
A	6.3	0.551	-0.60	1.81	0.097
B	10.8	0.453	-0.79	2.21	0.153
C	15.2	0.402	-0.91	2.49	0.213
D	19.6	0.347	-1.06	2.88	0.241
E	26.4	0.279	-1.28	3.58	0.331
F	32.3	0.229	-1.47	4.37	0.369
G	41.2	0.176	-1.74	5.68	0.411
H	50.4	0.138	-1.98	7.25	0.446
I	58.3	0.102	-2.28	9.80	0.459
J	68.7	0.080	-2.53	12.50	0.463
K	78.3	0.062	-2.78	16.13	0.469
L	89.3	0.042	-3.17	23.81	0.477

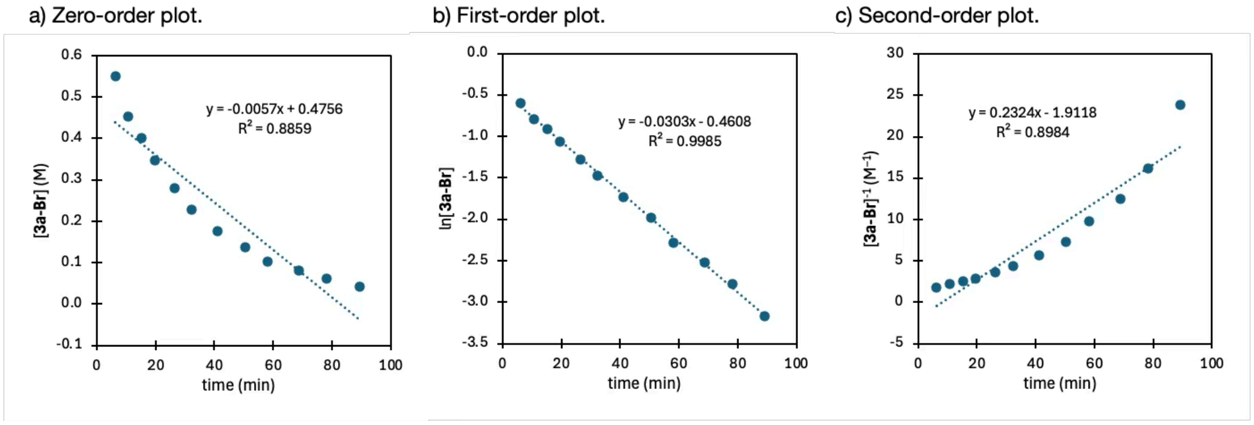

Figure S34. Kinetic plots for reaction of **3a-Br** at 25 ºC with the addition of LiBr.

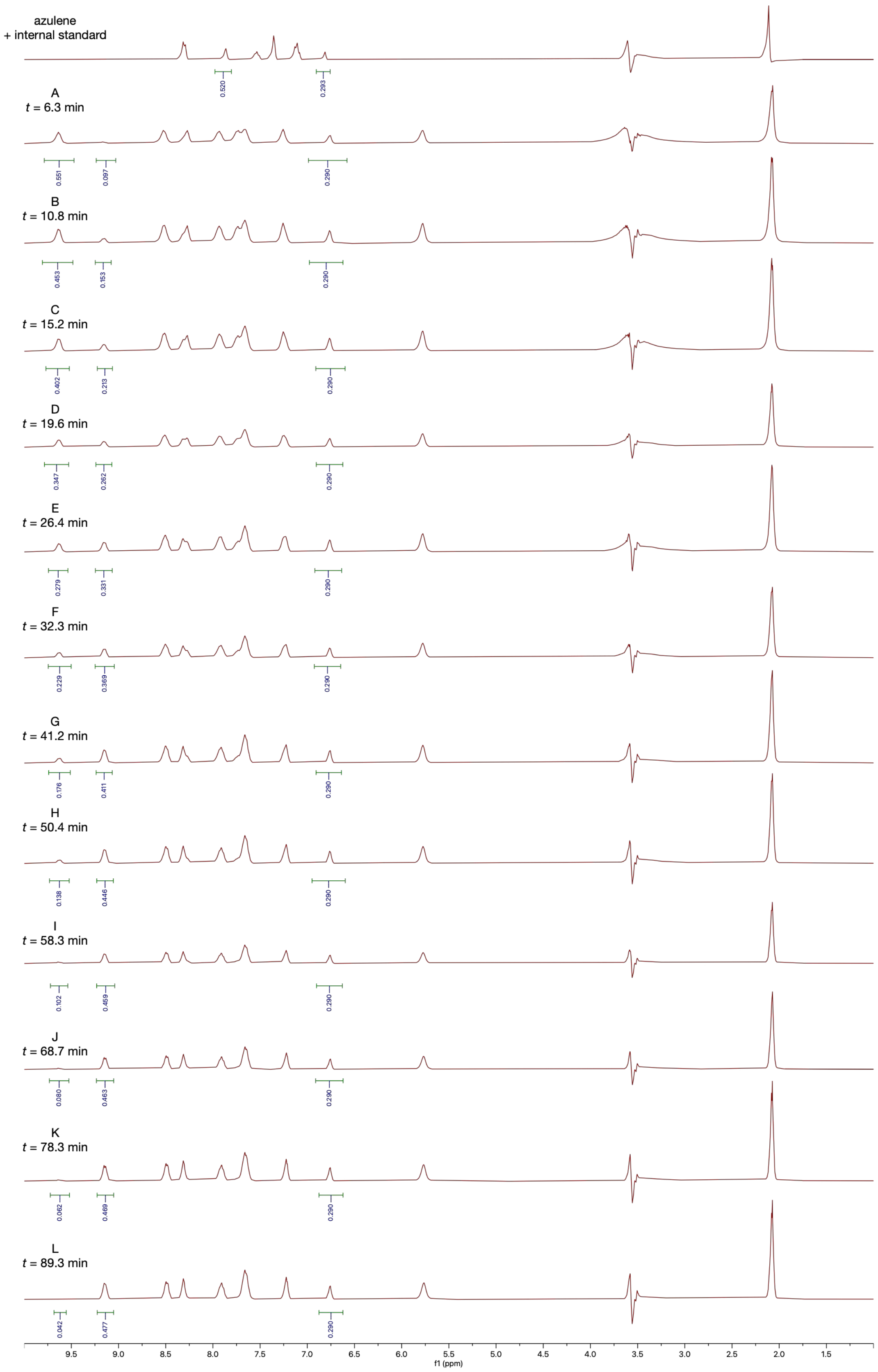

Figure S35. No-D ^{1}H NMR data for reaction of **3a-Br** at 25 ºC with the addition of LiBr.

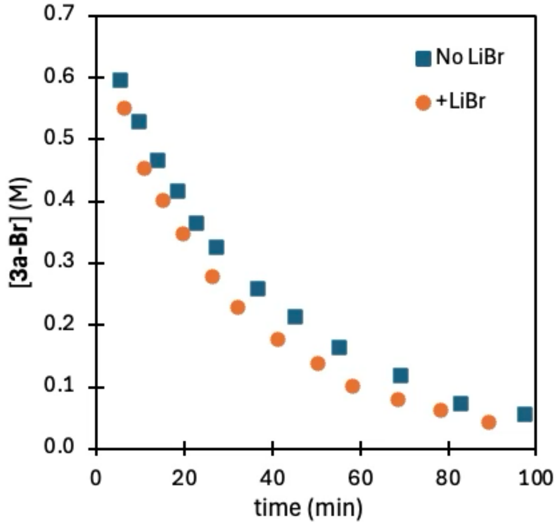

Figure S36. Reaction kinetics for conversion of **3a-Br** in the absence/presence of exogenous LiCl at 25 ºC. Initial rates were measured as –089 M/h (no LiBr) and –0.82 M/h (+ LiBr).

II.g. NMR Data for Acyl Halides

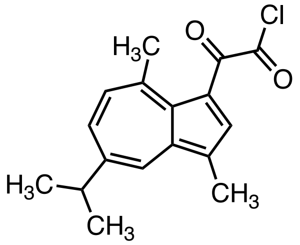

3g-Cl

2-(5-Isopropyl-3,8-dimethylazulen-1-yl)-2-oxoacetyl chloride (**3g-Cl**). A screw-cap NMR tube was charged with guaiazulene **2** (49.0 mg, 0.247 mmol, 1.00 equiv) and dioxane (0.5 mL), then inverted several times to dissolve the solid. Oxalyl chloride (22.0 µL, 0.260 mmol, 1.05 equiv) was added *via* syringe and the tube was again inverted several times to mix. The tube was inserted into the NMR and no-D NMR spectra of **3g-Cl** were acquired at rt.

No-D ^{1}H NMR (400 MHz, dioxane): δ 8.29 (s, 1H), 7.93 (s, 1H), 7.71 (d, *J* = 11.0 Hz, 1H), 7.50 (d, *J* = 11.2 Hz, 1H), 3.18 – 3.09 (m, 1H), 2.92 (s, 3H), 2.49 (s, 3H), 1.33 (d, *J* = 5.3 Hz, 6H) ppm.

No-D ^{13}C{^{1}H} NMR (101 MHz, dioxane): δ 173.7, 167.7, 150.8, 148.6, 144.5, 142.2, 140.5, 137.4, 134.8, 126.1, 116.4, 37.7, 27.9, 23.6, 11.7 ppm. Note that the peak at δ 134.8 ppm represents two overlapping carbon signals, as determined by HMQC.

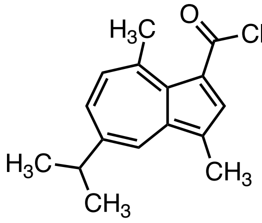

4g-Cl

5-Isopropyl-3,8-dimethylazulene-1-carbonyl chloride (**4g-Cl**). The NMR tube containing **4g-Cl** from above was heated to 70 ºC in the NMR probe for 30 min, then cooled to rt before acquiring no-D spectra of **4g-Cl**.

No-D ^{1}H NMR (400 MHz, dioxane): δ 8.26 (s, 1H), 8.15 (s, 1H), 7.65 (d, *J* = 10.2 Hz, 1H), 7.42 (d, *J* = 10.3 Hz, 1H), 3.17 – 3.06 (m, 1H), 2.81 (s, 3H), 2.47 (s, 3H), 1.32 (d, *J* = 6.9 Hz, 6H) ppm.

No-D ^{13}C{^{1}H} NMR (101 MHz, dioxane): δ 159.9, 149.1, 147.4, 144.4, 143.7, 138.5, 136.8, 134.9, 134.0, 124.8, 117.5, 37.6, 27.4, 23.6, 11.7 ppm.

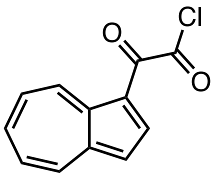

3a-Cl

2-(Azulen-1-yl)-2-oxoacetyl chloride (**3a-Cl**). A screw-cap NMR tube was charged with azulene **1** (32.0 mg, 0.247 mmol, 1.00 equiv), dioxane (0.5 mL), then inverted several times to dissolve the solid. Oxalyl chloride (22.0 µL, 0.260 mmol, 1.05 equiv) was added *via* syringe and the tube was again inverted several times to mix. The tube was inserted into the NMR and heated at 25 ºC for 1 h before acquiring no-D NMR spectra of **3a-Cl** at 25 ºC.

No-D ^{1}H NMR (400 MHz, dioxane): δ 9.67 (d, *J* = 10.3 Hz, 1H), 8.52 (d, *J* = 9.9 Hz, 1H), 8.38 – 8.29 (m, 1H), 7.93 (t, *J* = 9.7 Hz, 1H), 7.73 (t, *J* = 9.8 Hz, 1H), 7.64 (t, *J* = 9.7 Hz, 1H), 7.25 (d, *J* = 4.3 Hz, 1H) ppm.

No-D ^{13}C{^{1}H} NMR (101 MHz, dioxane): δ 173.7, 167.2, 147.6, 143.4, 141.8, 140.6, 139.3, 139.1, 131.4, 130.2, 119.6, 117.1 ppm.

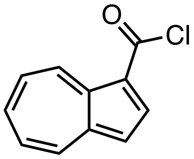

4a-Cl

Azulene-1-carbonyl chloride (**4a-Cl**). The NMR tube containing **3a-Cl** from above was heated to 90 ºC in the NMR probe for 3 h, then cooled to rt before acquiring no-D spectra of **4a-Cl**.

No-D ^{1}H NMR (400 MHz, dioxane): δ 9.26 (d, *J* = 10.2 Hz, 1H), 8.49 (d, *J* = 10.4 Hz, 1H), 8.33 (d, *J* = 4.3 Hz, 1H), 7.89 (t, *J* = 9.9 Hz, 1H), 7.70 – 7.55 (m, 2H), 7.22 (d, *J* = 4.2 Hz, 1H) ppm.

No-D ^{13}C{^{1}H} NMR (101 MHz, dioxane): δ 160.2, 147.2, 144.0, 141.5, 140.3, 139.4, 137.9, 130.6, 129.5, 118.7, 118.5 ppm.

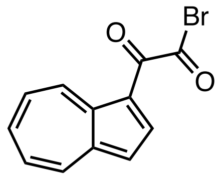

3a-Br

2-(Azulen-1-yl)-2-oxoacetyl bromide (**3a-Br**). A screw-cap NMR tube was charged with azulene **1** (31.0 mg, 0.242 mmol, 1.00 equiv), dioxane (0.5 mL), then inverted several times to dissolve the solid. Oxalyl bromide (23.0 µL, 0.245 mmol, 1.01 equiv) was added *via* syringe and the tube was again inverted several times to mix. The tube was inserted into the NMR and no-D NMR spectra of **3a-Br** were acquired at rt. Note that due to the high rate of the decarbonylation, signals for **4a-Br** are evident in the no-D ^{1}H NMR, and we were unable to obtain good 2D NMR spectra for this species for the same reason.

No-D ^{1}H NMR (400 MHz, dioxane): δ 9.64 (d, *J* = 9.2 Hz, 1H), 8.53 (d, *J* = 9.3 Hz, 1H), 8.28 (d, *J* = 4.3 Hz, 1H), 7.94 (t, *J* = 9.5 Hz, 2H), 7.74 (t, *J* = 9.8 Hz, 1H), 7.67 (td, *J* = 9.6, 3.8 Hz, 1H), 7.27 (t, *J* = 4.1 Hz, 1H) ppm.

No-D ^{13}C{^{1}H} NMR (101 MHz, dioxane): δ 173.1, 166.1, 147.8, 143.6, 141.5, 140.8, 139.3, 139.3, 131.6, 130.5, 119.8, 115.3 ppm.

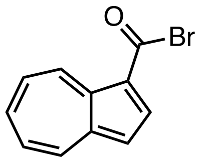

4a-Br

Azulene-1-carbonyl bromide (**4a-Br**). The NMR tube containing **3a-Br** from above was heated to 45 ºC in the NMR probe for 20 min, then cooled to rt before acquiring no-D spectra of **4a-Br**.

No-D ^{1}H NMR (400 MHz, dioxane): δ 9.26 (d, *J* = 10.2 Hz, 1H), 8.49 (d, *J* = 10.4 Hz, 1H), 8.33 (d, *J* = 4.3 Hz, 1H), 7.89 (t, *J* = 9.9 Hz, 1H), 7.70 – 7.55 (m, 2H), 7.22 (d, *J* = 4.2 Hz, 1H) ppm.

No-D ^{13}C{^{1}H} NMR (101 MHz, dioxane): δ 160.2, 147.2, 144.0, 141.5, 140.3, 139.4, 137.9, 130.6, 129.5, 118.7, 118.5 ppm.

II.h. *In Situ* IR Studies

In **dioxane without LiBr**, reactions of oxalyl chloride and oxalyl bromide show clean spectral profiles and exhibit **first-order kinetics** with respect to CO disappearance, consistent with a unimolecular decarbonylation of the acyl intermediate. Specifically, the product carbonyl absorptions shift as follows:

- **Oxalyl chloride:** 1642.7 cm^{-1} and 1782.5 cm^{-1} → 1740.1 cm^{-1}
- **Oxalyl bromide:** 1640.7 cm^{-1} and 1796.5 cm^{-1} → 1760.2 cm^{-1}

Guaiazulene + oxalyl chloride in dioxane

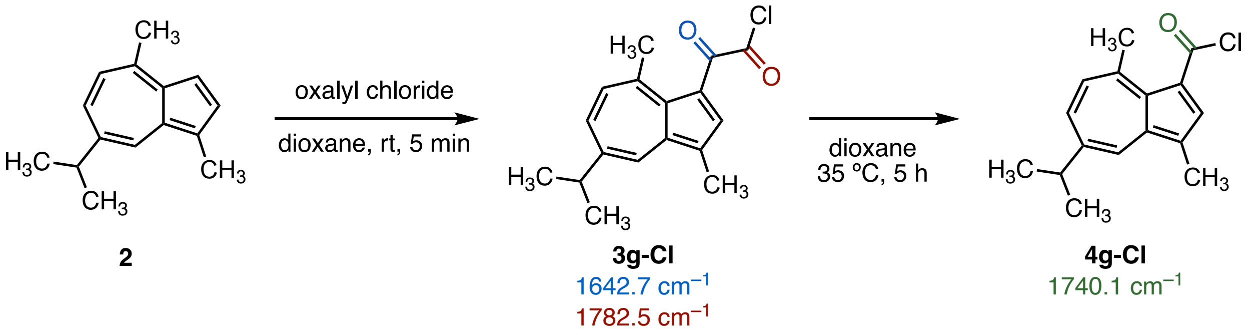

To a 25 mL multi-neck round-bottom flask equipped with a stir bar and fitted with a septum was added 1,4-dioxane (1 mL) at room temperature. Guaiazulene **2** (100 mg, 0.504 mmol, 1.00 equiv) was added, and the mixture was stirred until homogeneous. The flask was placed under ambient atmosphere and maintained at room temperature with stirring.

The ReactIR probe was blanked in air and then in 1,4-dioxane prior to insertion into the reaction vessel through the septum. Data acquisition was initiated using iC IR (ReactIR software).

Oxalyl chloride (45.0 µL, 0.532 mmol, 1.05 equiv) was added dropwise via syringe at room temperature to initiate the reaction. Infrared spectra were collected continuously over the course of the reaction. ReactIR data analysis was performed using the double derivative of the IR spectra to improve peak resolution and deconvolution. Reaction progress was monitored by trending the disappearance of starting material bands (1642.7 cm^{-1} and 1782.5 cm^{-1}) and the appearance of product-associated carbonyl absorptions (characteristic peaks at [1740.1 cm^{-1}]).

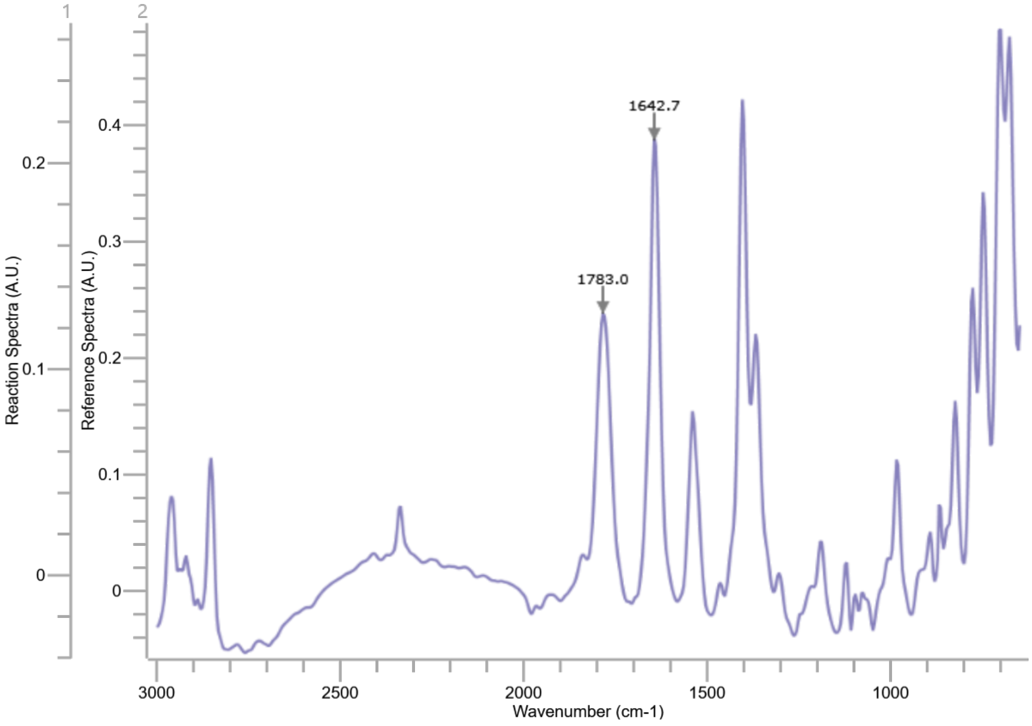

Figure S37. Reference spectrum for **3g-Cl** in dioxane.

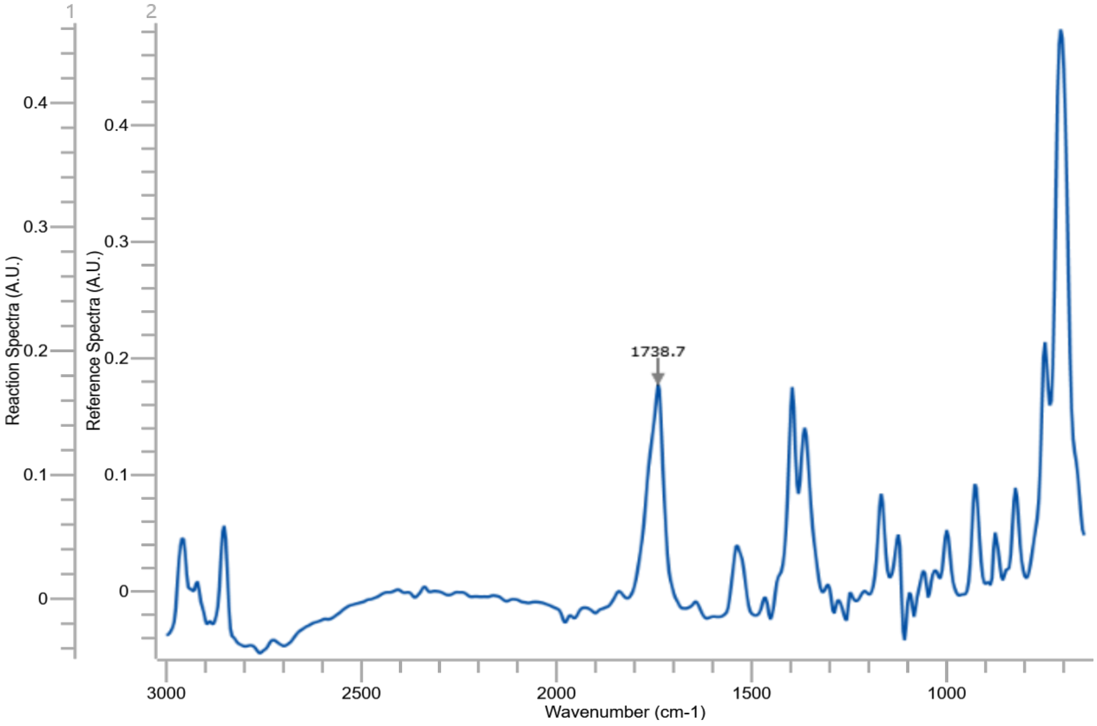

Figure S38. Reference spectrum for **4g-Cl** in dioxane.

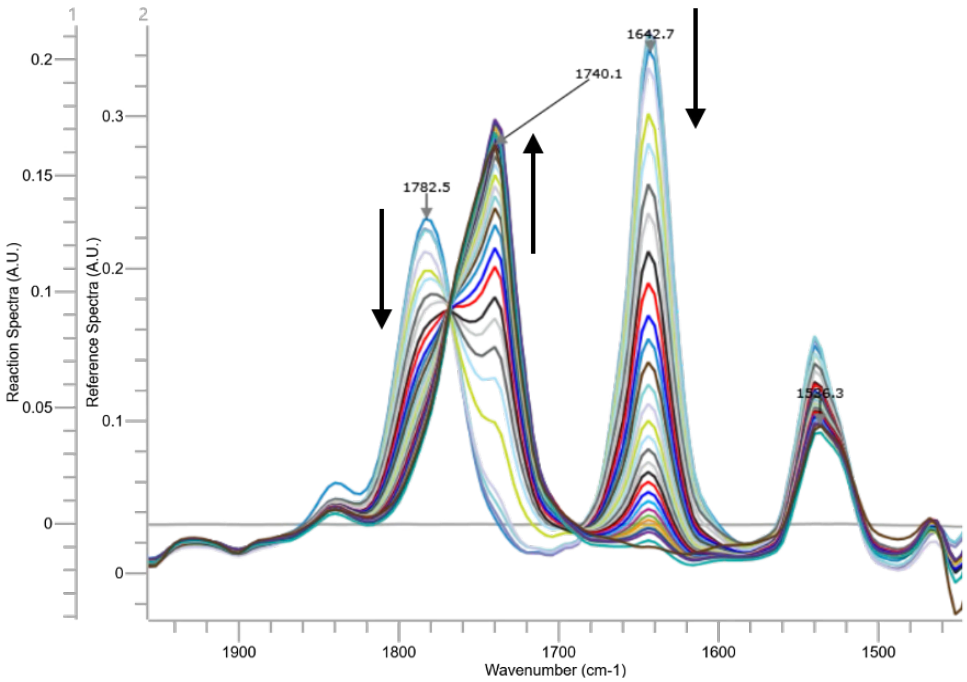

Figure S39. ReactIR data for reaction of guaiazulene + oxalyl chloride in dioxane at 35 ºC.

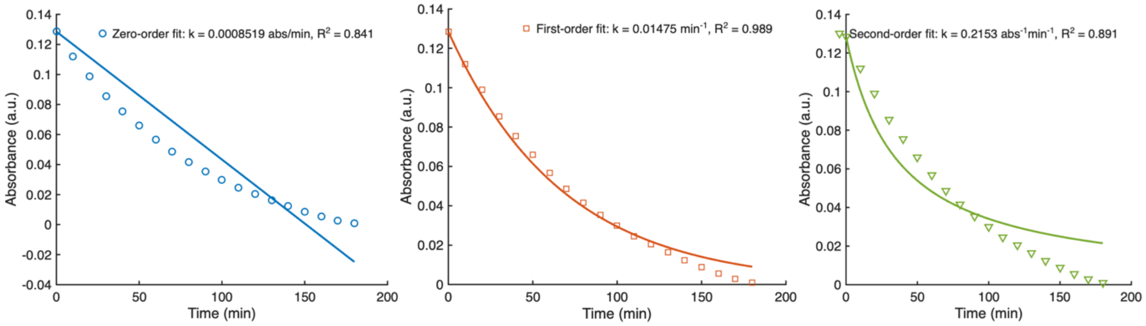

Figure S40. Kinetic plots for reaction of guaiazulene + oxalyl chloride in dioxane at 35 ºC *via* reactIR.

Guaiazulene + oxalyl bromide in dioxane

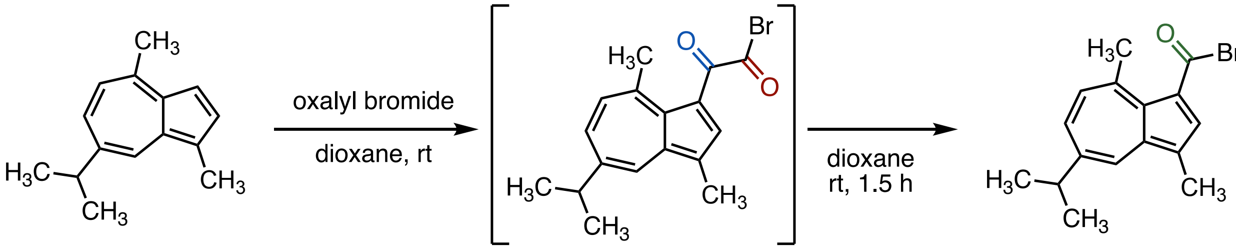

2 | 3g-Br: 1640.7 cm^{-1}, 1796.5 cm^{-1} | 4g-Br: 1760.2 cm^{-1}

To a 10 mL multi-neck round-bottom flask equipped with a stir bar and fitted with a septum was added 1,4-dioxane (1 mL) at room temperature. Guaiazulene (100 mg, 0.504 mmol, 1.00 equiv) was added, and the mixture was stirred until homogeneous. The flask was placed under ambient atmosphere and maintained at room temperature with stirring.

The ReactIR probe was blanked in air and then in 1,4-dioxane prior to insertion into the reaction vessel through the septum. Data acquisition was initiated using iC IR (ReactIR software).

Oxalyl bromide (40.0 μL, 0.426 mmol, 0.85 equiv) was added dropwise via syringe at room temperature to initiate the reaction. Infrared spectra were collected continuously over the course of the reaction. ReactIR data analysis was performed using the double derivative of the IR spectra to improve peak resolution and deconvolution. Reaction progress was monitored by trending the disappearance of starting material band (1640.7 cm^{-1} and 1796.5 cm^{-1}) and the appearance of product-associated carbonyl absorption (characteristic peak at 1760.2 cm^{-1}).

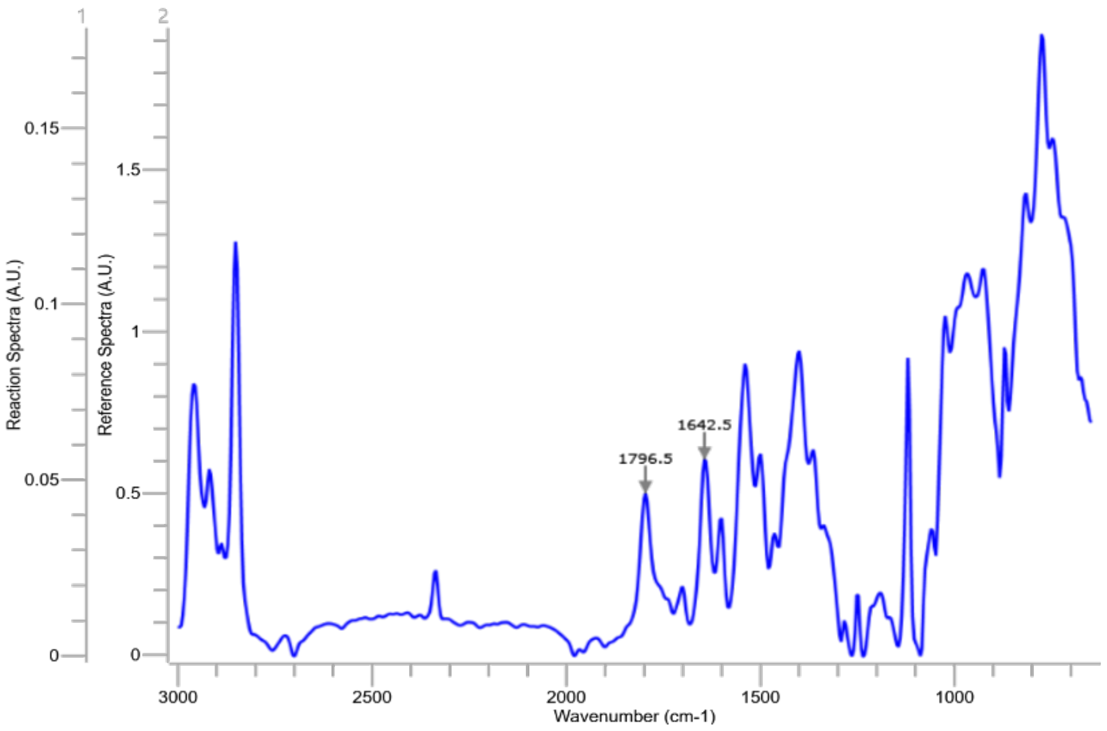

Figure S41. Reference spectrum for **3g-Br** in dioxane. Note that the signals for **4g-Br** are already apparent due to the rapid rate of decarbonylation with this substrate.

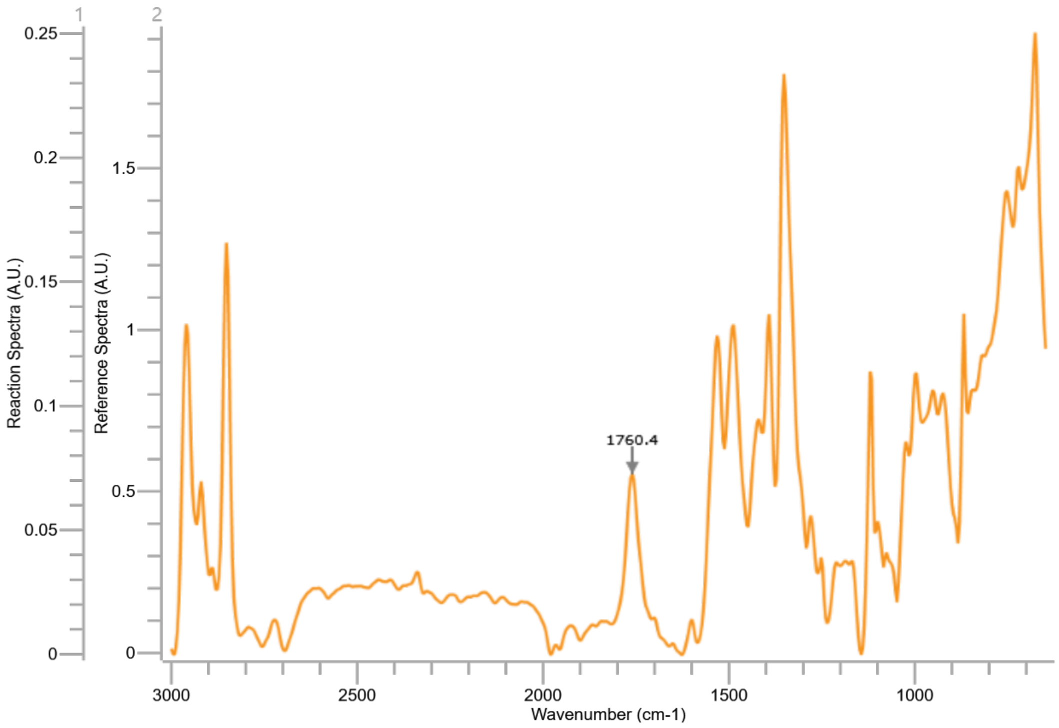

Figure S42. Reference spectrum for **4g-Br** in dioxane.

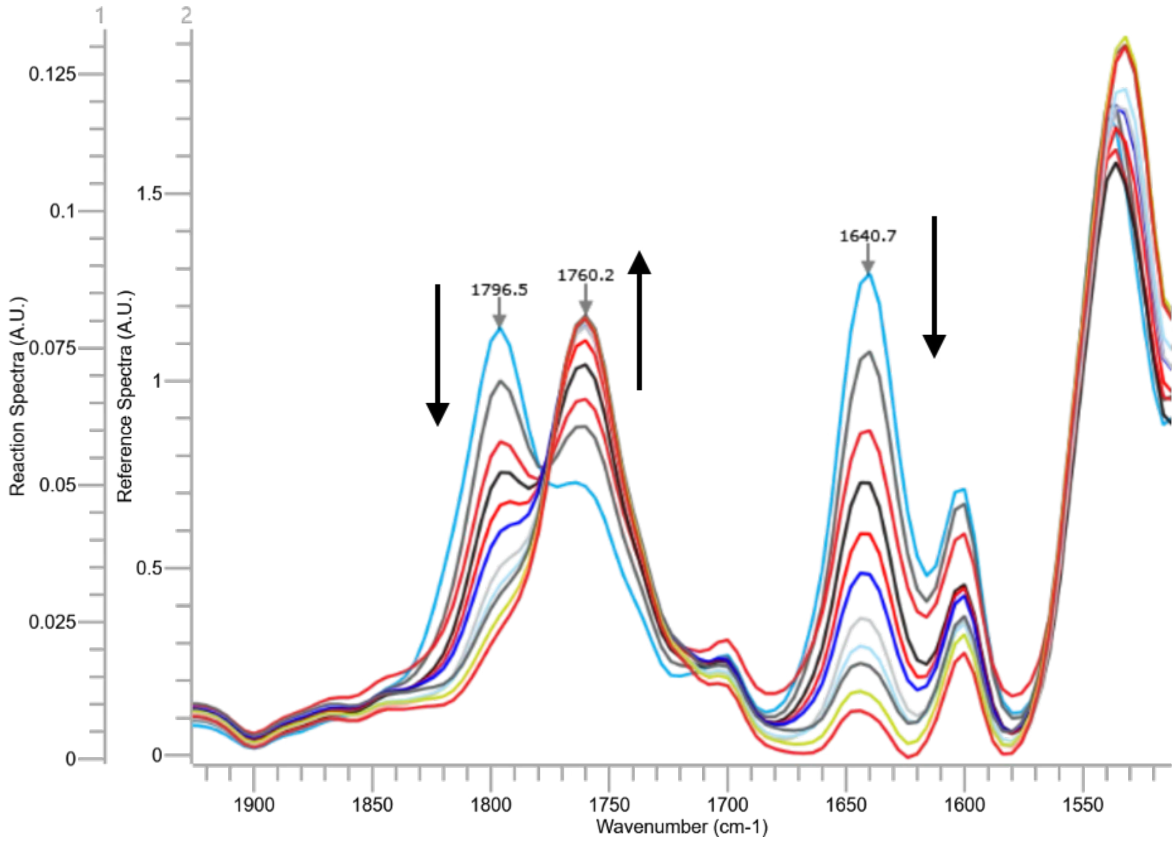

Figure S43. ReactIR data for reaction of guaiazulene + oxalyl chloride in dioxane at rt.

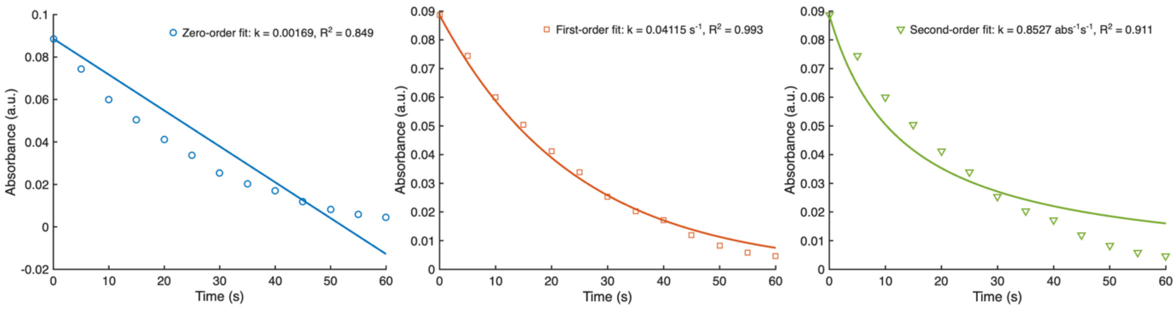

Figure S44. Kinetic plots for reaction of guaiazulene + oxalyl bromide in dioxane at rt.

Guaiazulene + oxalyl bromide in THF

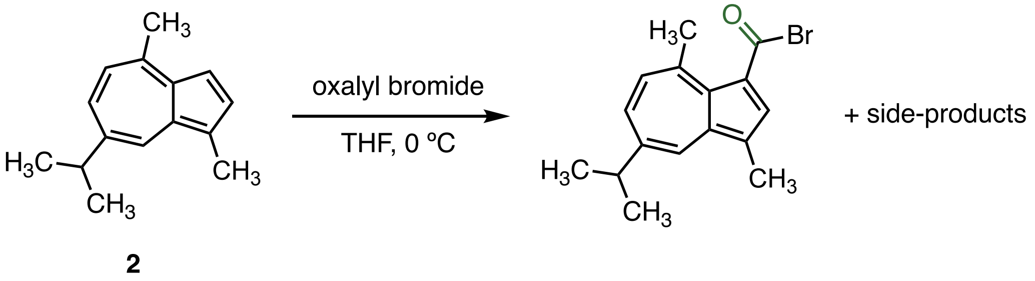

To a 10 mL multi-neck round-bottom flask equipped with a stir bar and fitted with a septum was added THF (solvent purification system-dry) (1 mL) at 0 °C. Guaiazulene (100 mg, 0.504 mmol, 1.00 equiv) was added, and the mixture was stirred until homogeneous. The flask was placed under ambient atmosphere and maintained at 0°C with stirring.

The ReactIR probe was blanked in air prior to insertion into the reaction vessel and then in THF into the reaction vessel through the septum. Data acquisition was initiated using iC IR (ReactIR software).

Oxalyl bromide (40.0 μL, 0.426 mmol, 0.85 equiv) was added dropwise via syringe at 0 °C to initiate the reaction. Infrared spectra were collected continuously over the course of the reaction (1 hour). ReactIR data analysis was performed using the double derivative of the IR spectra to improve peak resolution and deconvolution.

Note: The major product formed in dioxane (1760 cm^{-1}) differs from that obtained in THF (1702.0 cm^{-1}). In THF, significant byproduct formation is observed. The reaction mechanism cannot be clearly distinguished due to the simultaneous formation of multiple intermediates and side products.

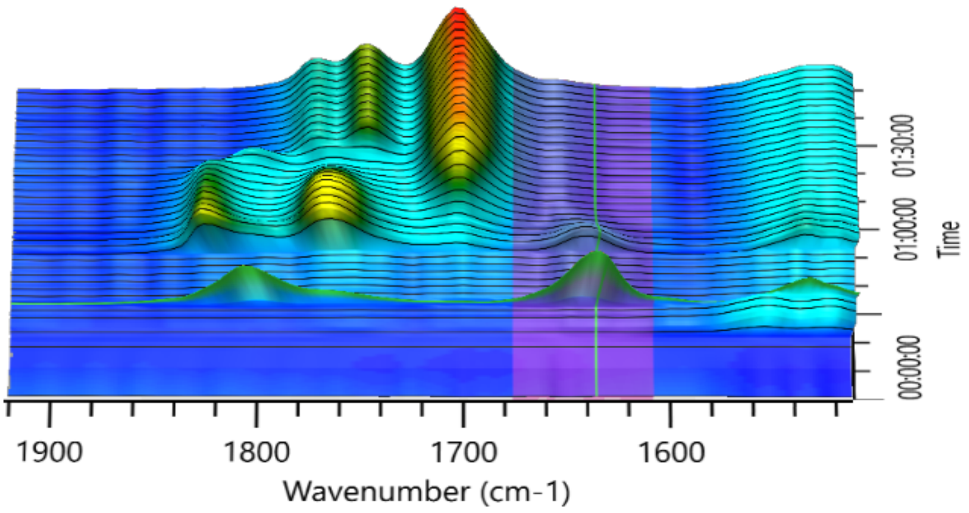

Figure S45. ReactIR data for reaction of guaiazulene + oxalyl bromide in THF at 0 ºC.

III. Computational Section

III.a. Computational Results

Our calculated transition state for intramolecular nucleophilic attack of the azulene π-system on the acyl chloride revealed a prohibitive activation barrier of 62.2 kcal/mol (Figure S46).

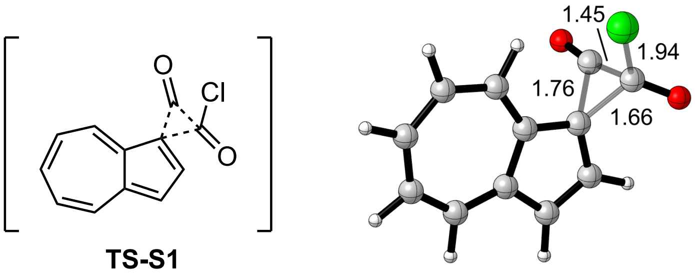

TS-S1
ΔG‡ = 62.2

Figure S46. Calculated transition state structure and activation free energy (kcal/mol) of the intramolecular π-system nucleophilic attack pathway for **3a-Cl** at the M06-2X/def2-TZVPP, SMD(1,4-dioxane)//M06-2X/def2-SVP, SMD(1,4-dioxane) level of theory. Interatomic distances are in Å.

Calculated transition states of the 1,2-chloride shift for 2-oxo-2-phenylacetyl chloride and oxalyl chloride are shown in Figure S47. While the barrier for oxalyl chloride is significantly higher than that for **3a-Cl**, the result for 2-oxo-2-phenylacetyl chloride is almost identical to the computed result for **3a-Cl**. This result may suggest that the 1,2-halide shift mechanism could occur more generally in systems other than those involving azulene scaffolds.

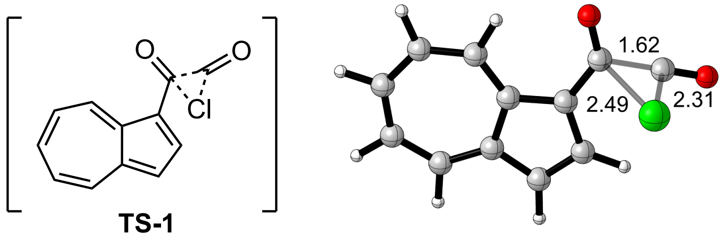

TS-1
ΔG‡ = 31.1

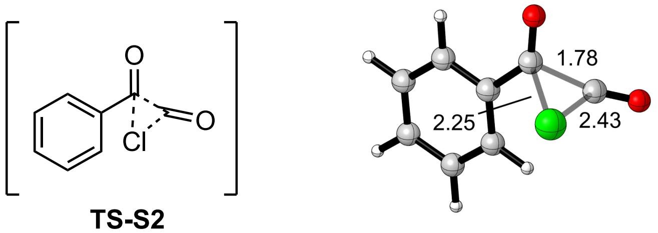

TS-S2
ΔG‡ = 31.0

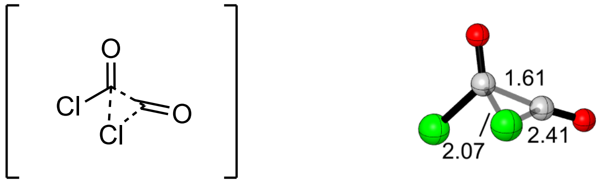

TS-S3
ΔG‡ = 38.6

Figure S47. Calculated structures and activation free energies (kcal/mol) of 1,2-chloride shift transition states for **3a-Cl**, 2-oxo-2-phenylacetyl chloride and oxalyl chloride at the M06-2X/def2-TZVPP, SMD(1,4-dioxane)//M06-2X/def2-SVP, SMD(1,4-dioxane) level of theory. Interatomic distances are in Å.

Calculated transition states for the potential 1,2-migration of Br, O- and N-containing groups are shown in Figure S48.

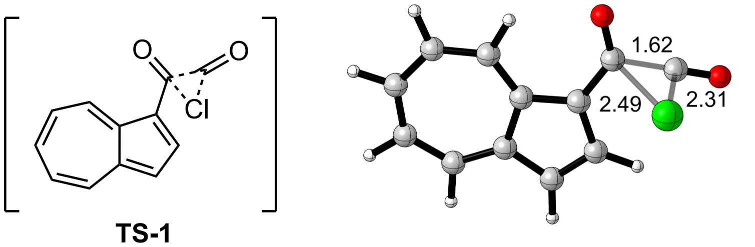

TS-1
ΔG‡ = 31.1

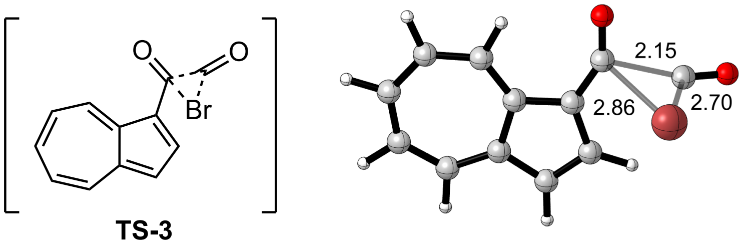

TS-3
ΔG‡ = 27.2

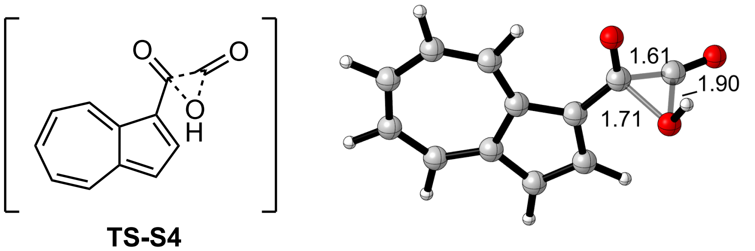

TS-S4
ΔG‡ = 60.3

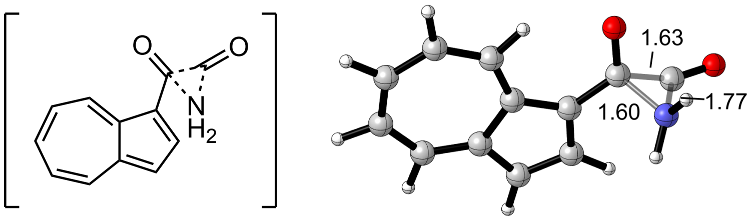

TS-S5
ΔG‡ = 68.5

Figure S48. Calculated structures and activation free energies (kcal/mol) of 1,2-migration transition states for **3a-Cl** (chloride migration), **3a-Br** (bromide migration), **7m** (OH migration), and **7a** (NH_2 migration) at the M06-2X/def2-TZVPP, SMD(1,4-dioxane)//M06-2X/def2-SVP, SMD(1,4-dioxane) level of theory. Interatomic distances are in Å.

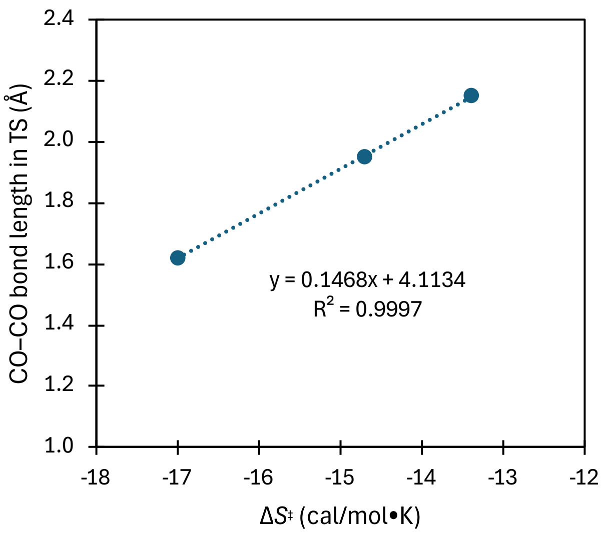

Figure S49. Relationship between calculated intercarbonyl C–C bond length in **TS-1**, **TS-2**, and **TS-3** and experimental Δ$S^‡$ for decarbonylation of **3a-Cl**, **3g-Cl**, and **3a-Br**.

III.b. Calculated Energies

Table S18. Calculated energies in Hartrees.

Structure	**Δ*G*** [M06-2X/def2-SVP, SMD(1,4-dioxane)]	**SPE** [M06-2X/def2-TZVPP, SMD(1,4-dioxane)]	***G*** (= Δ*G* + SPE)	**Imaginary Frequency** (cm^{-1})
**3a-Cl**	0.110946	-1072.088898	-1071.977952	-
**3g-Cl**	0.238917	-1268.630390	-1268.391473	-
**3a-Br**	0.106761	-3186.082494	-3185.975733	-
**2-oxo-2-phenylacetyl chloride**	0.068895	-918.501455	-918.432560	-
**oxalyl chloride**	-0.015539	-1147.058800	-1147.074339	-
**7m**	0.124630	-687.735309	-687.610679	-
**7a**	0.136768	-667.866680	-667.729912	-
**TS-1**	0.107286	-1072.035754	-1071.928468	-374.50
**TS-2**	0.234922	-1268.579520	-1268.344598	-372.52
**TS-3**	0.101570	-3186.034015	-3185.932445	-311.17
**TS-S1**	0.107400	-1071.986225	-1071.878825	-562.76
**TS-S2**	0.065141	-918.448367	-918.383226	-433.12
**TS-S3**	-0.019797	-1146.993091	-1147.012888	-406.19
**TS-S4**	0.119122	-687.633647	-687.514525	-530.96
**TS-S5**	0.131158	-667.751910	-667.620752	-541.49

III.c. Cartesian Coordinates of Calculated Structures

3a-Cl

C	-3.97446300	-1.25645000	0.02342000
C	-4.22669200	0.11362900	-0.10278100
C	-3.31094700	1.15821100	-0.16058100
C	-2.75717800	-1.92611700	0.12398700
C	-1.91476800	1.10850100	-0.10692000
C	-1.45800500	-1.40869800	0.12706300
C	-1.05109600	-0.07933100	0.02814500
H	-4.86202700	-1.89412300	0.04607100
H	-5.27909100	0.39882900	-0.16424900
H	-3.73405100	2.16191800	-0.26274700
H	-2.82408500	-3.01250700	0.21392700
H	-0.65101300	-2.13662900	0.21666000
C	-1.07043100	2.23951400	-0.17586600
H	-1.41282800	3.26708000	-0.28118400
C	0.24698800	1.81577700	-0.08567500
H	1.11522400	2.47040700	-0.10823700
C	0.29386400	0.40069900	0.04017300
C	1.47482200	-0.43821900	0.13113700
O	1.50192200	-1.64835500	0.15199300
C	2.83595700	0.30580400	0.23871600
O	3.02345100	1.36192200	0.73228000
Cl	4.17156300	-0.65667200	-0.42466700

3g-Cl

C	2.80800300	-1.72793100	-0.24991500
C	3.20175600	-0.38645300	-0.11411600
C	2.31252900	0.68330500	-0.12170100
C	1.55605600	-2.29873100	-0.42923700
C	0.91413400	0.70273500	-0.15426000
C	0.25263600	-1.75866300	-0.44027800
C	-0.05959600	-0.41159000	-0.18709200
H	3.63256800	-2.44877800	-0.25946500
H	2.76231800	1.67781300	-0.07021300
H	1.58575300	-3.37846200	-0.59678800
C	0.18749400	1.92499400	-0.11969200
C	-1.16067100	1.60819300	-0.09627500
H	-1.96182700	2.34384800	-0.03120400
C	-1.34993200	0.19859100	-0.10814300
C	-2.63317900	-0.41009200	0.20328000
O	-2.83973000	-1.46892800	0.74658000
C	-3.87498500	0.43811400	-0.21074300
O	-3.91894500	1.23691900	-1.07857600
Cl	-5.31853000	0.02141500	0.73894500
C	-0.83207200	-2.74783200	-0.77708200
H	-1.32115400	-3.12176200	0.13230700
H	-1.61300900	-2.28707200	-1.39435400
H	-0.40890400	-3.59803700	-1.32600800
C	0.77949300	3.29999100	-0.08264600
H	1.40003900	3.45604600	0.81383600
H	1.41366700	3.49719100	-0.96087000
H	-0.01482100	4.05782900	-0.07134100
C	4.69532700	-0.13903100	0.06874100
H	5.21271600	-0.87656400	-0.56626400
C	5.08778400	-0.42117900	1.52450700
H	6.17284900	-0.30435500	1.66352800
H	4.57680700	0.28109300	2.20126000
H	4.81150800	-1.44130700	1.82842300
C	5.16889200	1.24835200	-0.35424300
H	4.81083900	2.02931600	0.33376200
H	6.26743100	1.28512400	-0.33846000
H	4.83561600	1.50345200	-1.37088200

3a-Br

C	-4.52854300	-1.37667900	-0.06372300
C	-4.83039100	-0.02338200	-0.26936600
C	-3.95929600	1.06365100	-0.31424900
C	-3.28980100	-1.98356800	0.15008700
C	-2.56764200	1.08235900	-0.16556600
C	-2.01748300	-1.40074100	0.21841800
C	-1.66572400	-0.05812600	0.08445400
H	-5.38792400	-2.05410600	-0.07154100
H	-5.88893200	0.20970800	-0.41429900
H	-4.42141900	2.04052600	-0.48969000
H	-3.31291500	-3.06851800	0.28547300
H	-1.18372300	-2.08099800	0.39904700
C	-1.76392600	2.25196500	-0.23146200
H	-2.14408300	3.25768400	-0.40760400
C	-0.43669400	1.89567200	-0.03477000
H	0.40162600	2.58959400	-0.03251500
C	-0.33637100	0.48364400	0.16329500
C	0.86630000	-0.28510700	0.37312000
O	0.95831400	-1.49583500	0.50778100
C	2.18272900	0.53774200	0.46499600
O	2.34688900	1.57212700	1.01476200
Br	3.70219400	-0.41796600	-0.39144600

2-oxo-2-phenylacetyl chloride

C	-0.64116100	0.65146900	0.10444200
O	-1.00828500	1.79428900	0.08070800
C	-1.71125700	-0.45704900	0.28978700
O	-1.55318600	-1.46924200	0.87682400
Cl	-3.26946000	-0.00592600	-0.40938600
C	0.78247400	0.23215700	0.02909800
C	1.18387600	-1.08709700	-0.22125600
C	1.74648400	1.24103500	0.17266800
C	2.54016800	-1.38802000	-0.32091100
H	0.44866000	-1.88139000	-0.34396400
C	3.09759200	0.93209600	0.08333800
H	1.41358800	2.26346800	0.35605600
C	3.49463600	-0.38412600	-0.16388900
H	2.85208000	-2.41391700	-0.52013400
H	3.84538800	1.71697600	0.20393300
H	4.55601000	-0.62755400	-0.23625300

oxalyl chloride

C	0.31190700	0.71145600	0.00023400
O	-0.31190700	1.70917600	0.00012000
C	-0.31190700	-0.71145600	0.00023400
O	0.31190700	-1.70917600	0.00012000
Cl	-2.05780400	-0.64884900	-0.00013900
Cl	2.05780400	0.64884900	-0.00013900

7m

C	-3.72546400	-1.07630000	0.03977800
C	-3.88016500	0.31416100	-0.04391200
C	-2.89206200	1.29663600	-0.09515900
C	-2.55406700	-1.83416800	0.09488400
C	-1.50084900	1.14795200	-0.07499100
C	-1.22002100	-1.40594800	0.08337900
C	-0.72063600	-0.10568600	0.01093500
H	-4.65713700	-1.64987200	0.06556600
H	-4.91180500	0.67572700	-0.07400200
H	-3.24843800	2.32986500	-0.16026400
H	-2.69554500	-2.91693000	0.15717900
H	-0.45882500	-2.18593900	0.13751800
C	-0.57184000	2.21859300	-0.13286300
H	-0.84129200	3.27219400	-0.20173500
C	0.71614900	1.69657400	-0.08346800
H	1.63411800	2.28022800	-0.10272800
C	0.66343100	0.27286000	0.00569500
C	1.79176700	-0.63600200	0.04193000
O	1.72958300	-1.86086800	0.07331700
C	3.19900500	-0.00531000	0.07757800
O	3.50015200	0.97907900	0.71111900
O	4.07055600	-0.71936200	-0.63759700
H	4.94509700	-0.29623300	-0.53895700

7a

C	3.70322400	-1.14787600	0.00015700
C	3.89715500	0.24007800	0.00012600
C	2.93588300	1.24977000	0.00004700
C	2.50963900	-1.87154800	0.00011100
C	1.54079700	1.13999000	-0.00002400
C	1.18738500	-1.40613600	0.00001400
C	0.72046800	-0.09183500	-0.00004900
H	4.61776900	-1.74898900	0.00022700
H	4.93837200	0.57451000	0.00017200
H	3.32020300	2.27511000	0.00004100
H	2.61910600	-2.95988300	0.00015300
H	0.40627000	-2.16742400	-0.00001900
C	0.64681100	2.24024000	-0.00009100
H	0.95084400	3.28679200	-0.00009400
C	-0.65781400	1.75919900	-0.00015600
H	-1.56557300	2.35555800	-0.00022500
C	-0.65429800	0.33028500	-0.00013800
C	-1.80327200	-0.55472600	-0.00017000
O	-1.72949700	-1.78582800	-0.00004600
C	-3.24021900	0.05190600	-0.00015400
O	-3.48784200	1.25093000	-0.00060600
N	-4.17513300	-0.91621000	0.00078900
H	-5.15910700	-0.67299600	0.00061900
H	-3.87779500	-1.88610700	0.00079800

TS-1

C	3.95501900	-0.93086900	0.13374700
C	4.03319200	0.46187400	0.14300300
C	2.99493200	1.38859300	0.05609700
C	2.83417000	-1.75959500	0.03568600
C	1.62450900	1.16161100	-0.06113300
C	1.48925700	-1.41254400	-0.07592800
C	0.92268900	-0.13640200	-0.12155300
H	4.91212900	-1.45219500	0.21670200
H	5.03765000	0.88085800	0.23195900
H	3.28923000	2.44181400	0.08606600
H	3.03950500	-2.83200000	0.05279000
H	0.78245200	-2.24272700	-0.12812000
C	0.64216600	2.17982200	-0.13336400
H	0.84743500	3.24766900	-0.09789900
C	-0.60442000	1.58437300	-0.23066200
H	-1.55850100	2.11233500	-0.25844200
C	-0.45533800	0.17475400	-0.23692600
C	-1.50873600	-0.80955600	-0.37013000
O	-1.48986400	-2.00107400	-0.36270200
C	-2.86904400	-0.11742200	-0.90474300
O	-3.82321900	0.36794100	-1.27970900
Cl	-3.07033000	0.01185200	1.39009800

TS-2

C	2.59838500	-1.82087300	-0.05073600
C	3.04068700	-0.49712700	0.00781500
C	2.18635400	0.60914400	0.00922600
C	1.31246100	-2.35820000	-0.11829300
C	0.80163300	0.68970900	-0.10806700
C	0.04110900	-1.77246300	-0.21274700
C	-0.20071100	-0.38380300	-0.28731600
H	3.39160200	-2.57379500	-0.01459800
H	2.67889800	1.58224000	0.10774500
H	1.29558600	-3.45034200	-0.09507900
C	0.11975400	1.94316700	-0.09706100
C	-1.22476600	1.68670600	-0.28071200
H	-1.99707000	2.45403100	-0.34450900
C	-1.44407300	0.29150000	-0.41910000
C	-2.71902700	-0.29376700	-0.74504200
O	-3.21146500	-1.05081000	-1.47394300
C	-4.01267200	1.08403900	-0.26529100
O	-4.93080500	1.72107300	-0.10610100
Cl	-3.65037200	-0.66729600	1.45982400
C	-1.12024300	-2.72922200	-0.21925400
H	-1.91117800	-2.39200900	0.46683500
H	-1.56318100	-2.80447800	-1.22299300
H	-0.79583300	-3.73146800	0.08337100
C	0.75528200	3.28935500	0.05771200
H	1.29342300	3.37794400	1.01392700
H	1.47579300	3.49723400	-0.74864500
H	-0.00759800	4.07836500	0.03296800
C	4.53815000	-0.23861800	0.11675900
H	5.03668000	-1.21707100	0.04120300
C	4.89991600	0.36604800	1.47620200
H	5.98937500	0.49090400	1.56332600
H	4.43971400	1.35816200	1.60593800
H	4.55998800	-0.27437800	2.30264900
C	5.05126700	0.63454400	-1.03067100
H	4.62248700	1.64780400	-0.98829800
H	6.14478600	0.73751200	-0.97198800
H	4.79997400	0.20044300	-2.00905600

TS-3

C	-4.48845600	-0.88537600	-0.31909500
C	-4.52399900	0.51207500	-0.35801200
C	-3.47462800	1.41630500	-0.18008900
C	-3.40305800	-1.73951000	-0.09411200
C	-2.12767500	1.15593000	0.08270900
C	-2.06658900	-1.41811300	0.15430300
C	-1.47941700	-0.15572900	0.24048600
H	-5.44693000	-1.38397600	-0.49102300
H	-5.50312500	0.95678700	-0.55552500
H	-3.73645400	2.47611000	-0.25771100
H	-3.63024800	-2.80865300	-0.11569400
H	-1.40064400	-2.27326200	0.29694800
C	-1.12360900	2.15638200	0.24590700
H	-1.30331600	3.22847700	0.17912300
C	0.09107800	1.54465000	0.48996200
H	1.04892600	2.04149100	0.63264000
C	-0.09506200	0.13046000	0.50204200
C	0.84216000	-0.89074200	0.69010600
O	1.02739500	-2.02956000	0.82332400
C	2.54625600	0.13025000	1.51431900
O	3.49441200	0.39490000	2.08803700
Br	2.84615200	-0.02569100	-1.16545200

TS-S1

C	-3.37225200	-1.39001400	0.04443800
C	-3.75419100	-0.05520400	-0.12590500
C	-2.94299000	1.06901600	-0.21519600
C	-2.09502900	-1.92961100	0.16719100
C	-1.54643200	1.15478400	-0.17626500
C	-0.85290300	-1.28759300	0.14088500
C	-0.57379400	0.06503000	-0.03525800
H	-4.19265400	-2.11025200	0.09379700
H	-4.82897300	0.12731700	-0.19022900
H	-3.45990000	2.02553500	-0.33390900
H	-2.05259800	-3.01231200	0.30361800
H	0.01258300	-1.94041600	0.26196500
C	-0.82493000	2.37565600	-0.27143300
H	-1.28151500	3.35549700	-0.39849100
C	0.52142000	2.09793600	-0.19591800
H	1.34254500	2.81036400	-0.27176100
C	0.72487900	0.68829700	-0.04507100
C	1.89099100	0.15545100	1.16282800
O	1.75532500	0.00892600	2.30714700
C	2.30087600	0.21219900	-0.22659700
O	3.09830400	0.97110200	-0.75449300
Cl	2.28103500	-1.64892000	-0.77814200

TS-S2

C	0.81267500	0.84920200	-0.15272300
O	1.19953700	1.95510600	-0.24189800
C	1.82853300	-0.31567900	-1.03061200
O	2.63411600	-0.84748800	-1.61147400
Cl	1.93459500	-0.34730300	1.39459400
C	-0.56474100	0.30022300	-0.07967500
C	-0.83939900	-1.06859300	-0.06046200
C	-1.59662500	1.24050300	-0.06131700
C	-2.16046200	-1.49774100	-0.03420400
H	-0.02019800	-1.79034600	-0.03316600
C	-2.91878000	0.80080200	-0.02556200
H	-1.35745300	2.30439300	-0.07836500
C	-3.19981600	-0.56355600	-0.01466800
H	-2.38222000	-2.56528600	-0.01676900
H	-3.73033600	1.52928400	-0.00968000
H	-4.23543700	-0.90580100	0.01219800

TS-S3

C	0.30550700	-0.05472500	0.57850100
O	0.44005700	-0.01767700	1.75417100
C	-0.84023900	-1.00100000	-0.03421500
O	-1.67419200	-1.68294700	-0.34633000
Cl	-0.89185200	1.39503600	-0.27854000
Cl	1.66135100	-0.22213400	-0.57607400

TS-S4

C	3.63765400	-1.11712100	-0.12637800
C	3.82427300	0.26892400	-0.13506500
C	2.85685500	1.27690500	-0.08419200
C	2.45039100	-1.85663100	-0.06341900
C	1.46796700	1.16138700	-0.01008300
C	1.13097000	-1.40276300	0.01306300
C	0.65647600	-0.08804100	0.04007600
H	4.55523600	-1.71202100	-0.17581700
H	4.86215400	0.60916200	-0.18986400
H	3.23684300	2.30390000	-0.10562500
H	2.57176000	-2.94357600	-0.07289700
H	0.35631900	-2.17113900	0.06109700
C	0.56206300	2.24782400	0.03480400
H	0.84703200	3.29943600	0.01872500
C	-0.73735000	1.74005200	0.10978800
H	-1.64892200	2.33693900	0.16434000
C	-0.69899000	0.32434400	0.11493800
C	-1.87276800	-0.56788400	0.16955600
O	-1.86919200	-1.79359100	0.23715600
C	-3.14436900	-0.02699600	-0.65383200
O	-4.16023600	-0.01190900	-1.18236000
O	-2.98317100	0.19016800	1.22494700
H	-3.47866700	-0.56005700	1.60656600

TS-S5

C	3.64348300	-1.11463100	-0.05466000
C	3.83578800	0.27100100	-0.04810600
C	2.87077000	1.28302600	-0.03401100
C	2.45286100	-1.85170500	-0.04790900
C	1.47993200	1.17218800	-0.01898300
C	1.13240600	-1.39357900	-0.02844100
C	0.66146400	-0.07734200	-0.01356600
H	4.56085100	-1.71208600	-0.06782700
H	4.87650400	0.60701600	-0.05619200
H	3.25571800	2.30861100	-0.03351300
H	2.57208100	-2.93897600	-0.05941000
H	0.34713800	-2.15317000	-0.03252000
C	0.57474300	2.25745300	-0.00784700
H	0.85855400	3.30961300	-0.01808600
C	-0.72917900	1.74622800	0.00410200
H	-1.63984900	2.34983500	-0.00123400
C	-0.69537300	0.33181000	0.00588000
C	-1.88048000	-0.57864900	0.05506900
O	-1.84468100	-1.79824100	-0.20290500
C	-3.26414800	0.12065600	-0.44099600
O	-4.35213900	0.15407800	-0.84907000
N	-2.85560400	-0.17114200	1.26131900
H	-2.50744200	0.56162100	1.88447900
H	-3.25335700	-0.97990900	1.74767700

IV. References

1. Hoffman, R.E.; Becker, E.D. *J. Magn. Reson.* **2005**, *176*, 87.
2. Gaussian 16, Revision A.03, M. J. Frisch, G. W. Trucks, H. B. Schlegel, G. E. Scuseria, M. A. Robb, J. R. Cheeseman, G. Scalmani, V. Barone, G. A. Petersson, H. Nakatsuji, X. Li, M. Caricato, A. V. Marenich, J. Bloino, B. G. Janesko, R. Gomperts, B. Mennucci, H. P. Hratchian, J. V. Ortiz, A. F. Izmaylov, J. L. Sonnenberg, D. Williams-Young, F. Ding, F. Lipparini, F. Egidi, J. Goings, B. Peng, A. Petrone, T. Henderson, D. Ranasinghe, V. G. Zakrzewski, J. Gao, N. Rega, G. Zheng, W. Liang, M. Hada, M. Ehara, K. Toyota, R. Fukuda, J. Hasegawa, M. Ishida, T. Nakajima, Y. Honda, O. Kitao, H. Nakai, T. Vreven, K. Throssell, J. A. Montgomery, Jr., J. E. Peralta, F. Ogliaro, M. J. Bearpark, J. J. Heyd, E. N. Brothers, K. N. Kudin, V. N. Staroverov, T. A. Keith, R. Kobayashi, J. Normand, K. Raghavachari, A. P. Rendell, J. C. Burant, S. S. Iyengar, J. Tomasi, M. Cossi, J. M. Millam, M. Klene, C. Adamo, R. Cammi, J. W. Ochterski, R. L. Martin, K. Morokuma, O. Farkas, J. B. Foresman, and D. J. Fox, Gaussian, Inc., Wallingford CT, **2016**.
3. Zhao, Y.; Truhlar, D. G. The M06 suite of density functionals for main group thermochemistry, thermochemical kinetics, noncovalent interactions, excited states, and transition elements: two new functionals and systematic testing of four M06-class functionals and 12 other functionals. *Theor. Chem. Acc.* **2008**, *120*, 215-241.
4. Grimme, S., Supramolecular Binding Thermodynamics by Dispersion-Corrected Density Functional Theory. *Chem. Eur. J.* **2012**, *18*, 9955-9964.
5. Luchini, G.; Alegre-Requena, J. V.; Funes-Ardoiz, I.; Paton, R. S. GoodVibes: Automated Thermochemistry for Heterogeneous Computational Chemistry Data. *F1000Research* **2020**, *9*, 291-304.
6. Marenich, A. V.; Cramer, C. J.; Truhlar, D. G. Universal Solvation Model Based on Solute Electron Density and on a Continuum Model of the Solvent Defined by the Bulk Dielectric Constant and Atomic Surface Tensions. *J. Phys. Chem. B* **2009**, *113*, 6378-6396.
7. Legault, C. Y. CYLview, 1.0b; Université de Sherbrooke, **2009**; http://www.cylview.org.
8. Spartan ’24, Wavefunction, Inc. Irvine, CA.

V. NMR Spectra

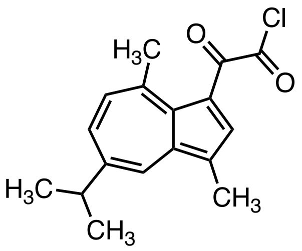

3g-Cl

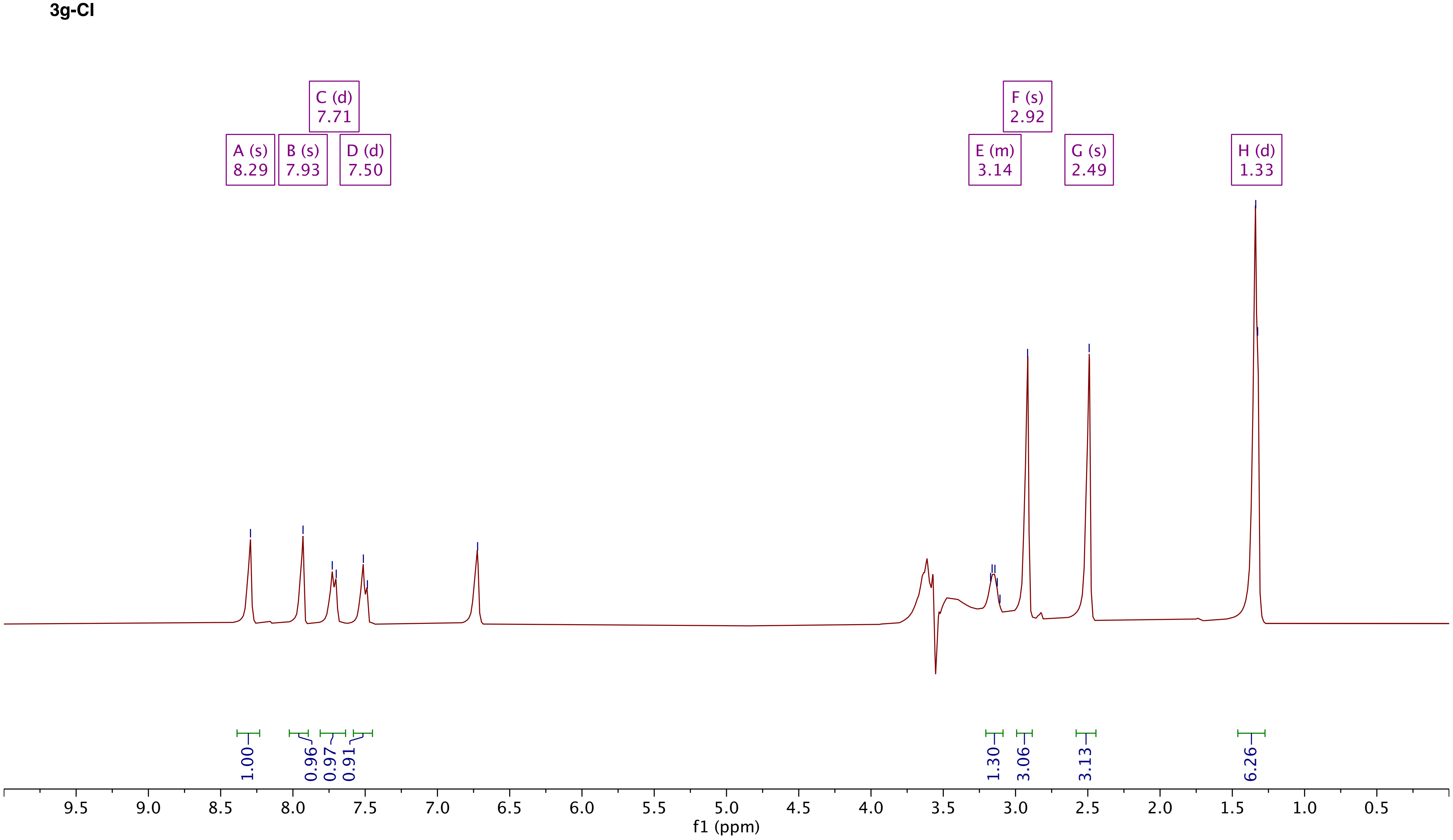

400 MHz no-D ^{1}H-NMR spectrum of **3g-Cl** in dioxane

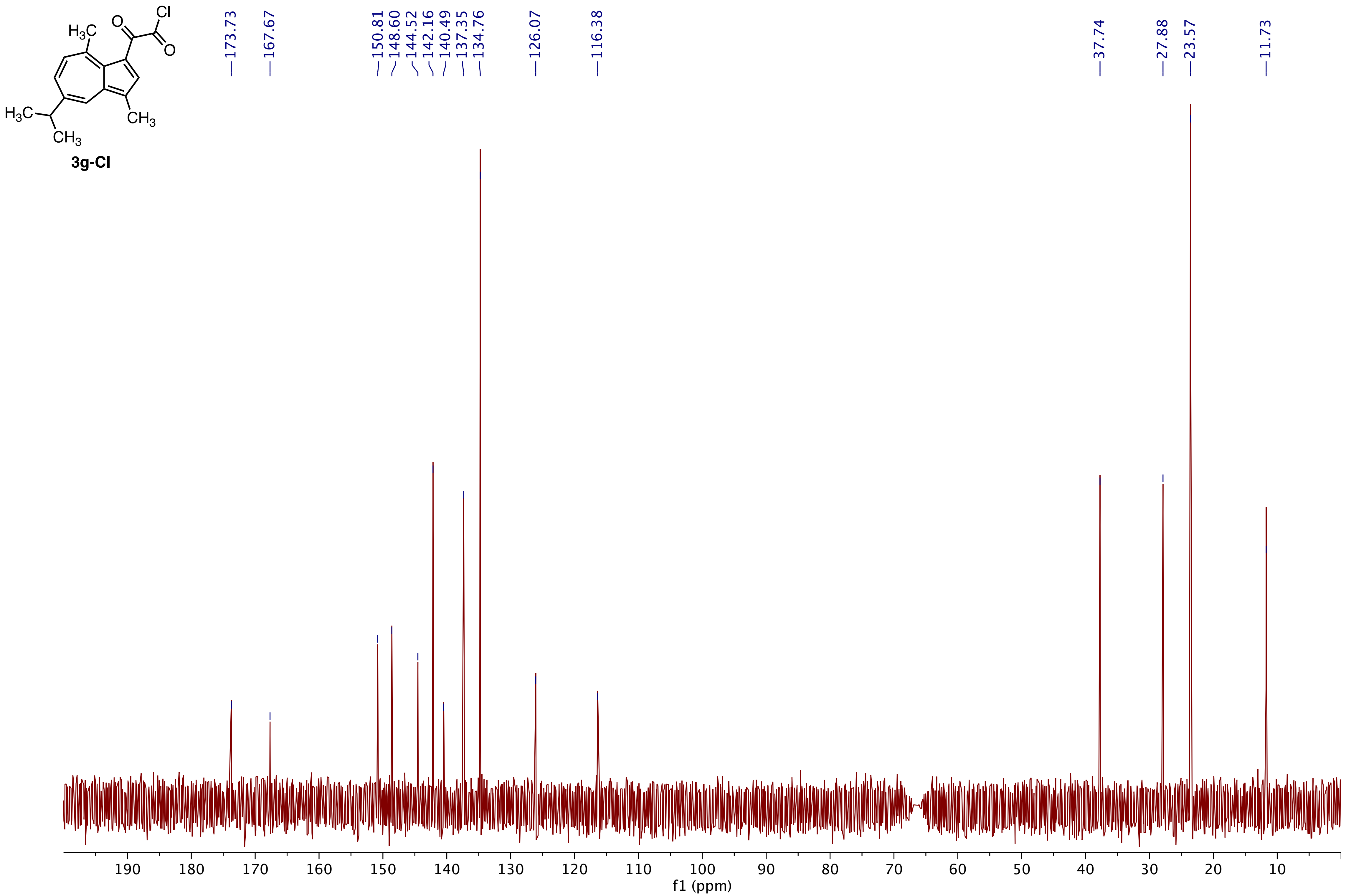

3g-Cl

101 MHz no-D $^{13}C\{^{1}H\}$-NMR spectrum of **3g-Cl** in dioxane

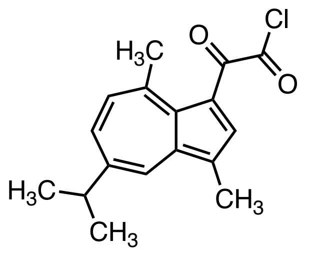

3g-Cl

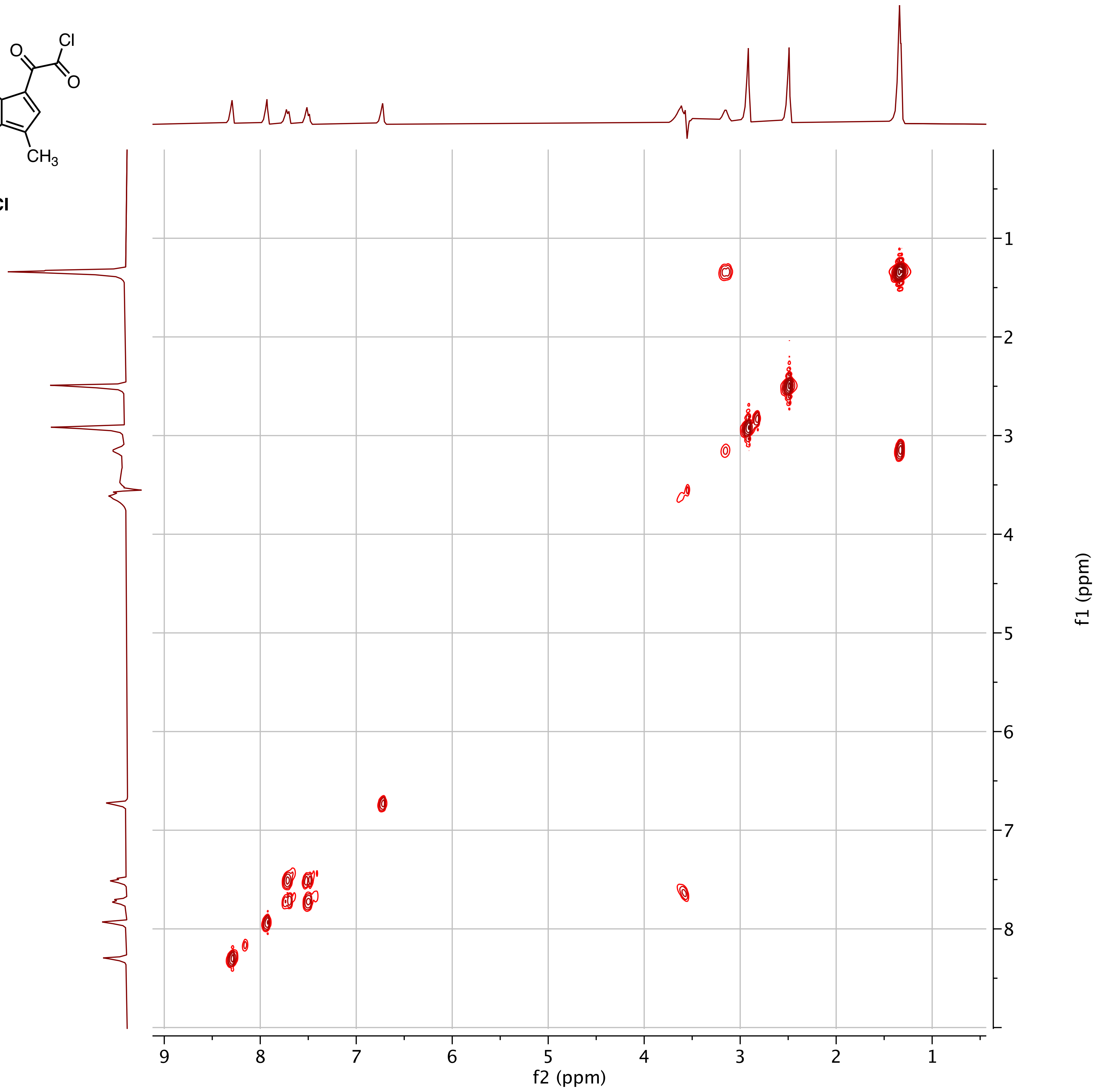

No-D COSY NMR spectrum of **3g-Cl** in dioxane

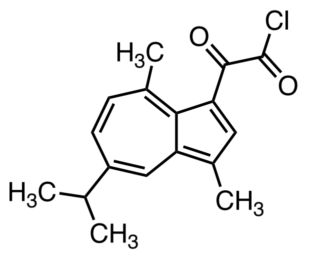

3g-Cl

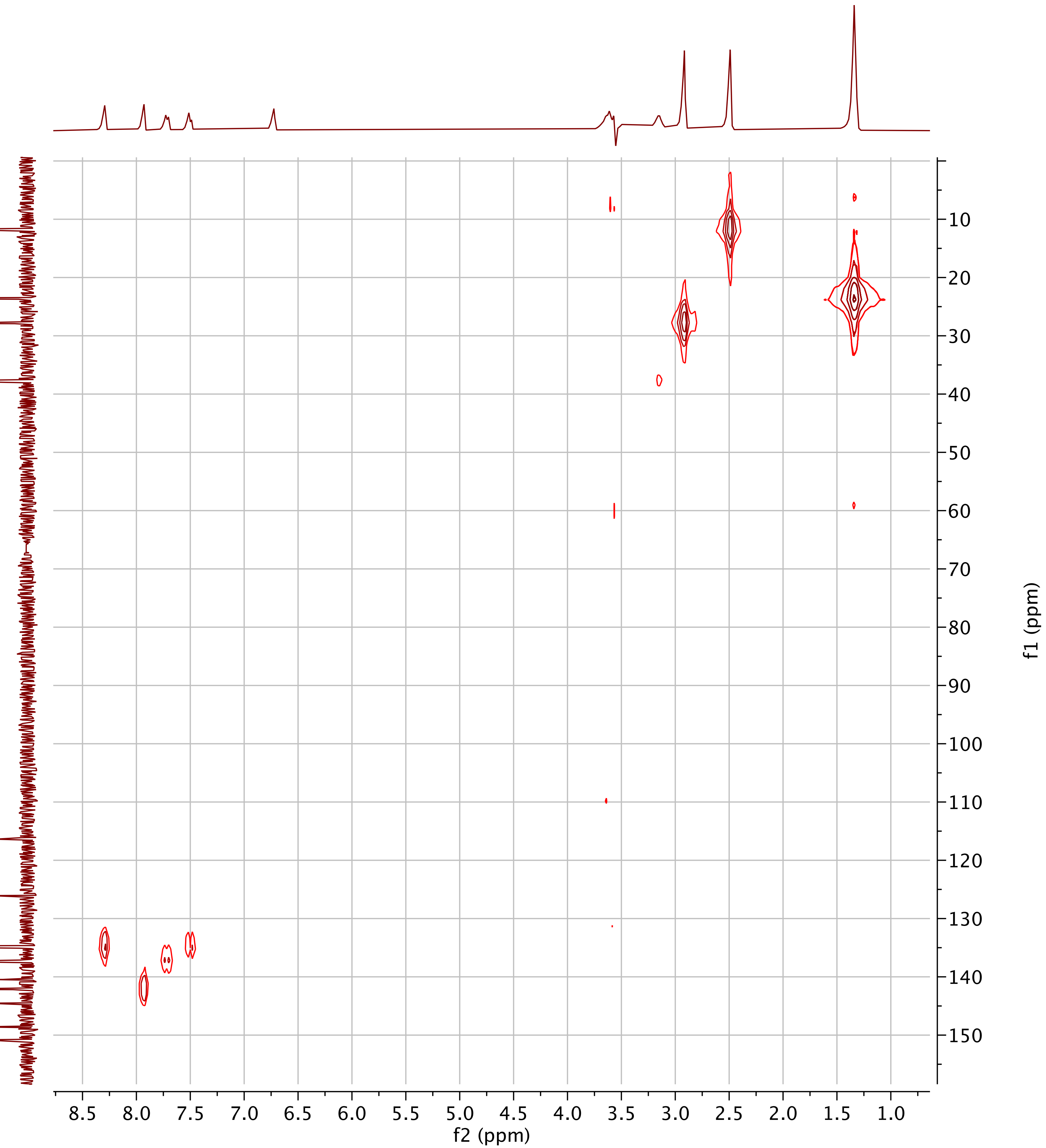

No-D HMQC NMR spectrum of **3g-Cl** in dioxane

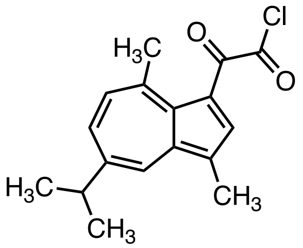

3g-Cl

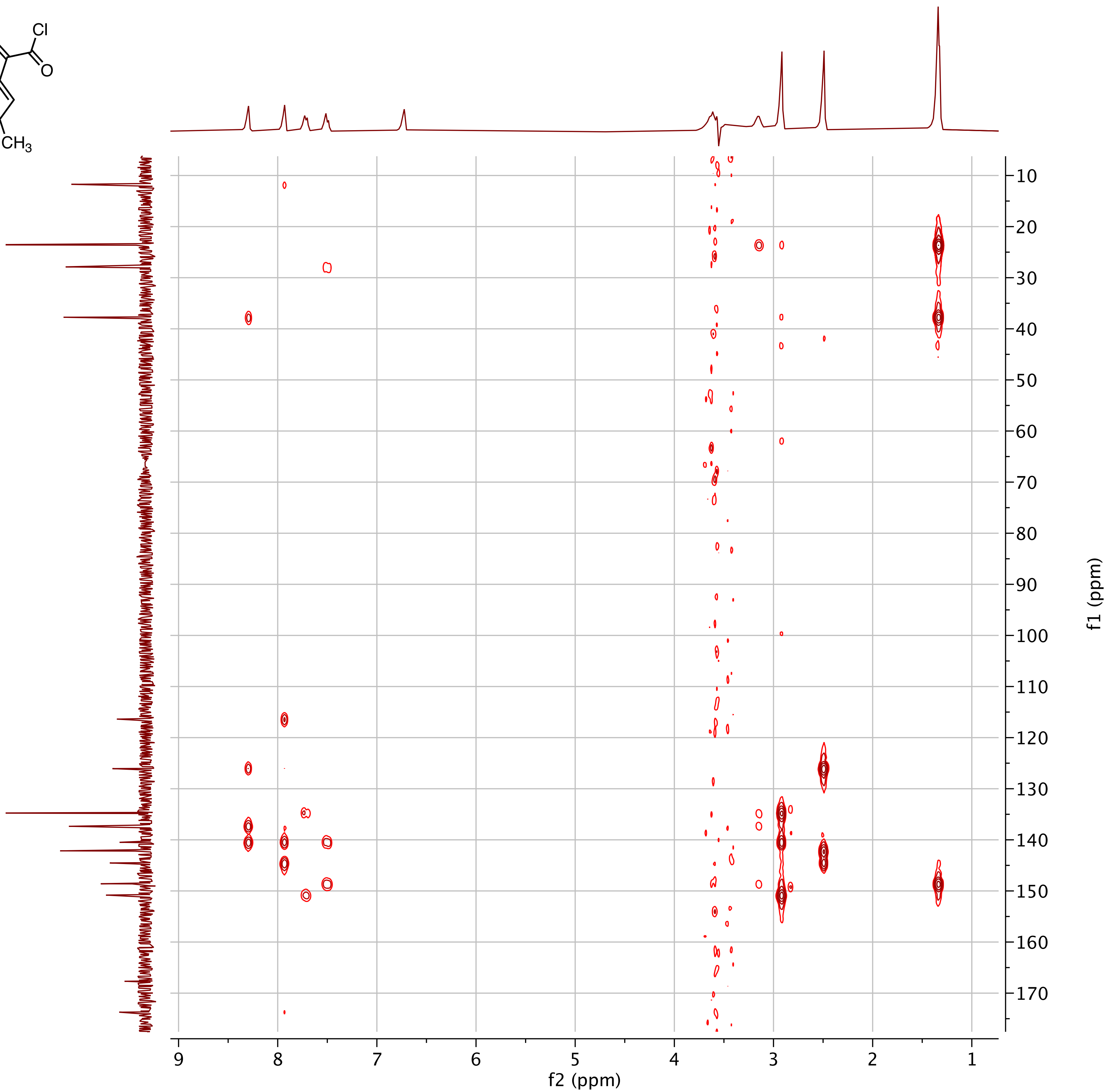

No-D HMBC NMR spectrum of **3g-Cl** in dioxane

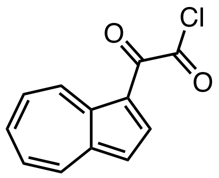

3a-Cl

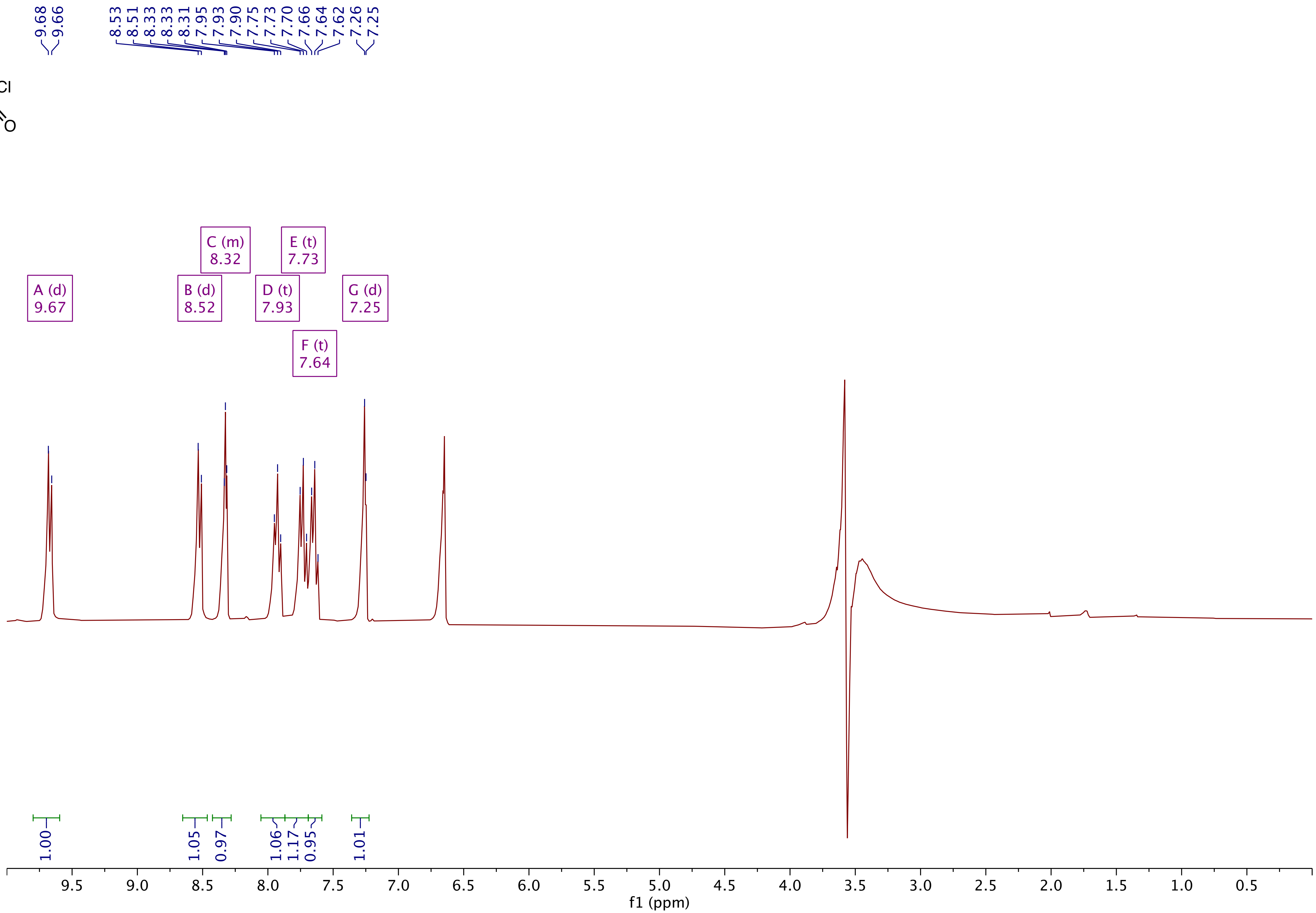

400 MHz no-D ^{1}H-NMR spectrum of **3a-Cl** in dioxane

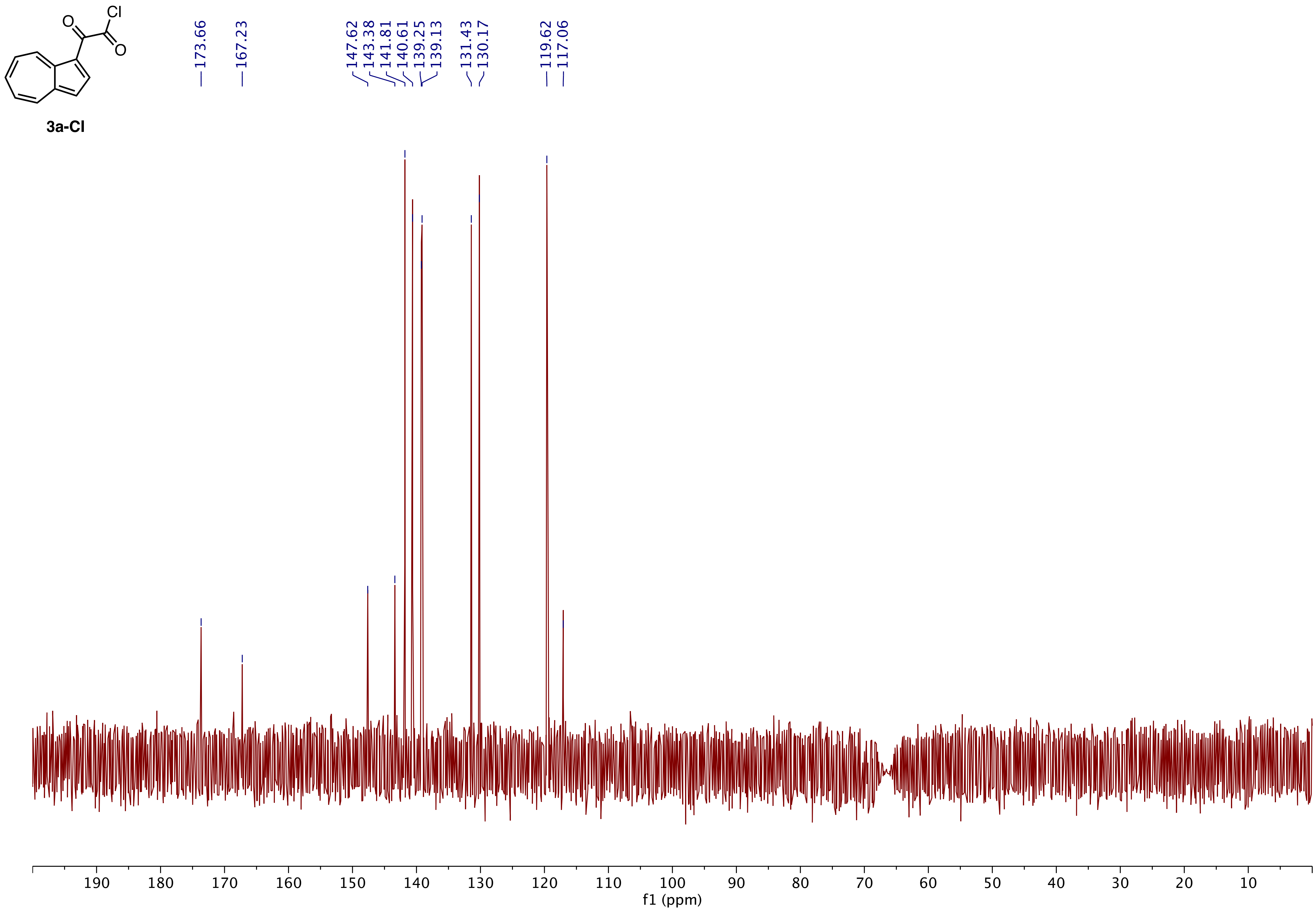

101 MHz no-D $^{13}C\{^{1}H\}$-NMR spectrum of **3a-Cl** in dioxane

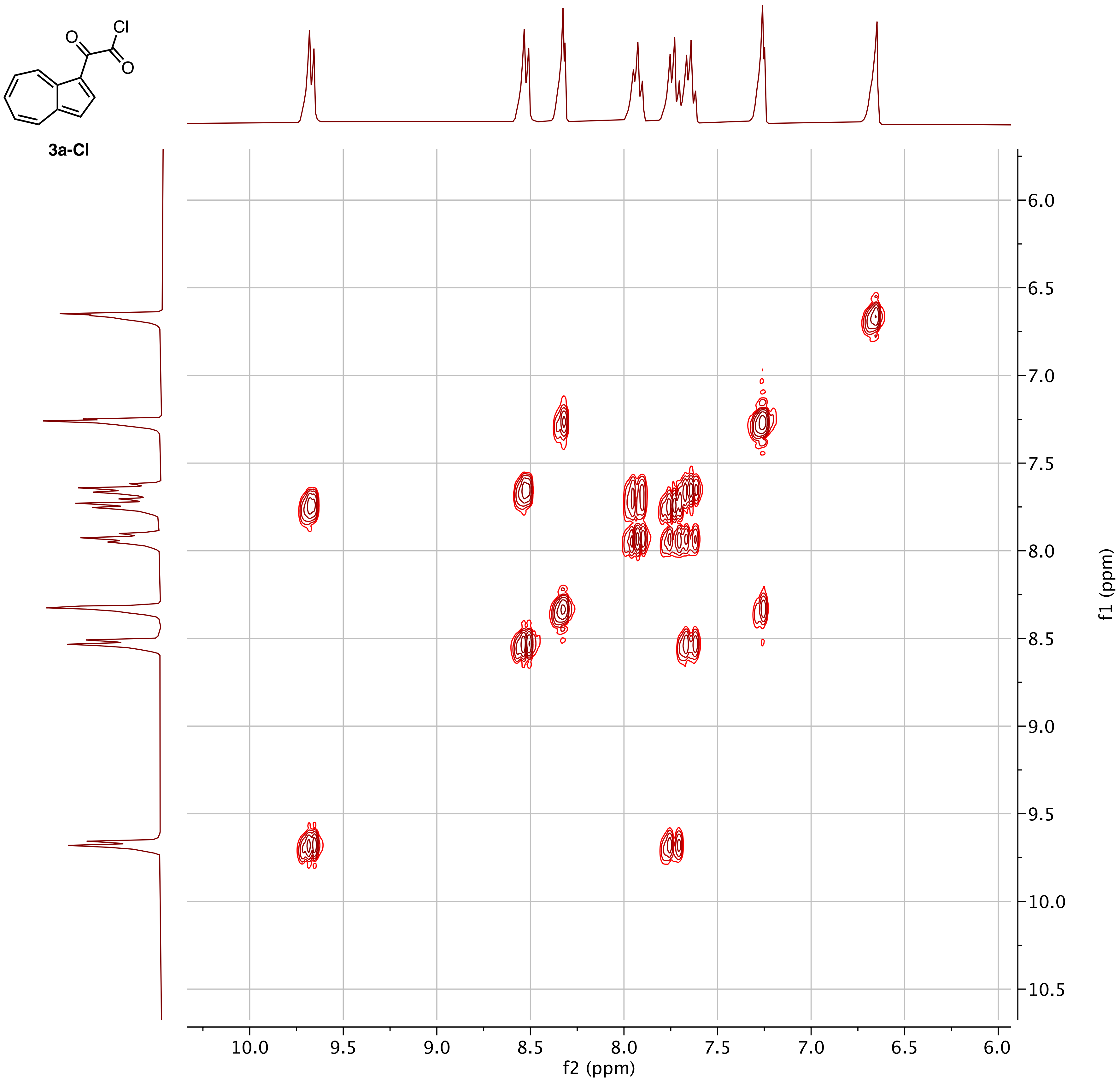

No-D COSY NMR spectrum of **3a-Cl** in dioxane

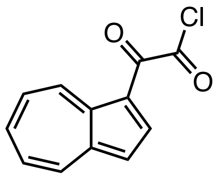

3a-Cl

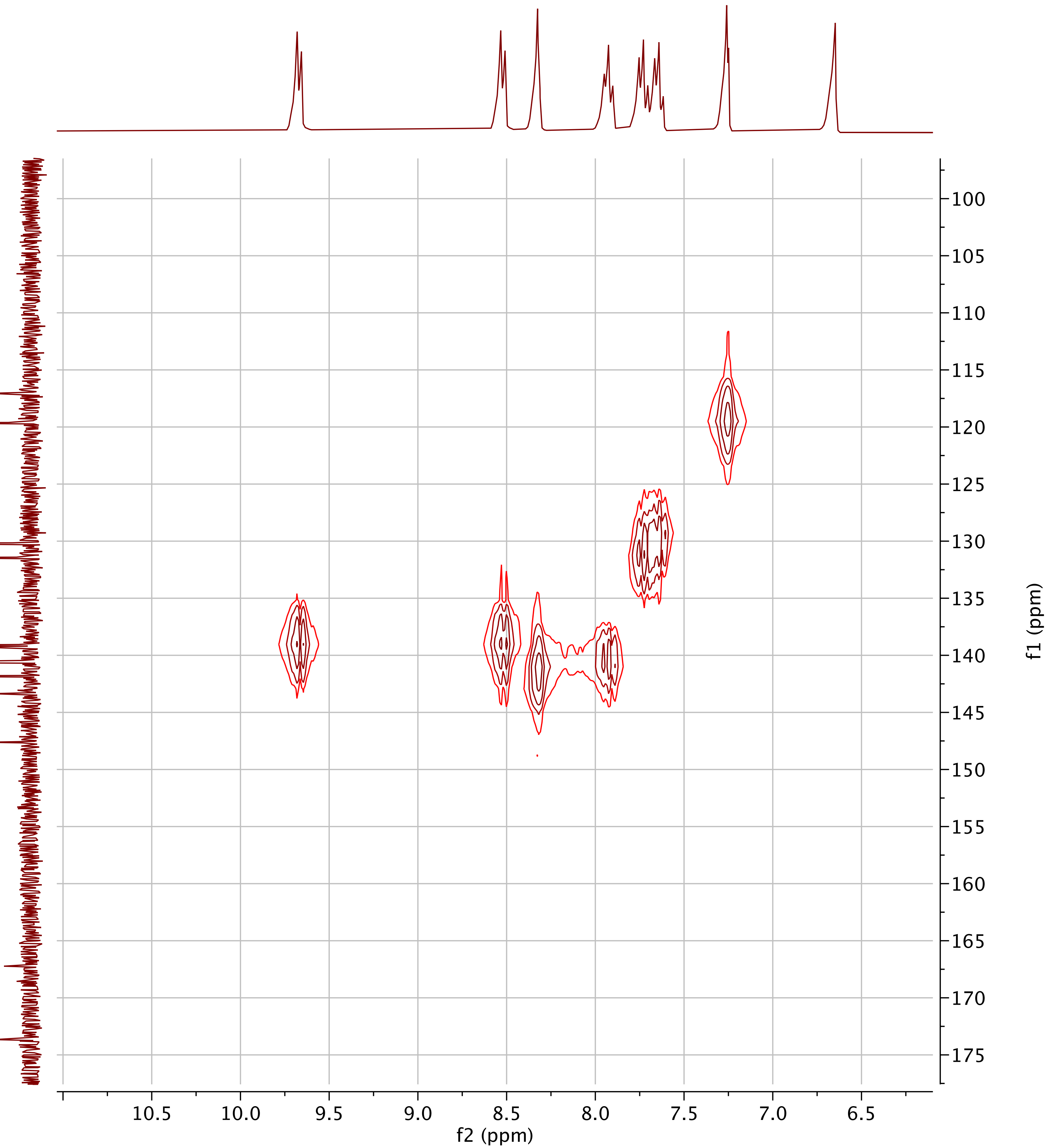

No-D HMQC NMR spectrum of **3a-Cl** in dioxane

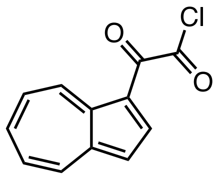

3a-Cl

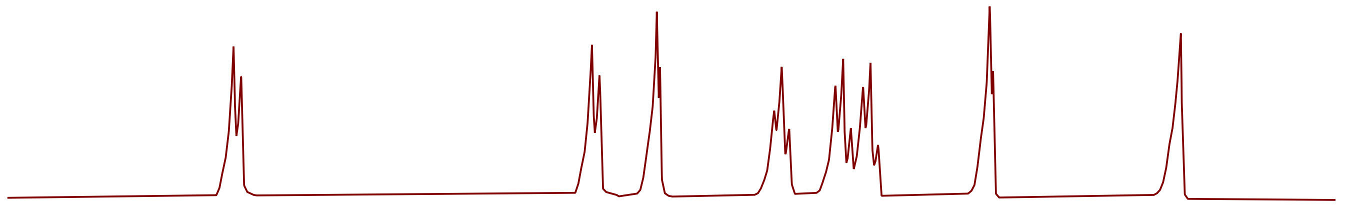

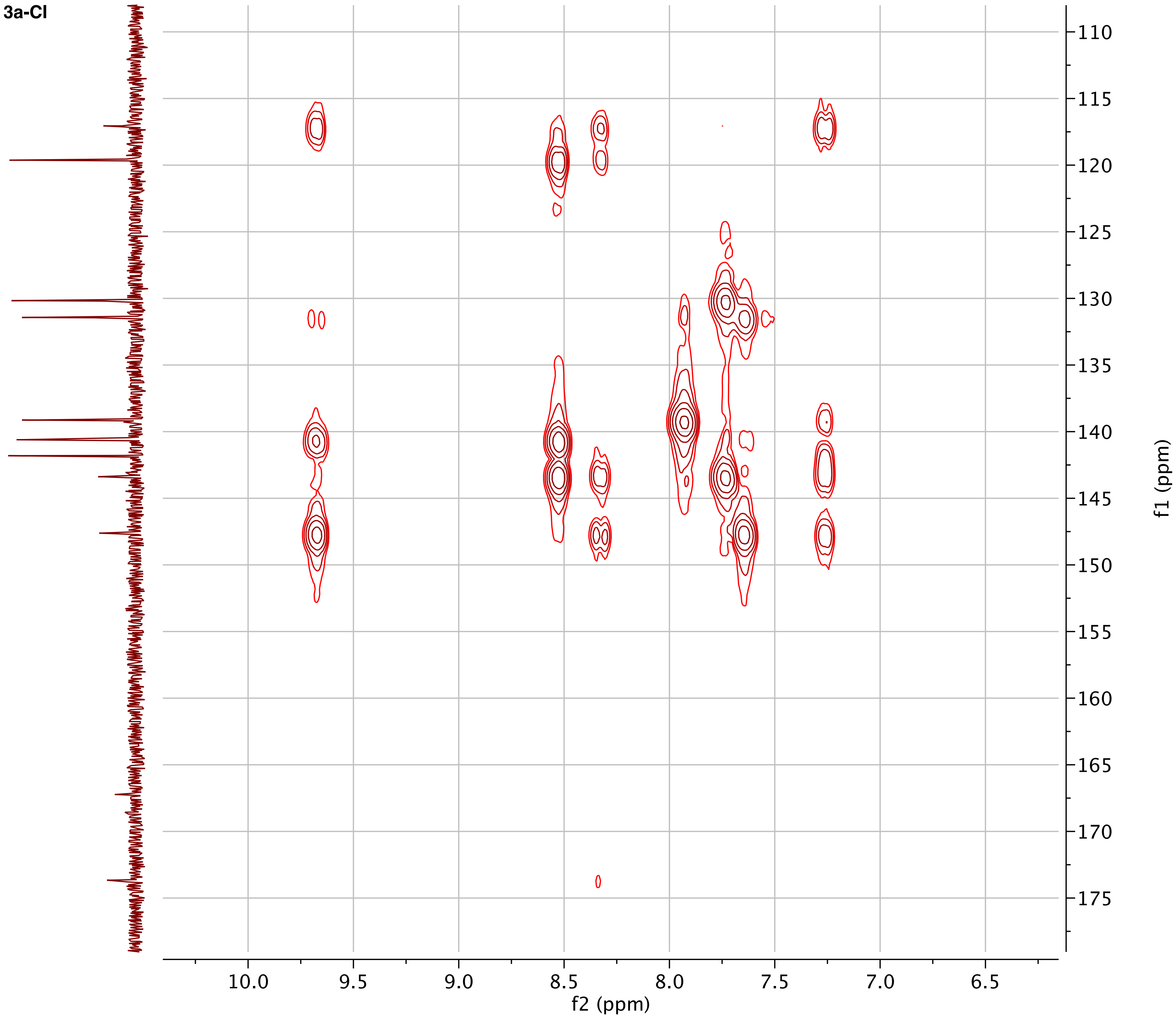

No-D HMBC NMR spectrum of **3a-Cl** in dioxane

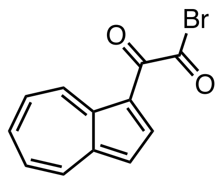

3a-Br

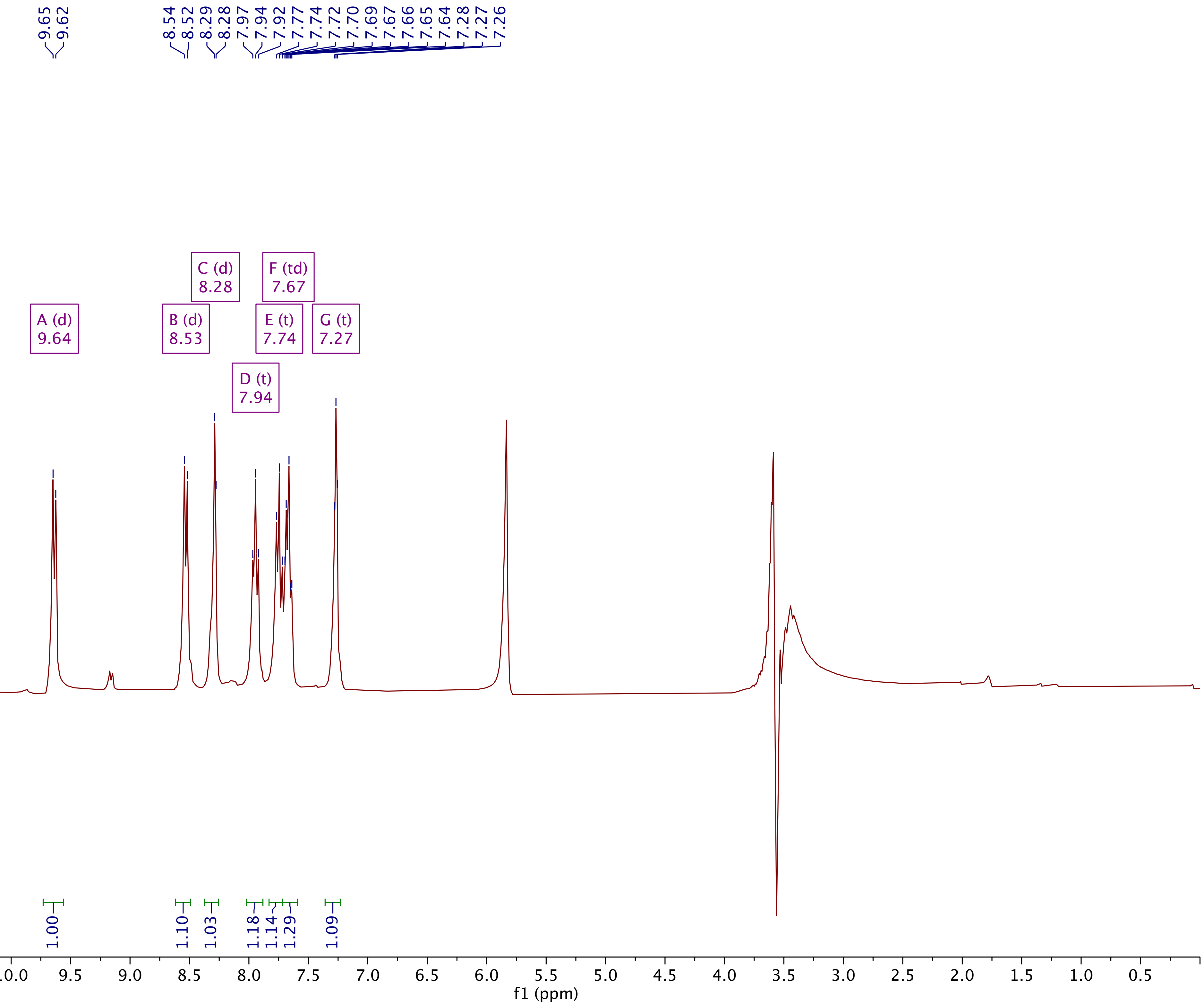

400 MHz no-D ^{1}H-NMR spectrum of **3a-Br** in dioxane

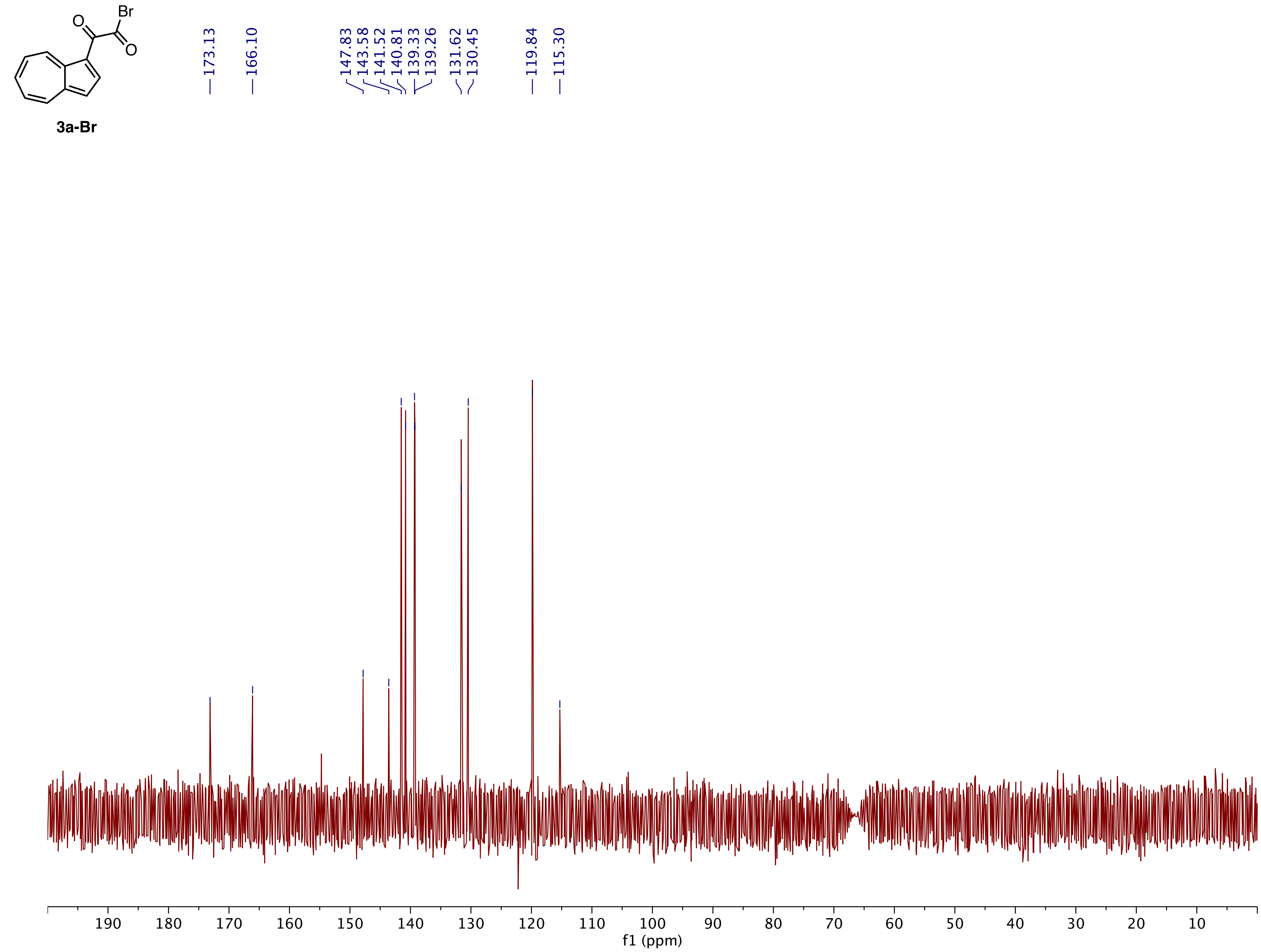

101 MHz no-D $^{13}C\{^{1}H\}$-NMR spectrum of **3a-Br** in dioxane

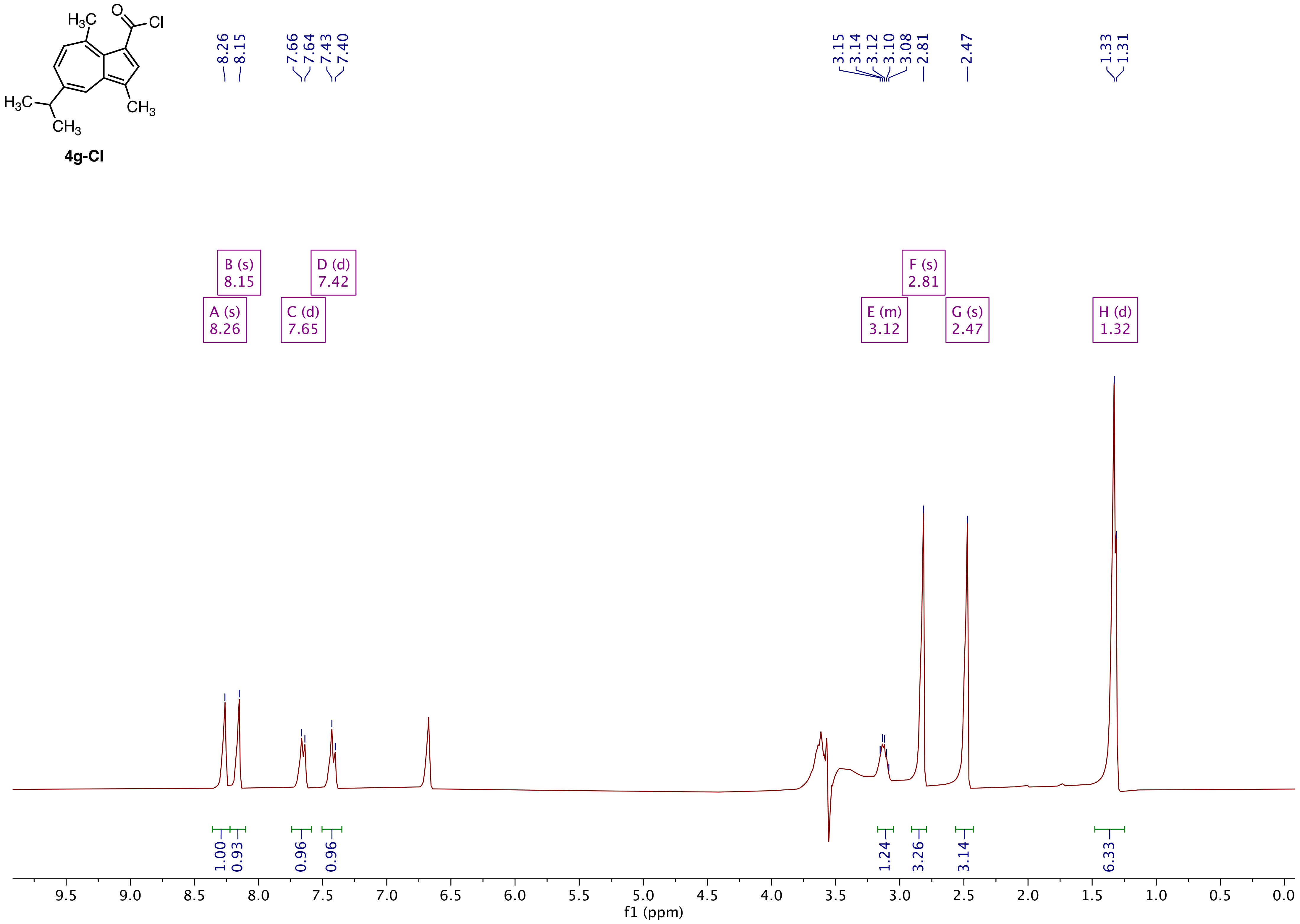

400 MHz no-D ^{1}H-NMR spectrum of **4g-Cl** in dioxane

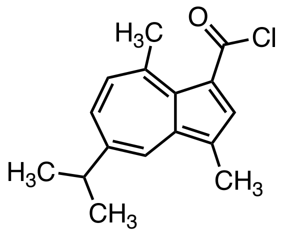

4g-Cl

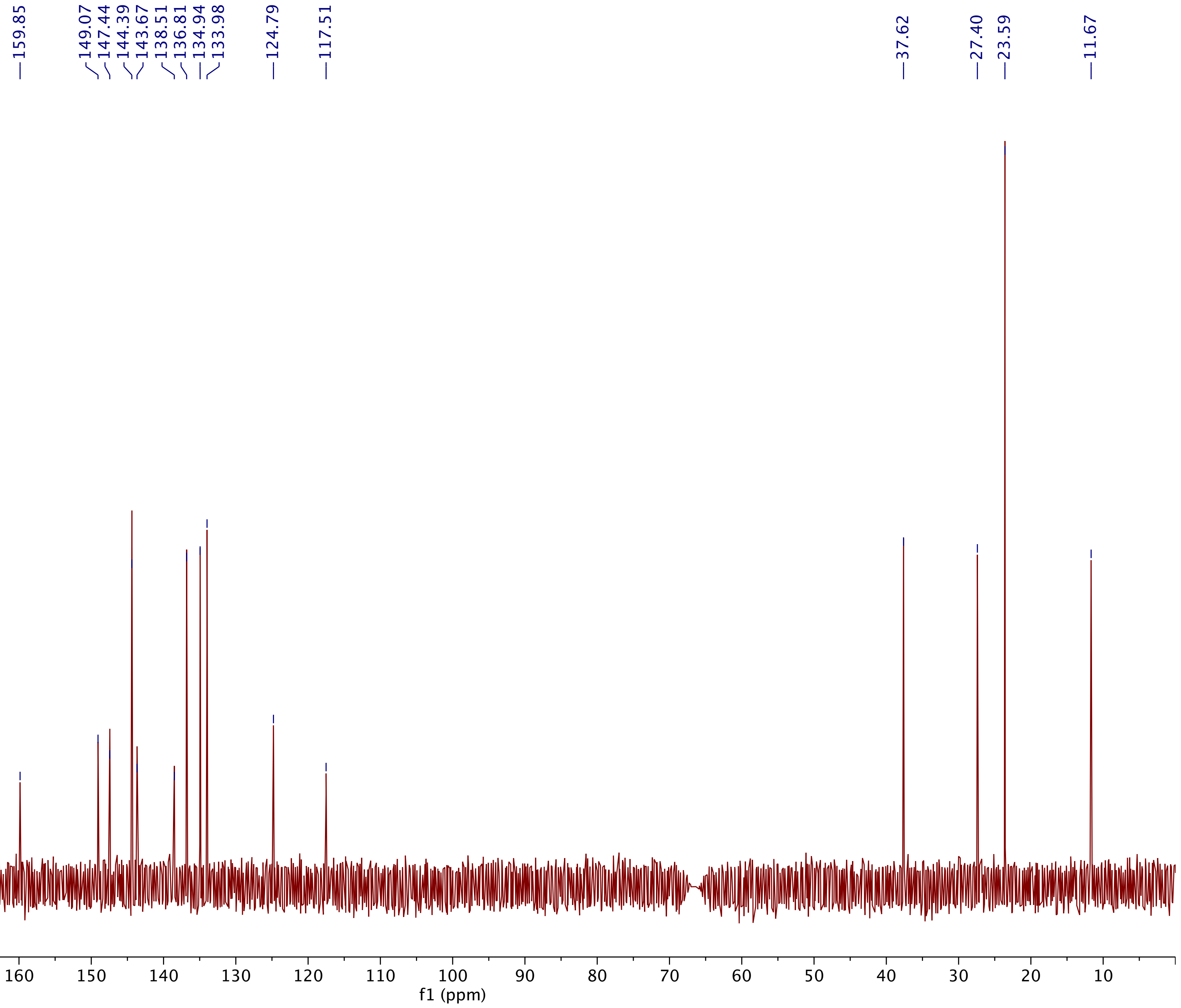

101 MHz no-D $^{13}C\{^{1}H\}$-NMR spectrum of **4g-Cl** in dioxane

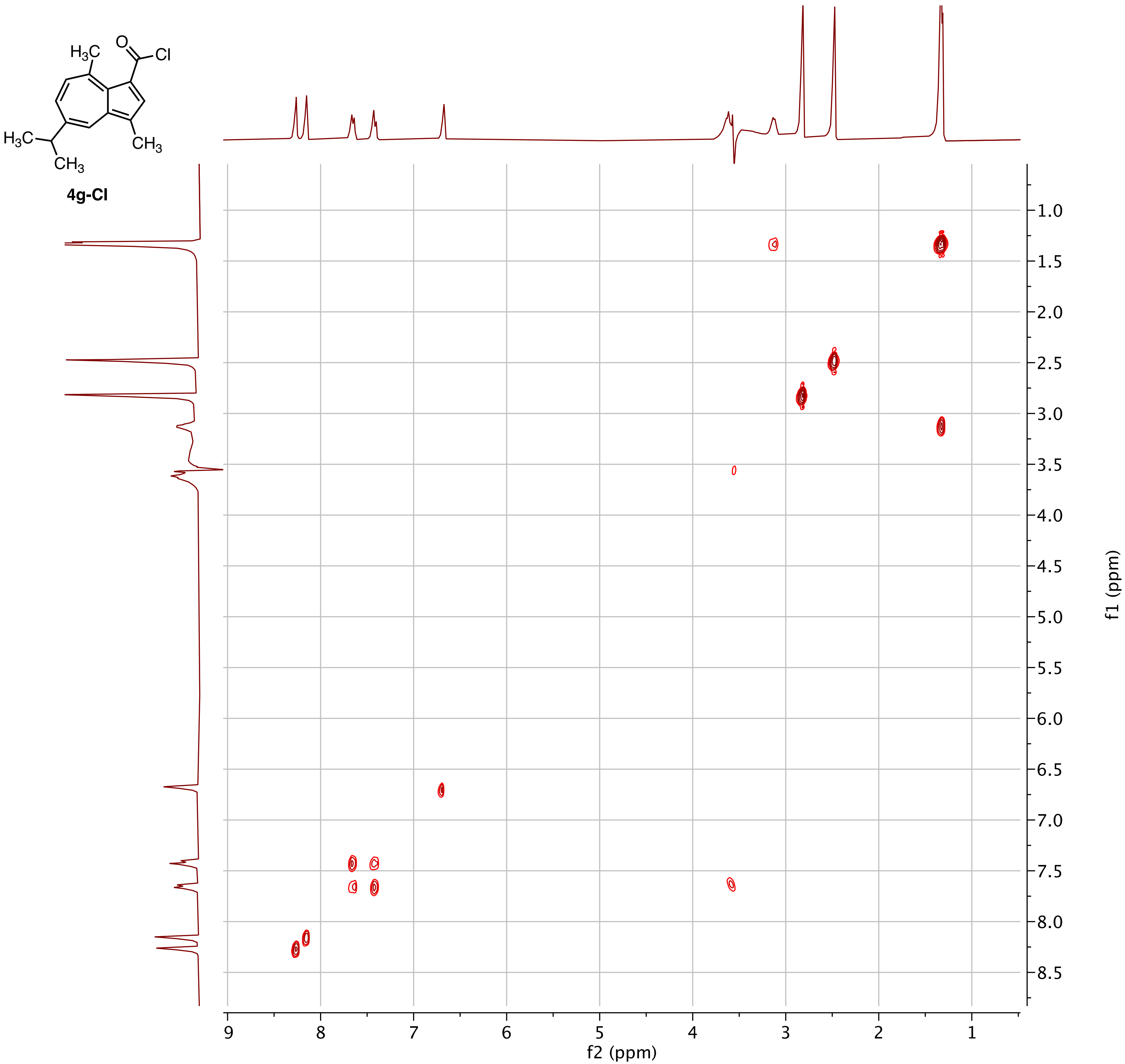

No-D COSY NMR spectrum of **4g-Cl** in dioxane

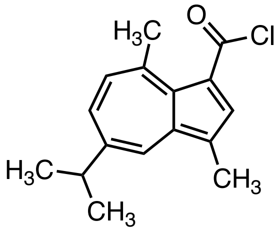

4g-Cl

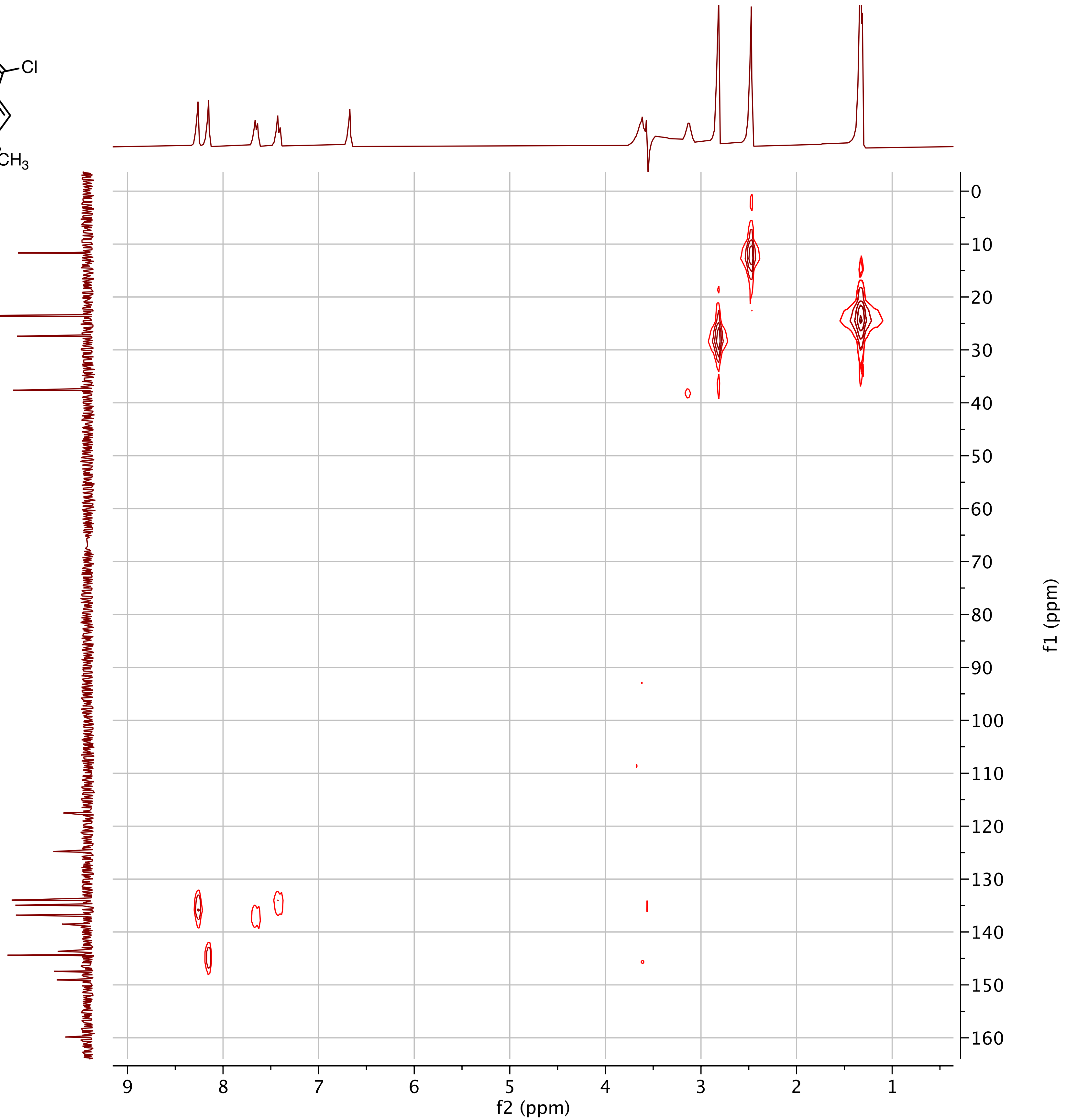

No-D HMQC NMR spectrum of **4g-Cl** in dioxane

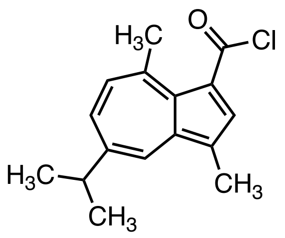

4g-Cl

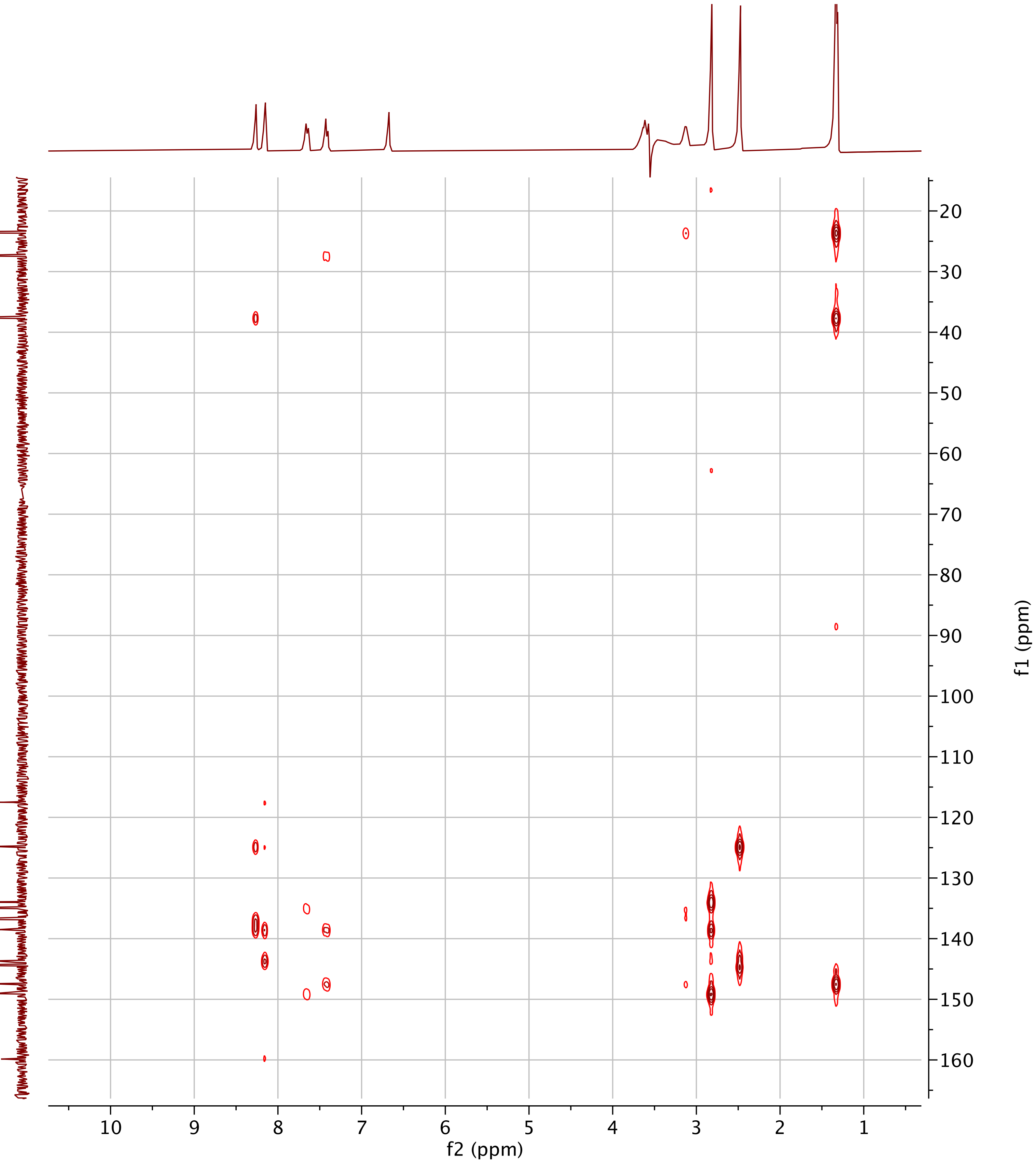

No-D HMBC NMR spectrum of **4g-Cl** in dioxane

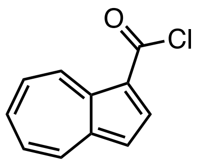

4a-Cl

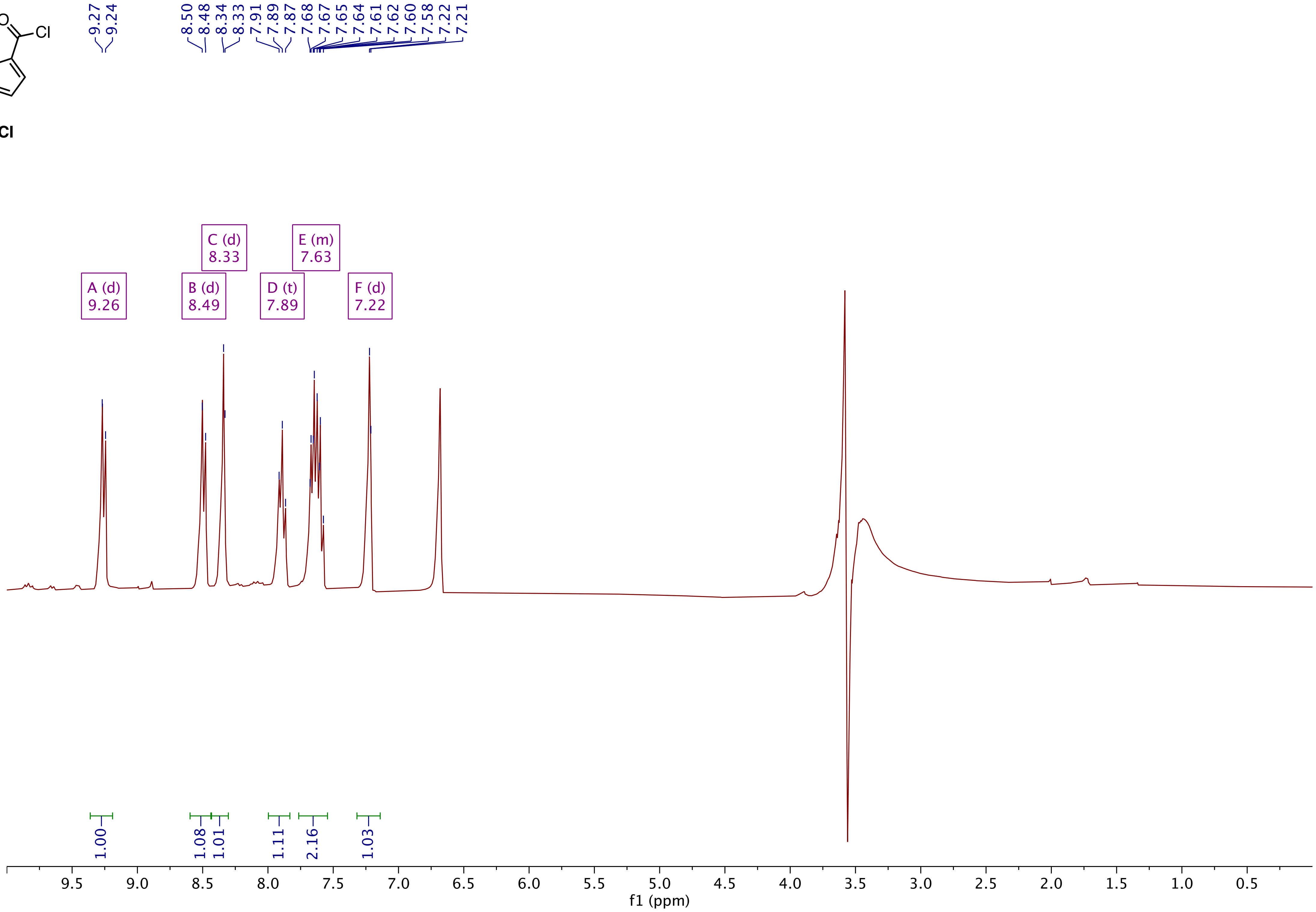

400 MHz no-D ^{1}H-NMR spectrum of **4a-Cl** in dioxane

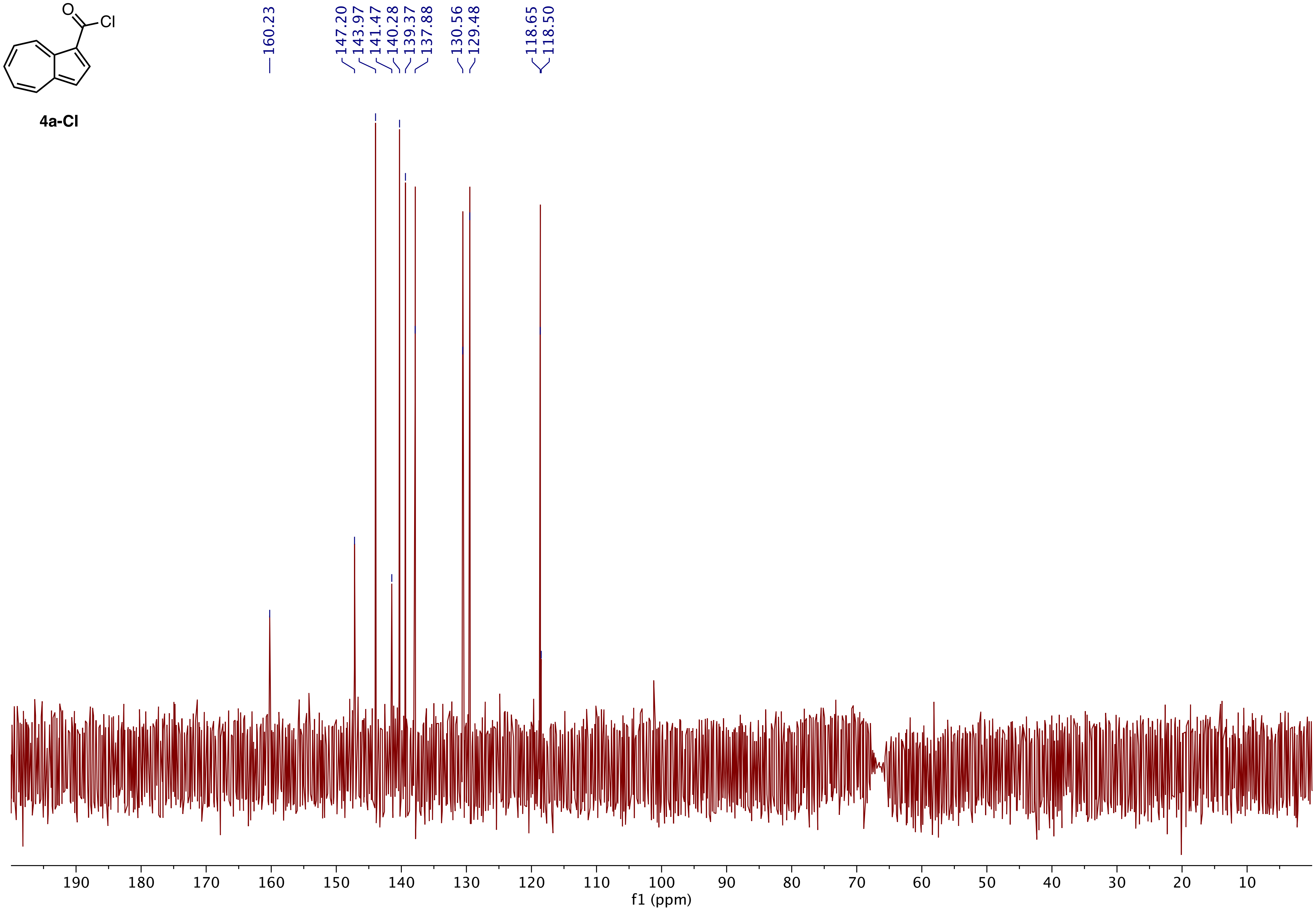

101 MHz no-D $^{13}C\{^1H\}$-NMR spectrum of **4a-Cl** in dioxane

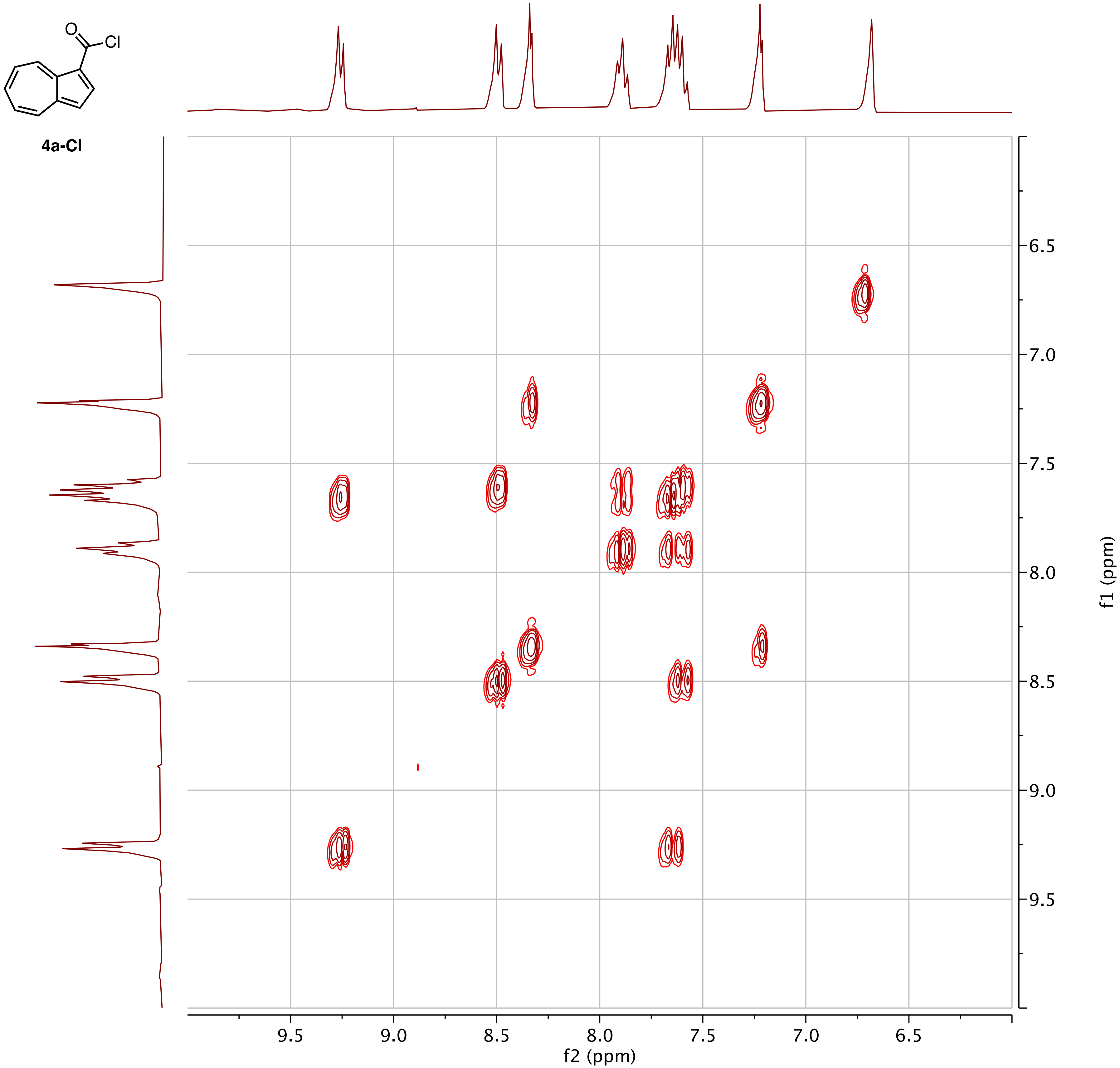

No-D COSY NMR spectrum of **4a-Cl** in dioxane

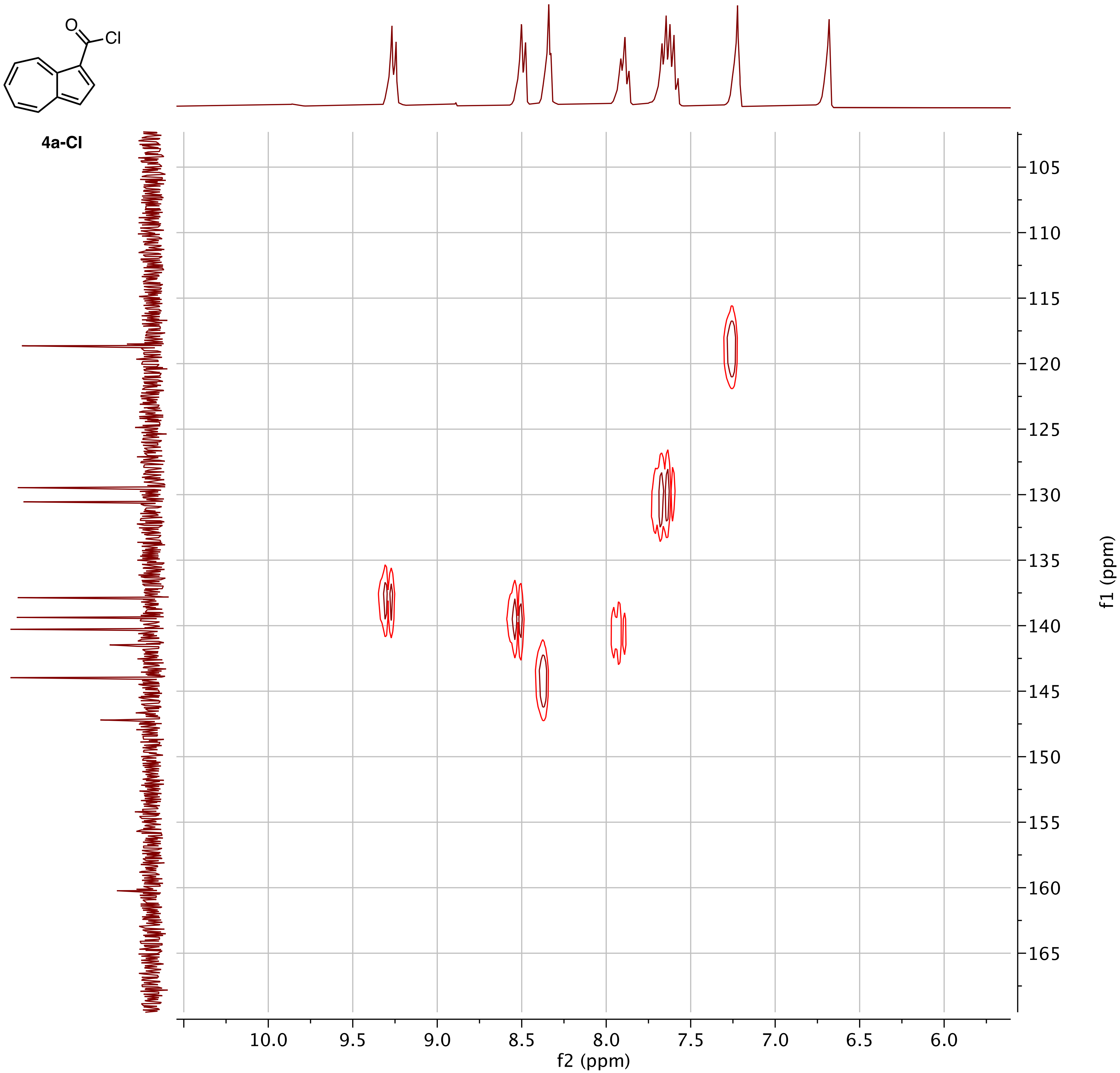

No-D HMQC NMR spectrum of **4a-Cl** in dioxane

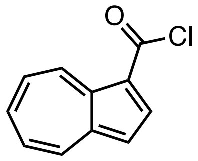

4a-Cl

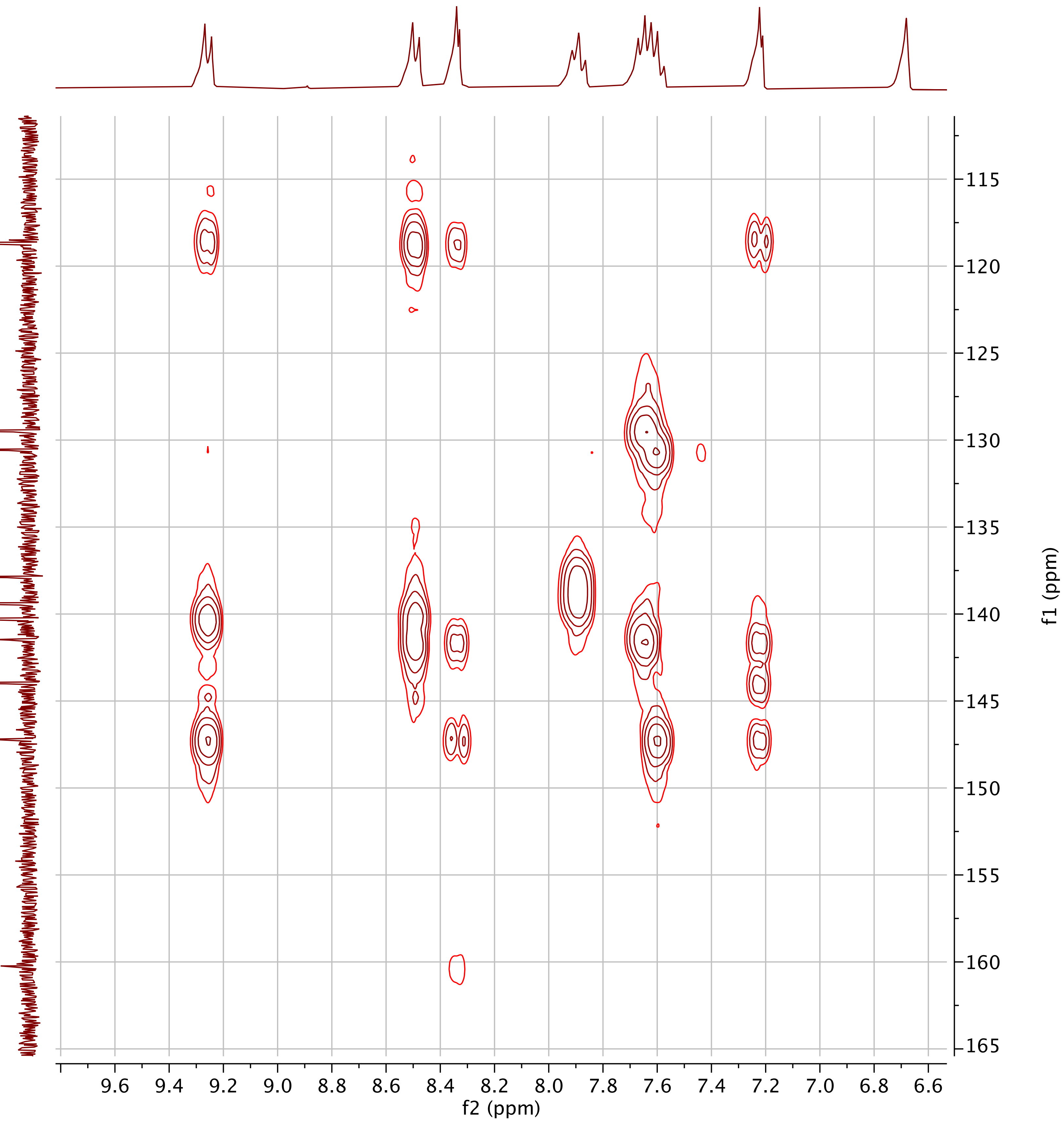

No-D HMBC NMR spectrum of **4a-Cl** in dioxane

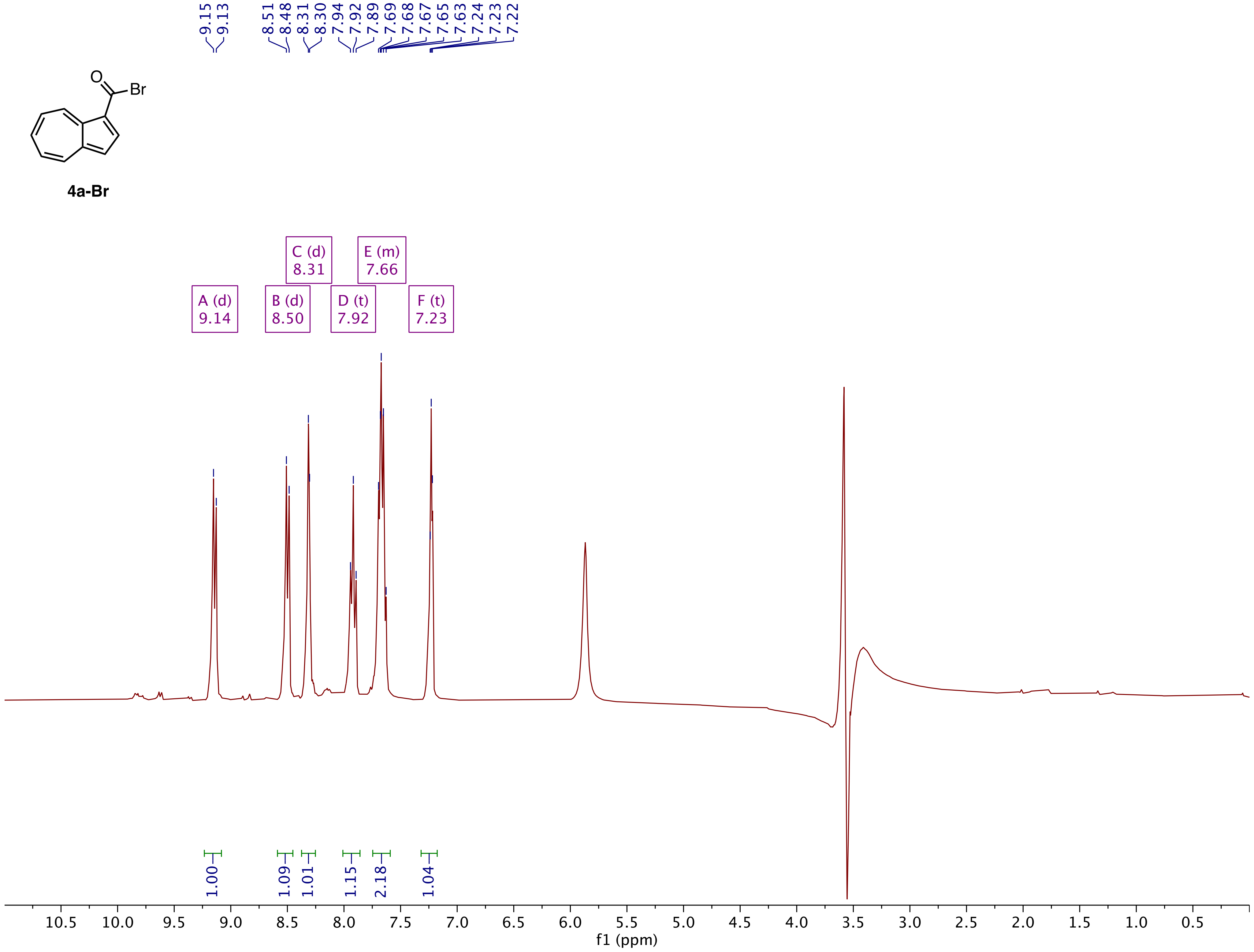

400 MHz no-D ^{1}H-NMR spectrum of **4a-Br** in dioxane

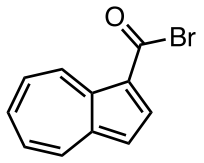

4a-Br

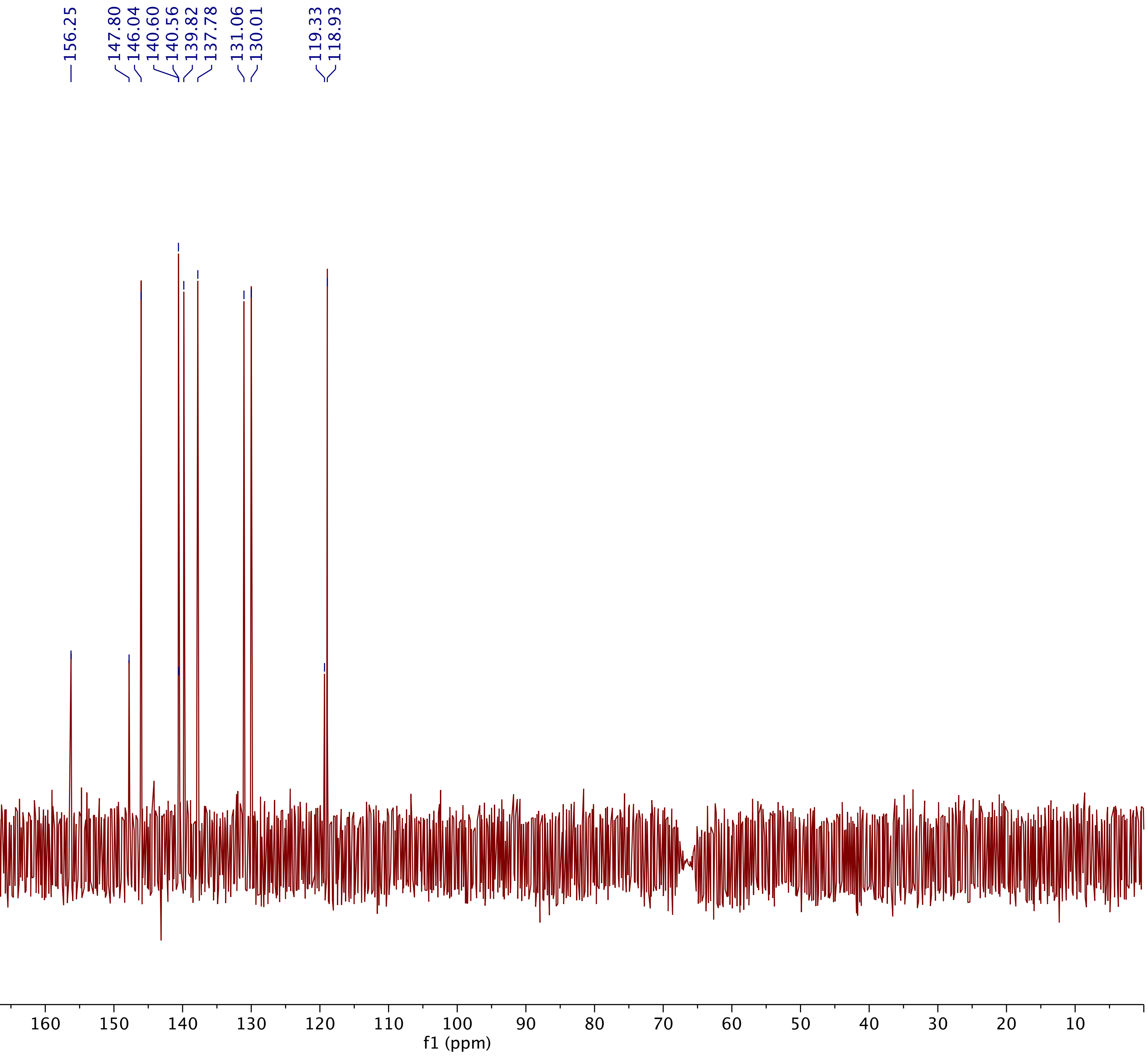

101 MHz no-D $^{13}C\{^{1}H\}$-NMR spectrum of **4a-Br** in dioxane

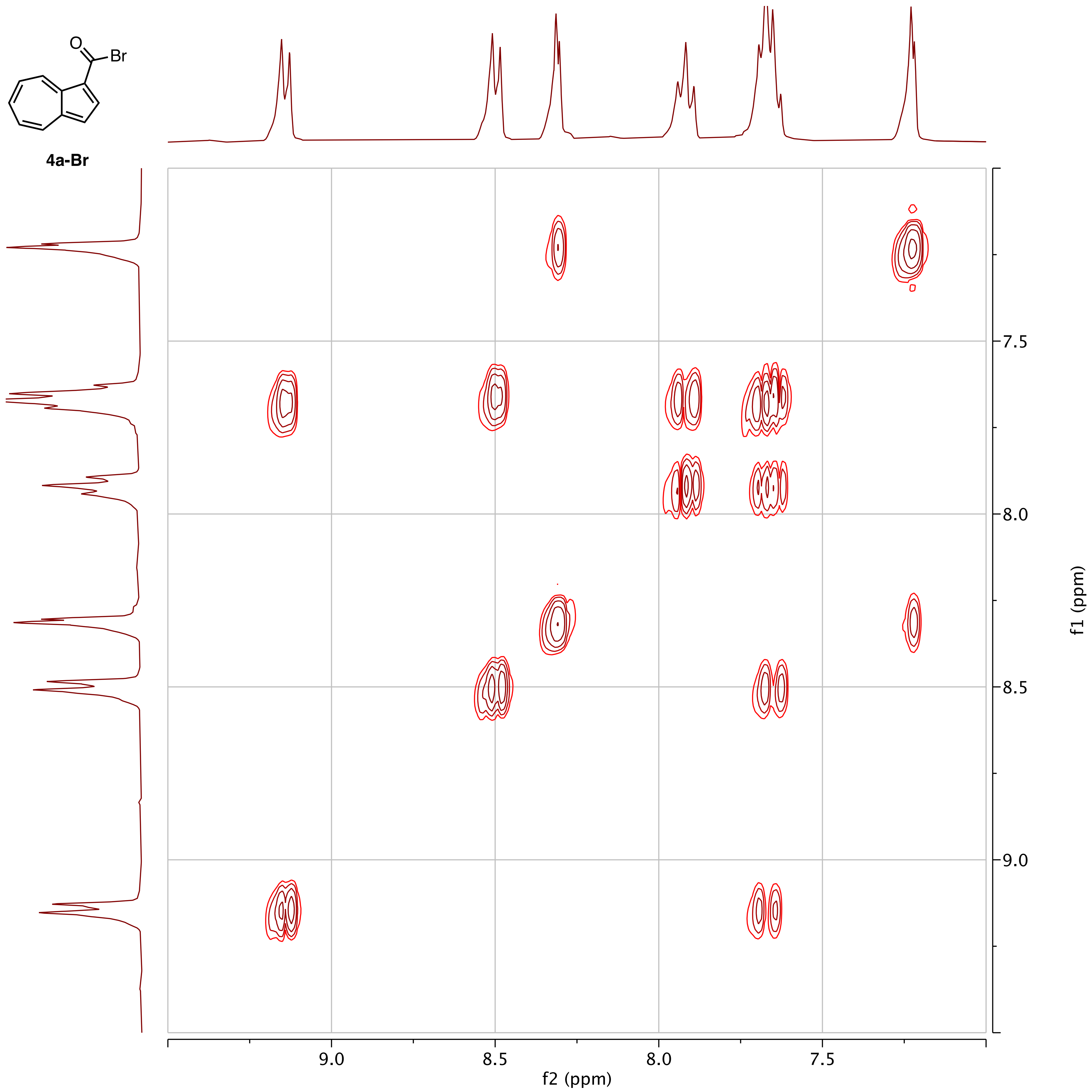

No-D COSY NMR spectrum of **4a-Br** in dioxane

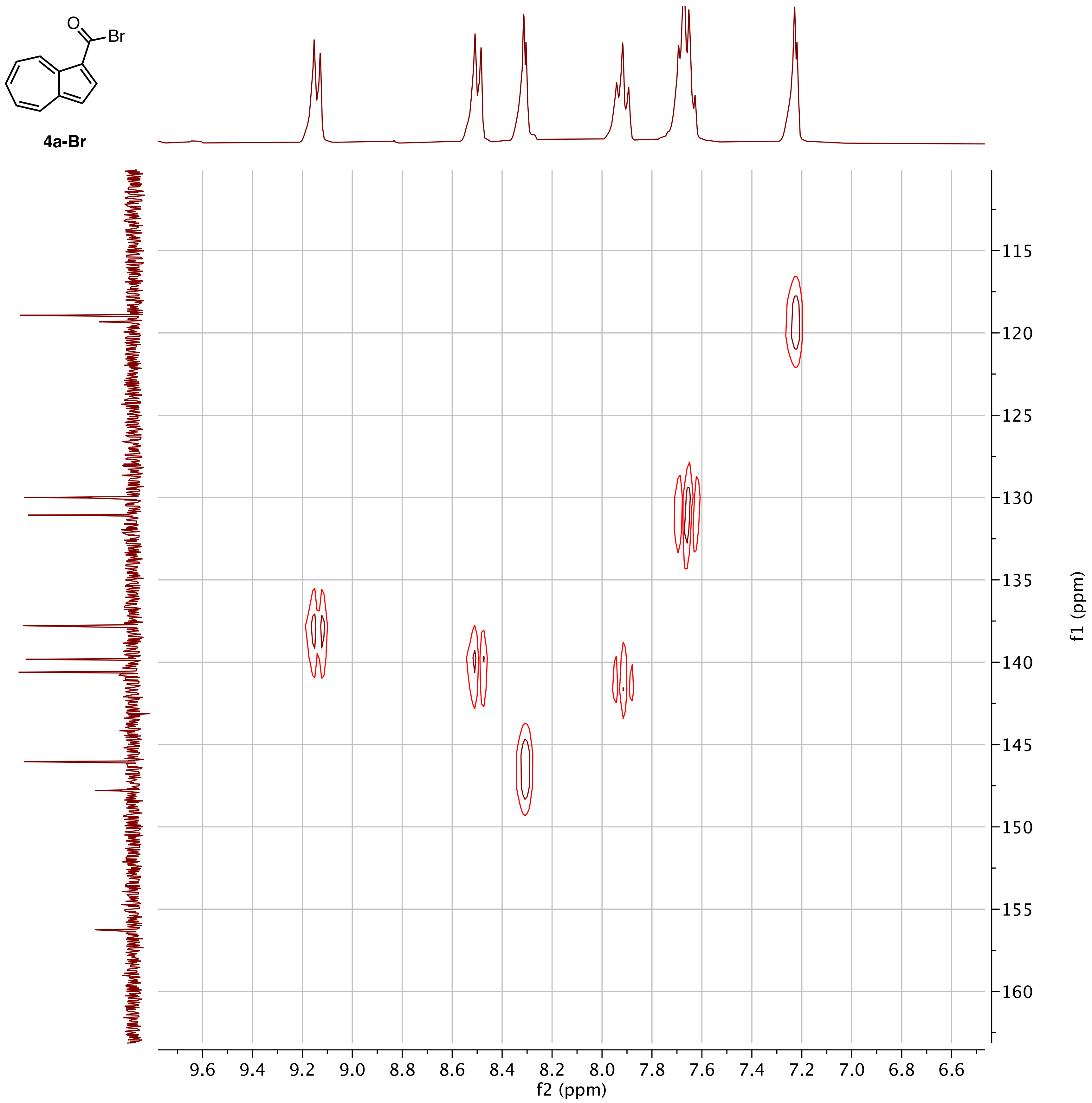

No-D HMQC NMR spectrum of **4a-Br** in dioxane

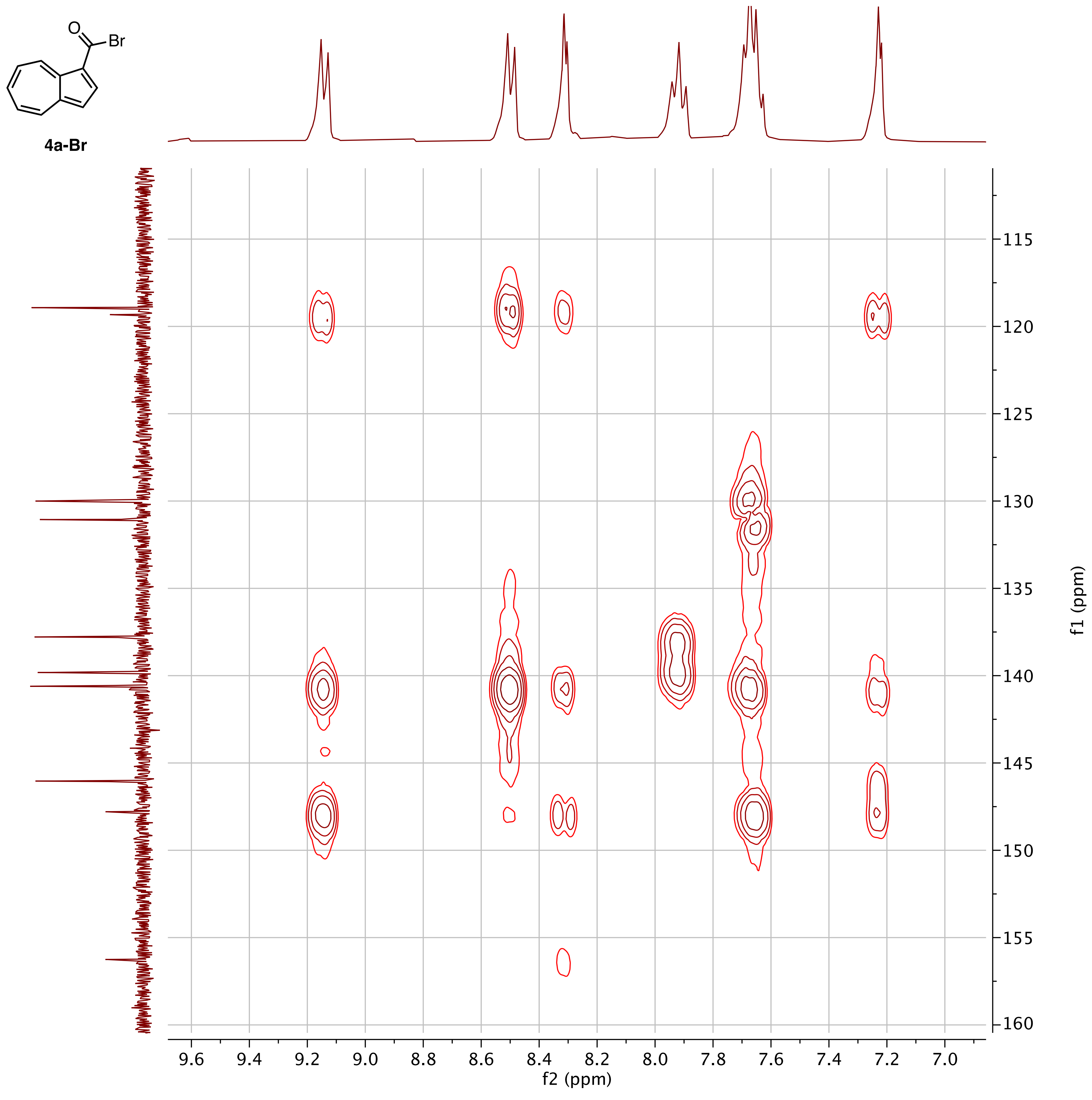

No-D HMBC NMR spectrum of **4a-Br** in dioxane

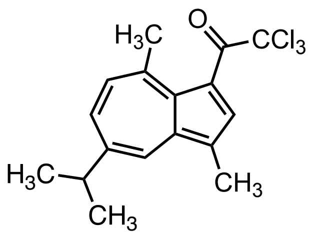

5

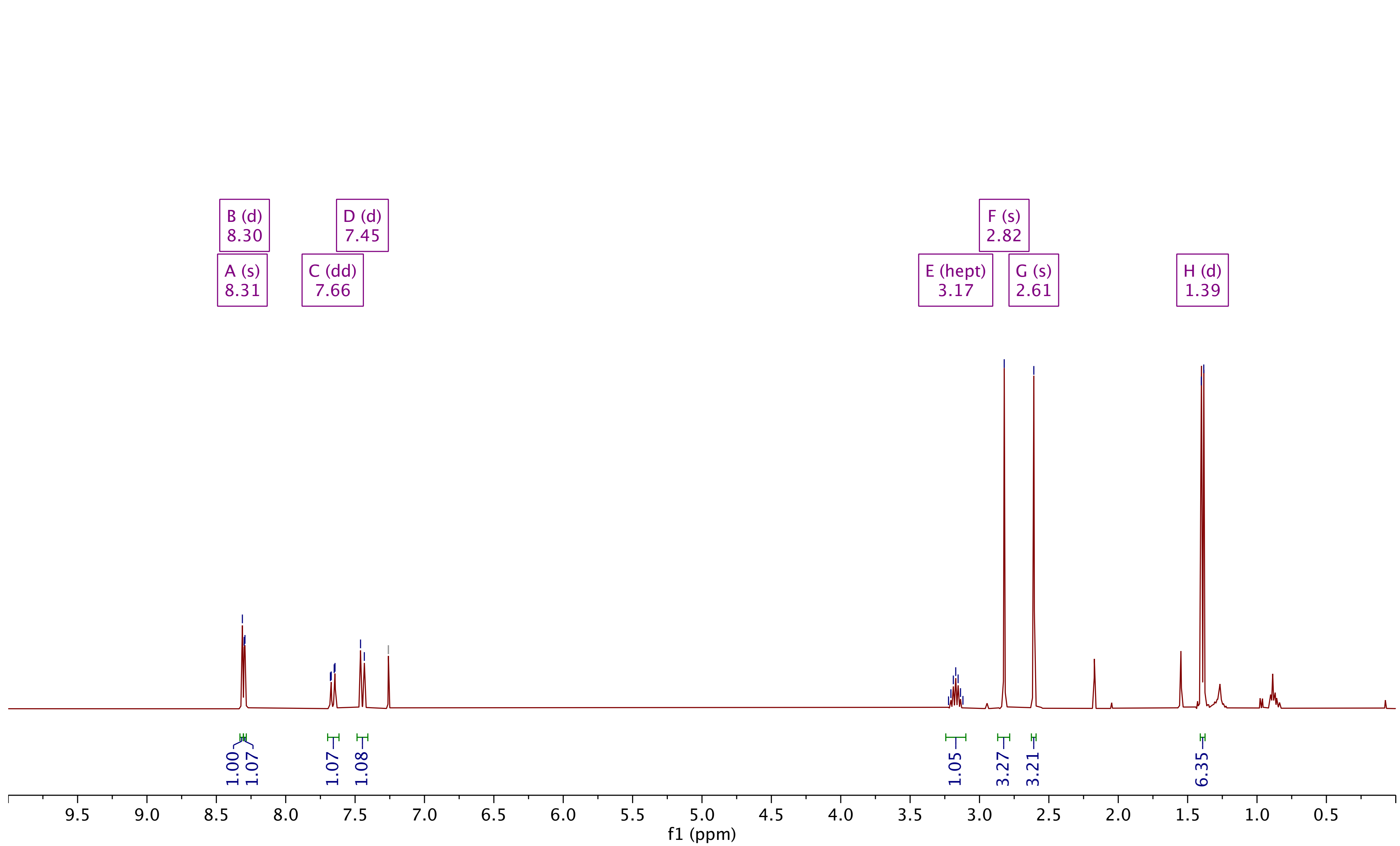

400 MHz ^{1}H-NMR spectrum of **5** in $CDCl_3$

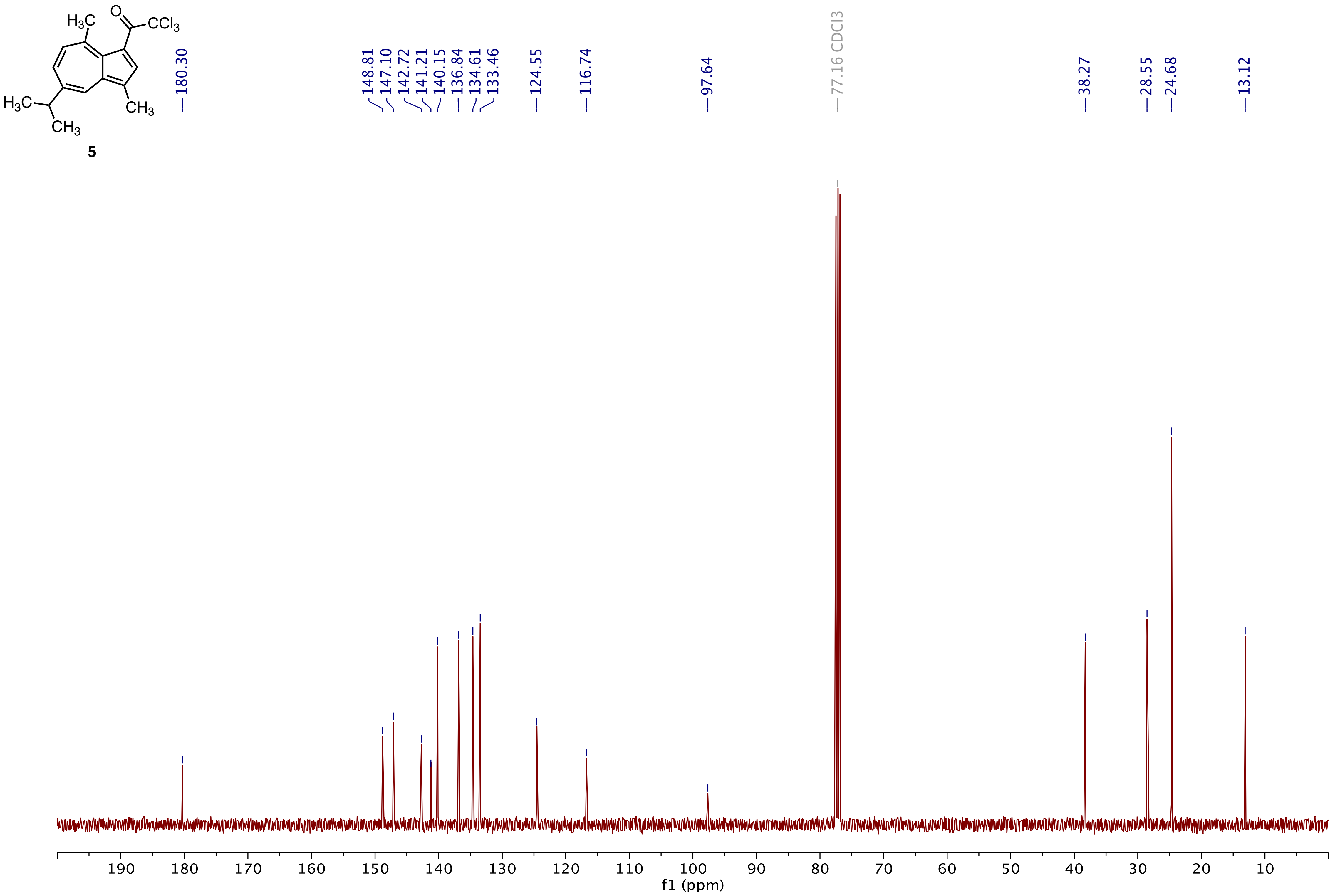

101 MHz $^{13}C\{^{1}H\}$-NMR spectrum of **5** in $CDCl_3$

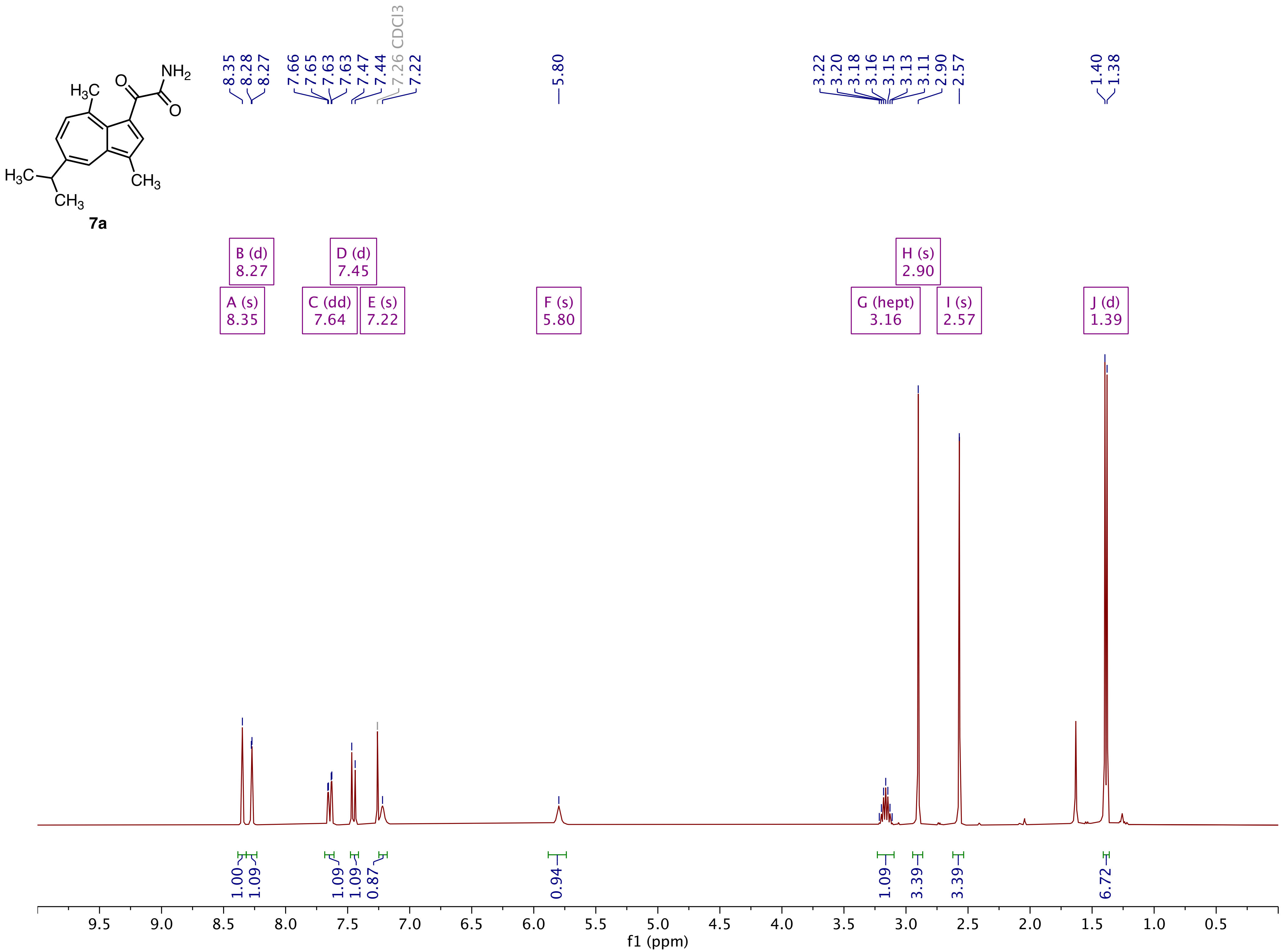

400 MHz ^{1}H-NMR spectrum of **7a** in $CDCl_3$

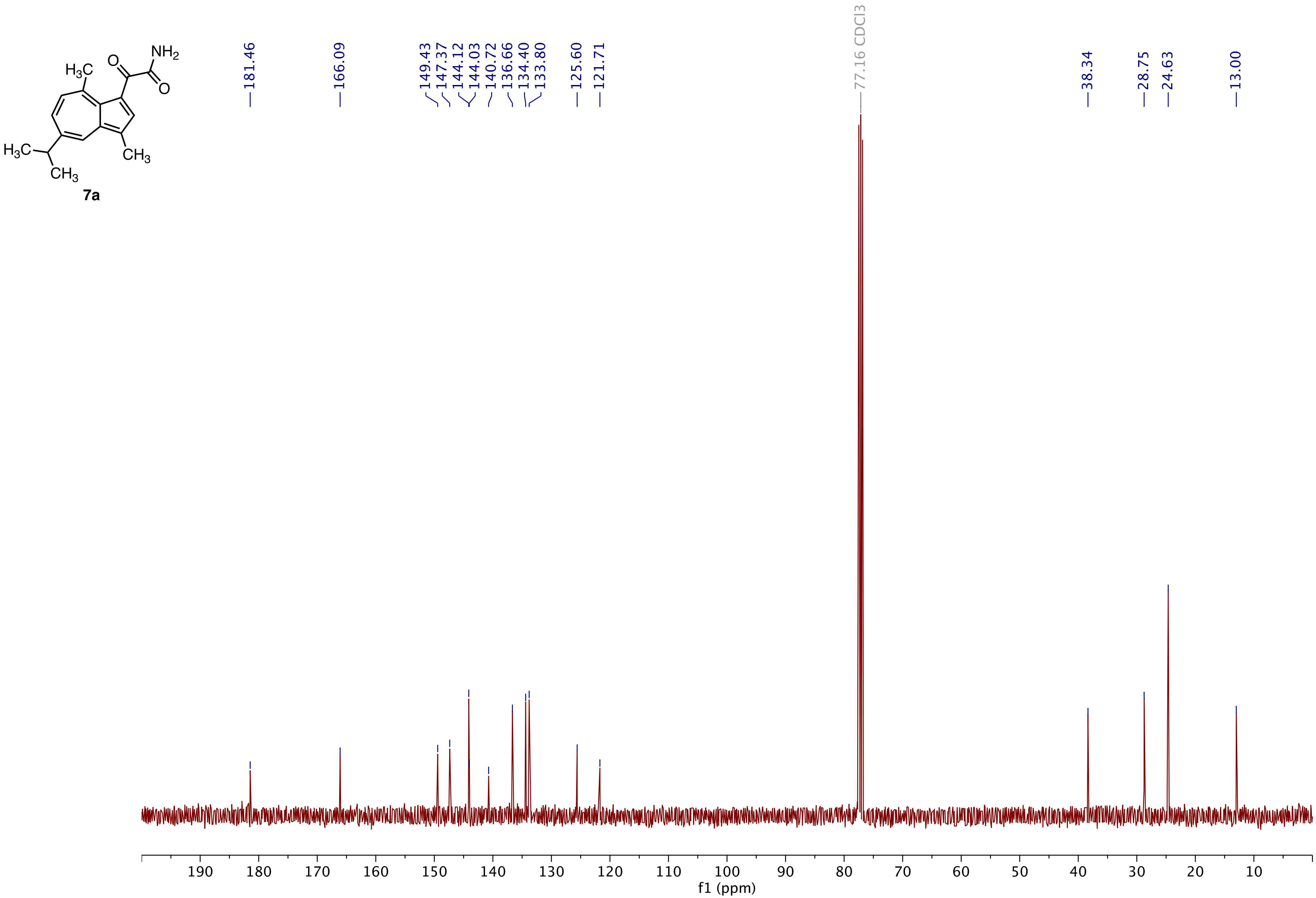

101 MHz $^{13}C\{^{1}H\}$-NMR spectrum of **7a** in $CDCl_3$

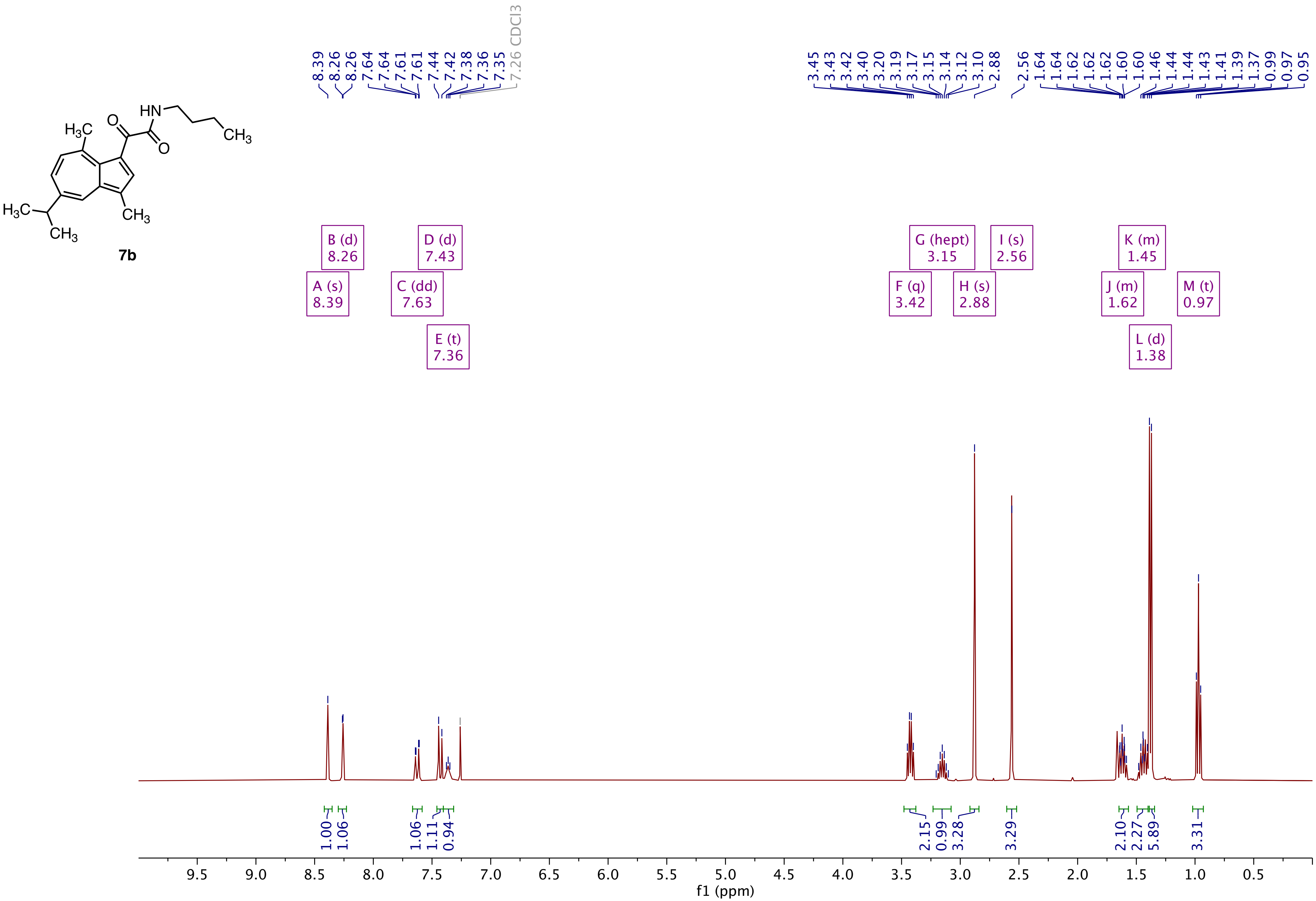

400 MHz ^{1}H-NMR spectrum of **7b** in $CDCl_3$

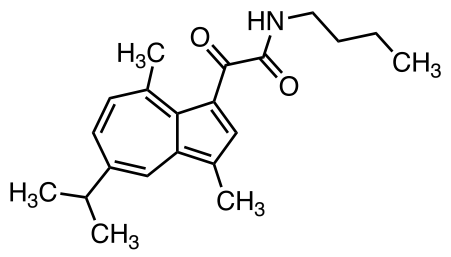

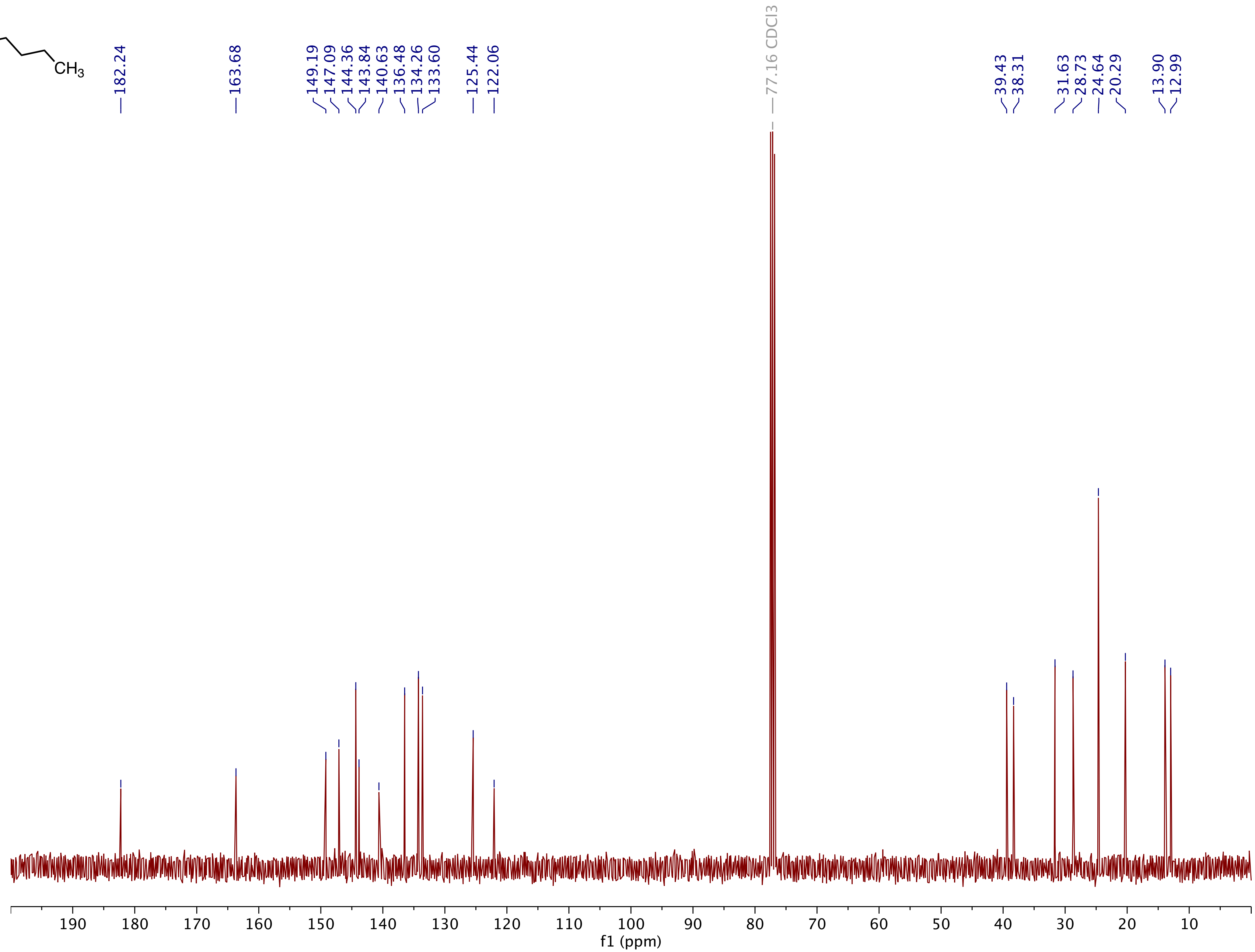

101 MHz $^{13}C\{^{1}H\}$-NMR spectrum of **7b** in $CDCl_3$

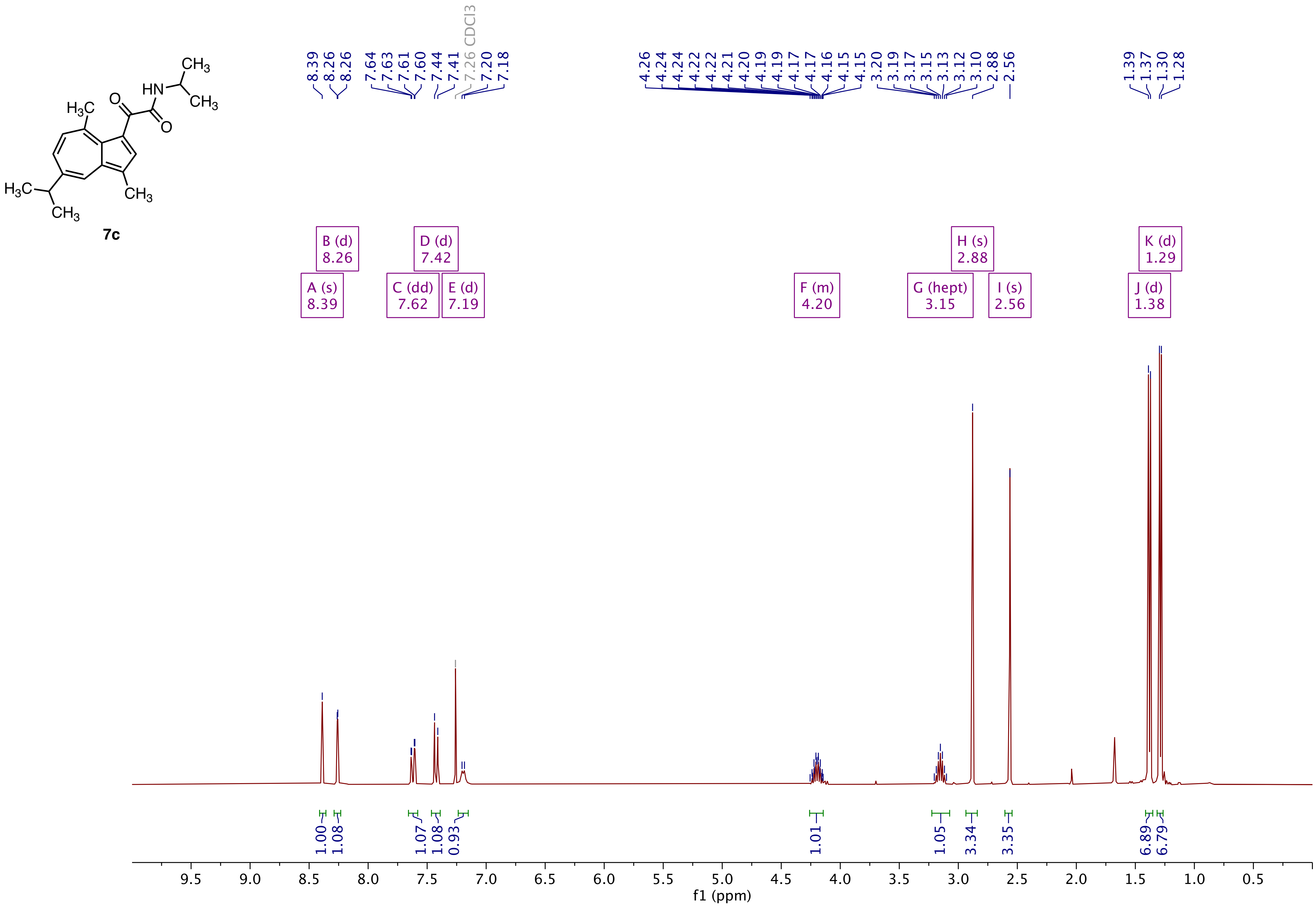

400 MHz ^{1}H-NMR spectrum of **7c** in $CDCl_3$

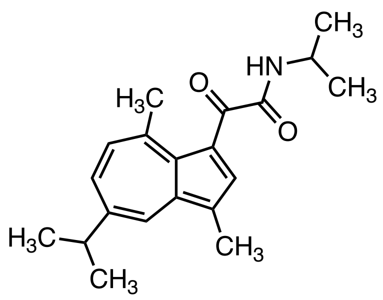

7c

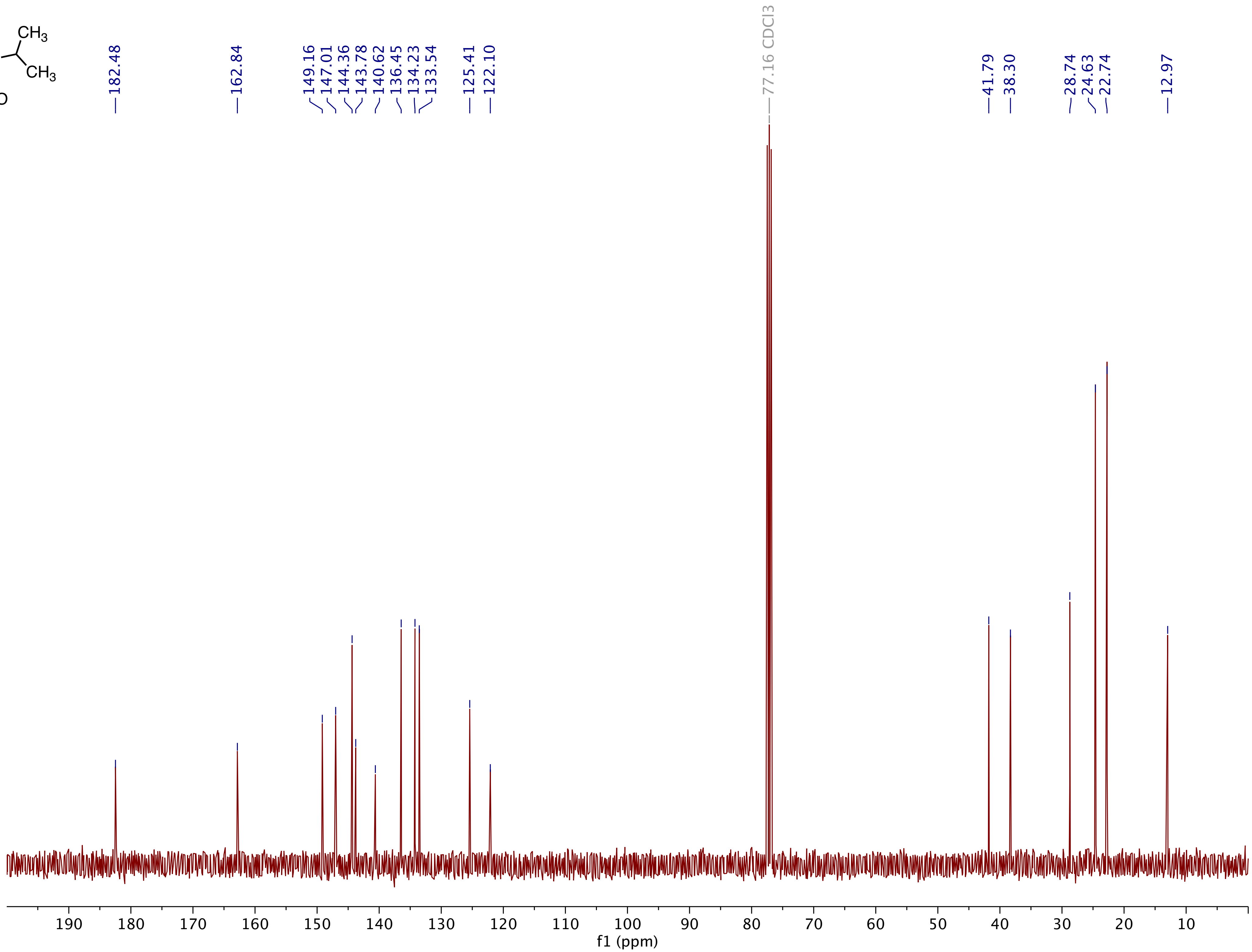

101 MHz $^{13}C\{^{1}H\}$-NMR spectrum of **7c** in $CDCl_3$

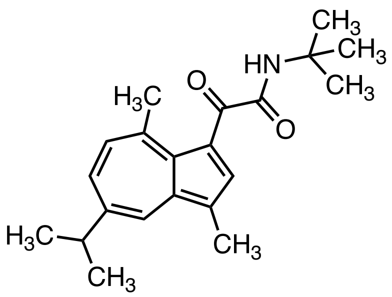

7d

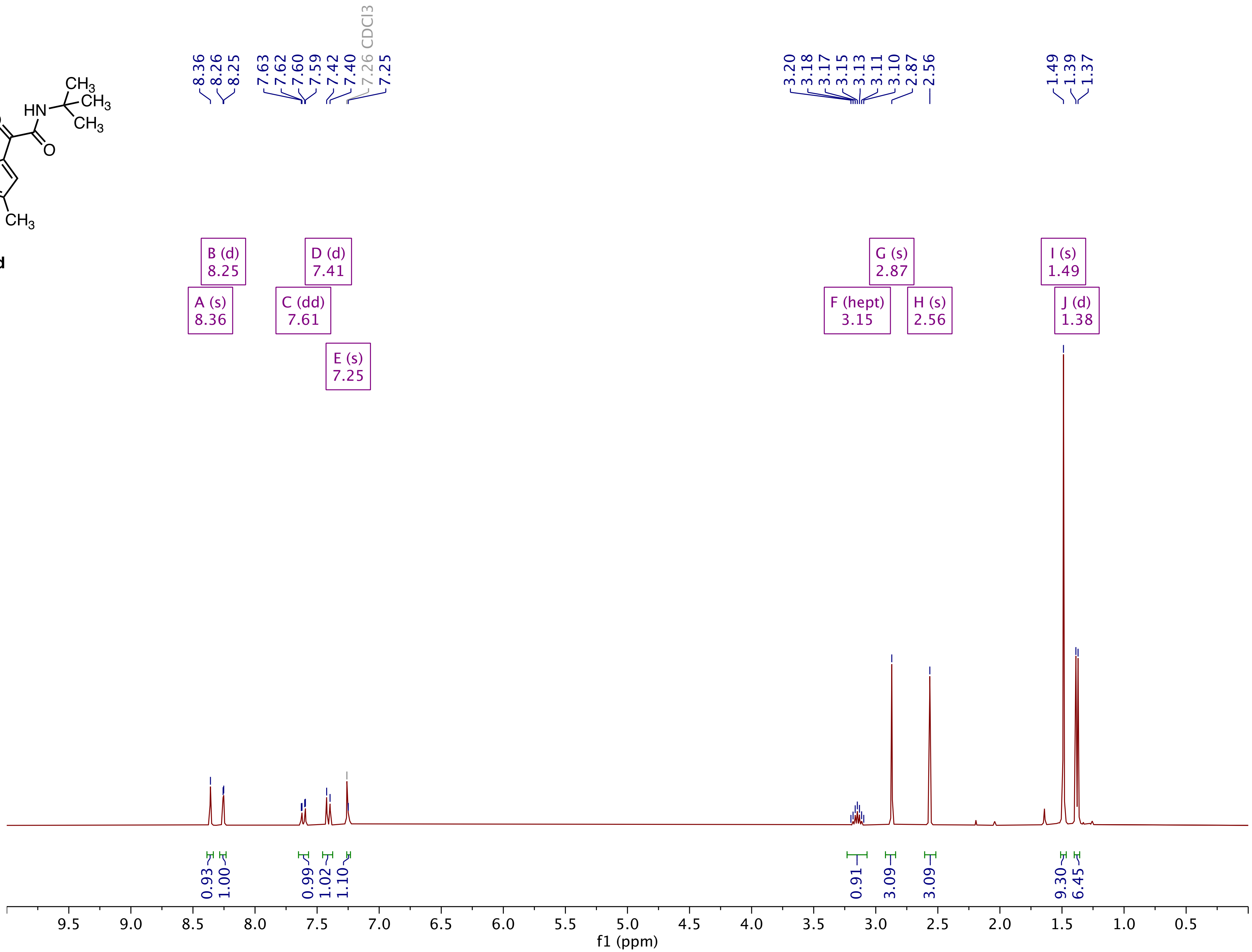

400 MHz ^{1}H-NMR spectrum of **7d** in $CDCl_3$

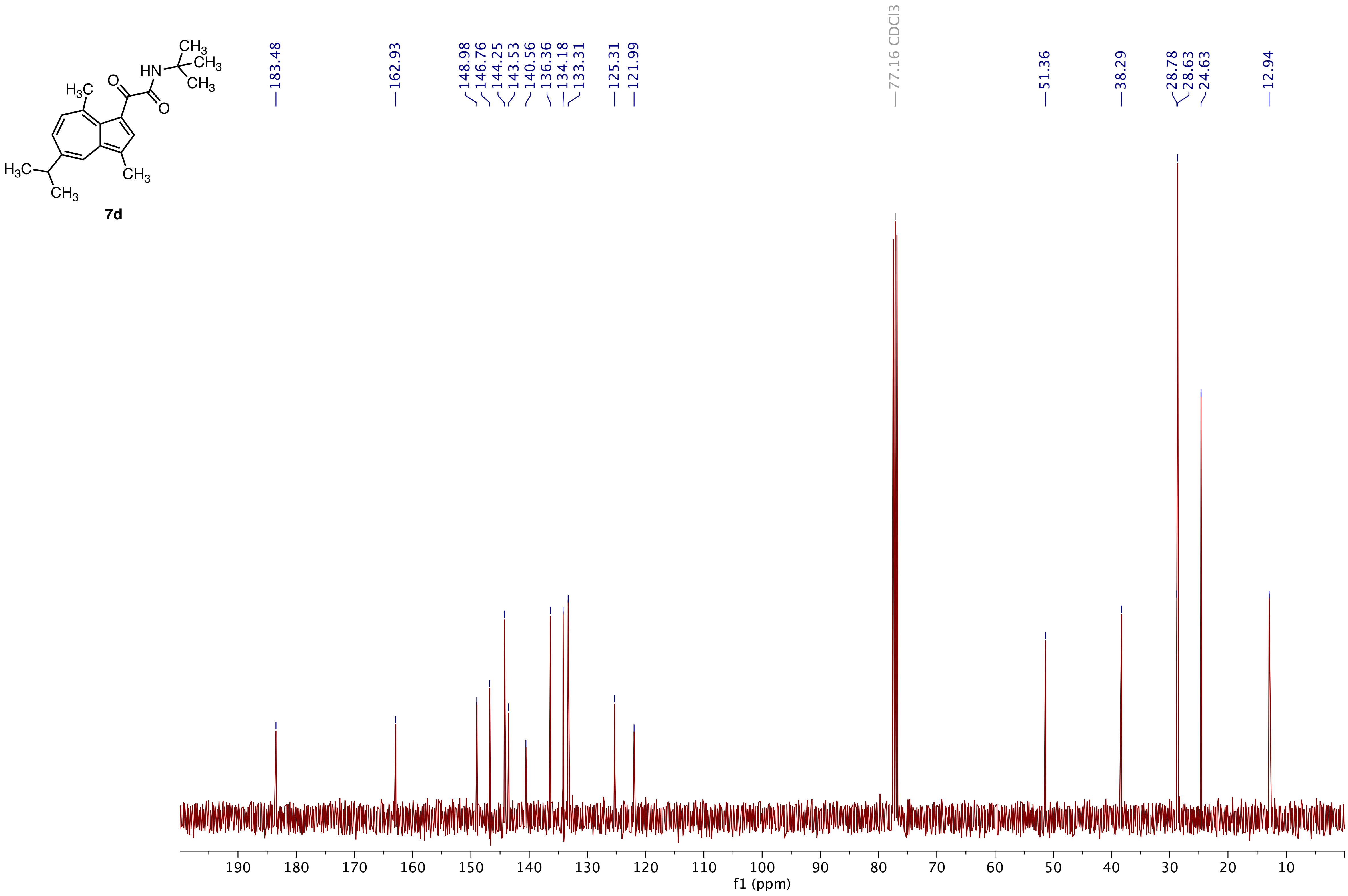

101 MHz $^{13}C\{^{1}H\}$-NMR spectrum of **7d** in $CDCl_3$

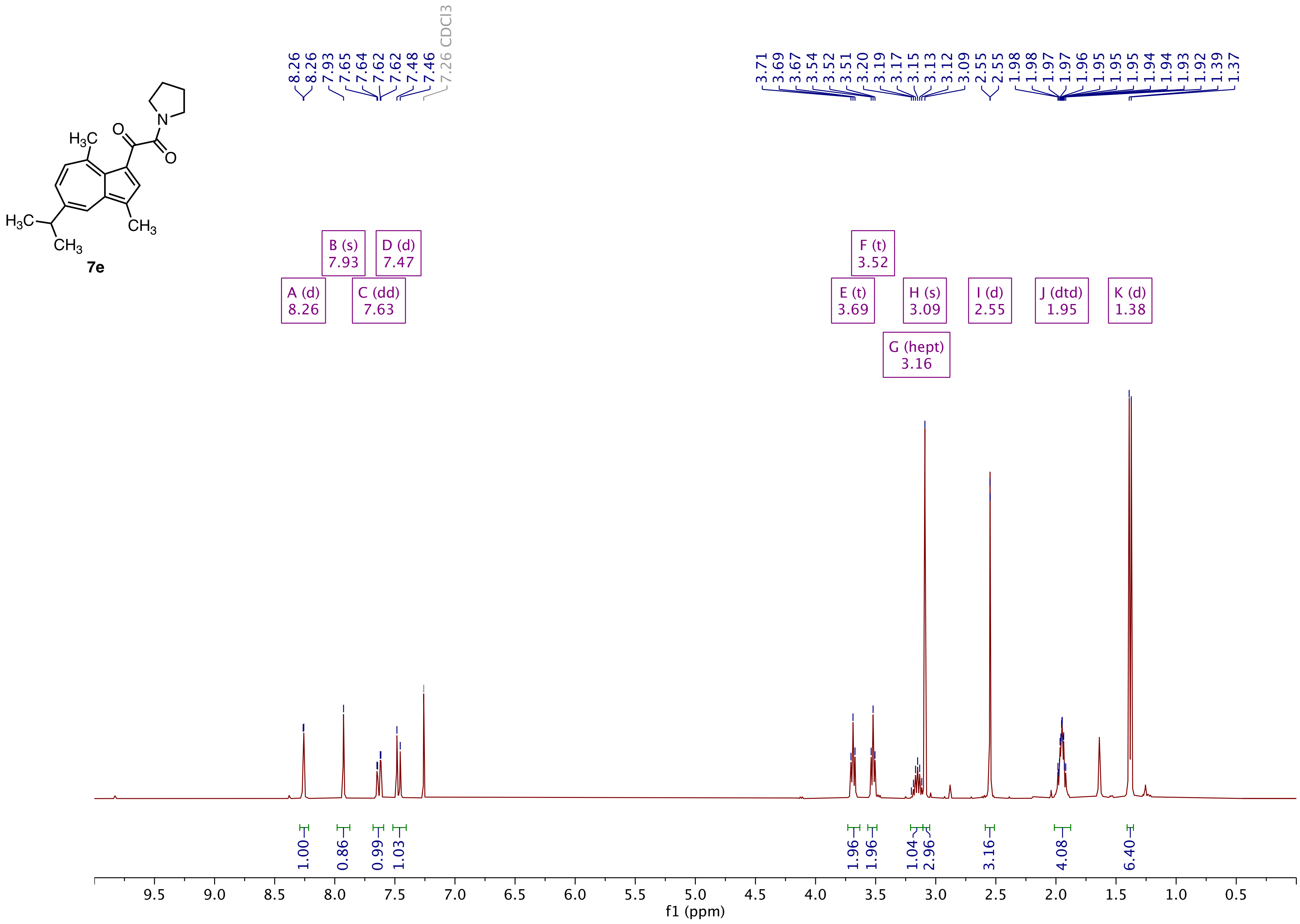

400 MHz ^{1}H-NMR spectrum of **7e** in $CDCl_3$

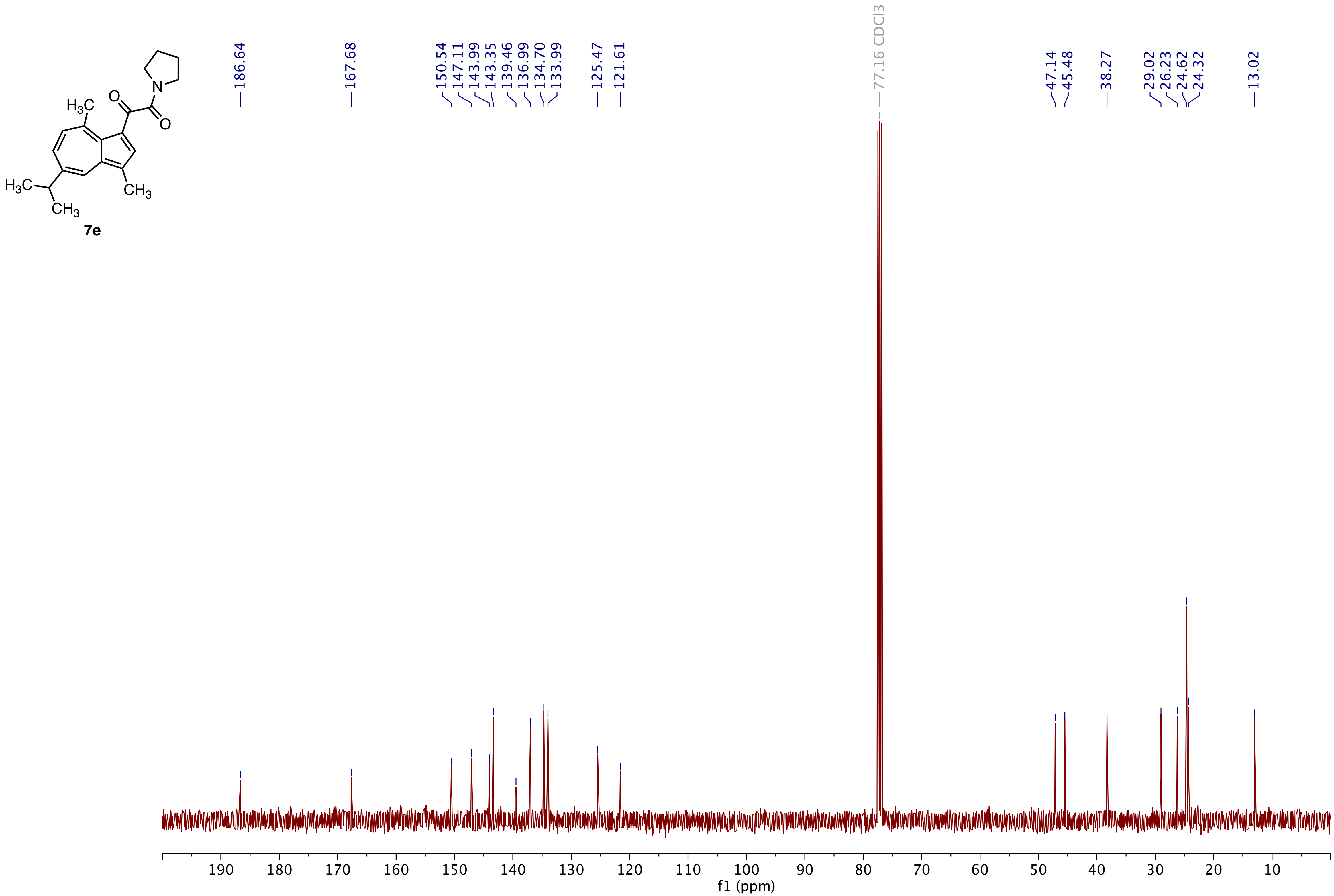

101 MHz $^{13}C\{^{1}H\}$-NMR spectrum of **7e** in $CDCl_3$

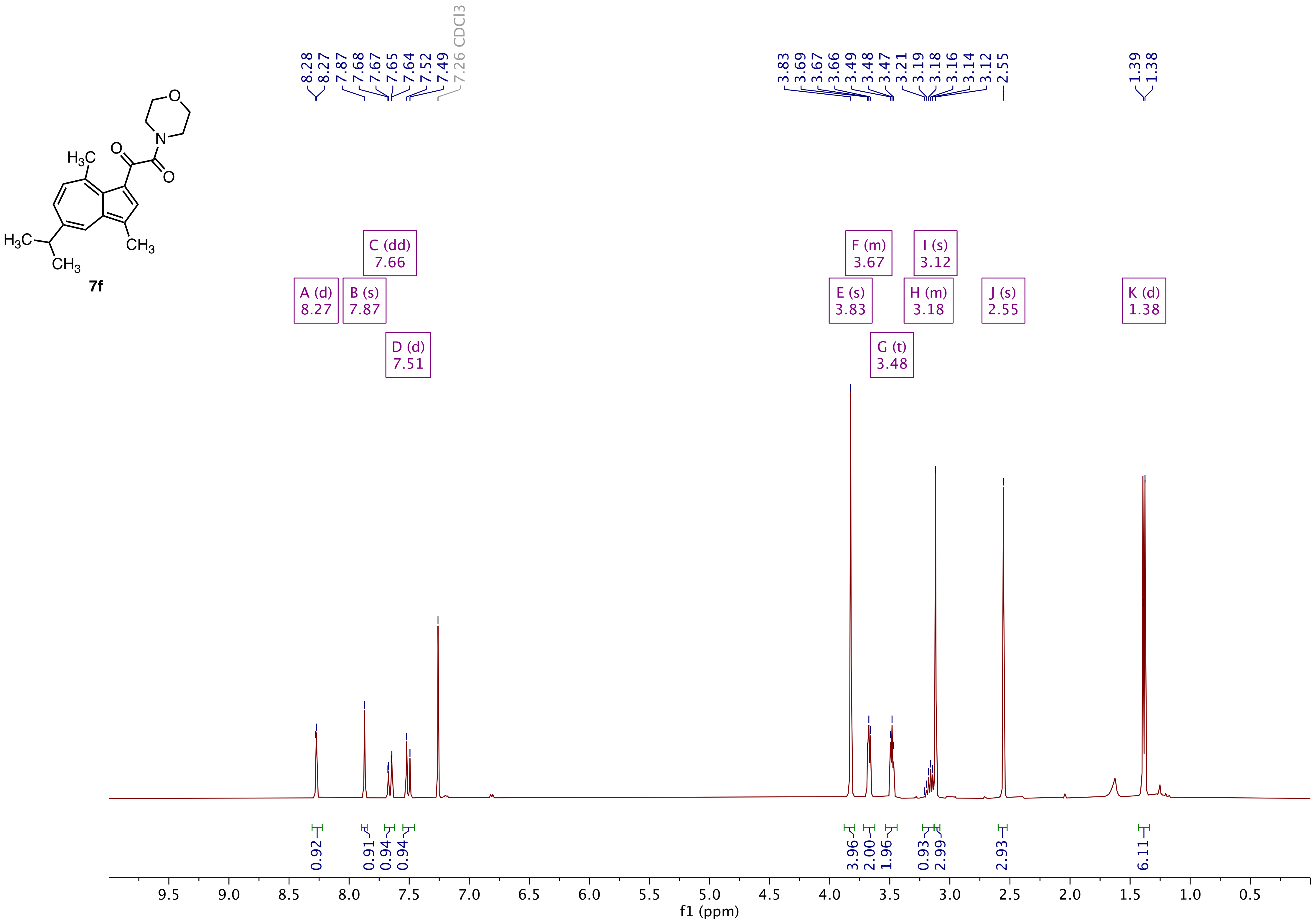

400 MHz ¹H-NMR spectrum of **7f** in $CDCl_3$

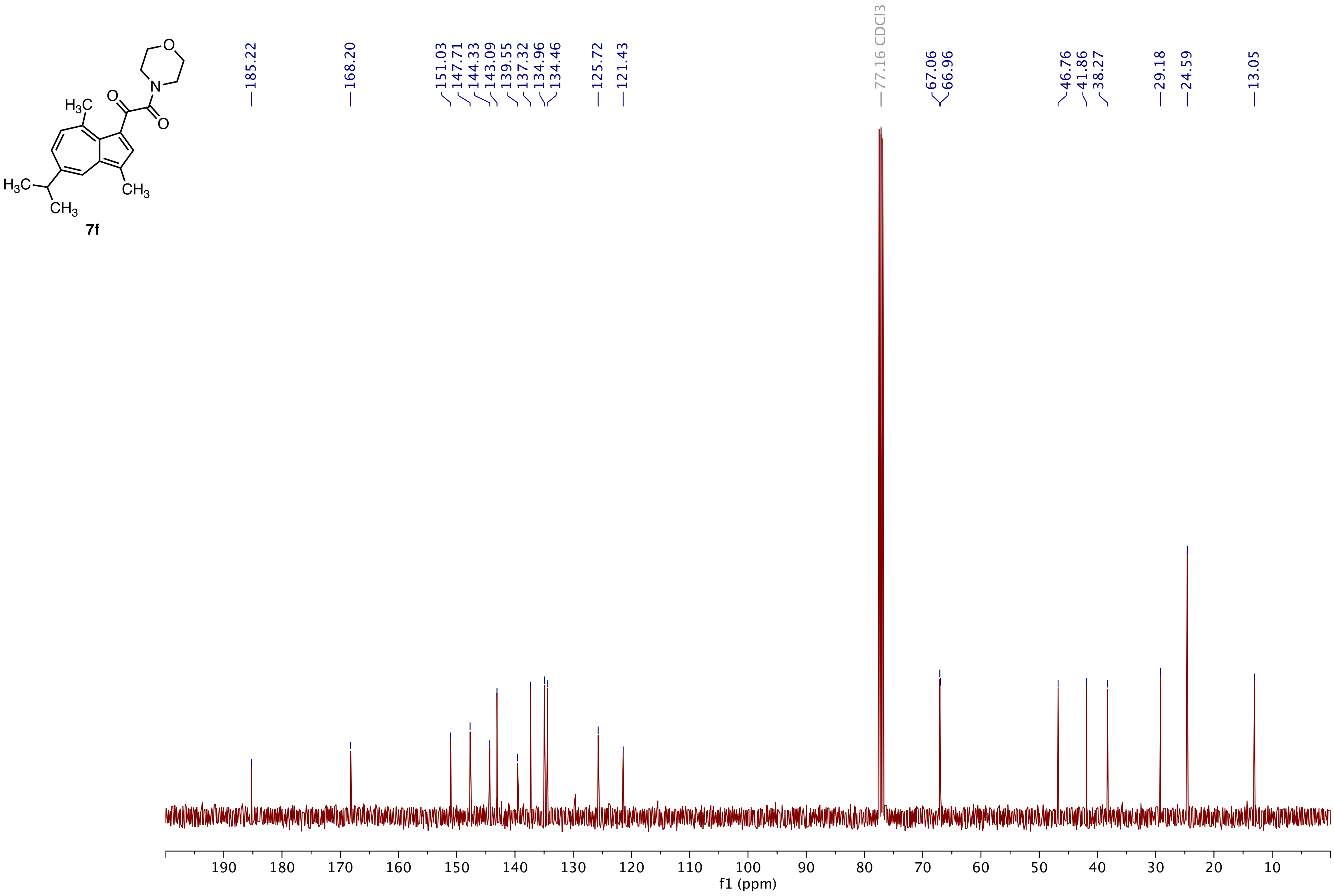

101 MHz $^{13}C\{^{1}H\}$-NMR spectrum of **7f** in $CDCl_3$

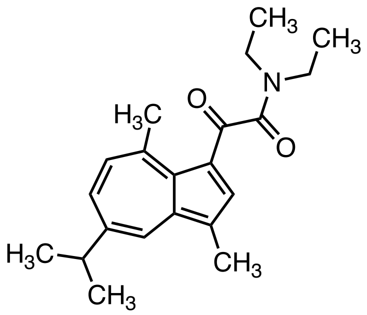

7g

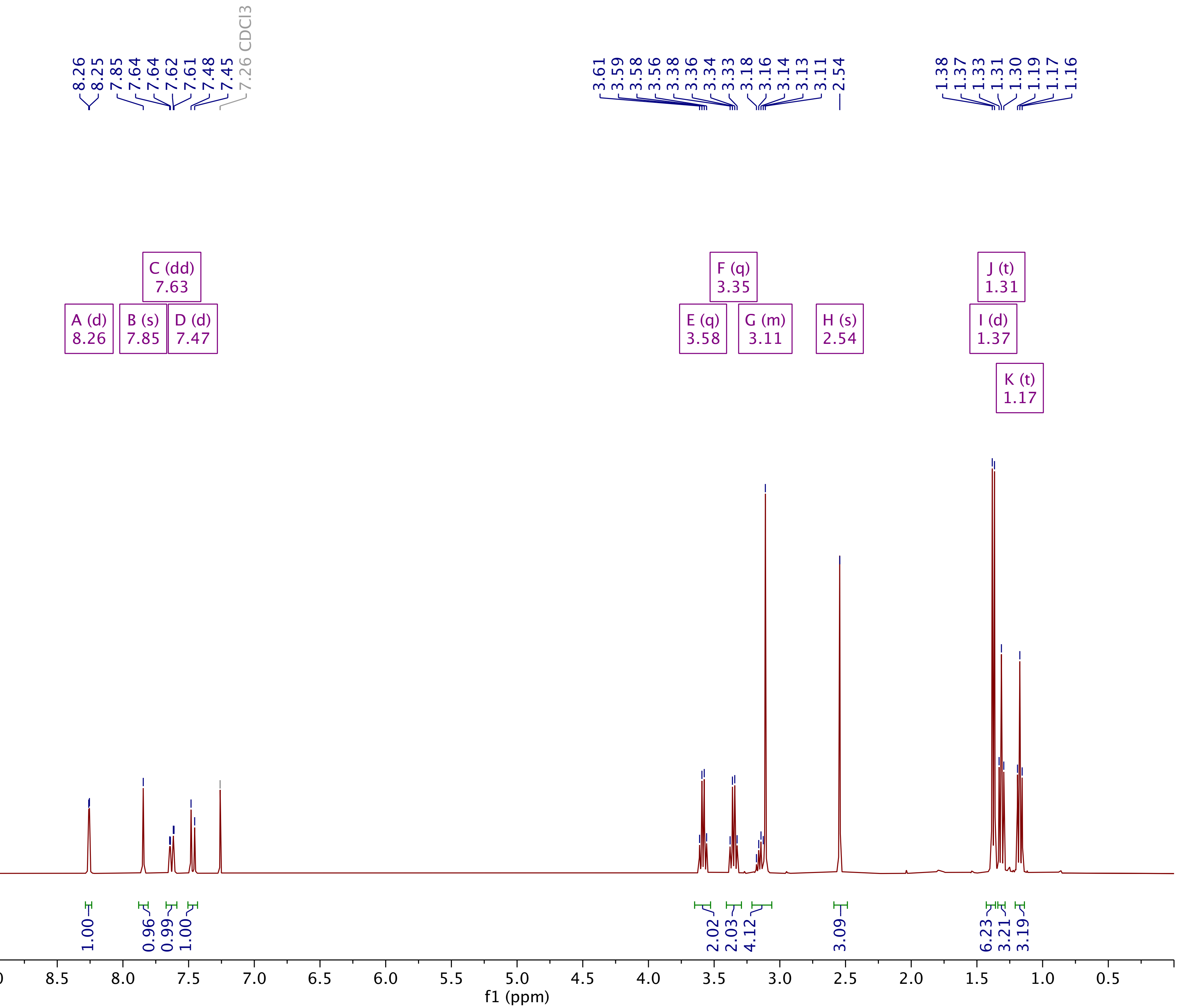

400 MHz ^{1}H-NMR spectrum of **7g** in $CDCl_3$

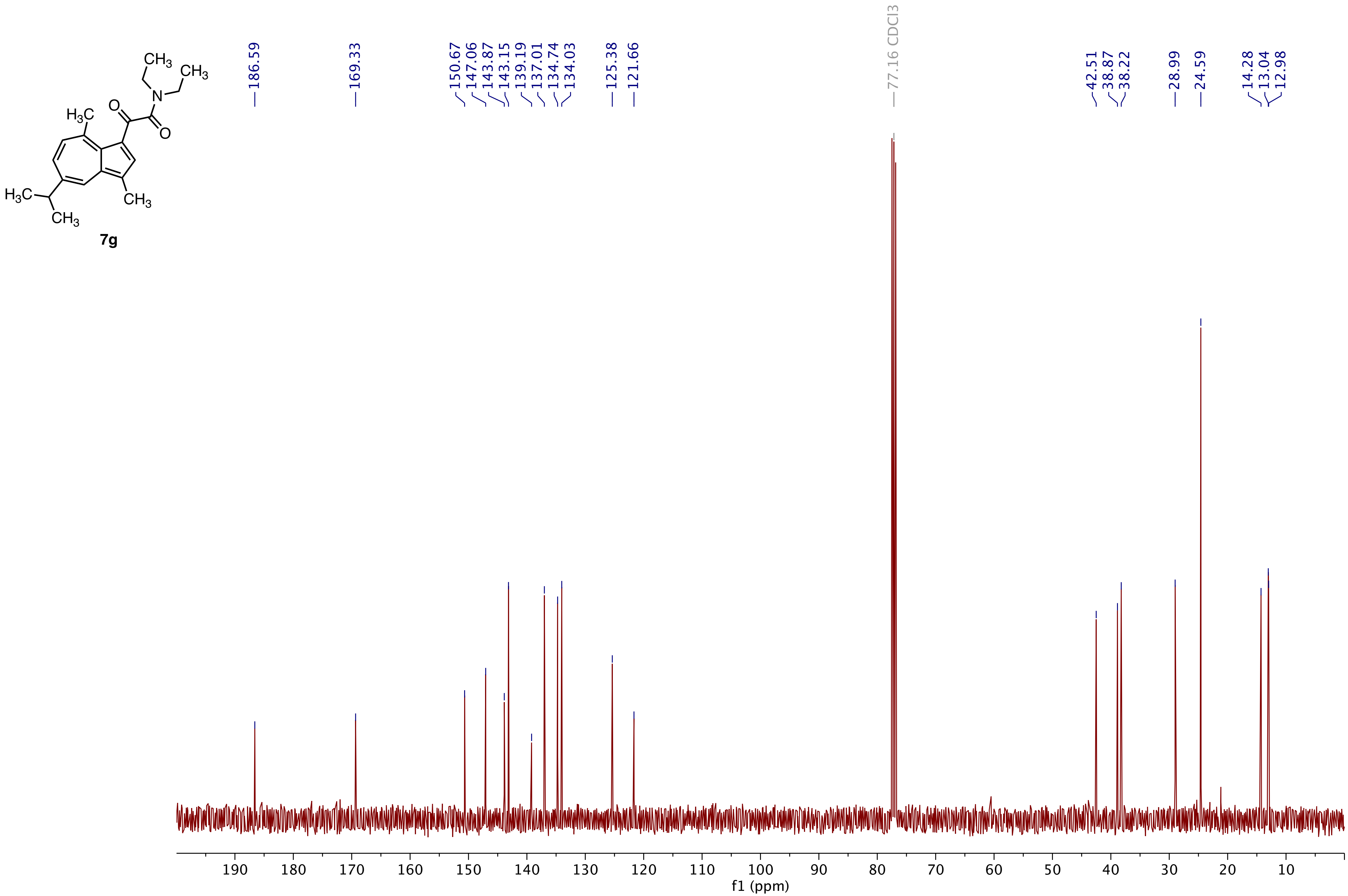

101 MHz $^{13}C\{^{1}H\}$-NMR spectrum of **7g** in $CDCl_3$

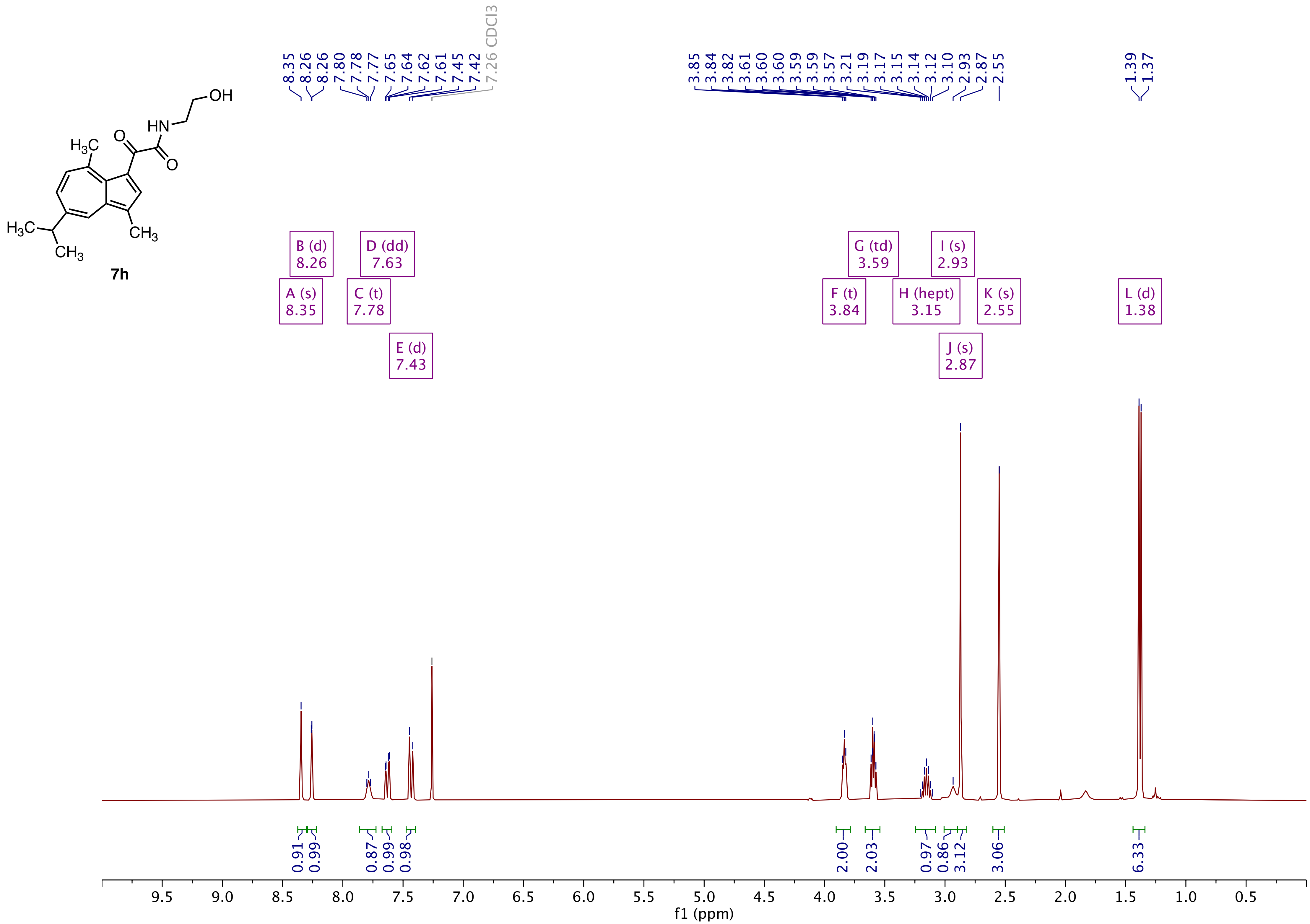

400 MHz ^{1}H-NMR spectrum of **7h** in $CDCl_3$

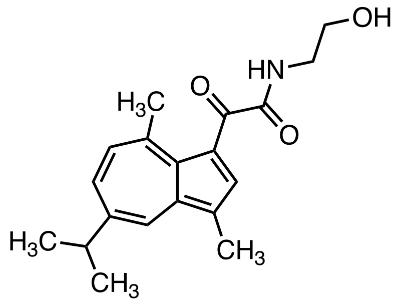

7h

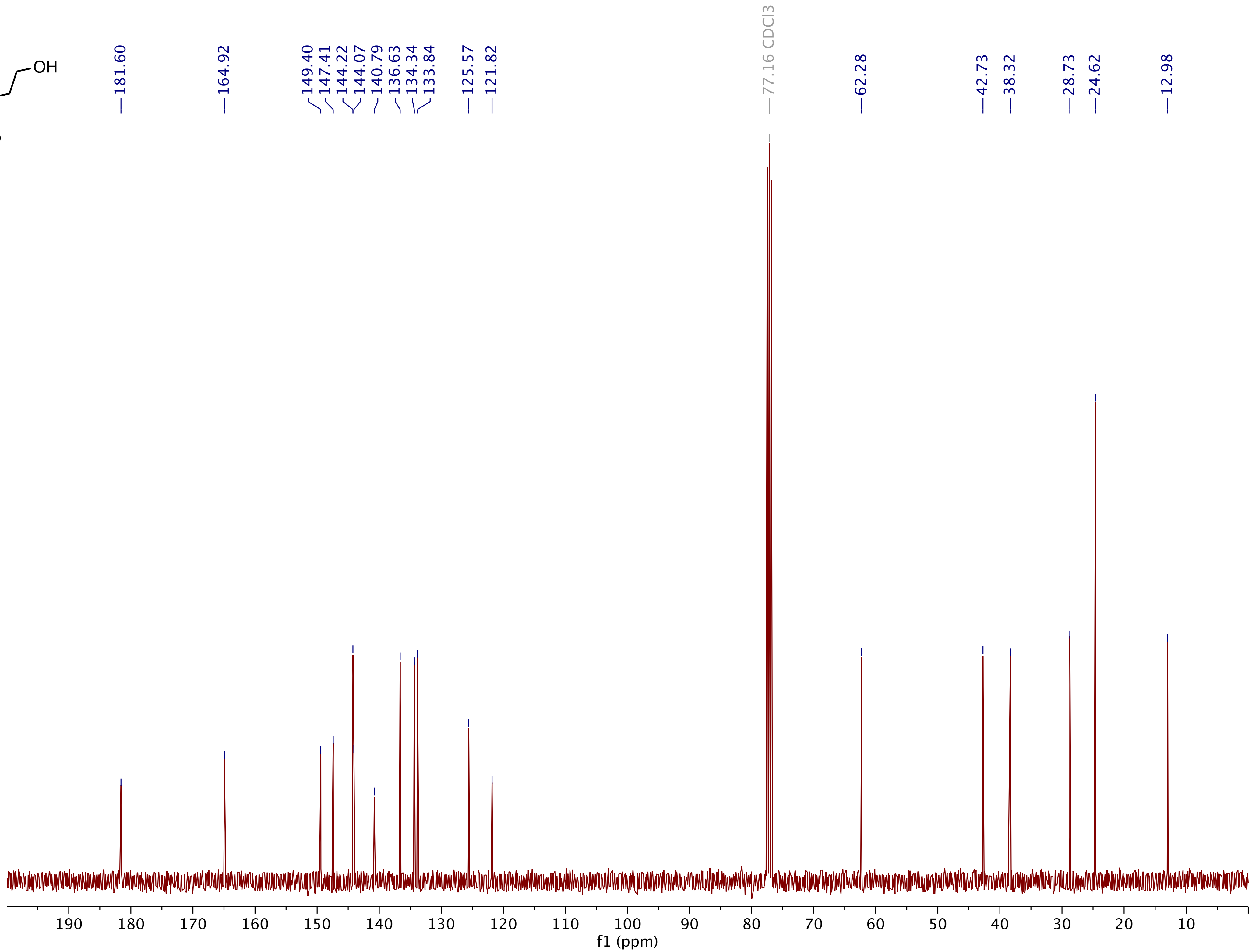

101 MHz $^{13}C\{^1H\}$-NMR spectrum of **7h** in $CDCl_3$

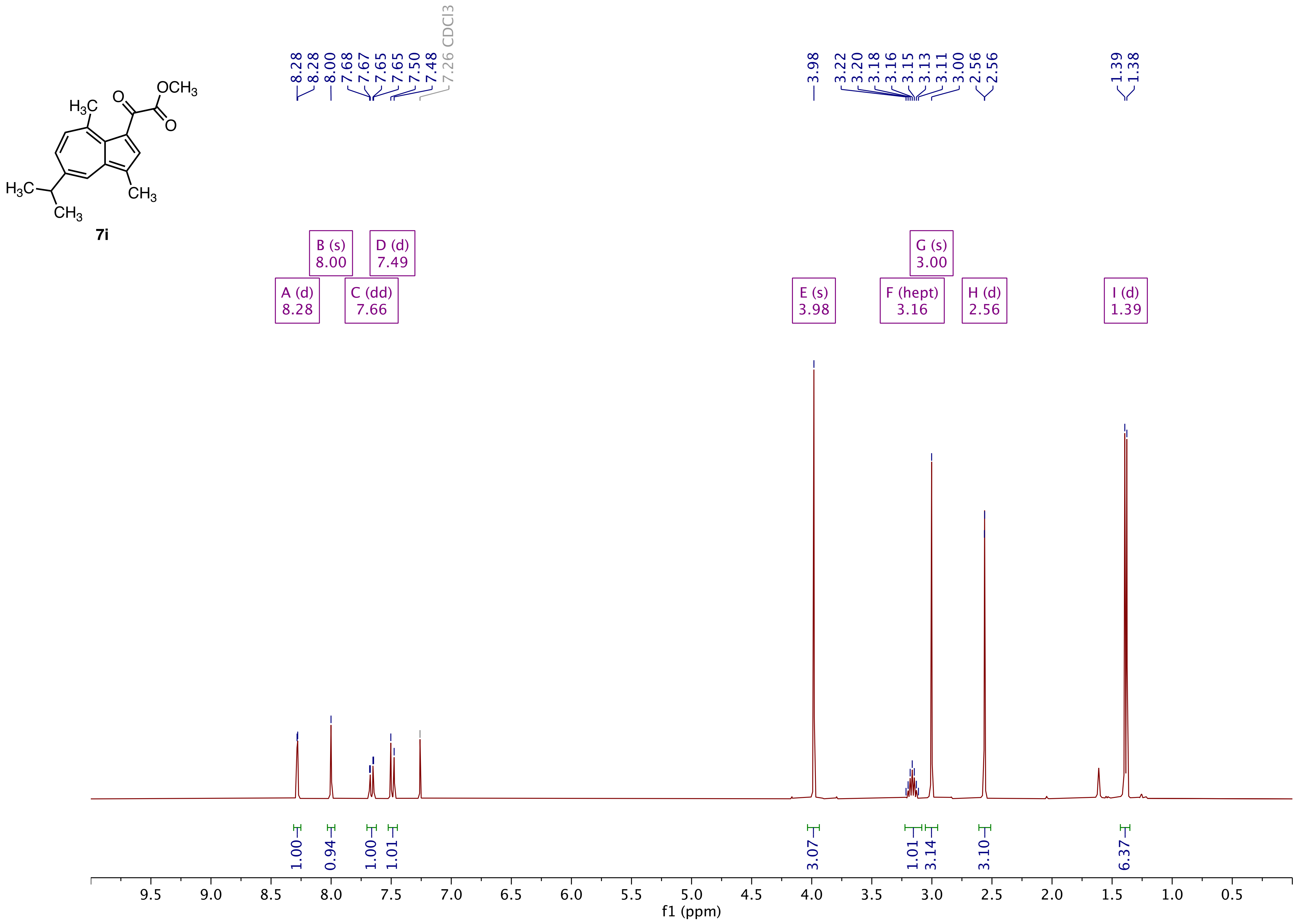

400 MHz ^{1}H-NMR spectrum of **7i** in $CDCl_3$

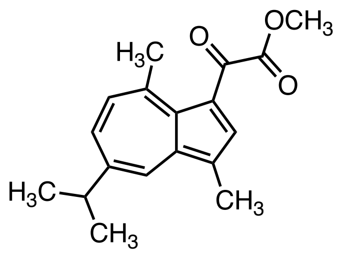

7i

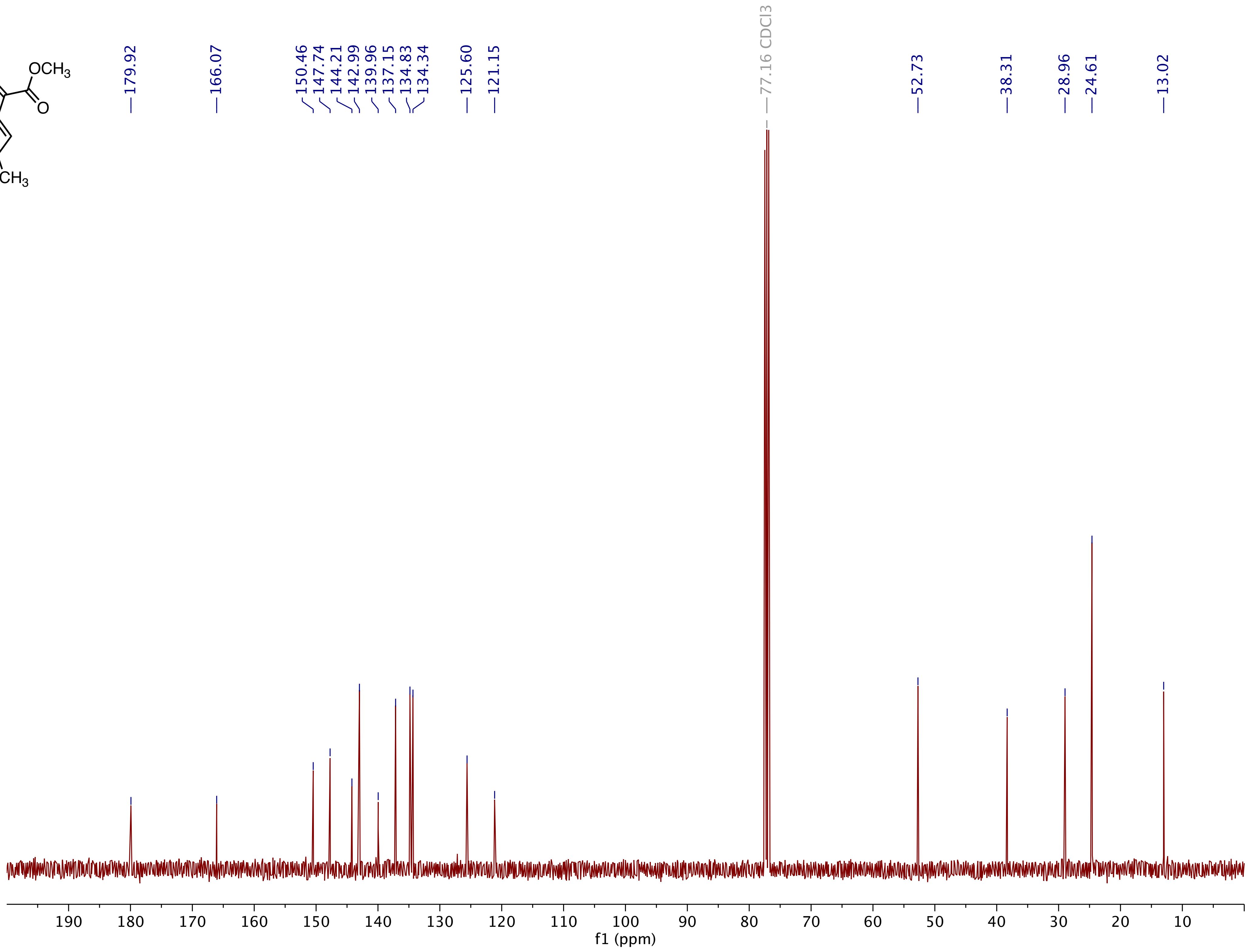

101 MHz $^{13}C\{^{1}H\}$-NMR spectrum of **7i** in $CDCl_3$

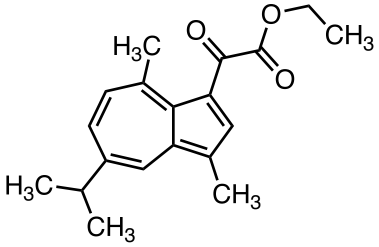

7j

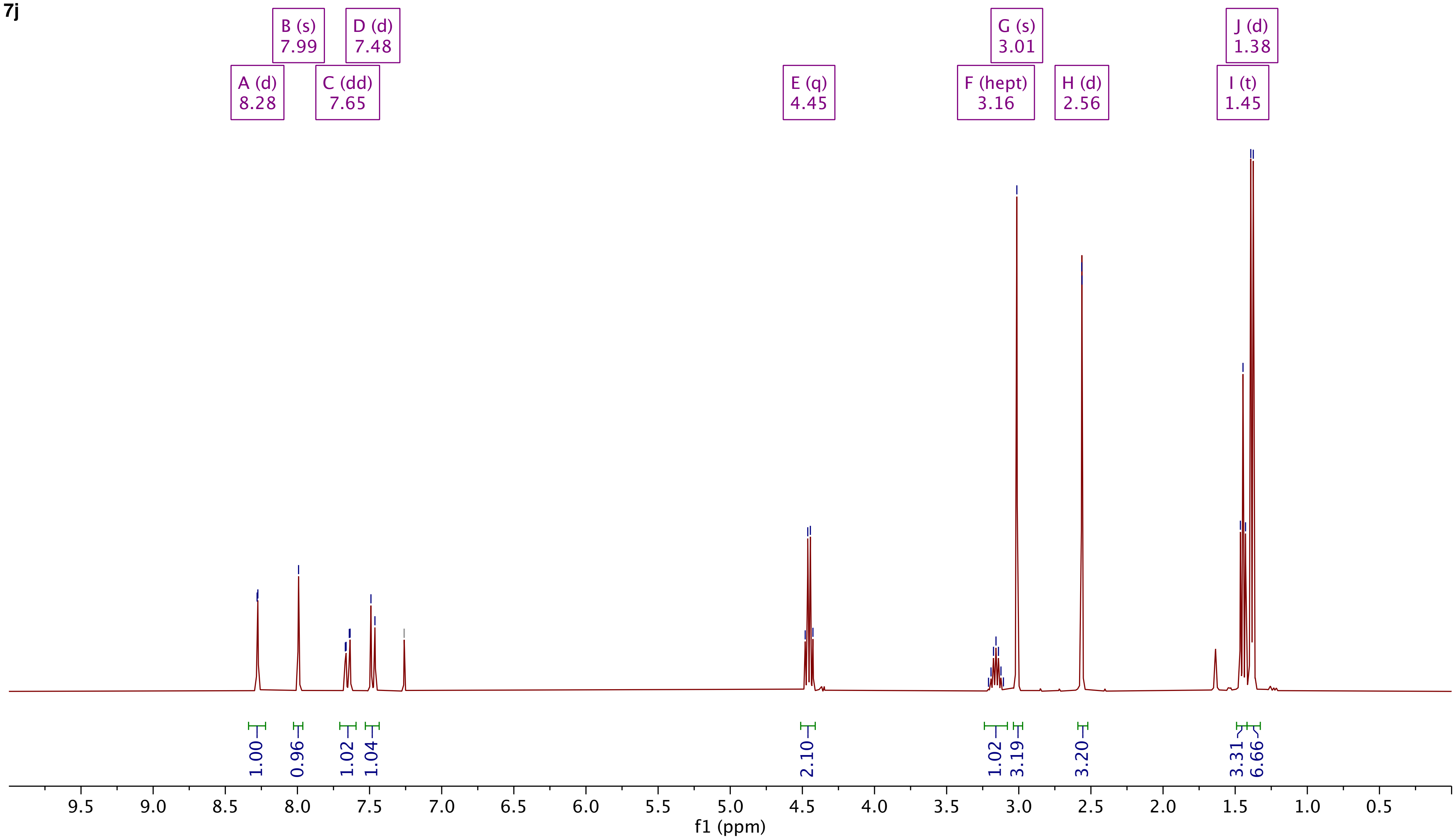

400 MHz ^{1}H-NMR spectrum of **7j** in $CDCl_3$

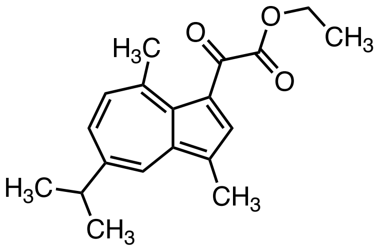

7j

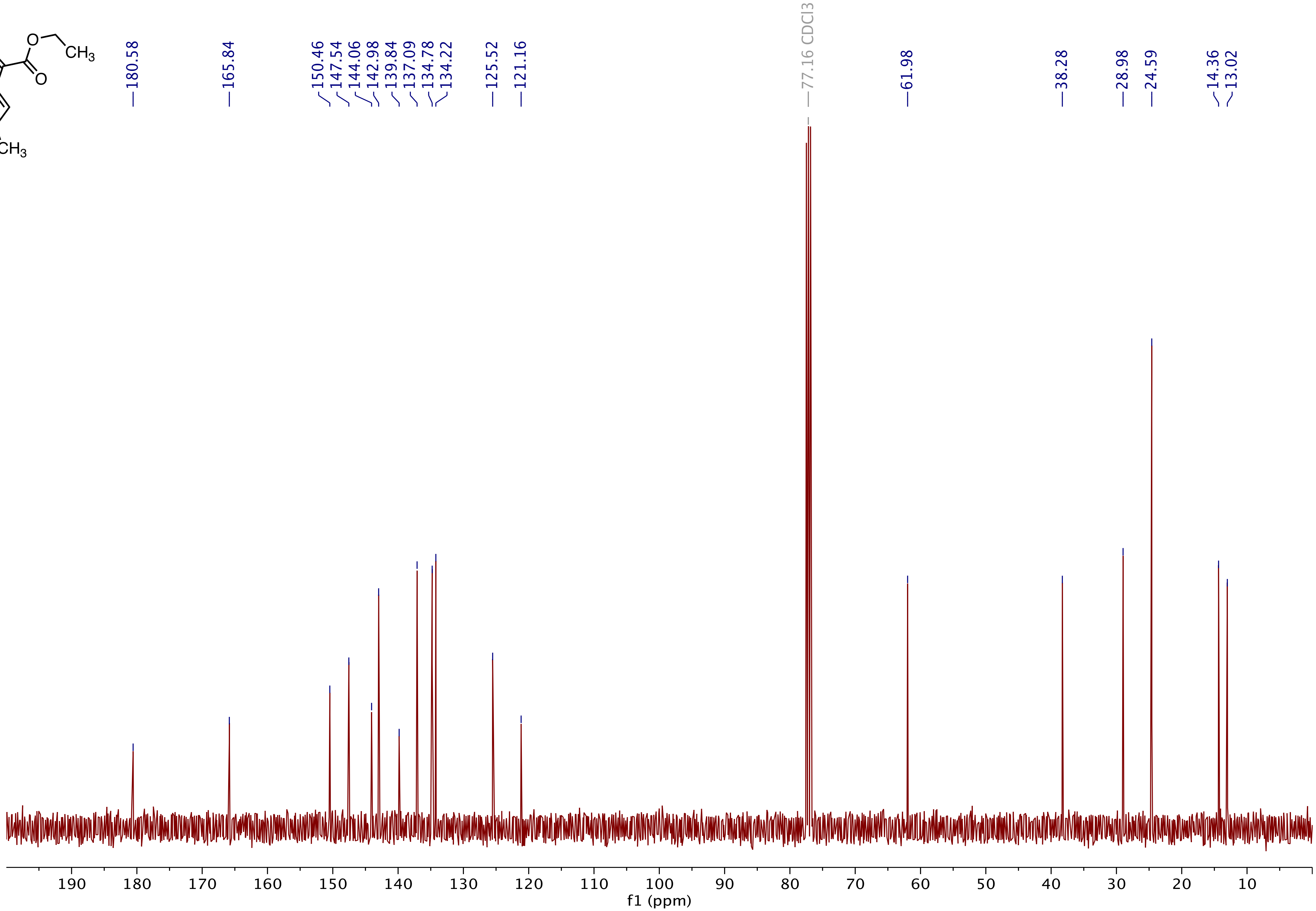

101 MHz $^{13}C\{^{1}H\}$-NMR spectrum of **7j** in $CDCl_3$

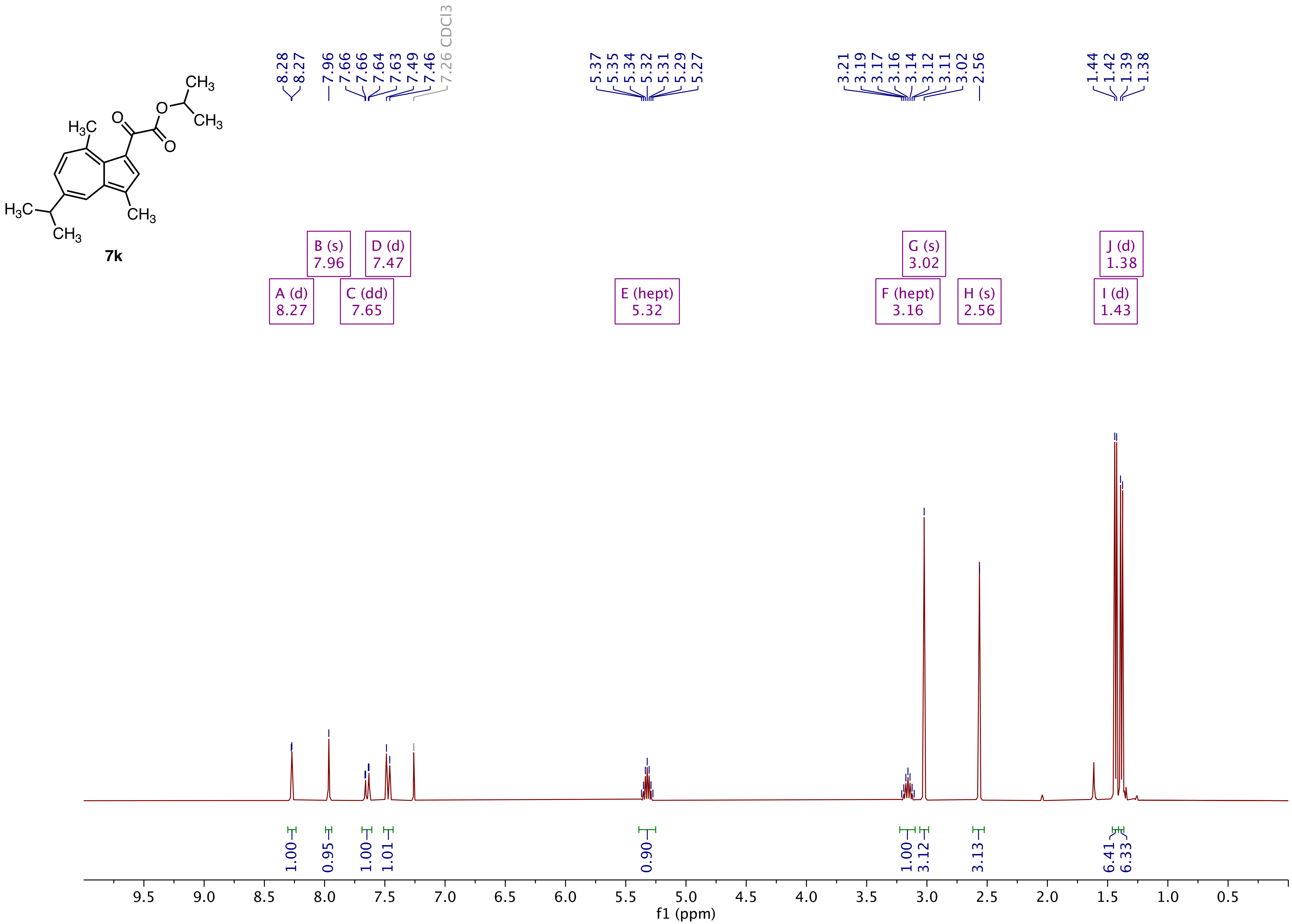

400 MHz ^{1}H-NMR spectrum of **7k** in $CDCl_3$

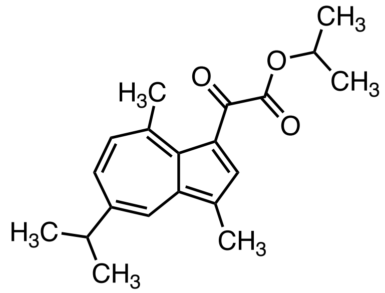

7k

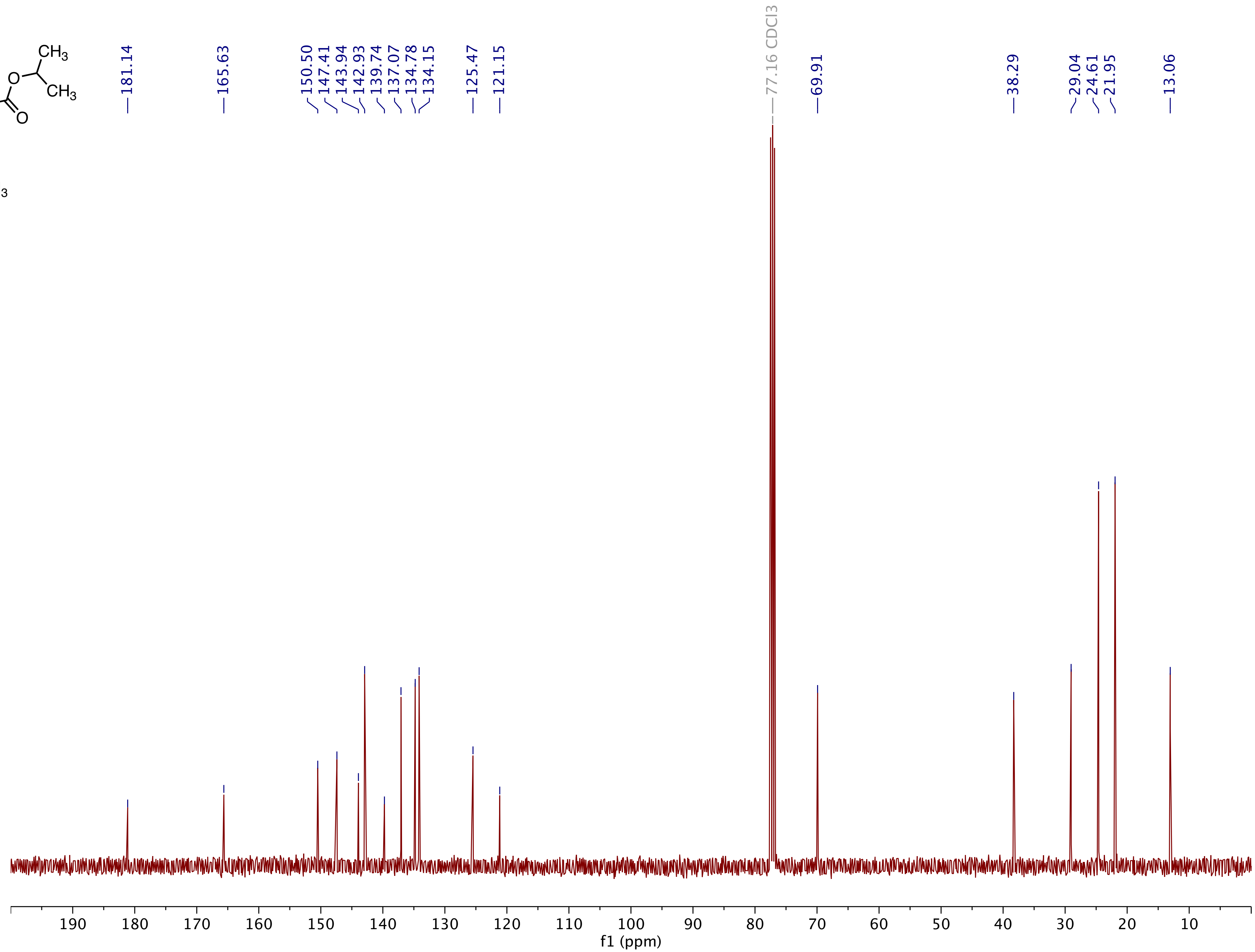

101 MHz $^{13}C\{^{1}H\}$-NMR spectrum of **7k** in $CDCl_3$

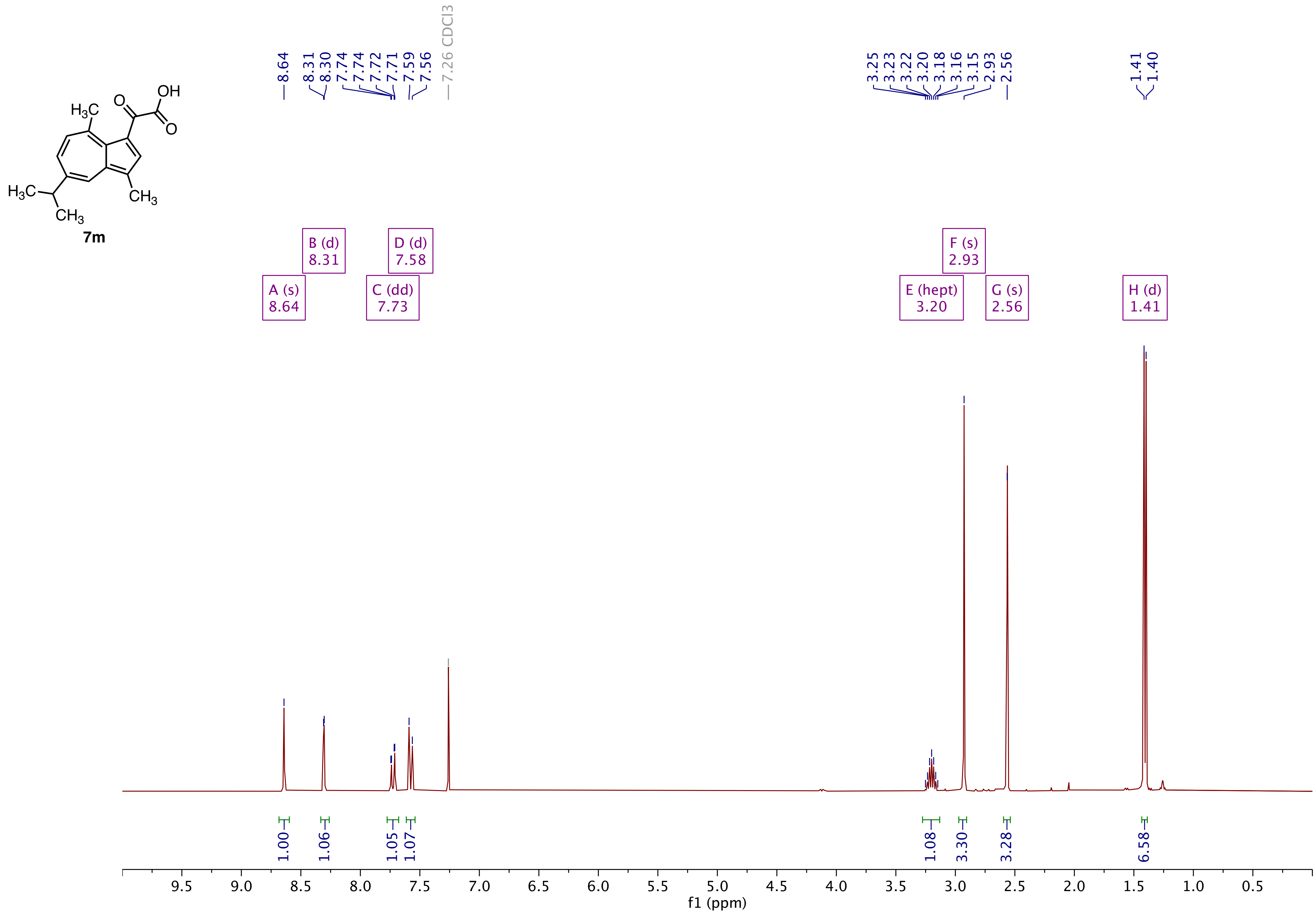

400 MHz ^{1}H-NMR spectrum of **7m** in $CDCl_3$

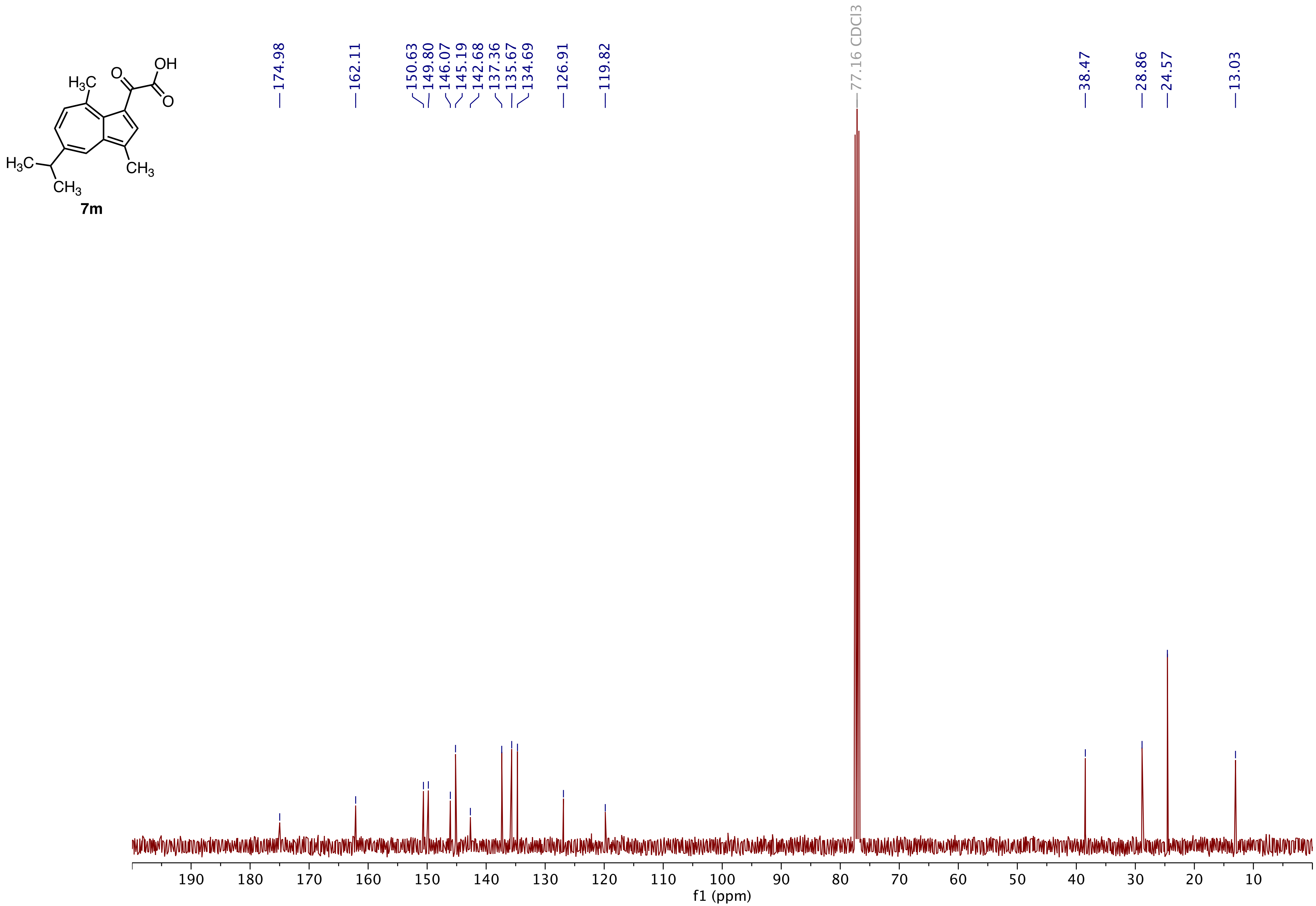

101 MHz $^{13}C\{^{1}H\}$-NMR spectrum of **7m** in $CDCl_3$

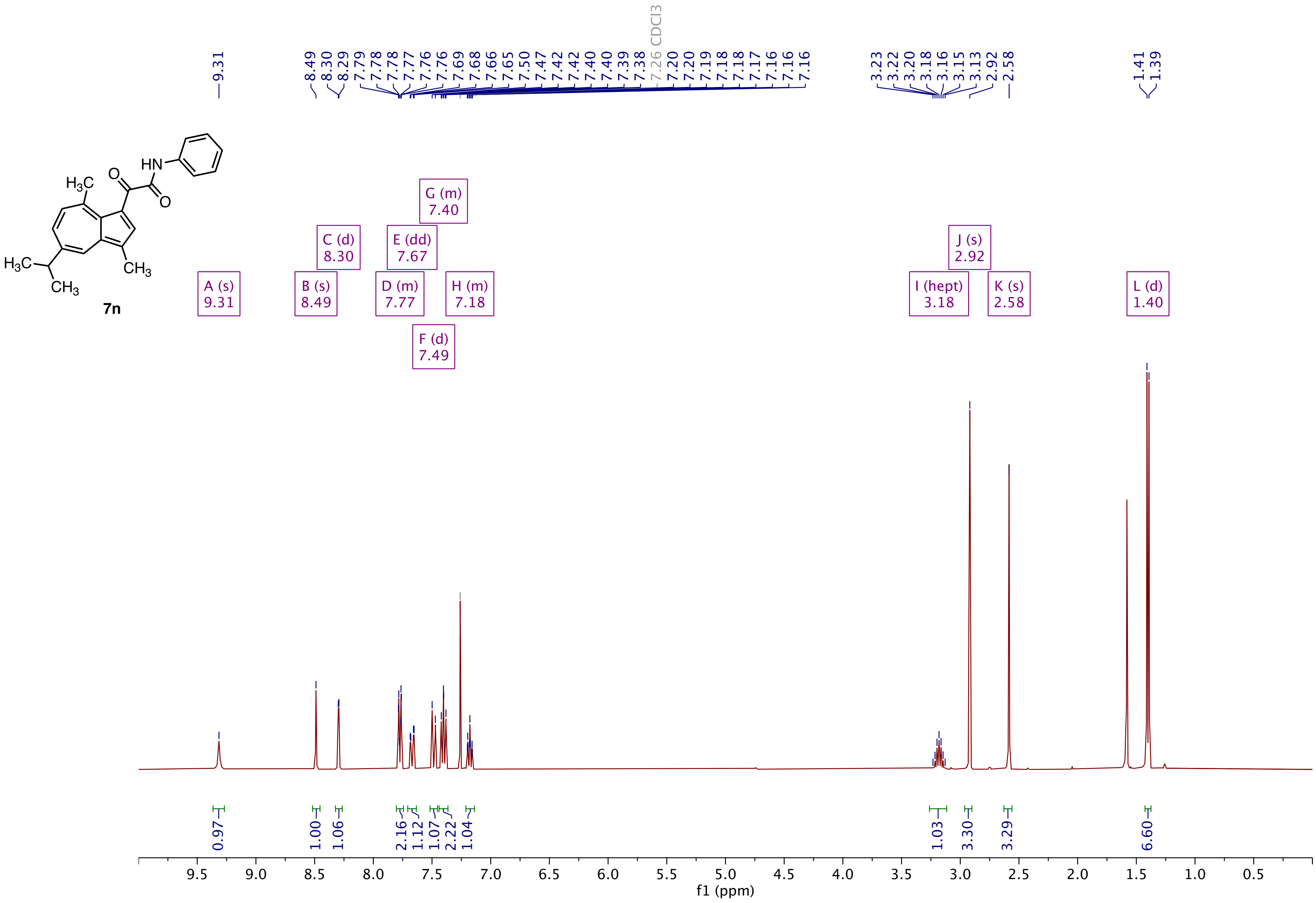

400 MHz ^{1}H-NMR spectrum of **7n** in $CDCl_3$

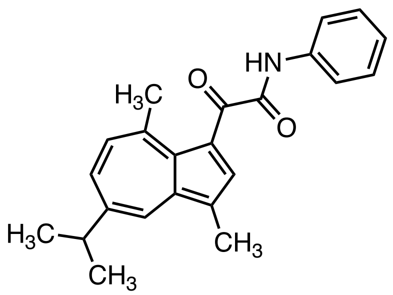

7n

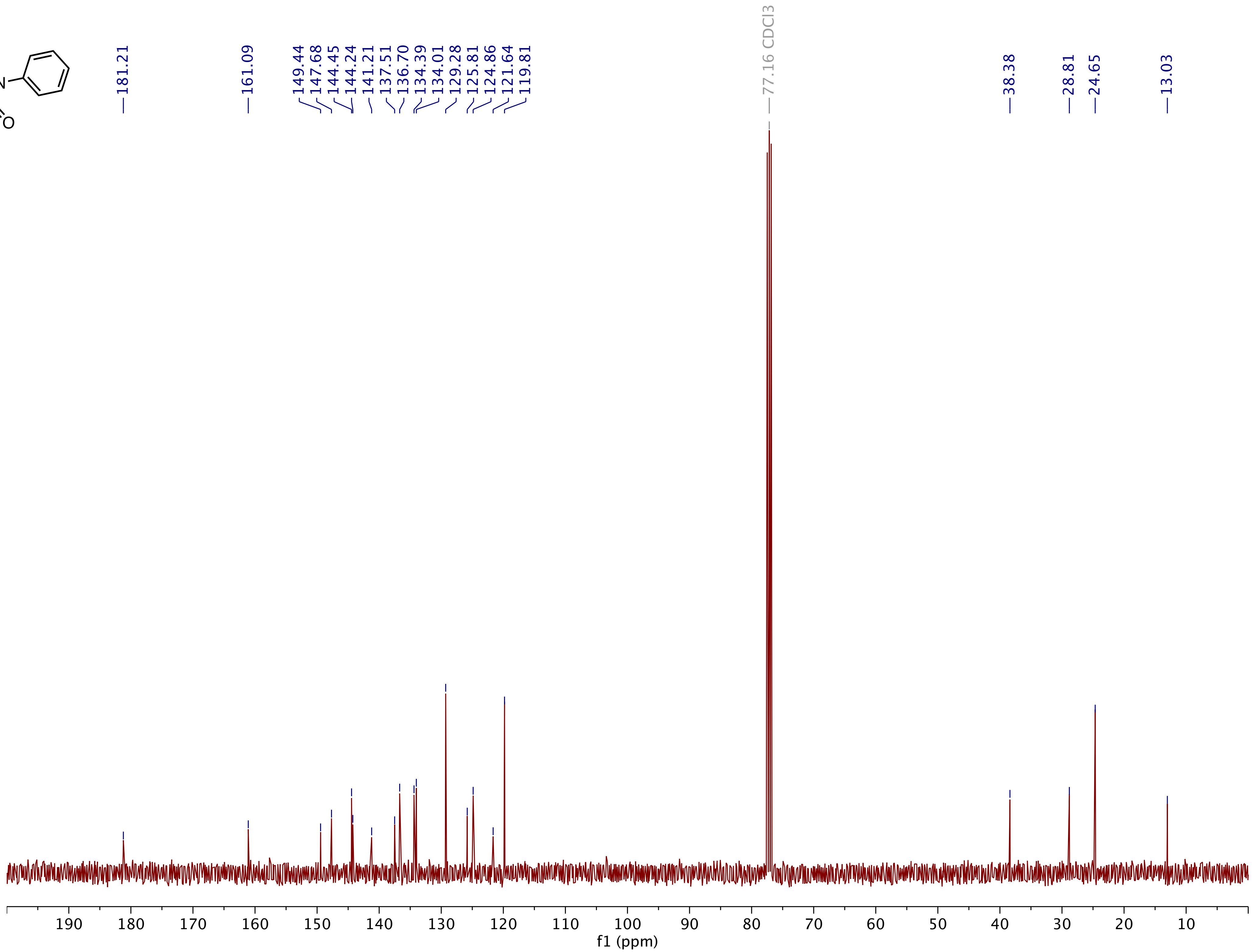

101 MHz $^{13}C\{^{1}H\}$-NMR spectrum of **7n** in $CDCl_3$

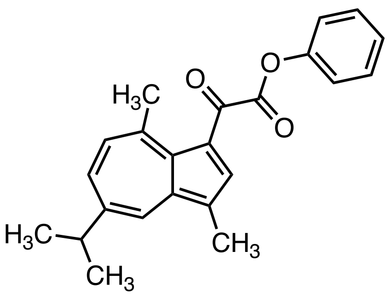

7o

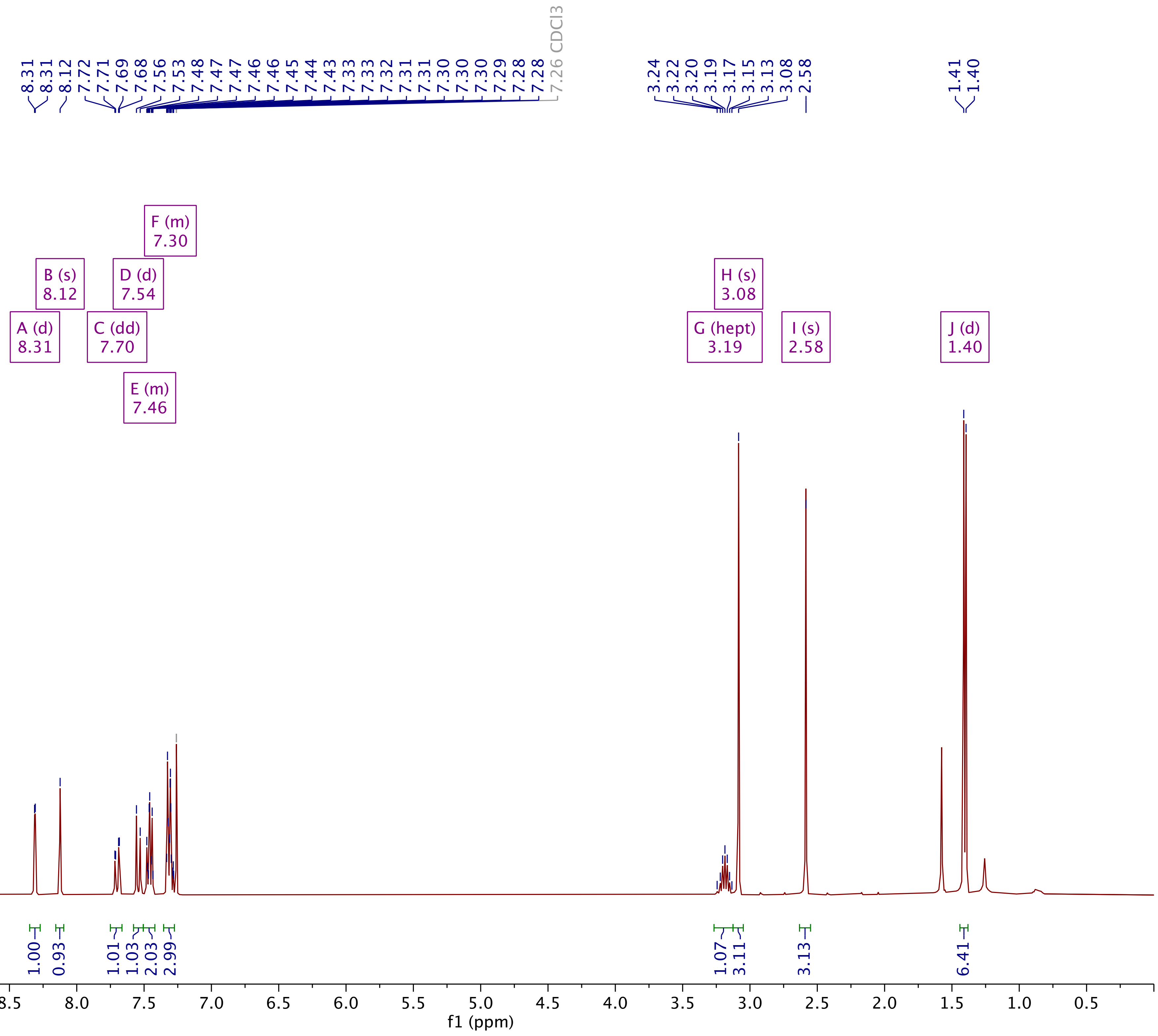

400 MHz ¹H-NMR spectrum of **7o** in $CDCl_3$

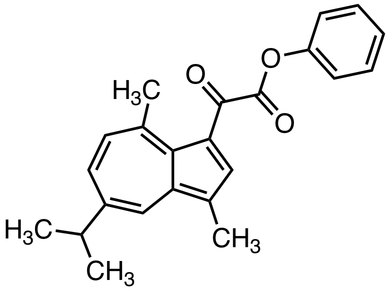

7o

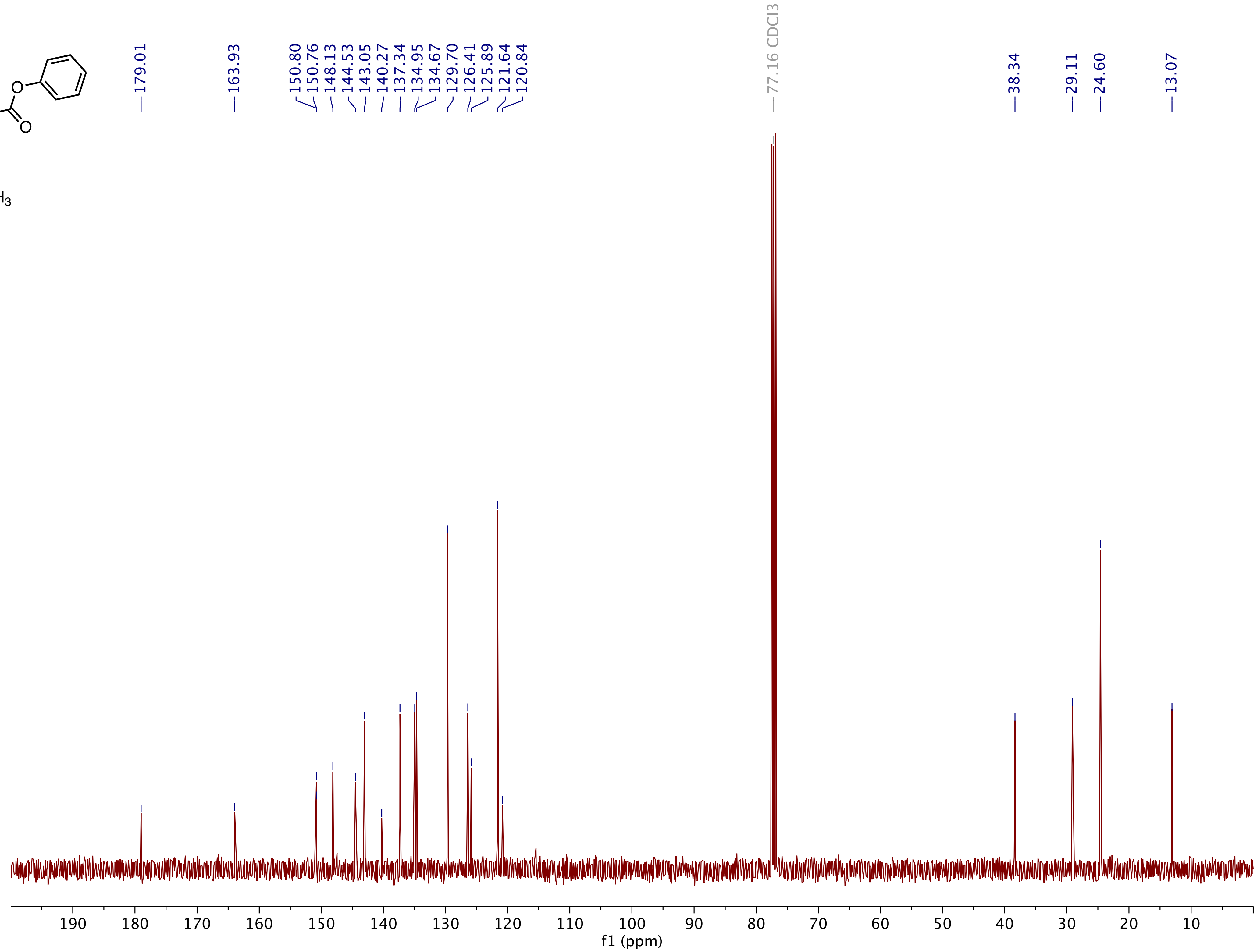

101 MHz $^{13}C\{^{1}H\}$-NMR spectrum of **7o** in $CDCl_3$

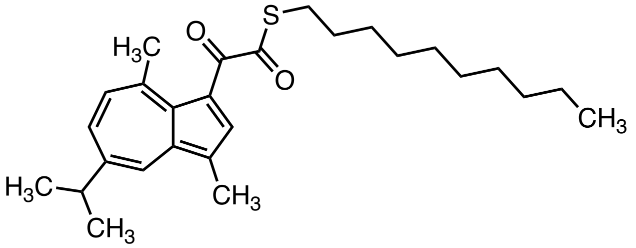

7p

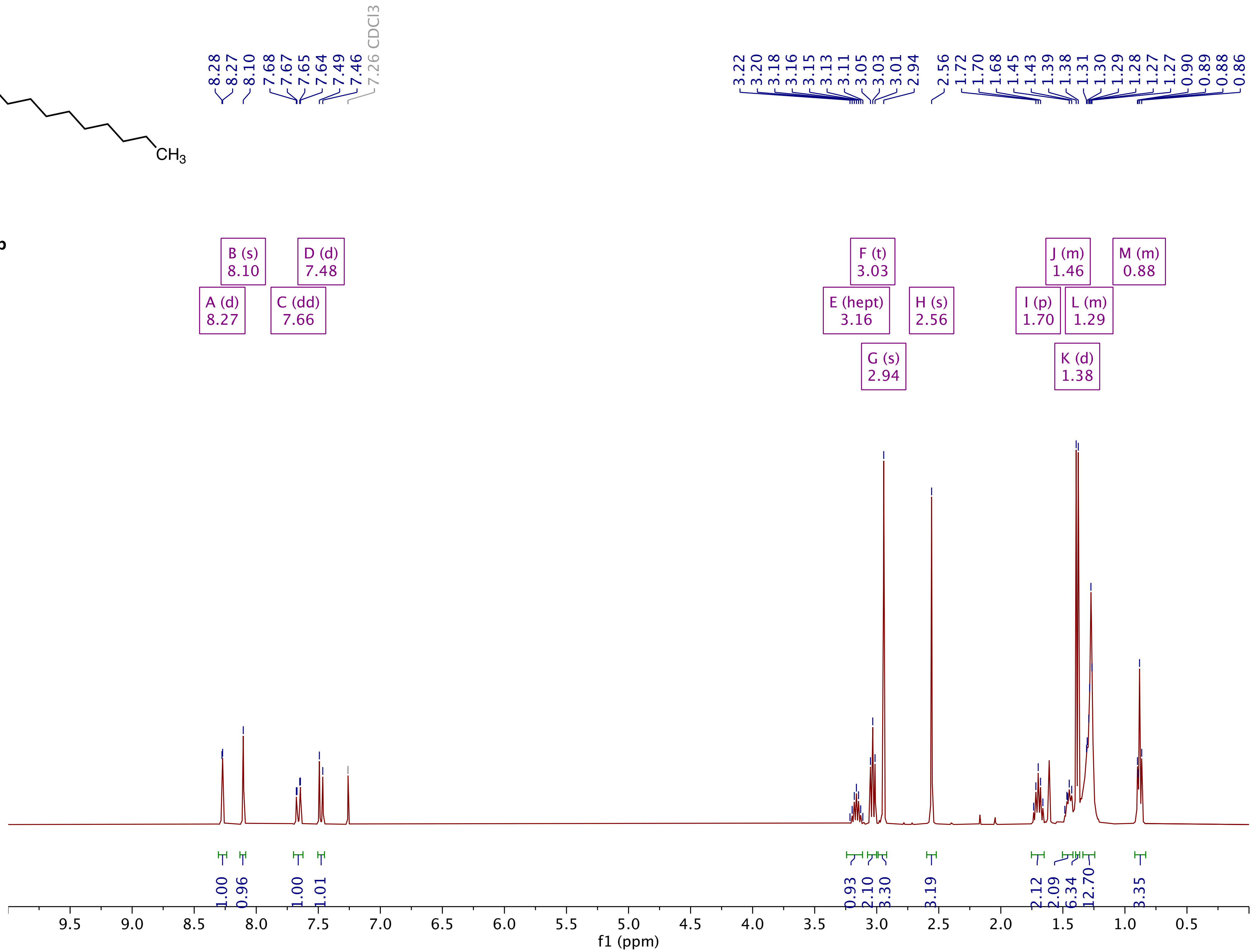

400 MHz ^{1}H-NMR spectrum of **7p** in $CDCl_3$

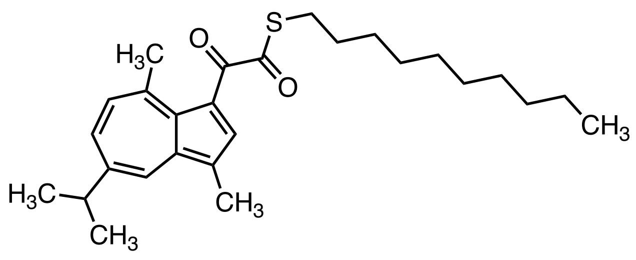

7p

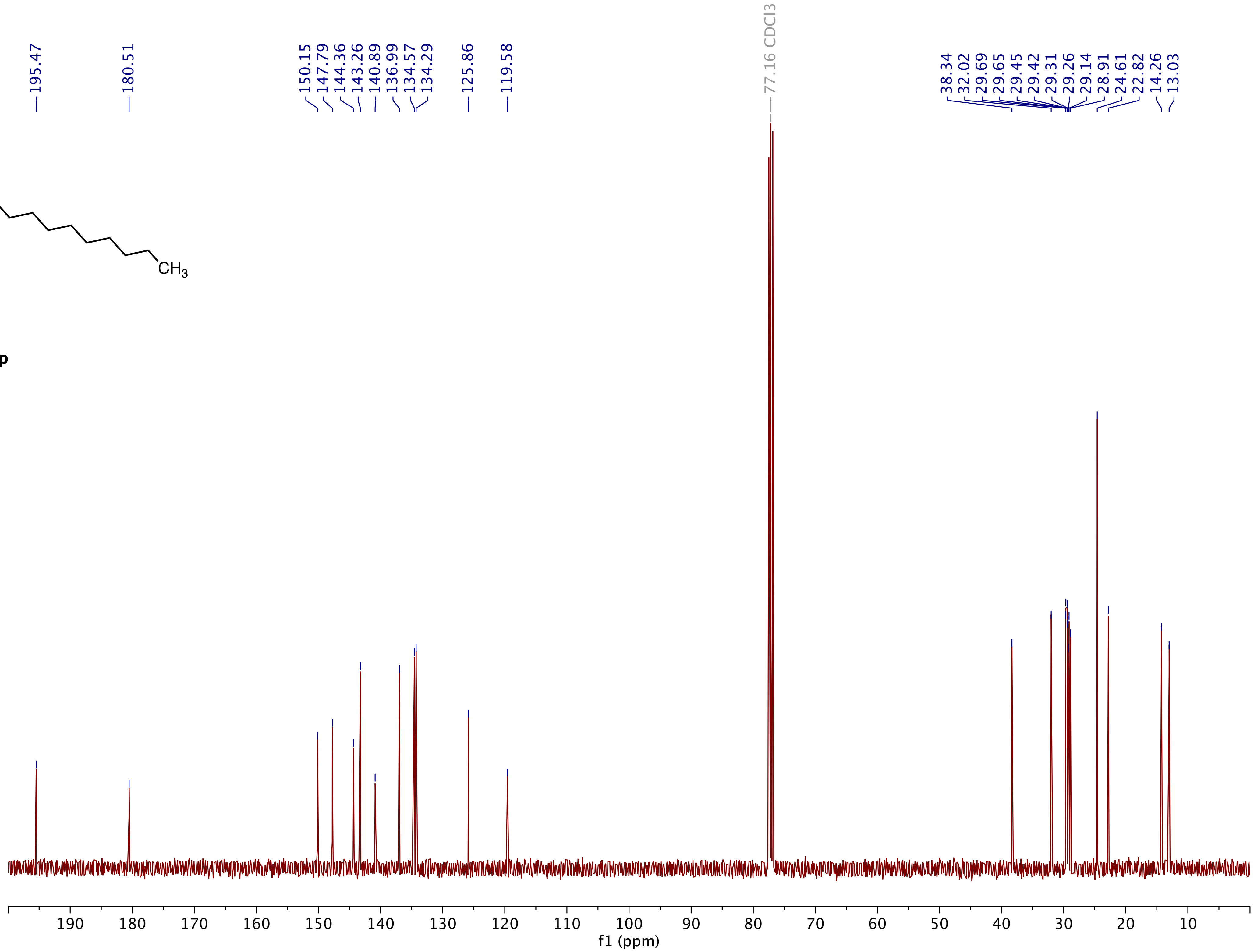

101 MHz $^{13}C\{^{1}H\}$-NMR spectrum of **7p** in $CDCl_3$

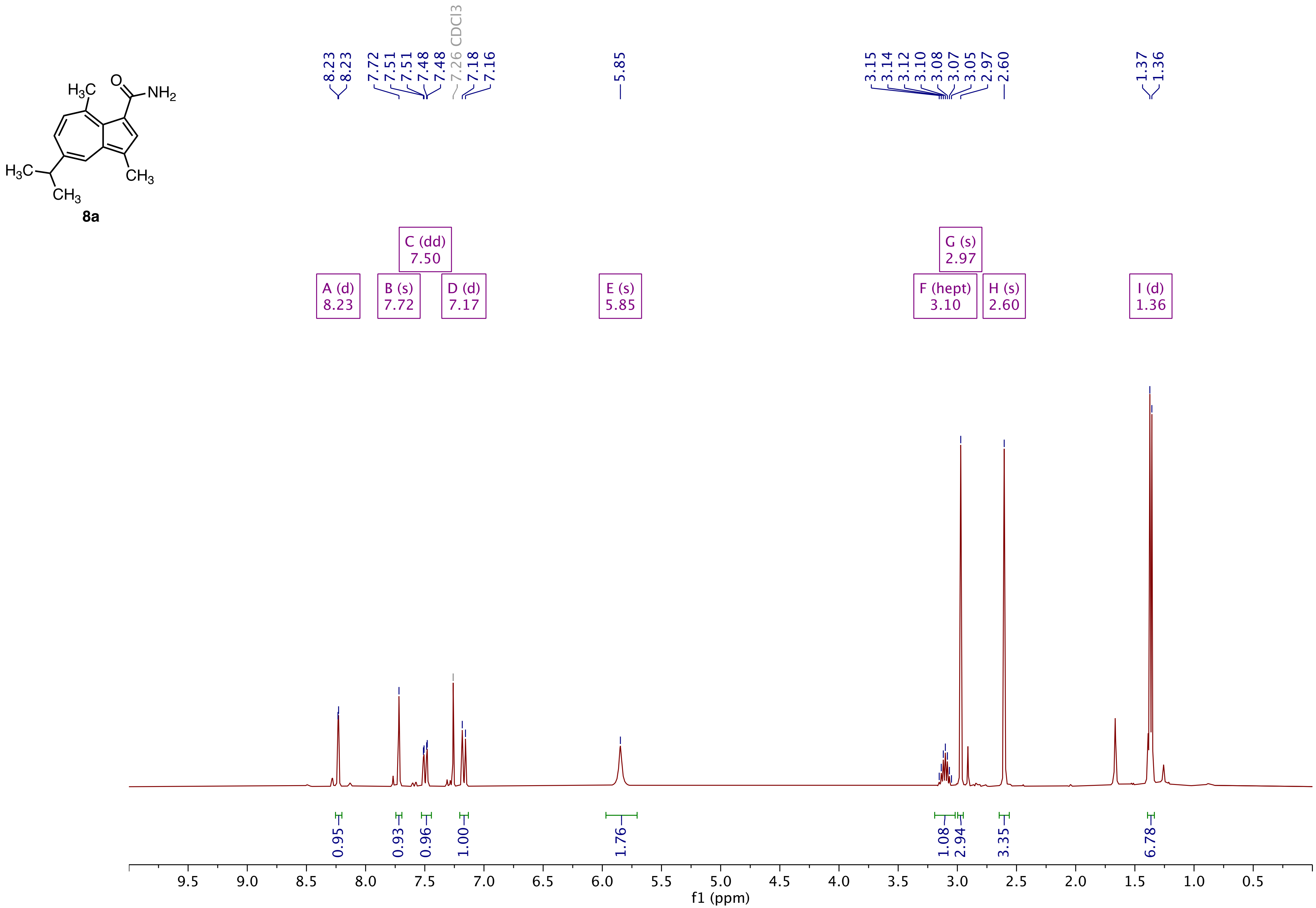

400 MHz ^{1}H-NMR spectrum of **8a** in $CDCl_3$

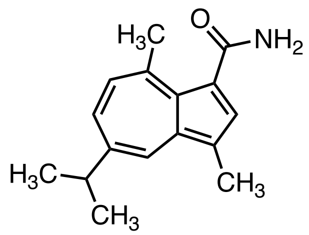

8a

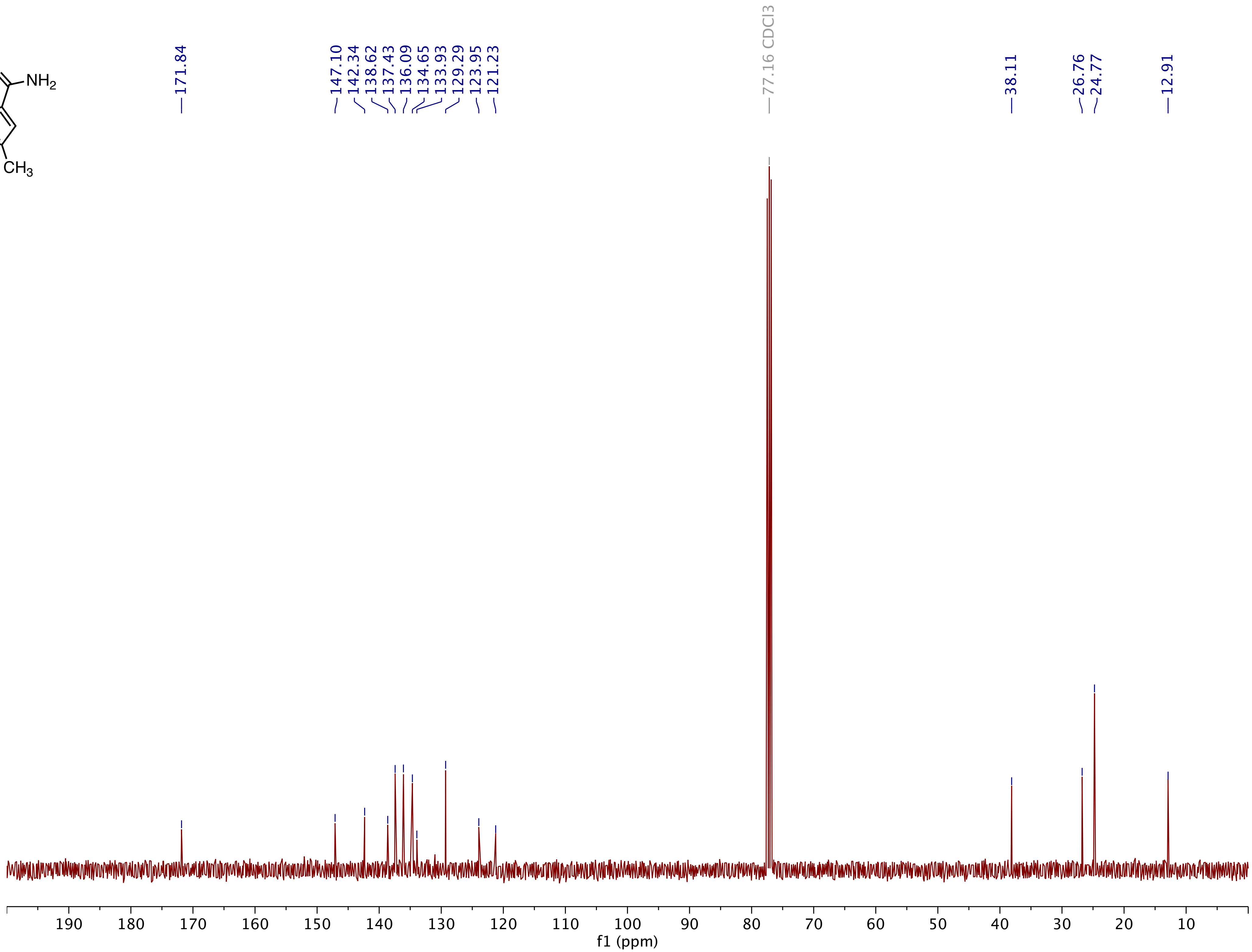

101 MHz $^{13}C\{^{1}H\}$-NMR spectrum of **8a** in $CDCl_3$

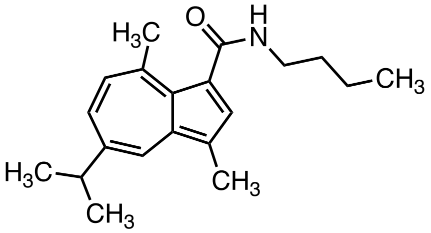

8b

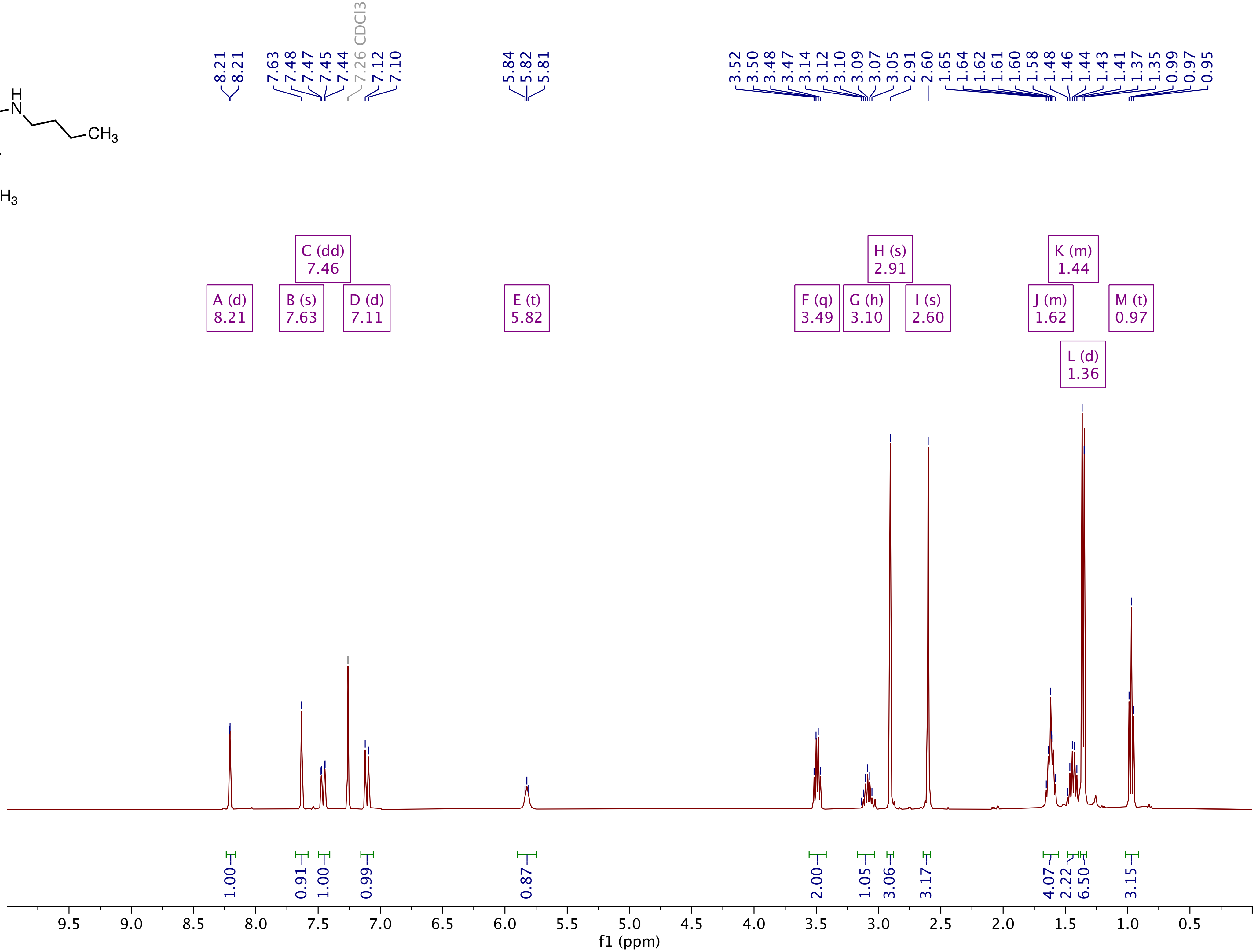

400 MHz ^{1}H-NMR spectrum of **8b** in $CDCl_3$

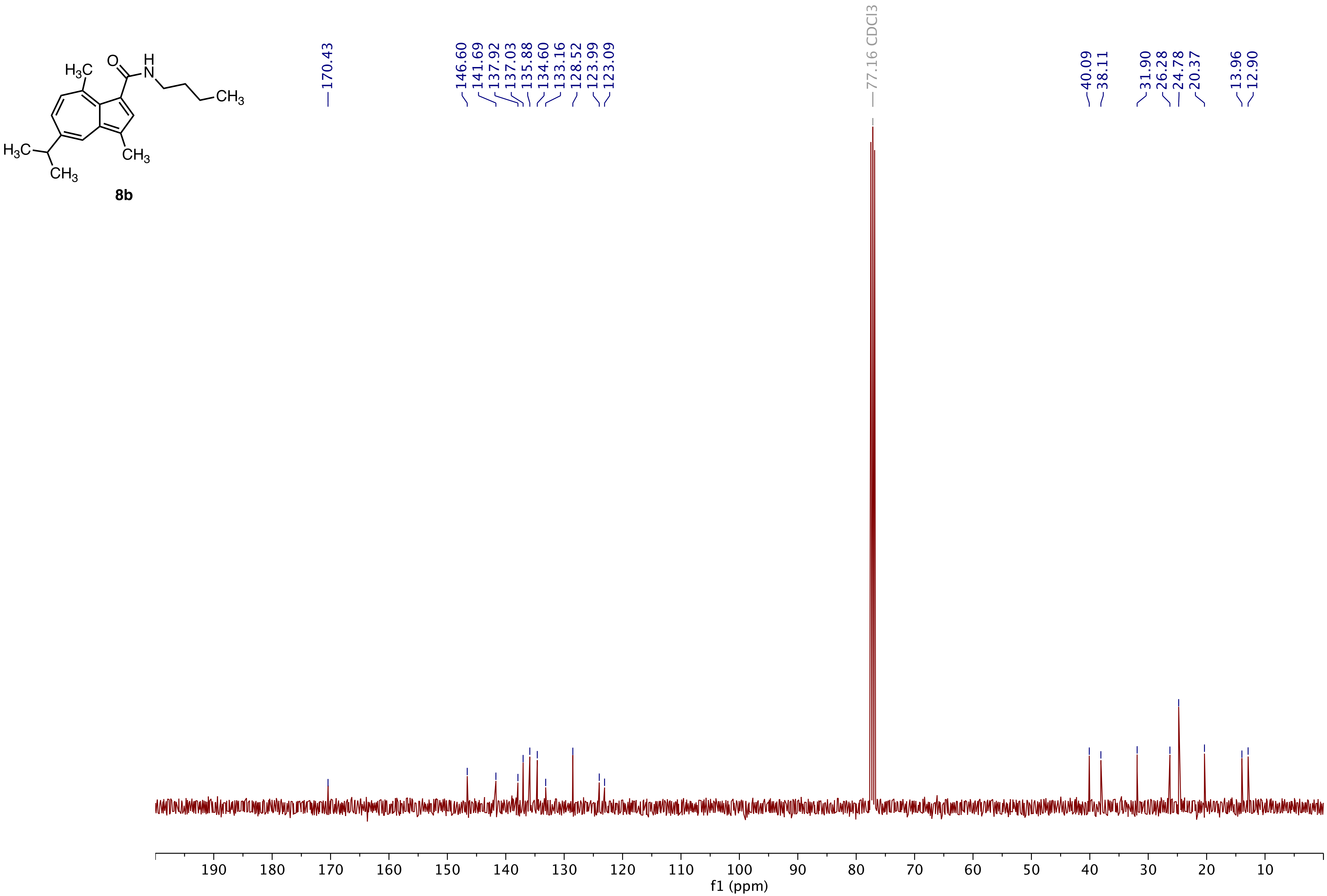

101 MHz $^{13}C\{^{1}H\}$-NMR spectrum of **8b** in $CDCl_3$

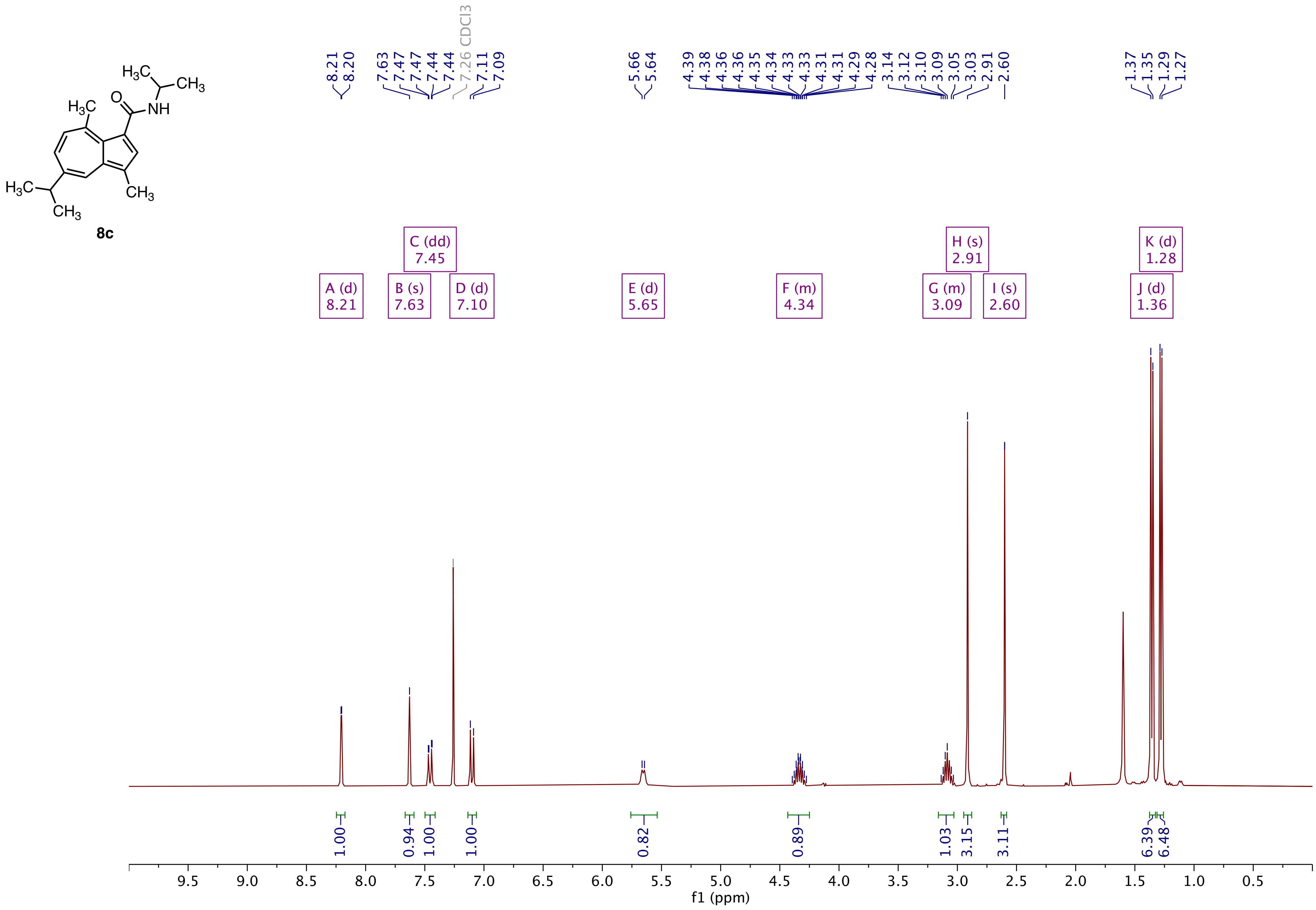

400 MHz ¹H-NMR spectrum of **8c** in $CDCl_3$

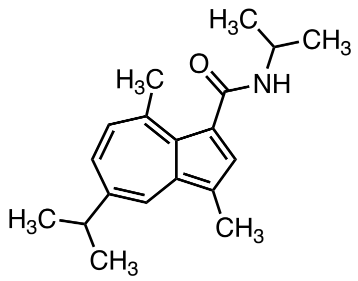

8c

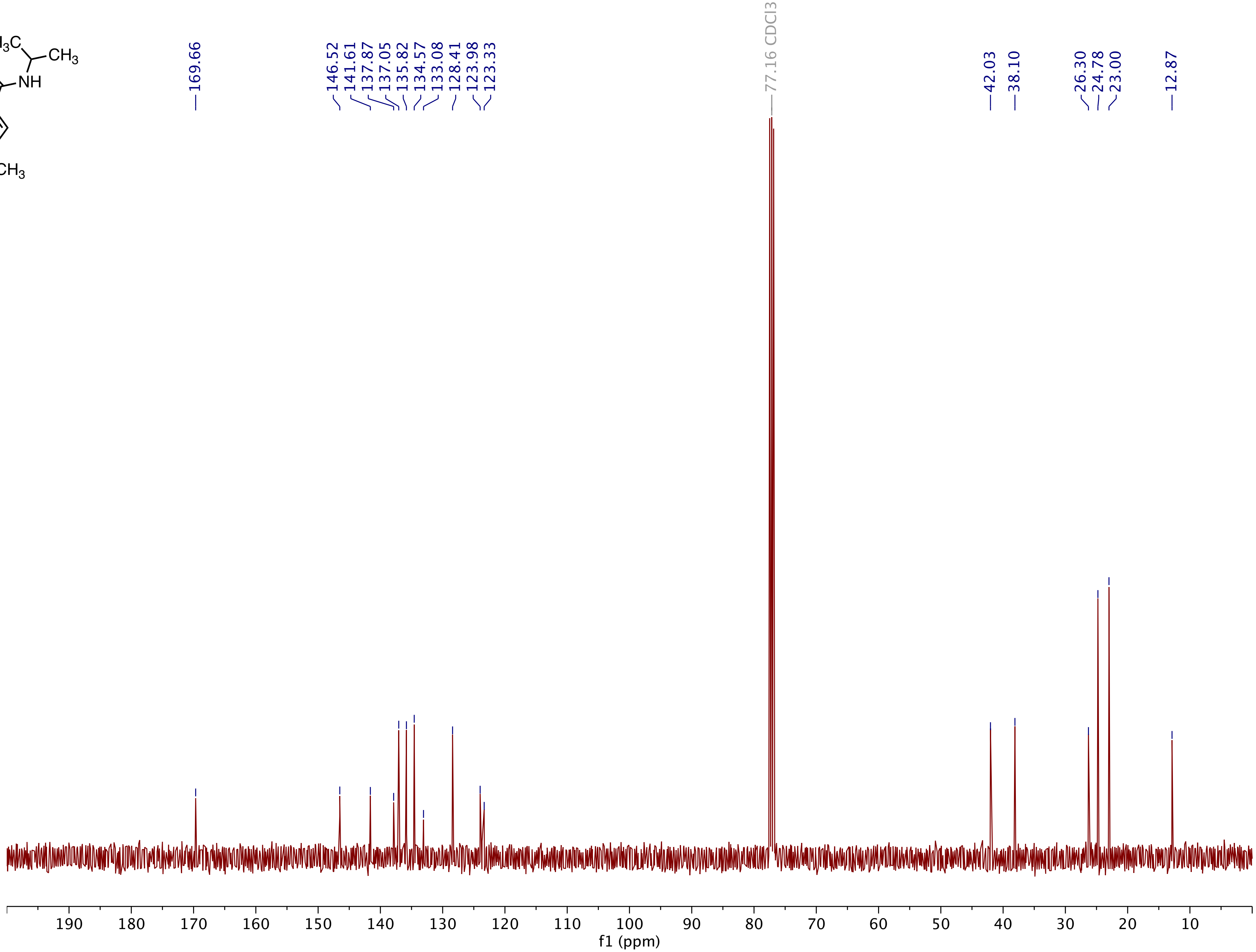

101 MHz $^{13}C\{^{1}H\}$-NMR spectrum of **8c** in $CDCl_3$

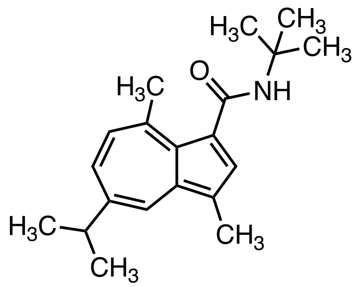

8d

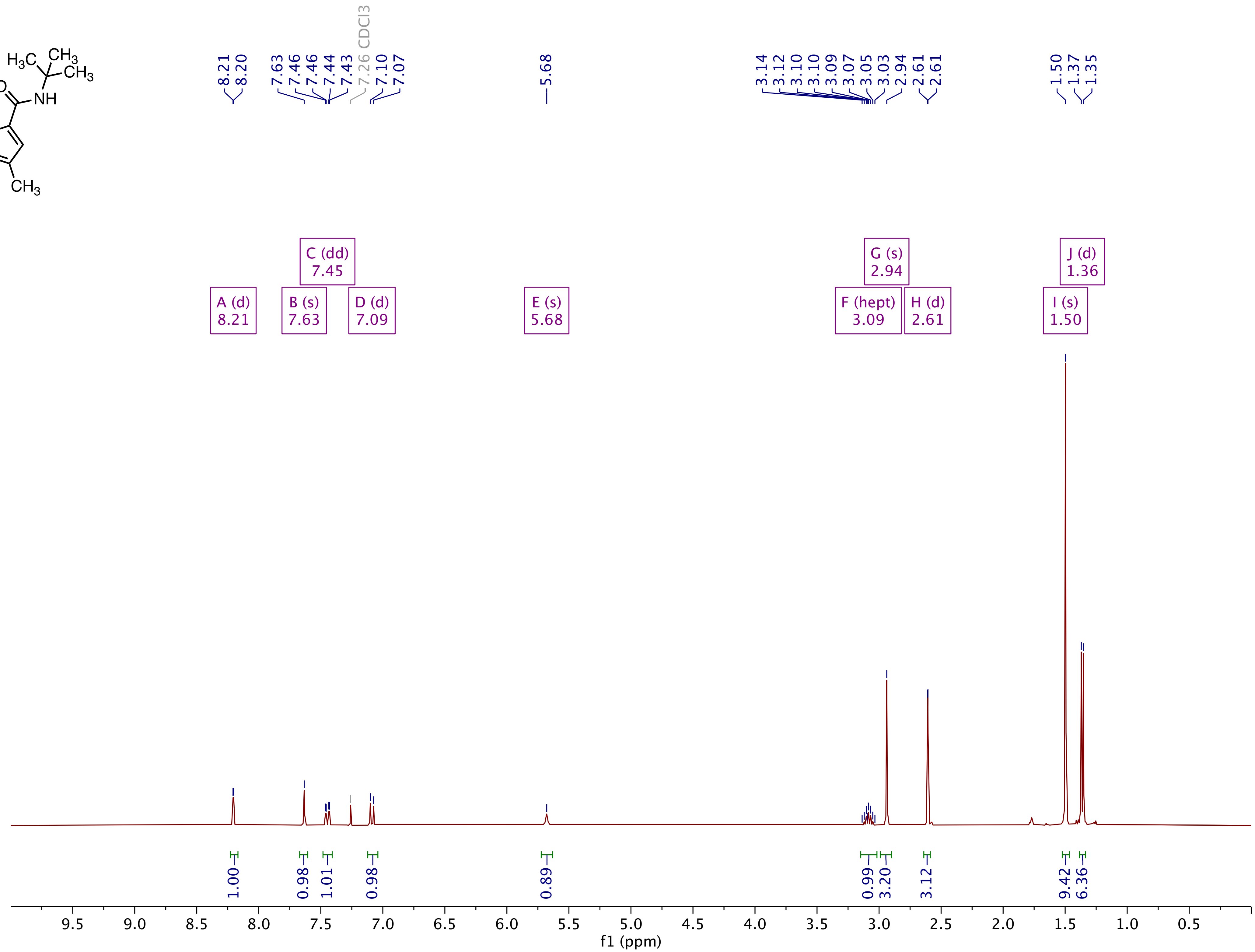

400 MHz ^{1}H-NMR spectrum of **8d** in $CDCl_3$

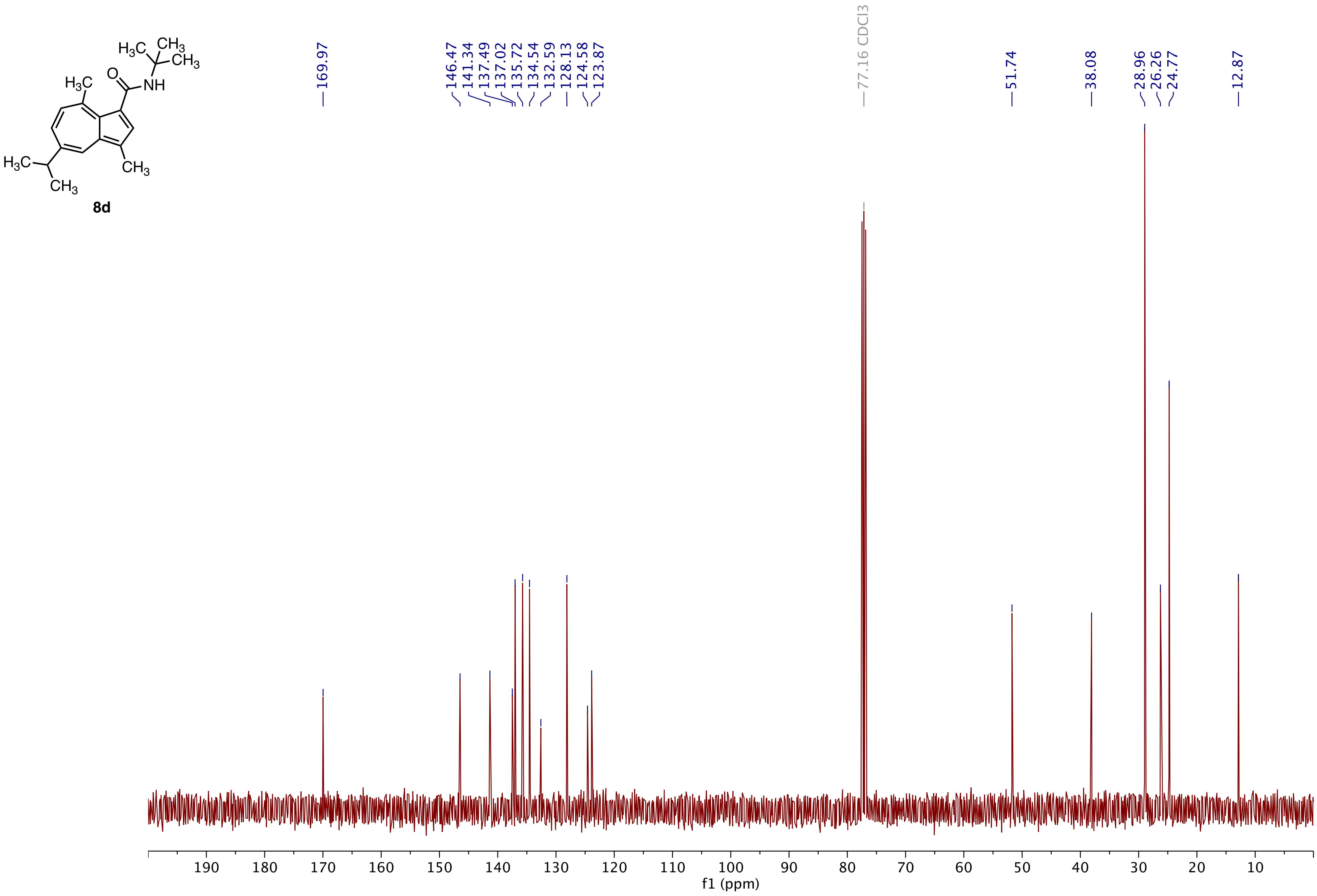

101 MHz $^{13}C\{^{1}H\}$-NMR spectrum of **8d** in $CDCl_3$

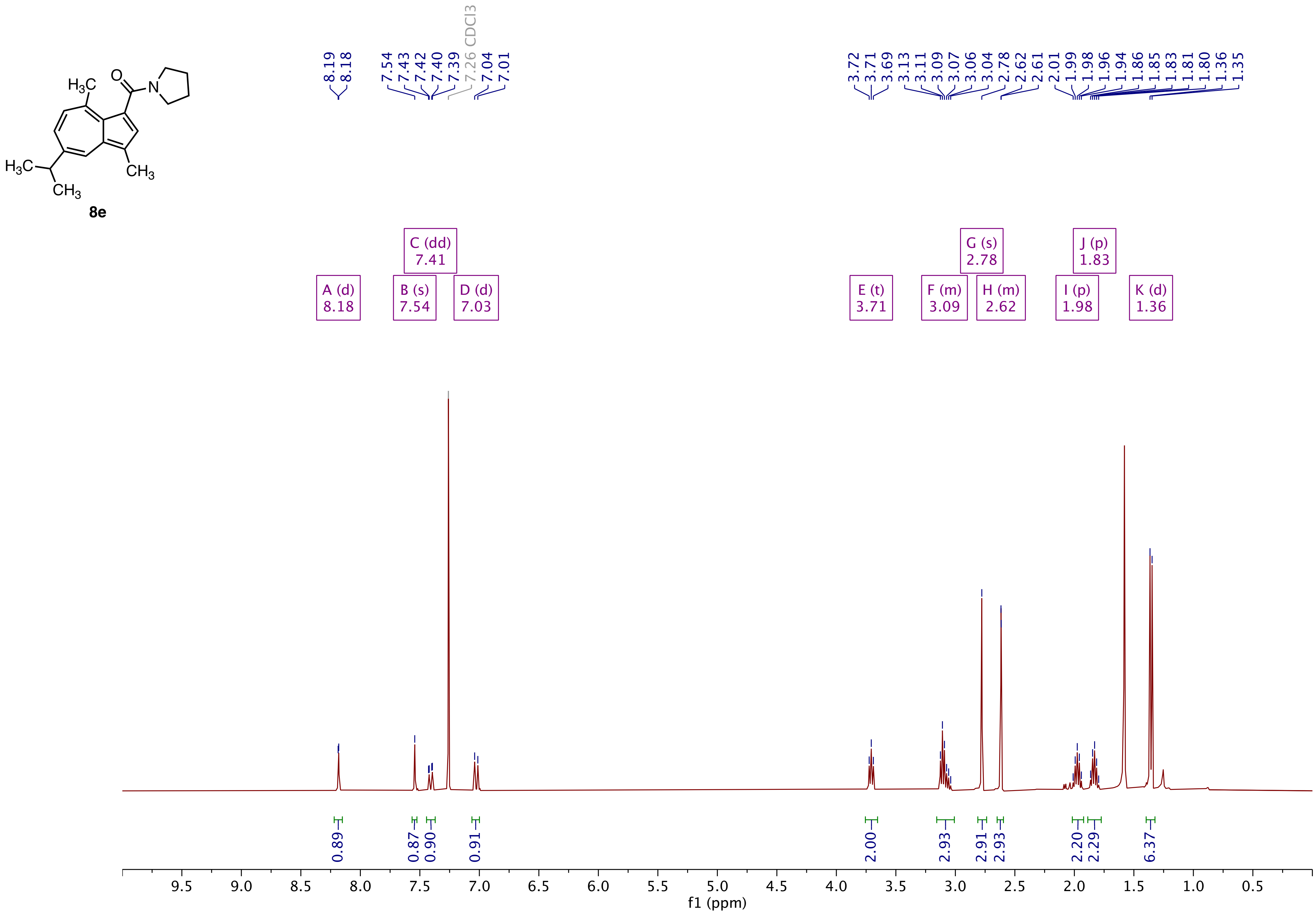

400 MHz ^{1}H-NMR spectrum of **8e** in $CDCl_3$

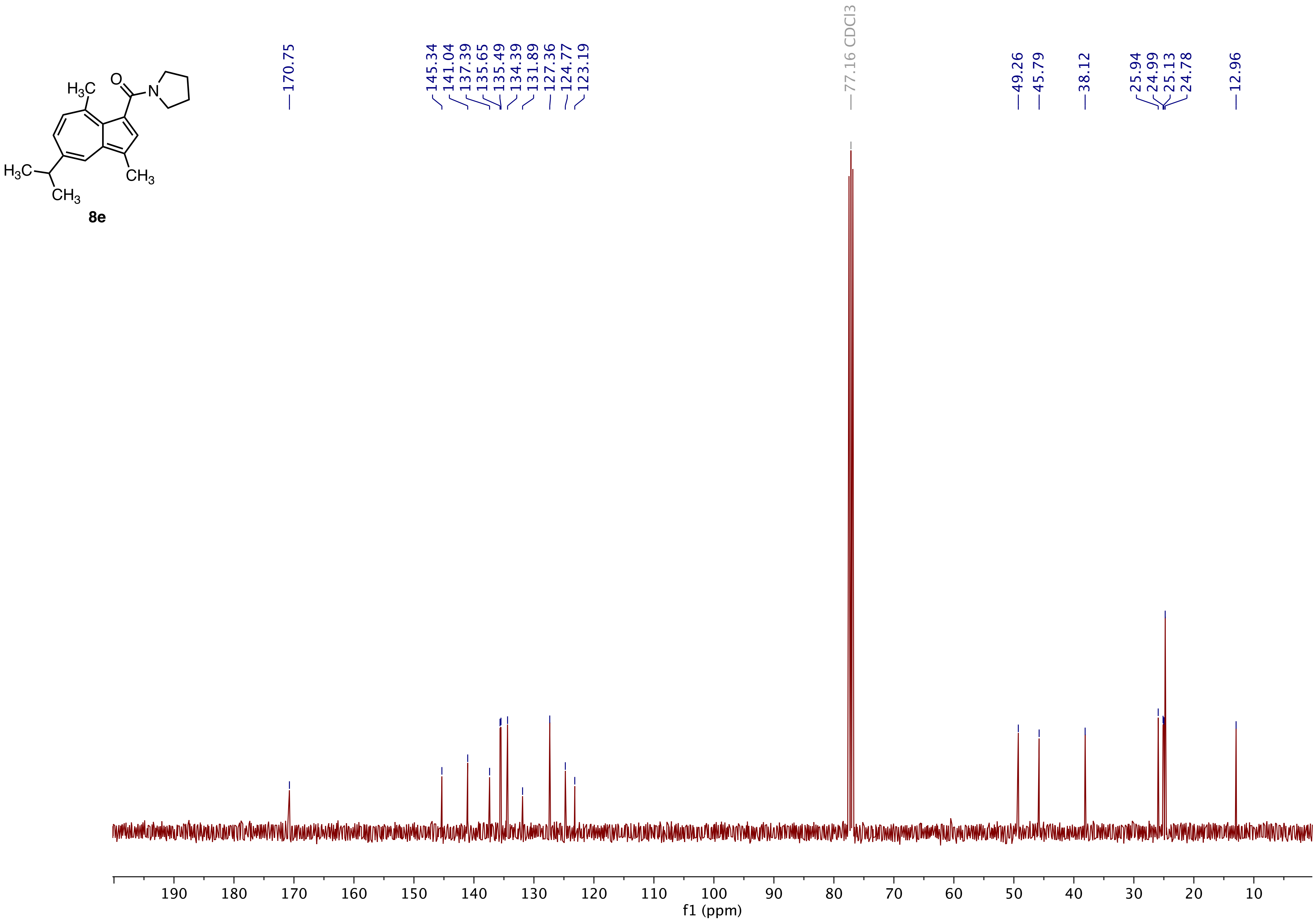

101 MHz $^{13}C\{^{1}H\}$-NMR spectrum of **8e** in $CDCl_3$

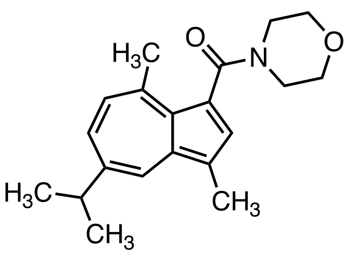

8f

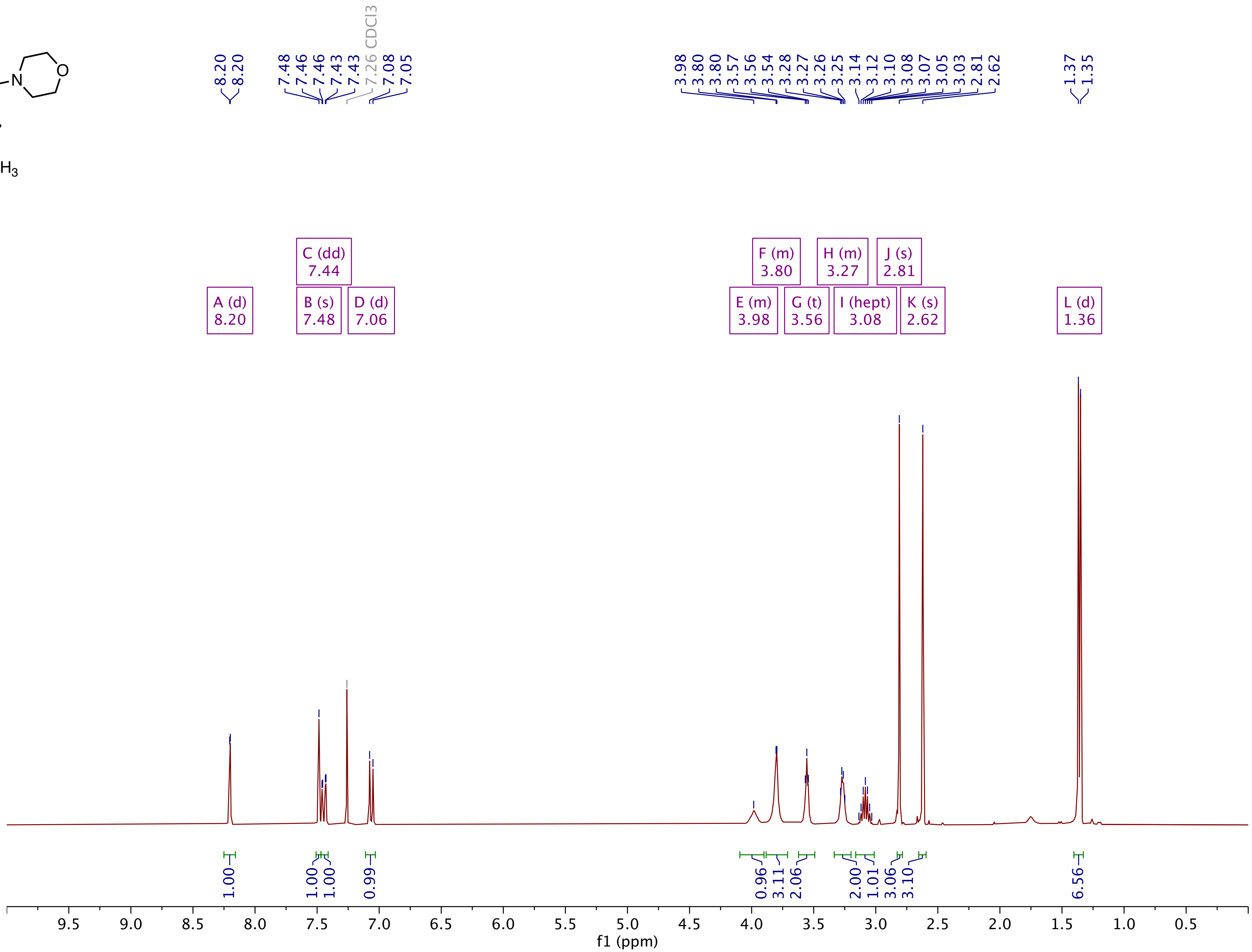

400 MHz ^{1}H-NMR spectrum of **8f** in $CDCl_3$

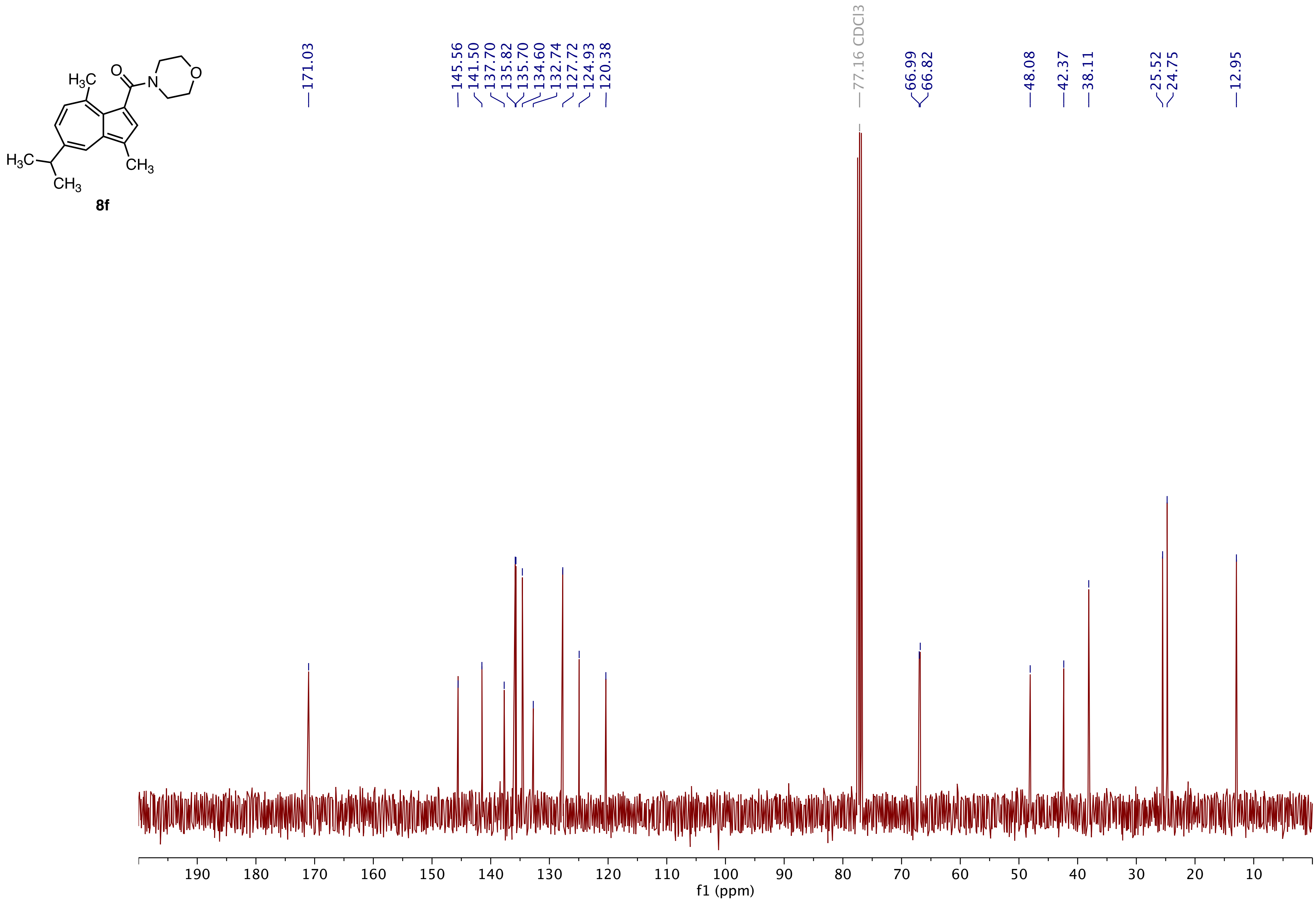

101 MHz $^{13}C\{^{1}H\}$-NMR spectrum of **8f** in $CDCl_3$

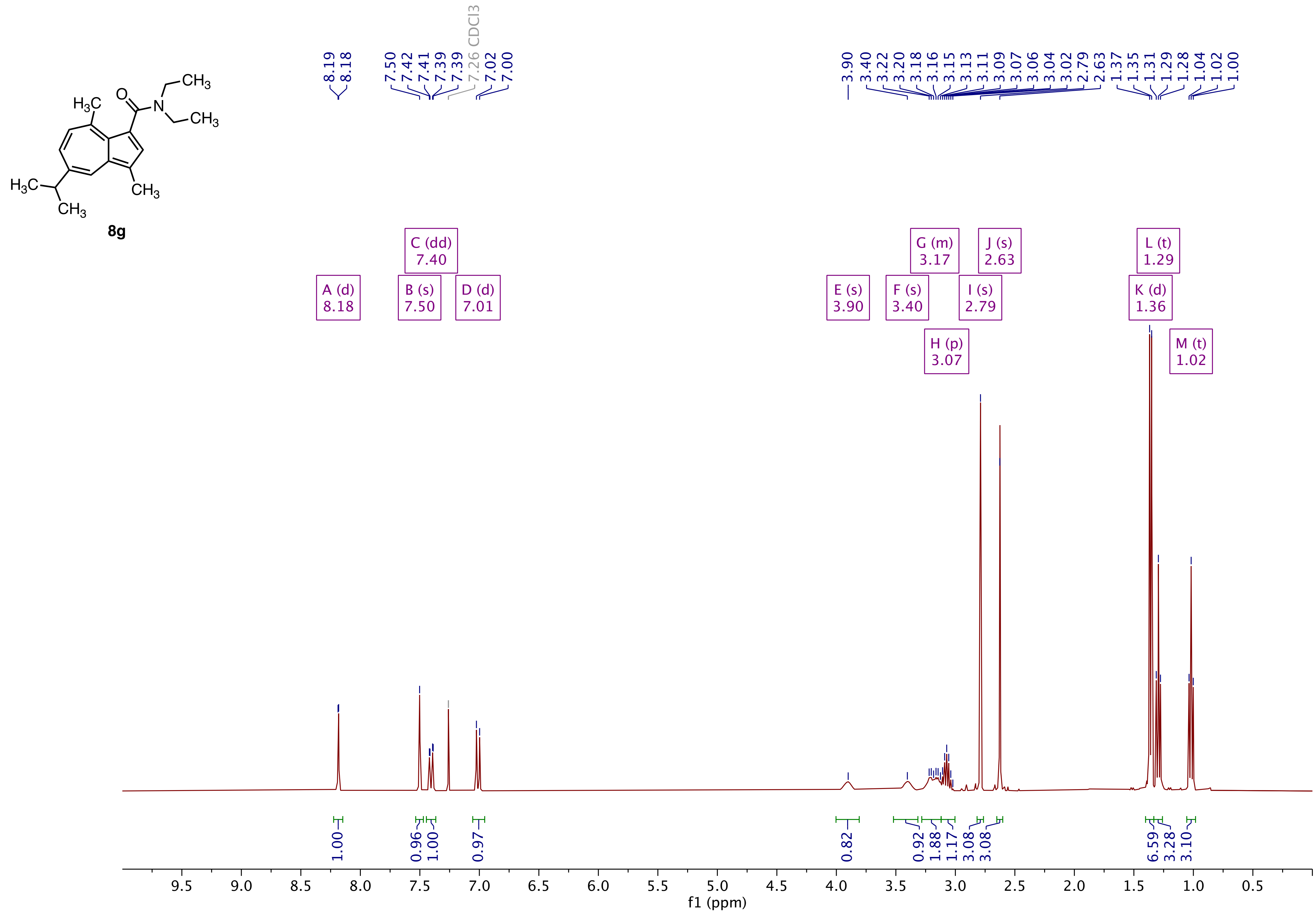

400 MHz ^{1}H-NMR spectrum of **8g** in $CDCl_3$

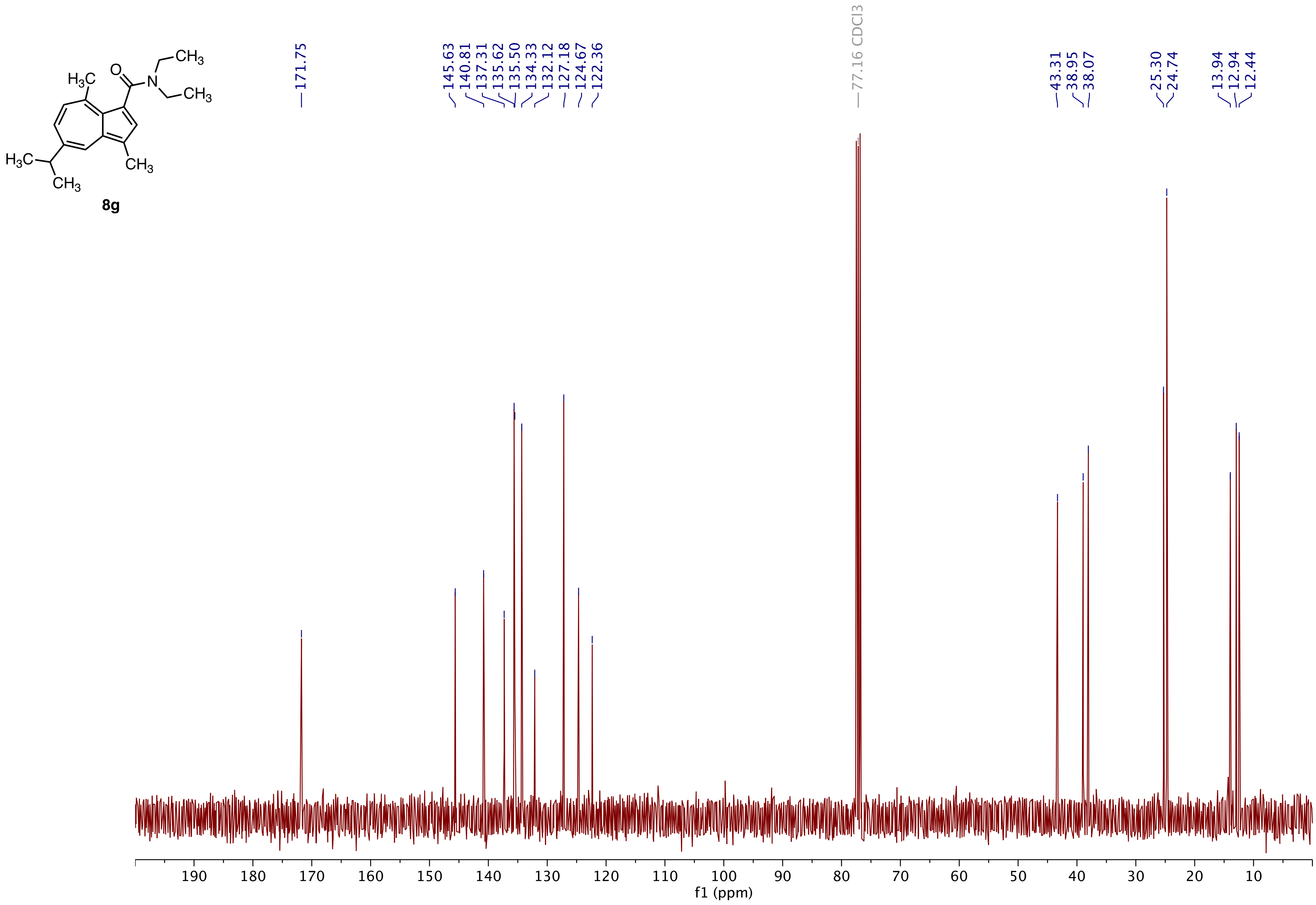

101 MHz $^{13}C\{^{1}H\}$-NMR spectrum of **8g** in $CDCl_3$

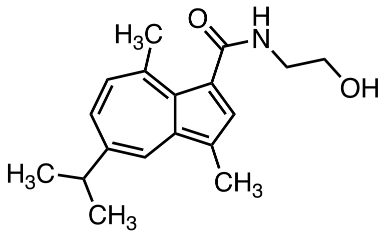

8h

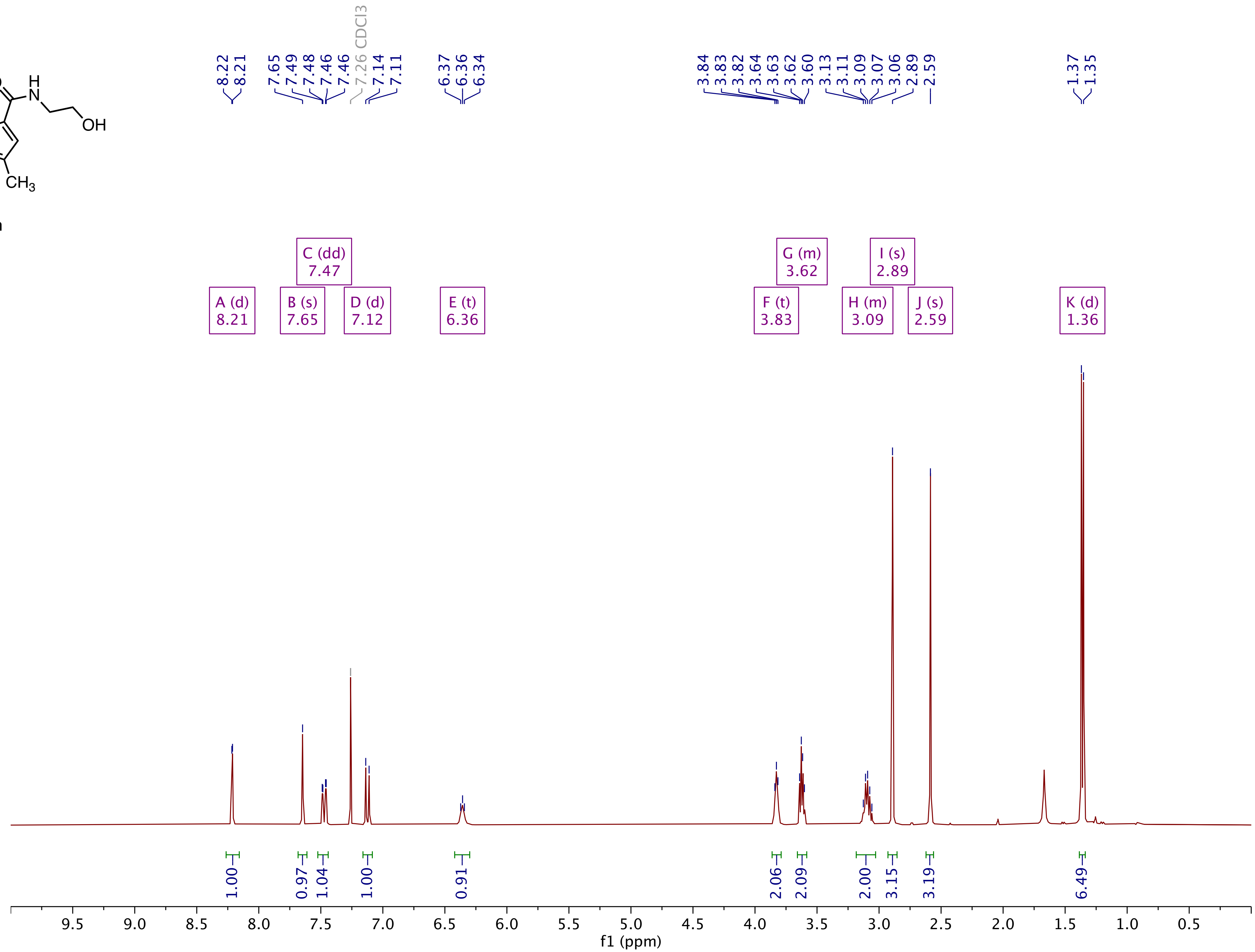

400 MHz ^{1}H-NMR spectrum of **8h** in $CDCl_3$

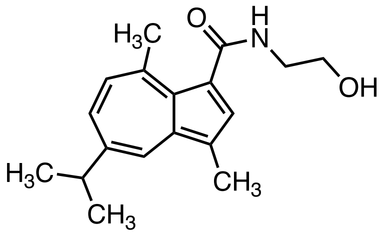

8h

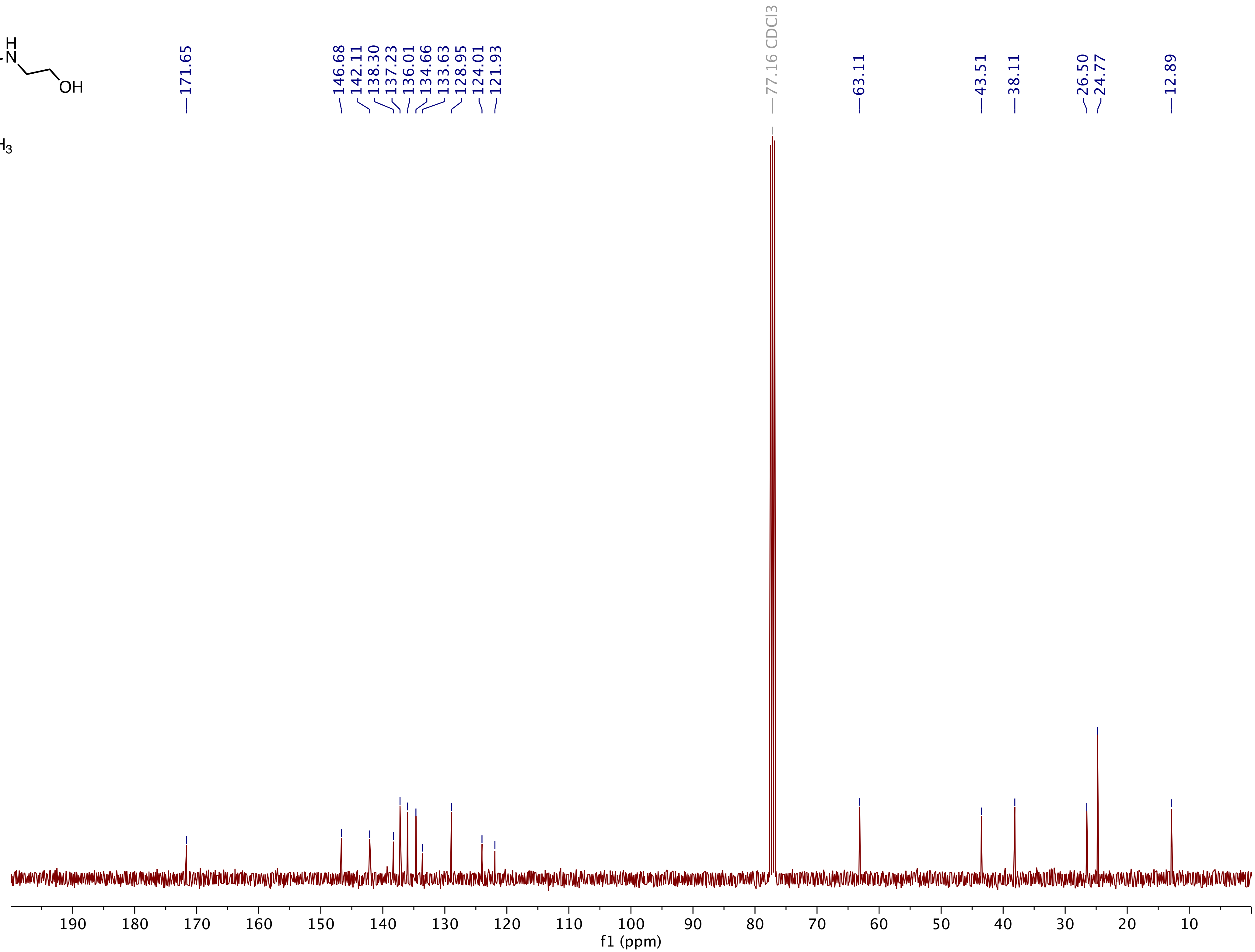

101 MHz $^{13}C\{^{1}H\}$-NMR spectrum of **8h** in $CDCl_3$

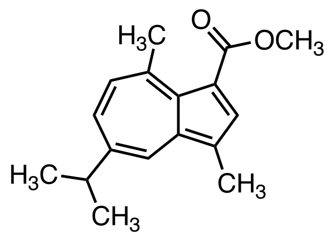

8i

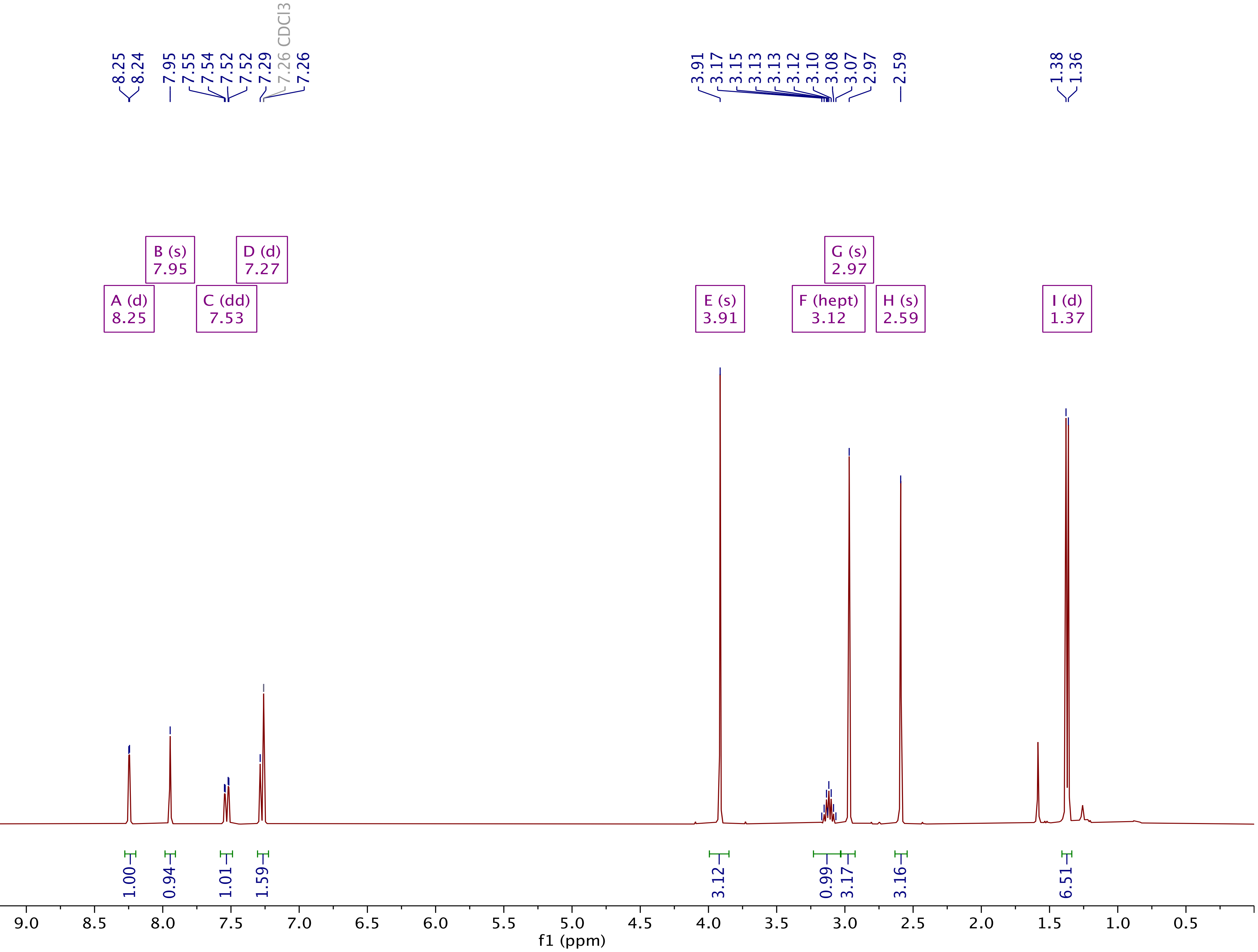

400 MHz ^{1}H-NMR spectrum of **8i** in $CDCl_3$

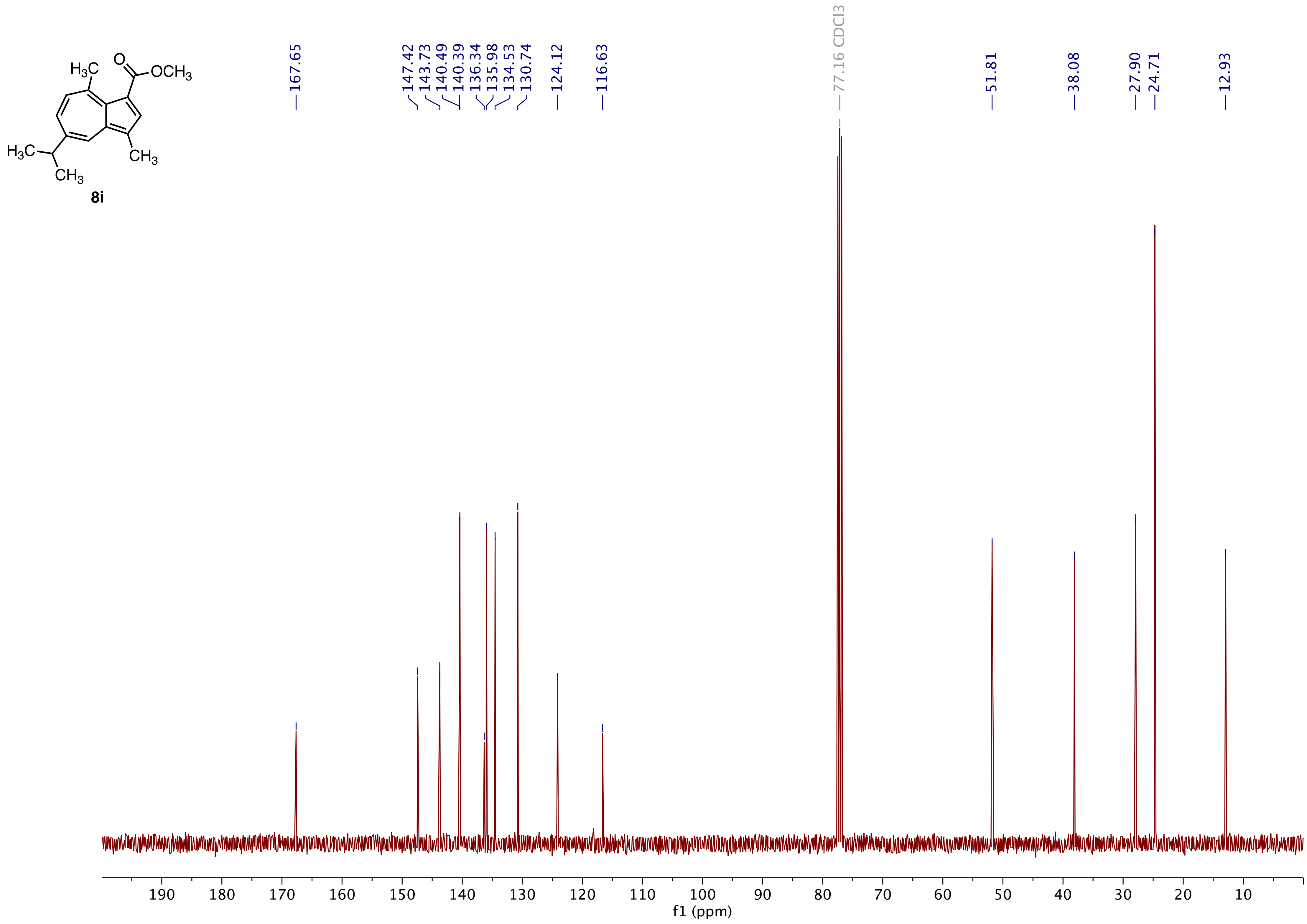

101 MHz $^{13}C\{^{1}H\}$-NMR spectrum of **8i** in $CDCl_3$

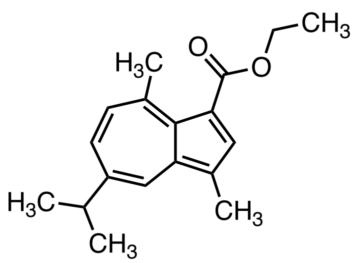

8j

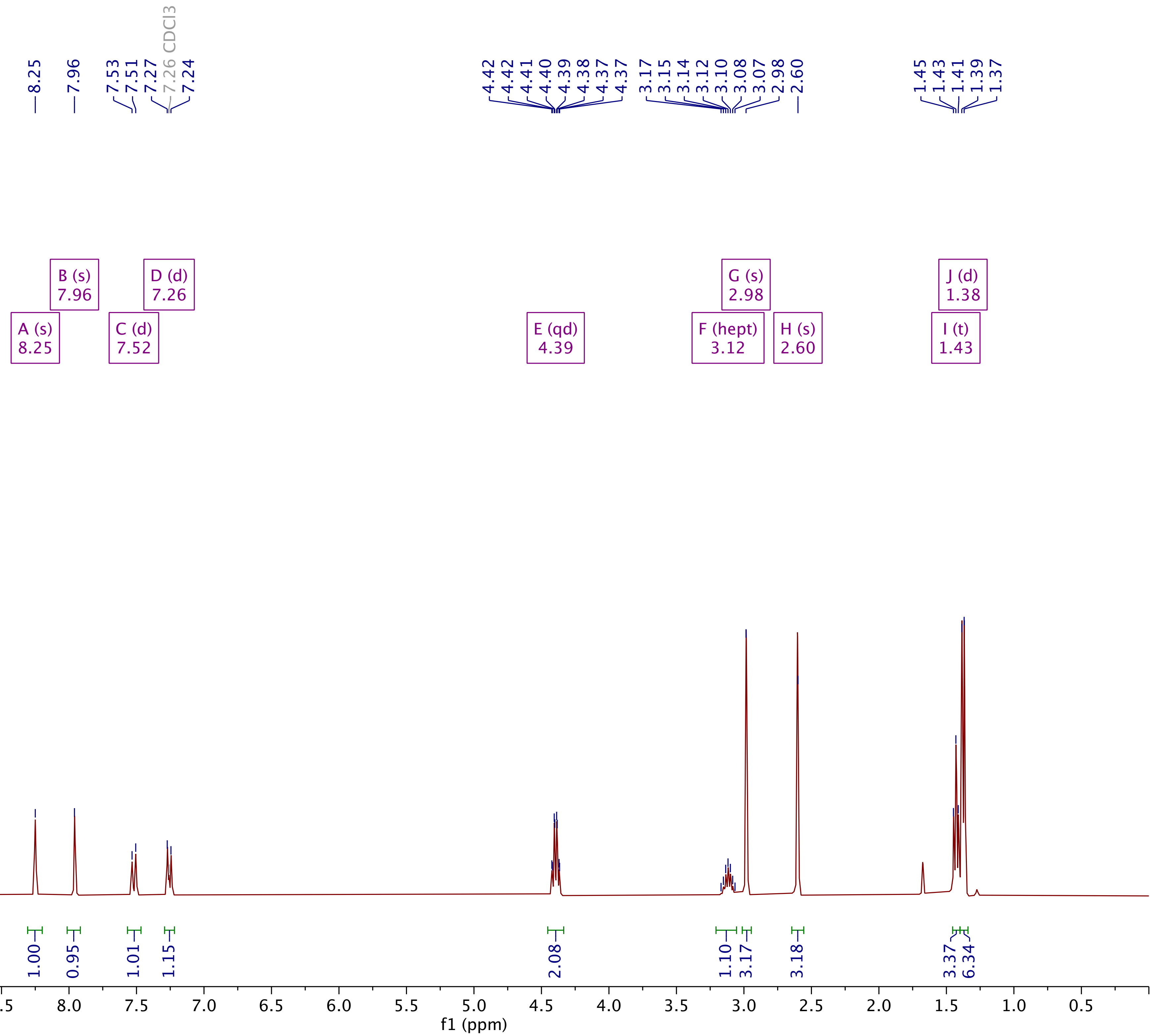

400 MHz ^{1}H-NMR spectrum of **8j** in $CDCl_3$

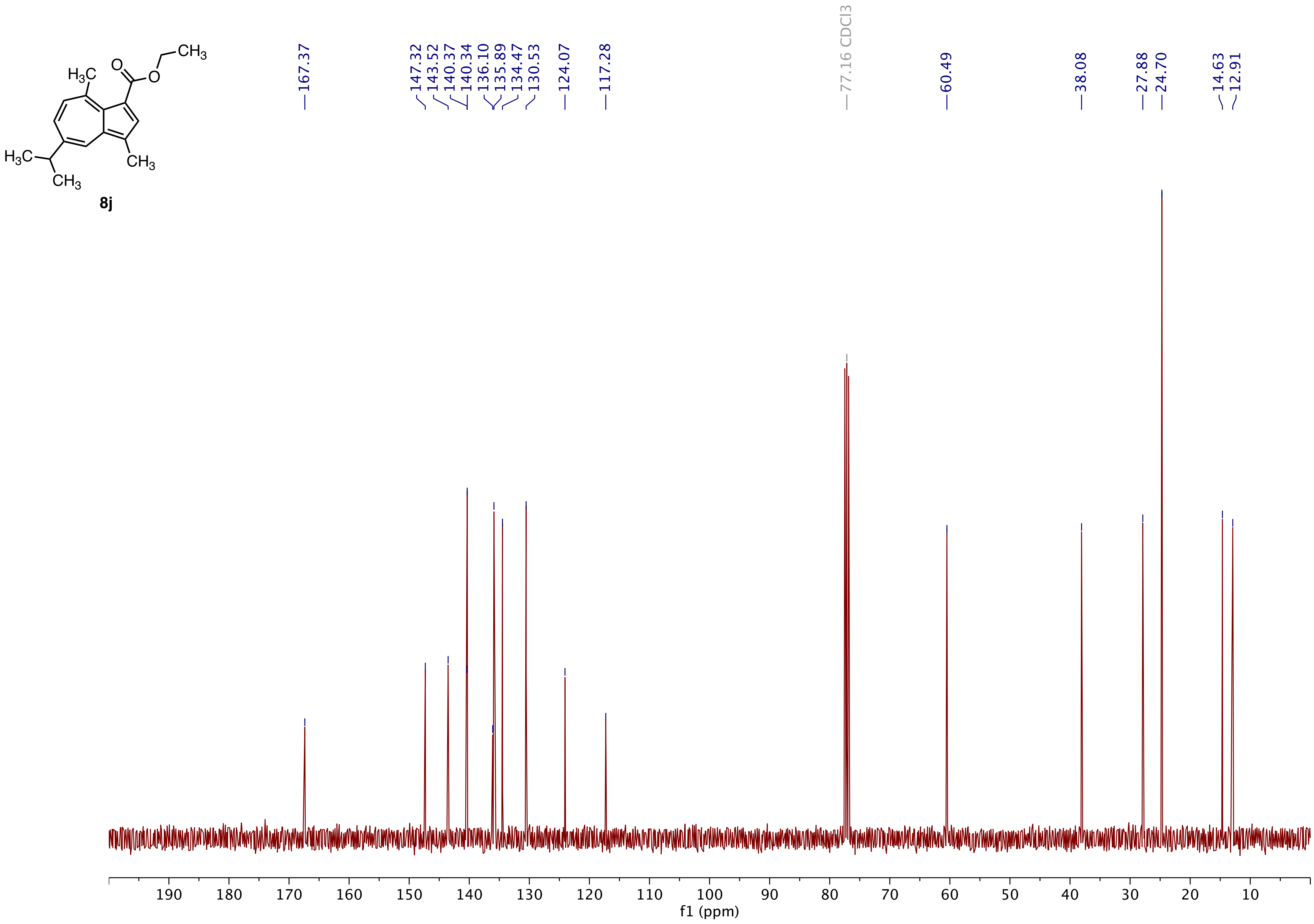

101 MHz $^{13}C\{^{1}H\}$-NMR spectrum of **8j** in $CDCl_3$

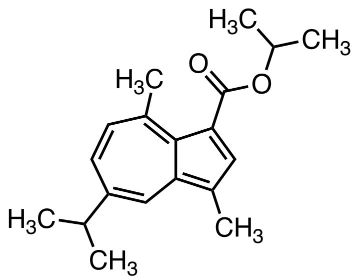

8k

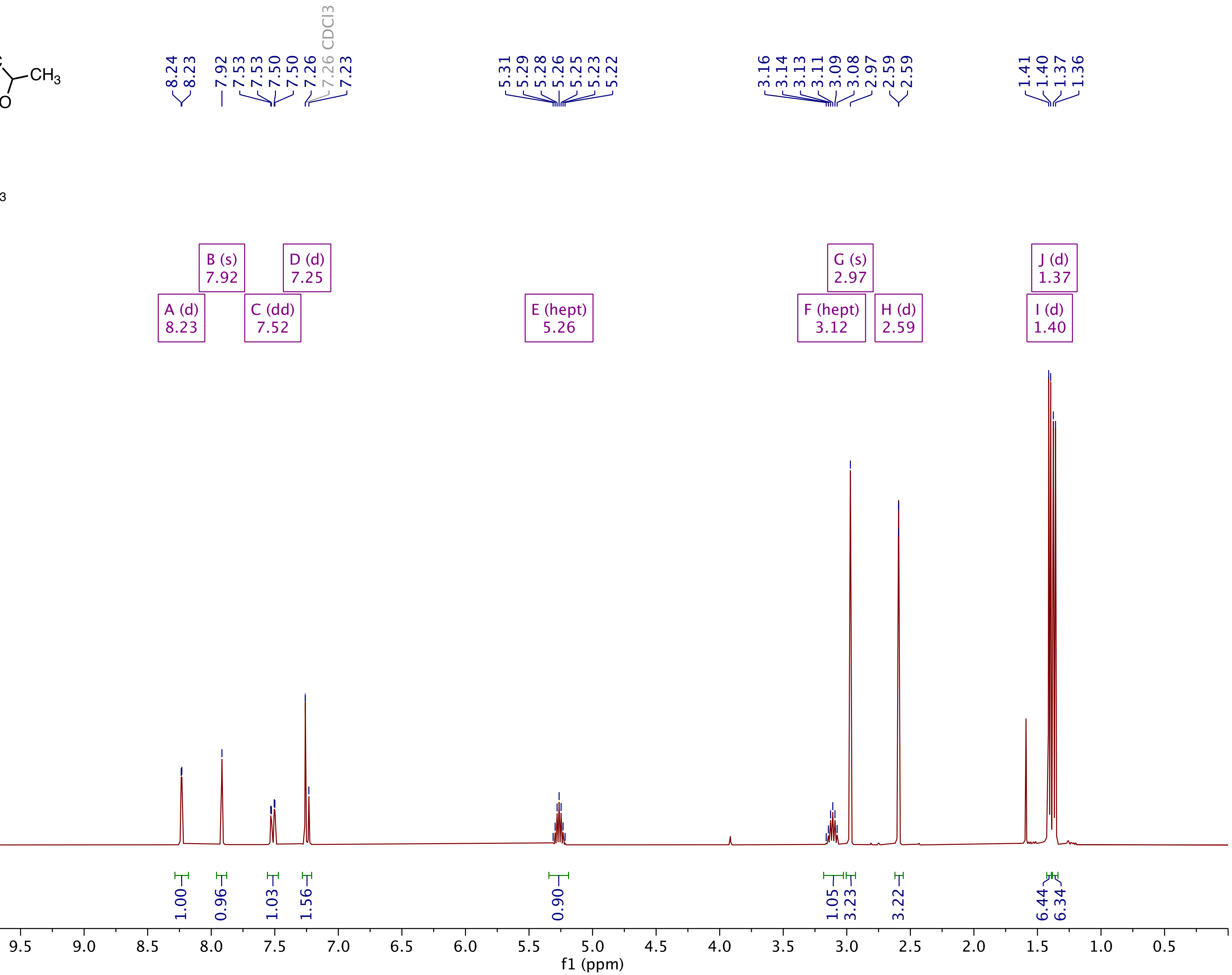

400 MHz ^{1}H-NMR spectrum of **8k** in $CDCl_3$

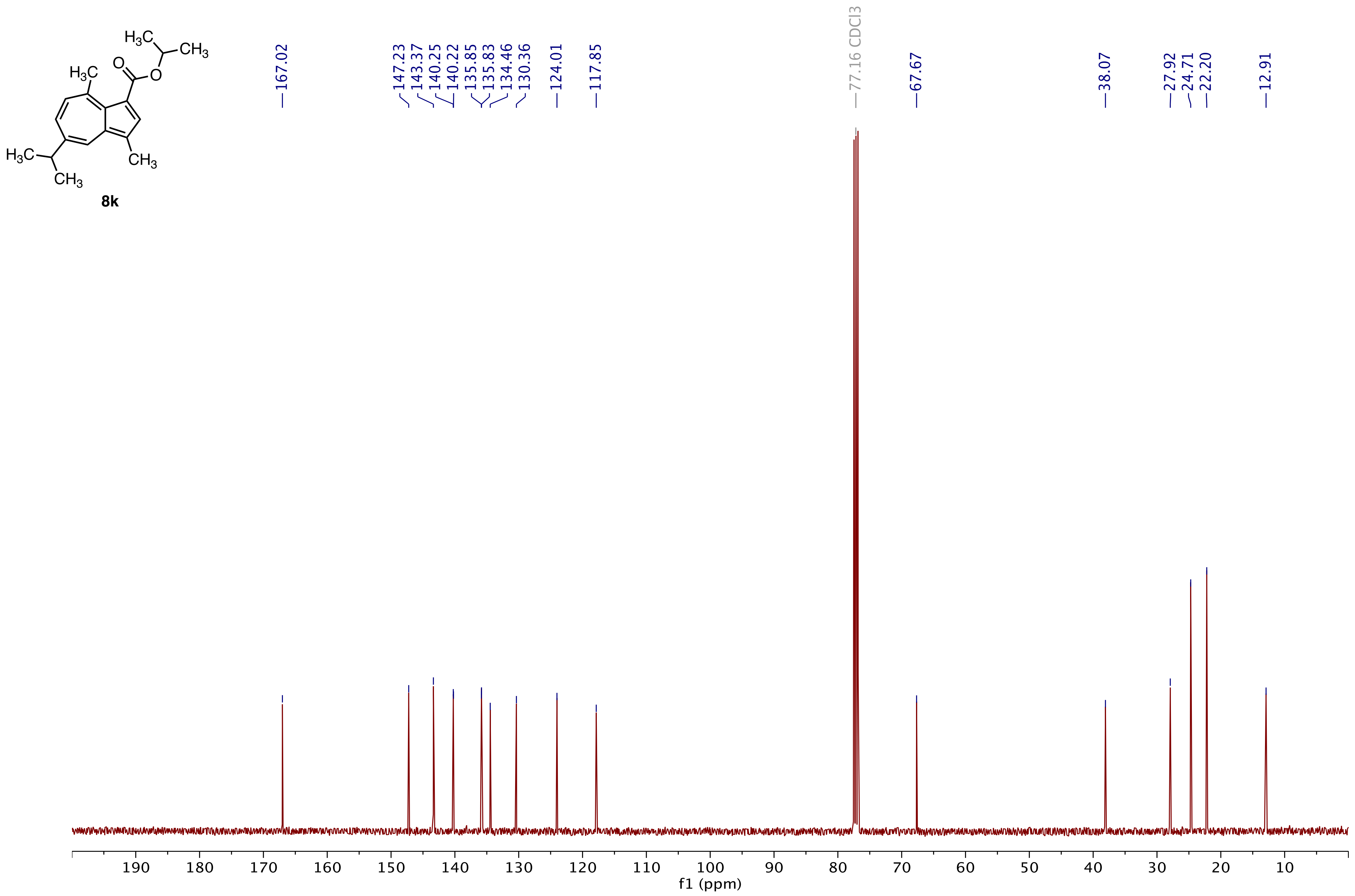

101 MHz $^{13}C\{^{1}H\}$-NMR spectrum of **8k** in $CDCl_3$

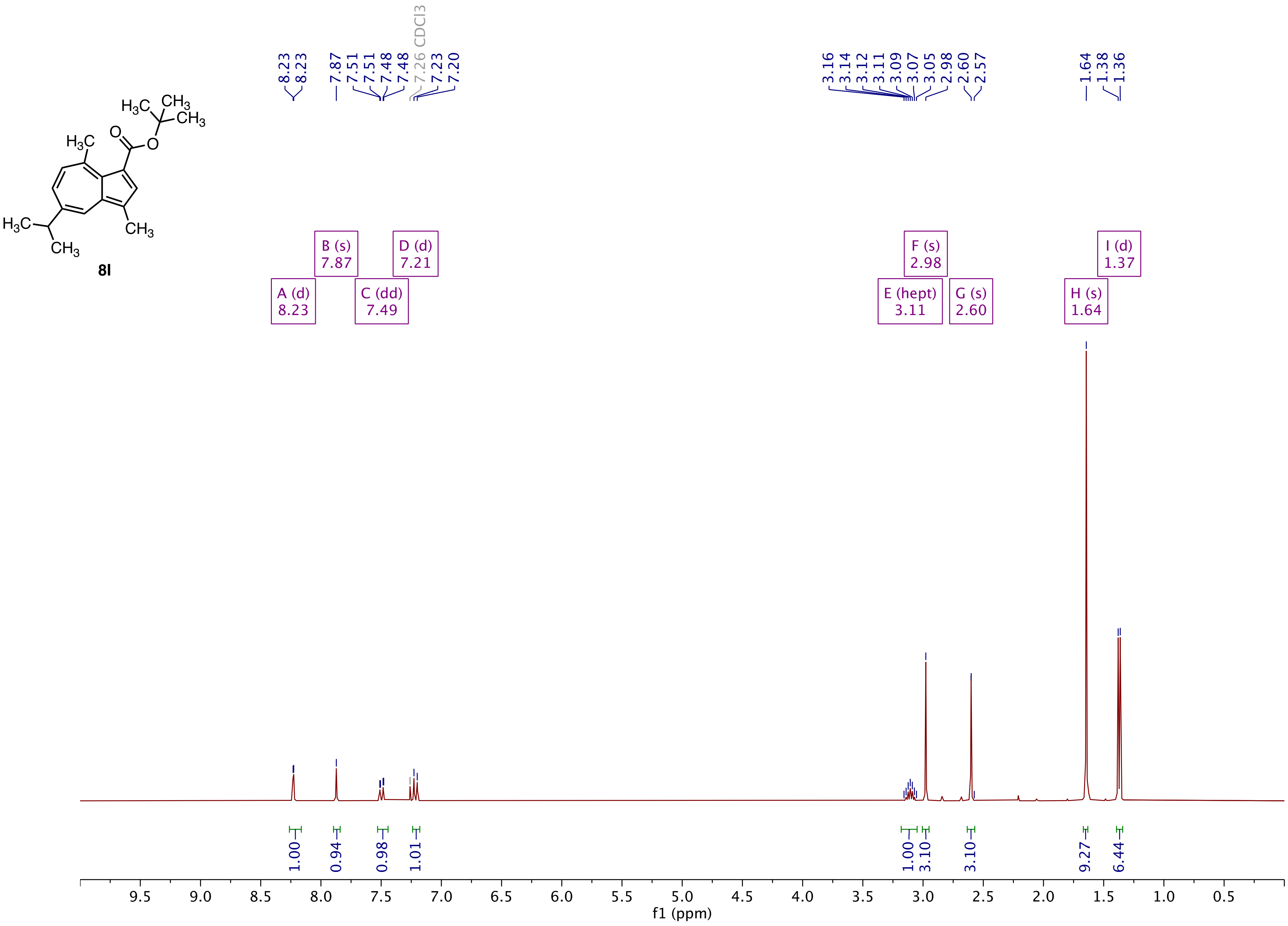

400 MHz ^{1}H-NMR spectrum of **8l** in $CDCl_3$

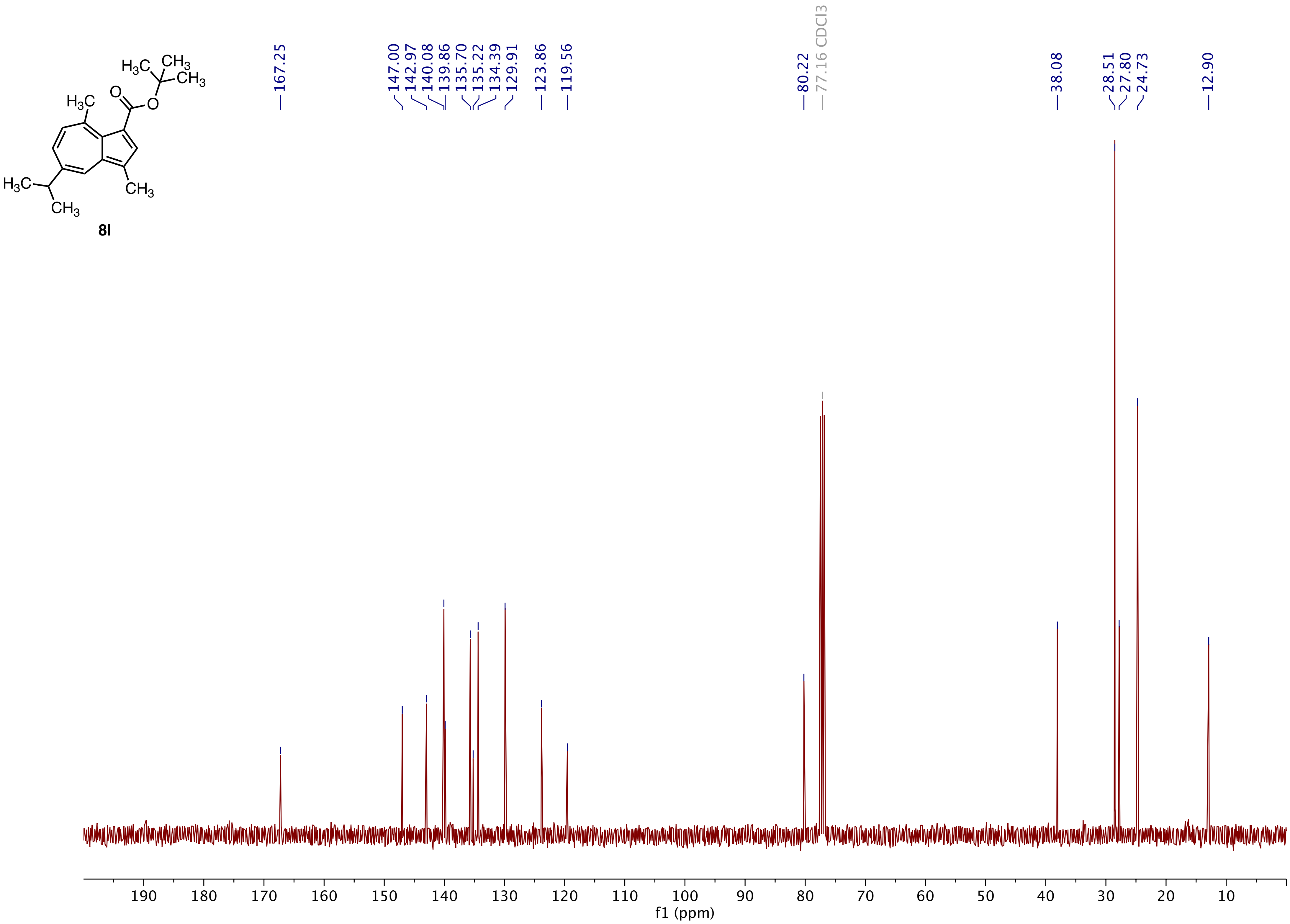

101 MHz $^{13}C\{^1H\}$-NMR spectrum of **8l** in $CDCl_3$

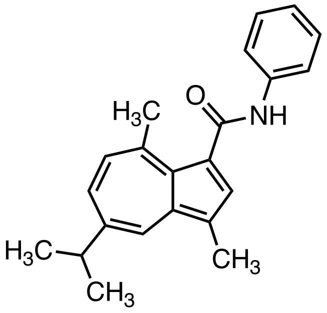

8n

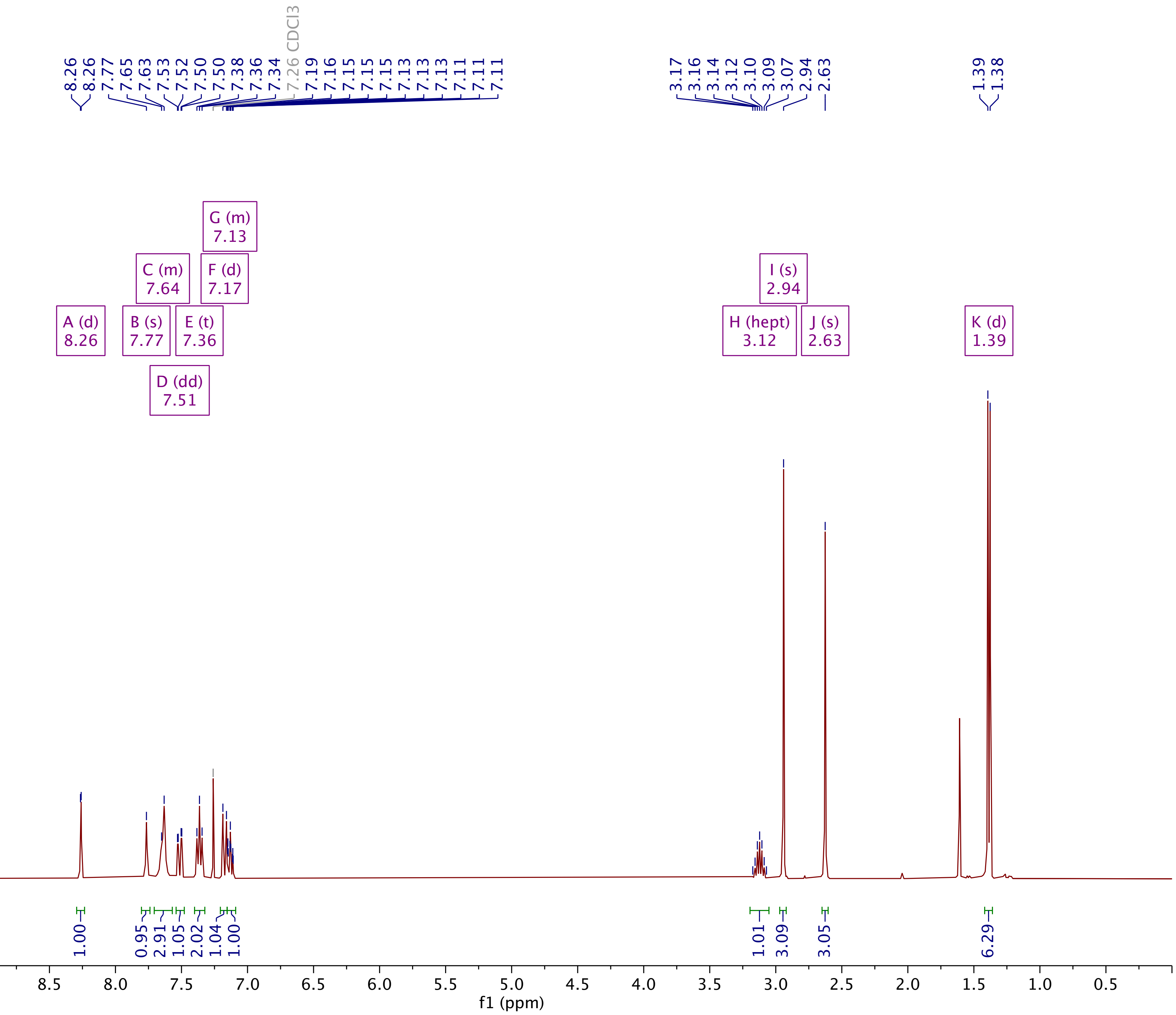

400 MHz ^{1}H-NMR spectrum of **8n** in $CDCl_3$

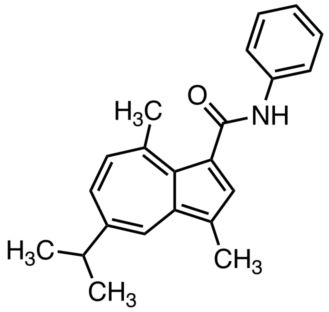

8n

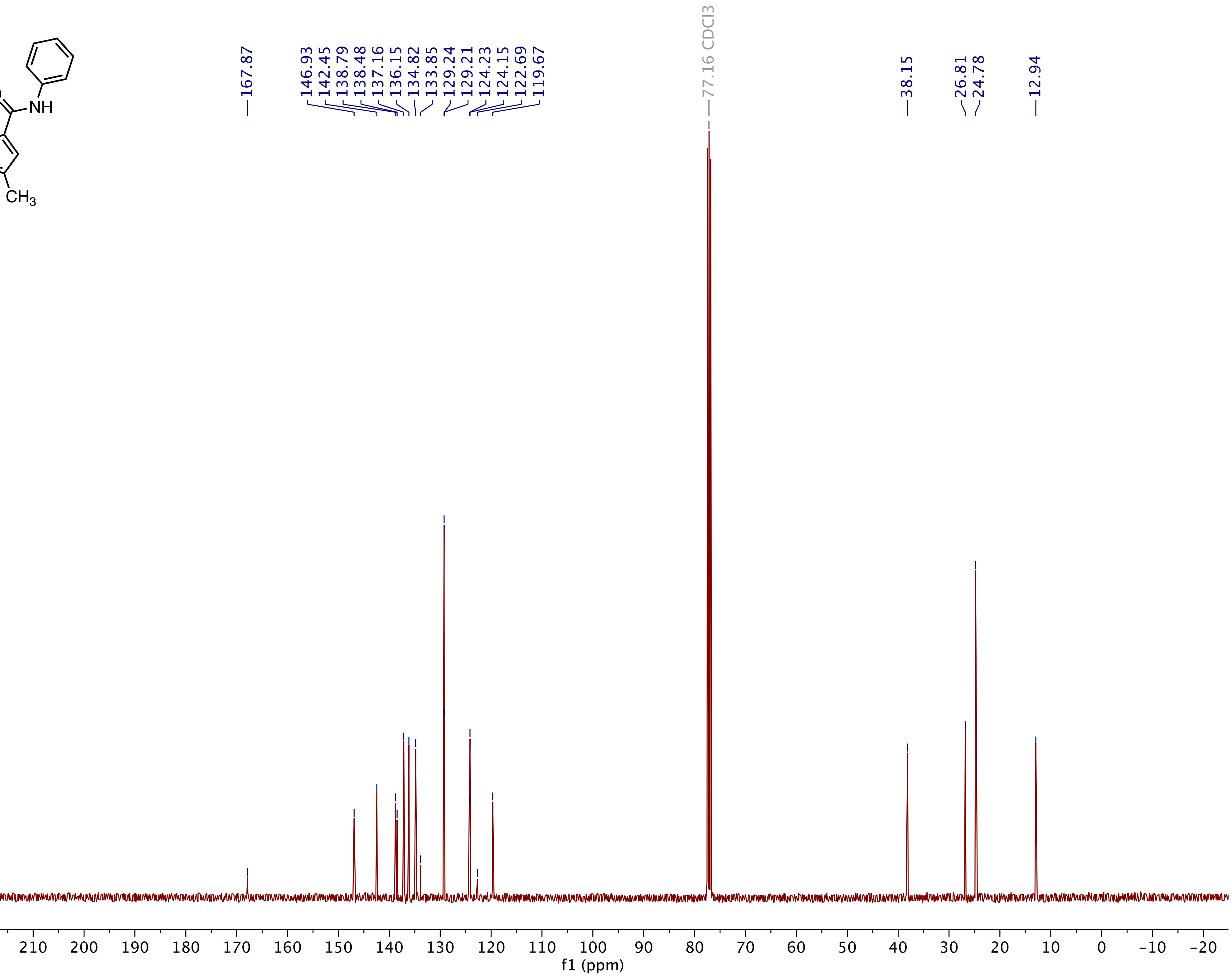

101 MHz $^{13}C\{^{1}H\}$-NMR spectrum of **8n** in $CDCl_3$

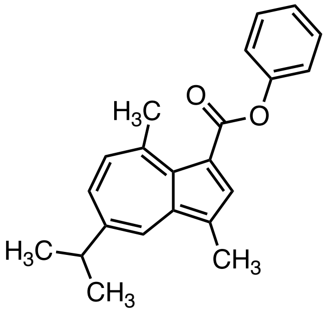

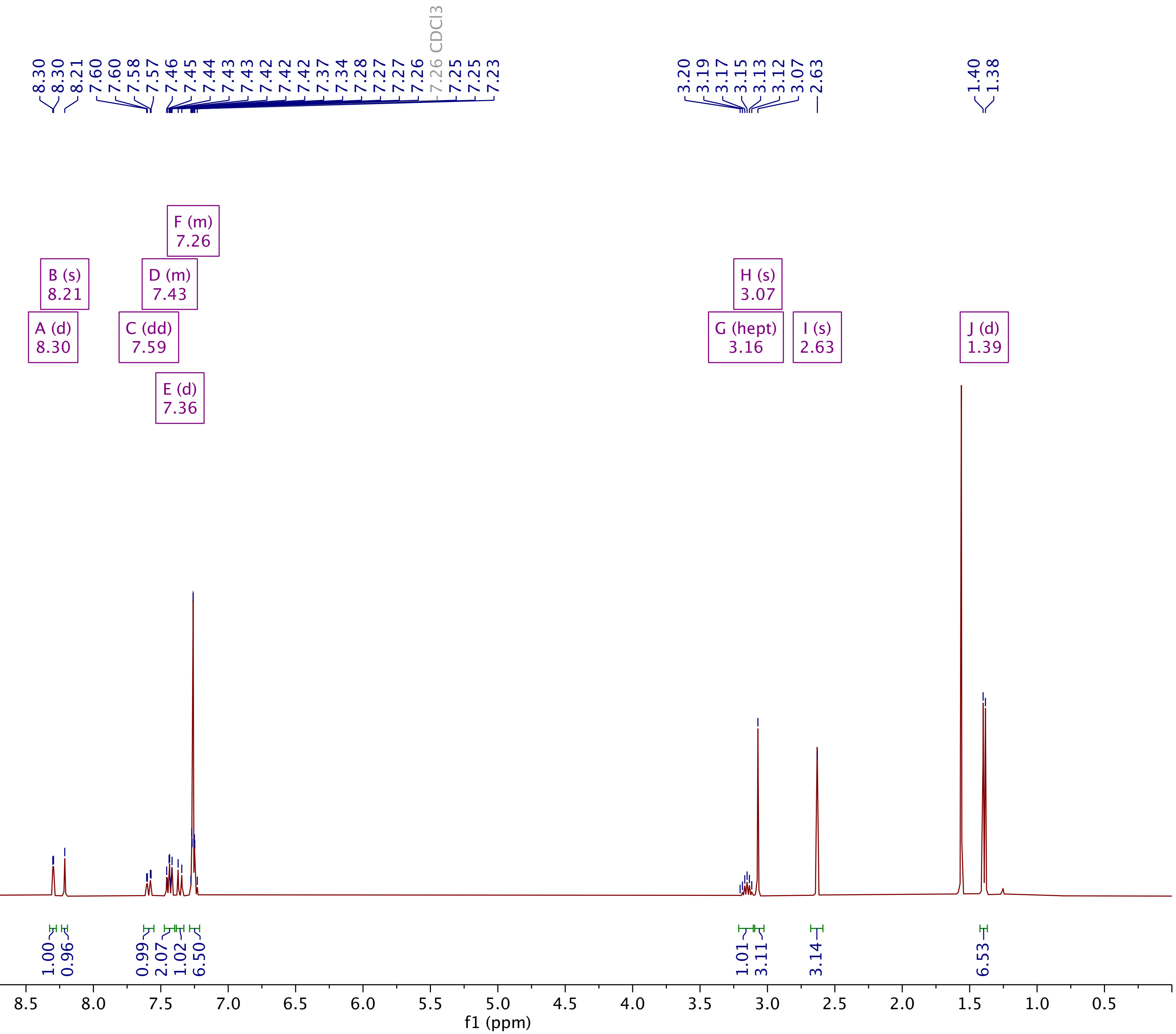

8o

400 MHz ^{1}H-NMR spectrum of **8o** in $CDCl_3$

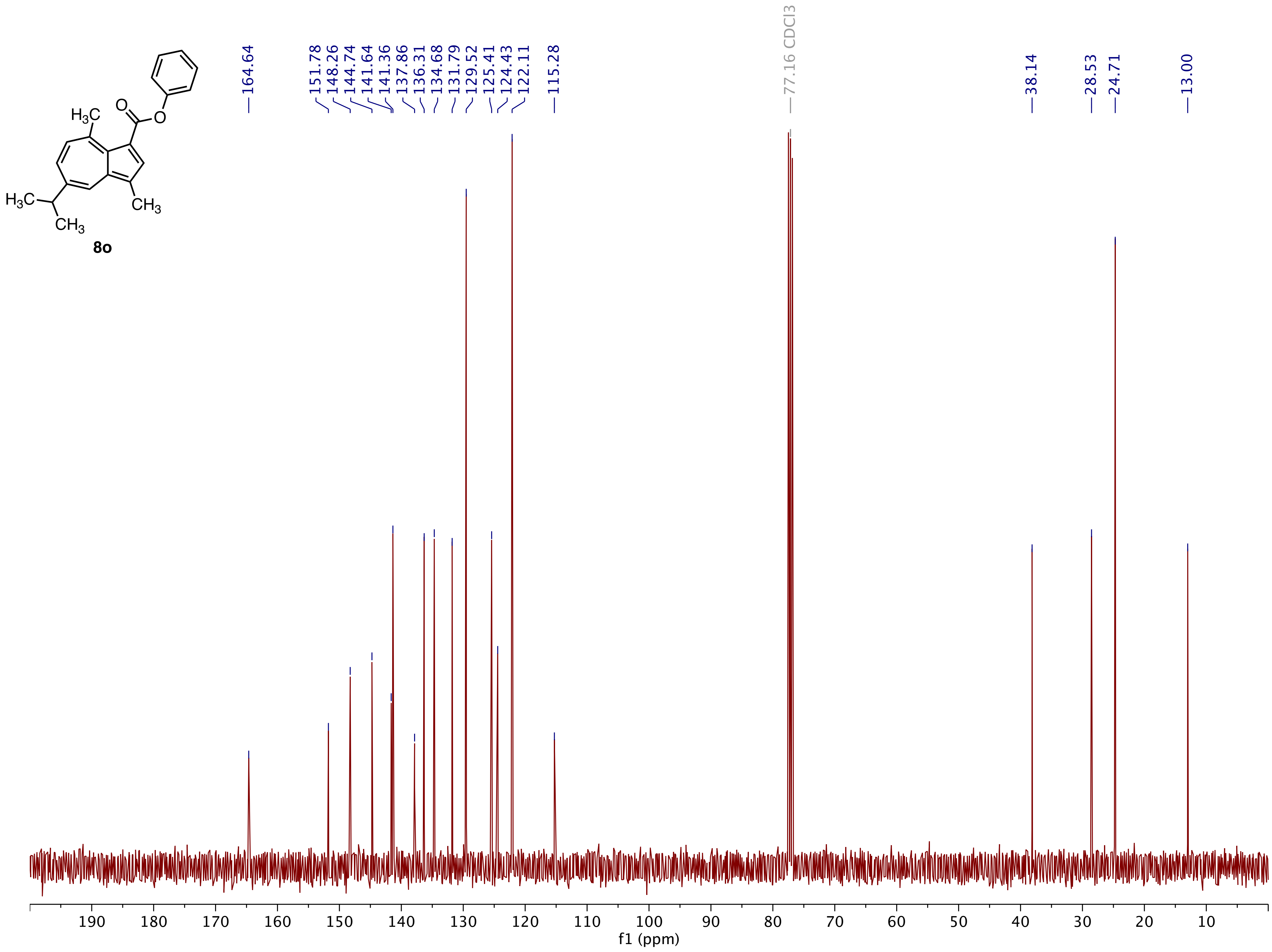

101 MHz $^{13}C\{^{1}H\}$-NMR spectrum of **8o** in $CDCl_3$

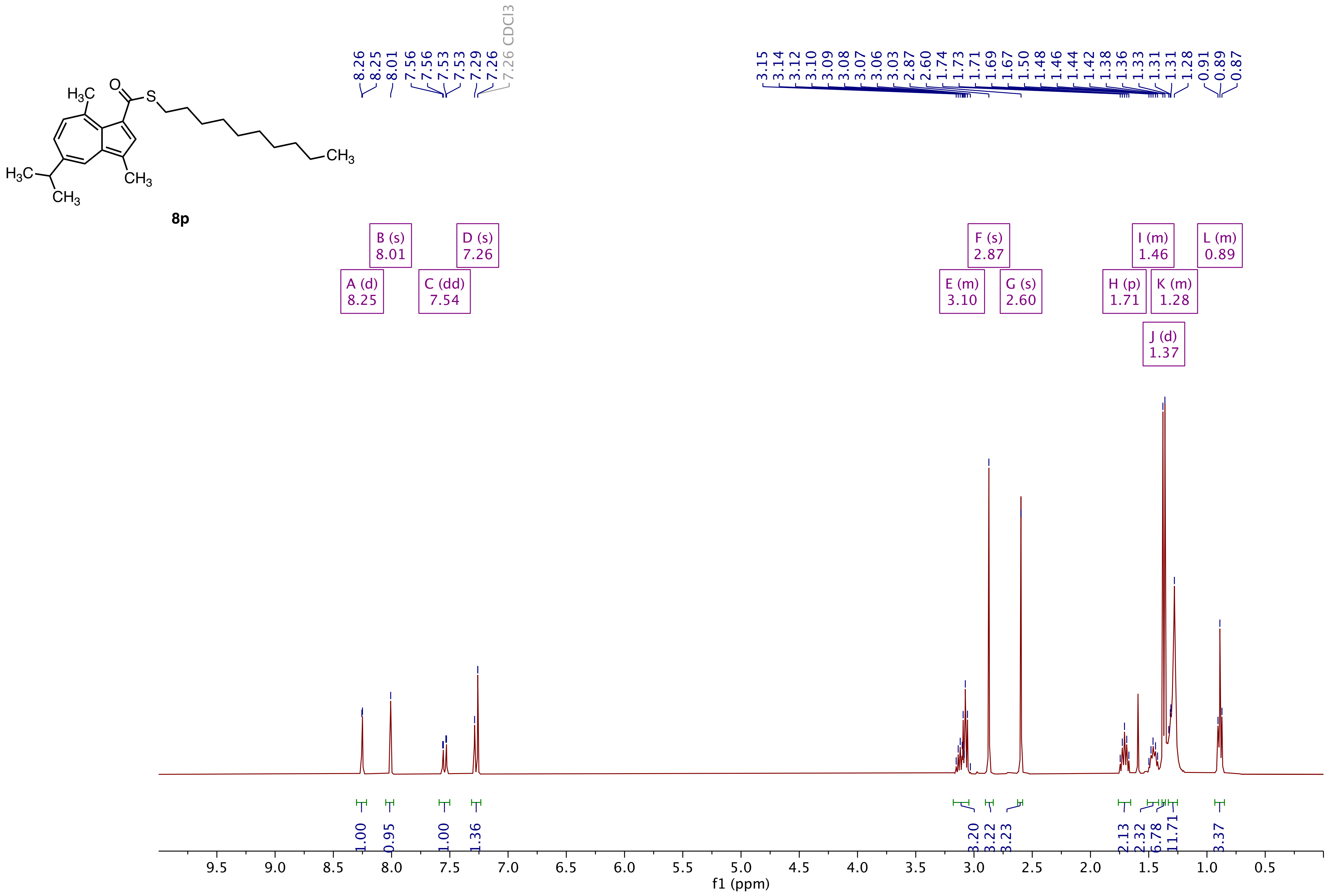

400 MHz ^{1}H-NMR spectrum of **8p** in $CDCl_3$

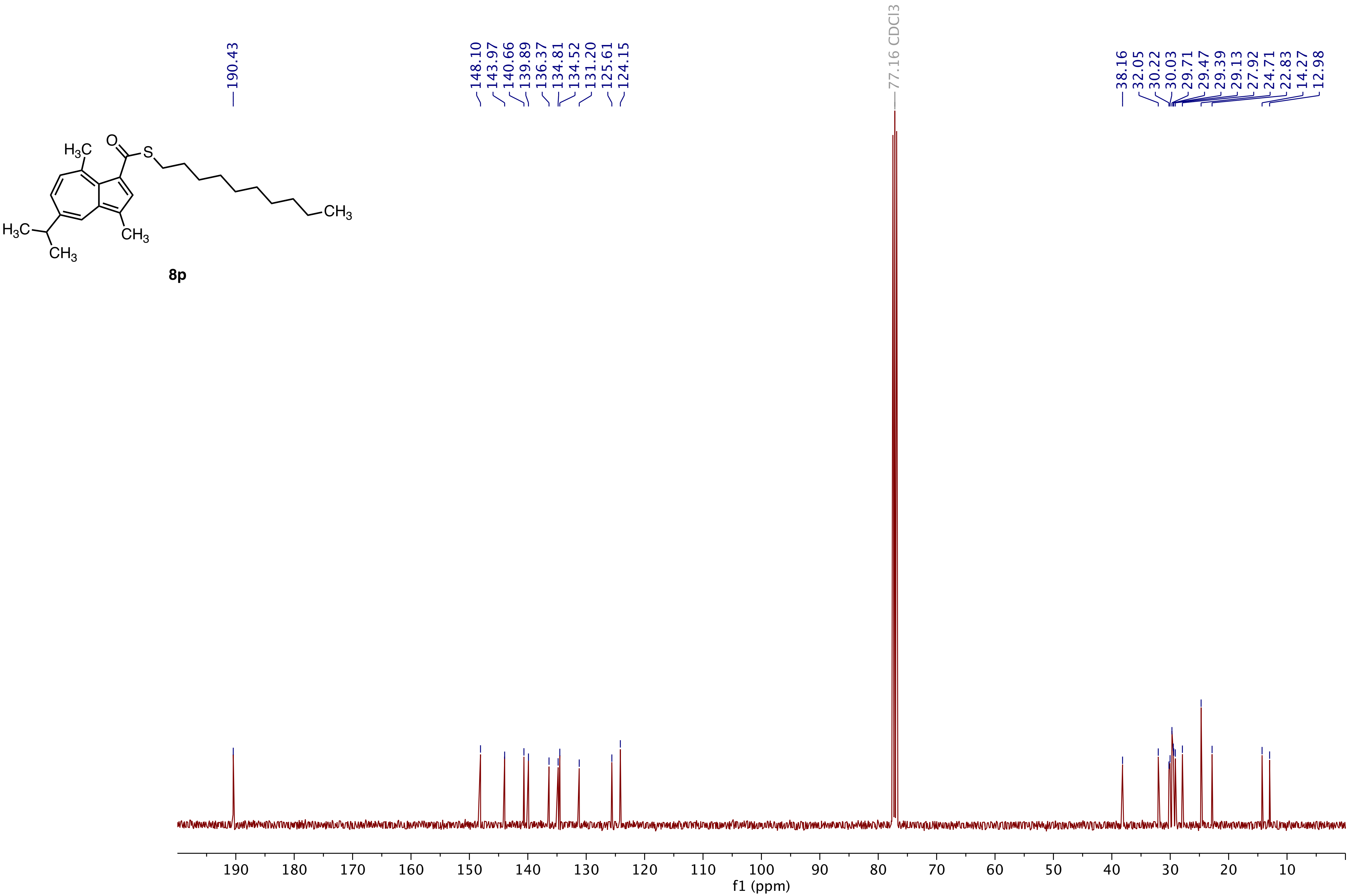

101 MHz $^{13}C\{^{1}H\}$-NMR spectrum of **8p** in $CDCl_3$

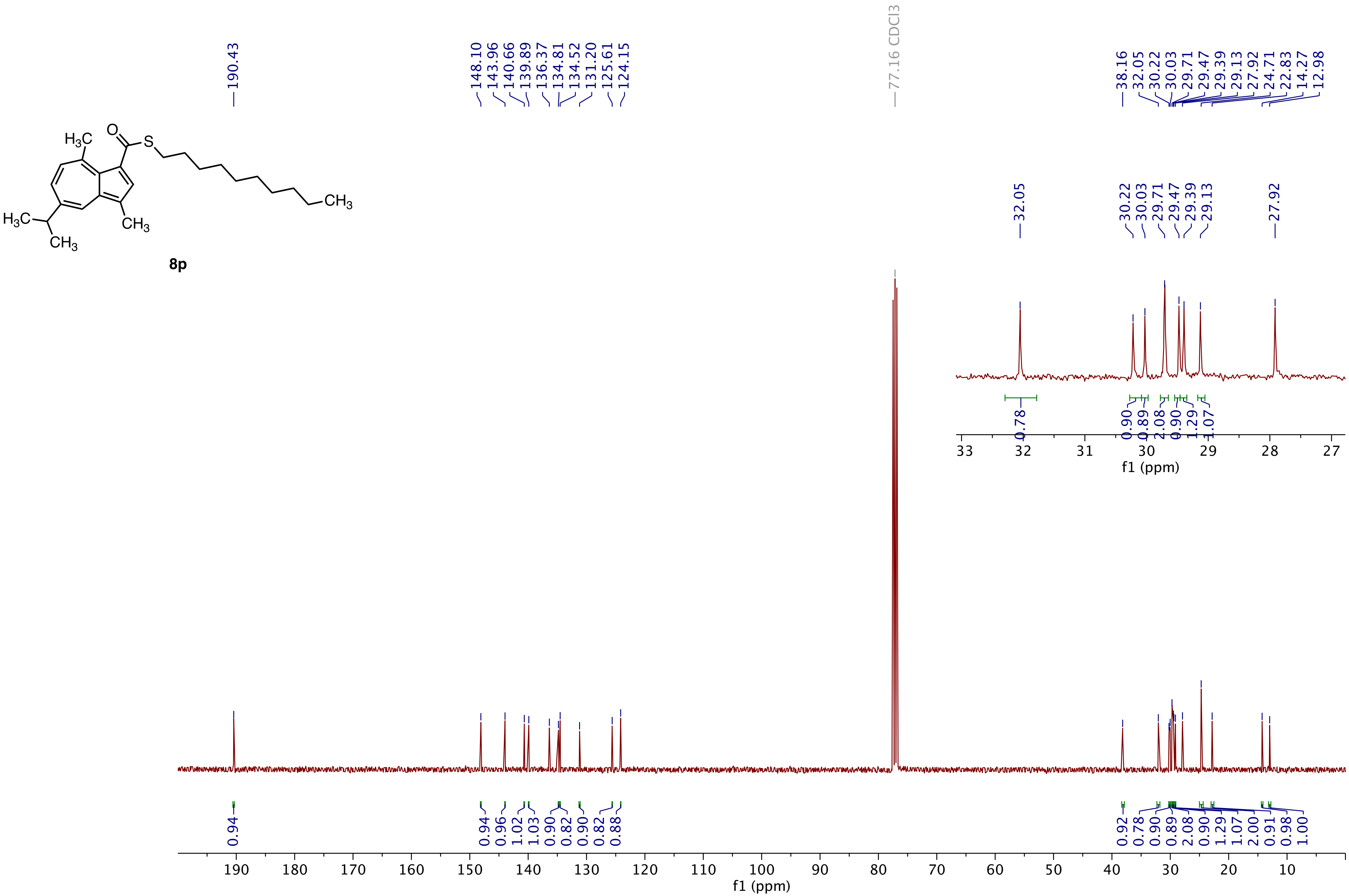

101 MHz quantitative $^{13}C\{^{1}H\}$-NMR spectrum of **8p** in $CDCl_3$

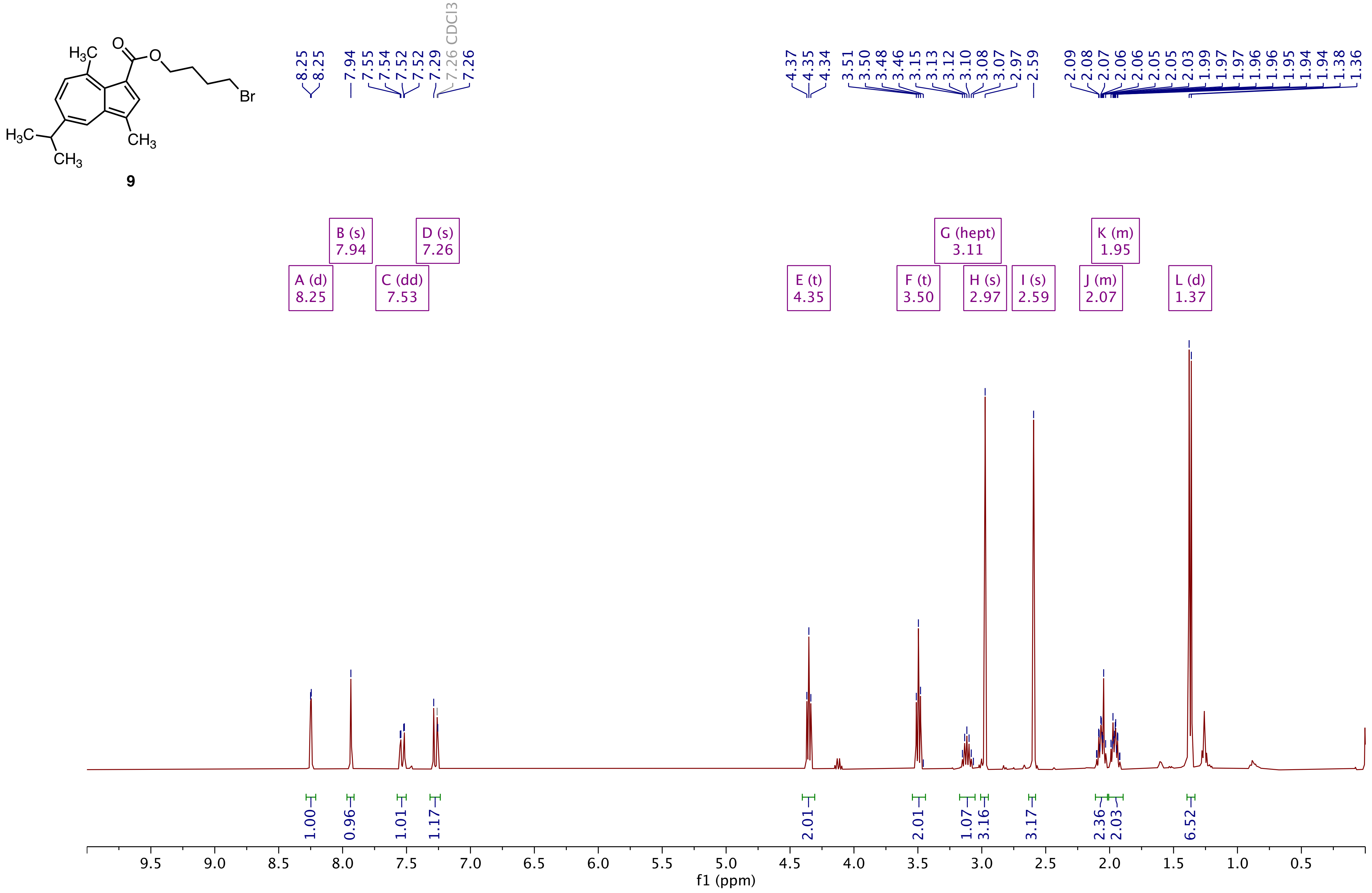

400 MHz ^{1}H-NMR spectrum of **9** in $CDCl_3$

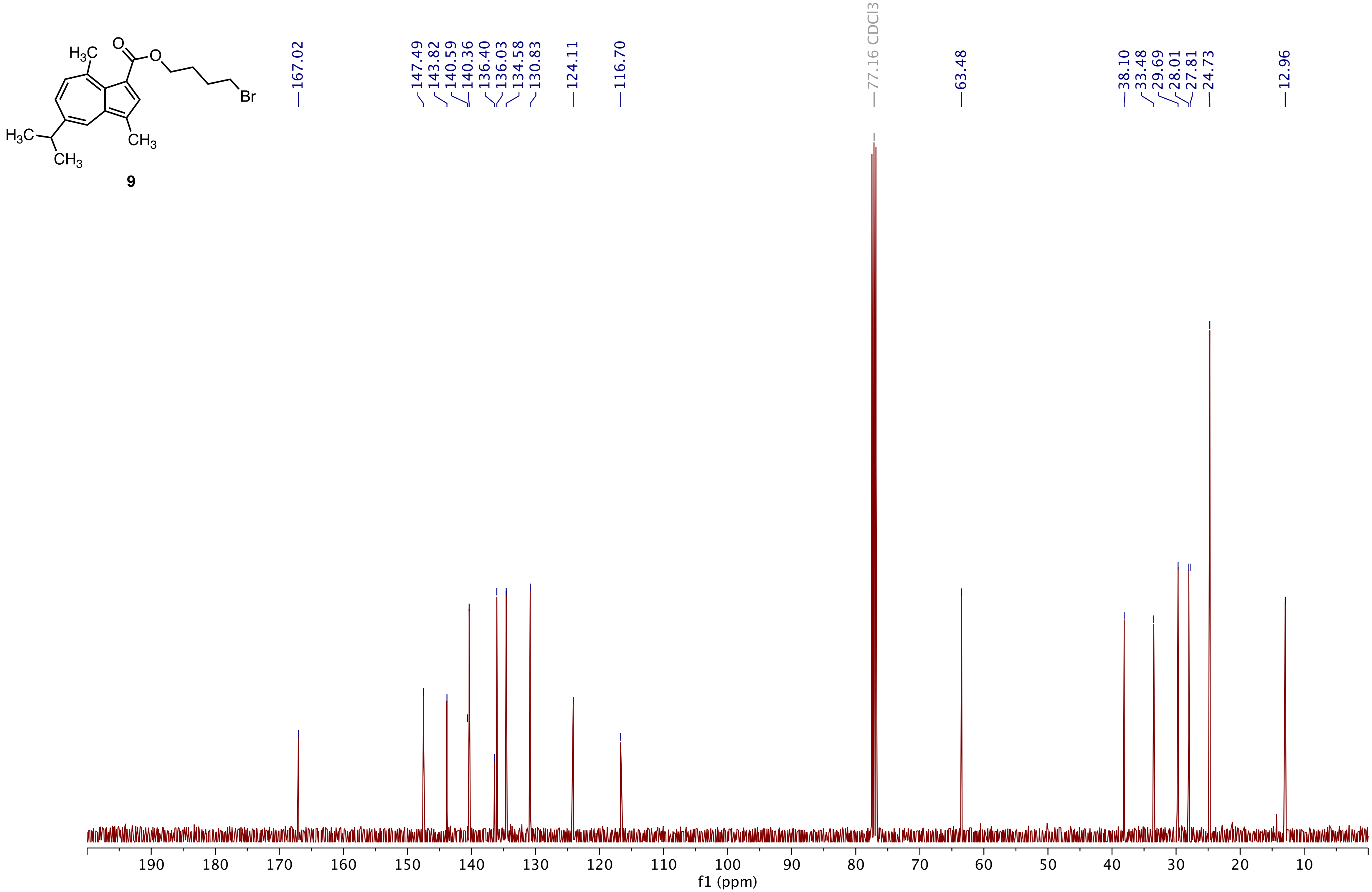

101 MHz $^{13}C\{^1H\}$-NMR spectrum of **9** in $CDCl_3$

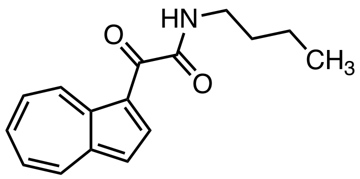

10a

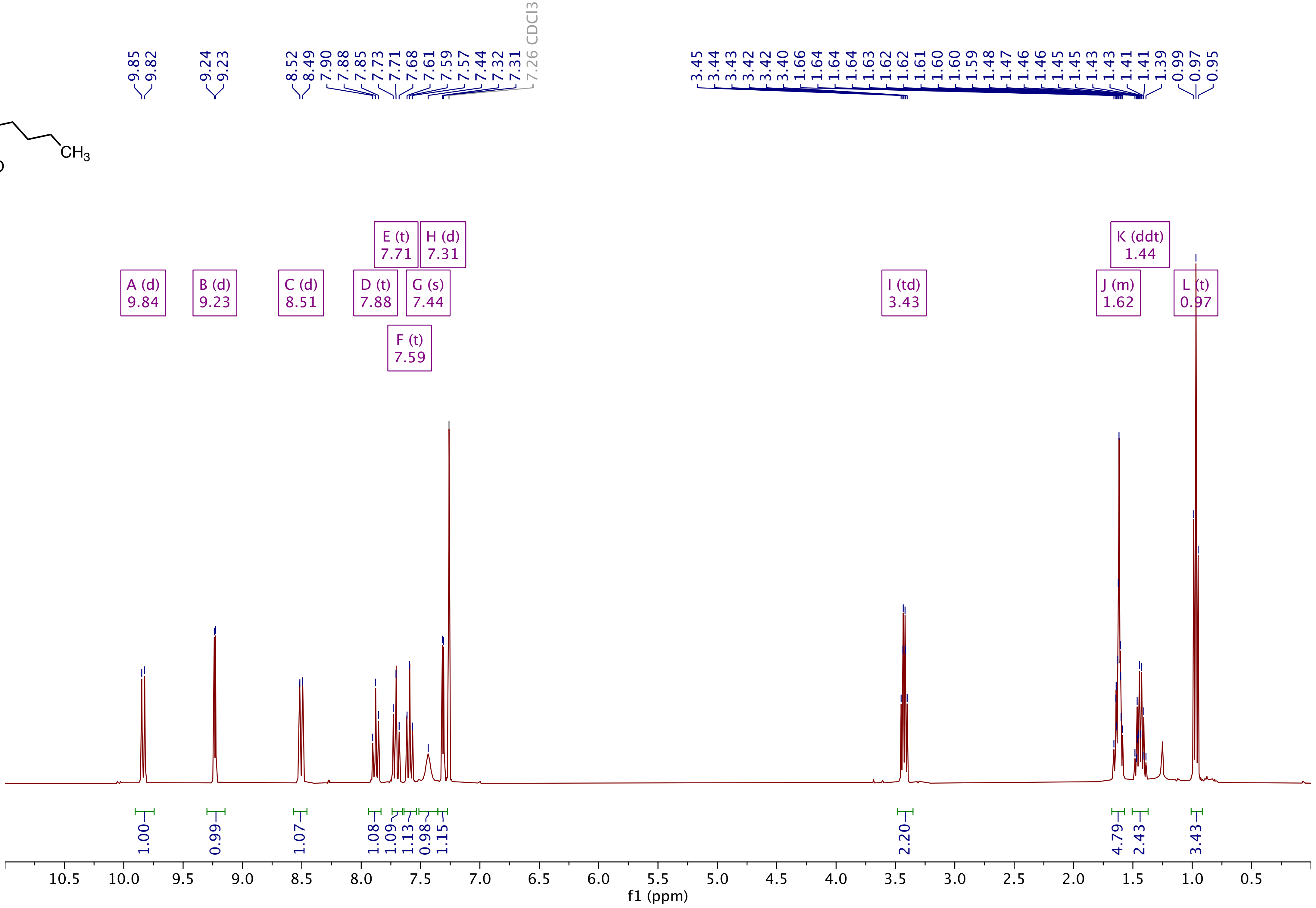

400 MHz ^{1}H-NMR spectrum of 10**a** in $CDCl_3$

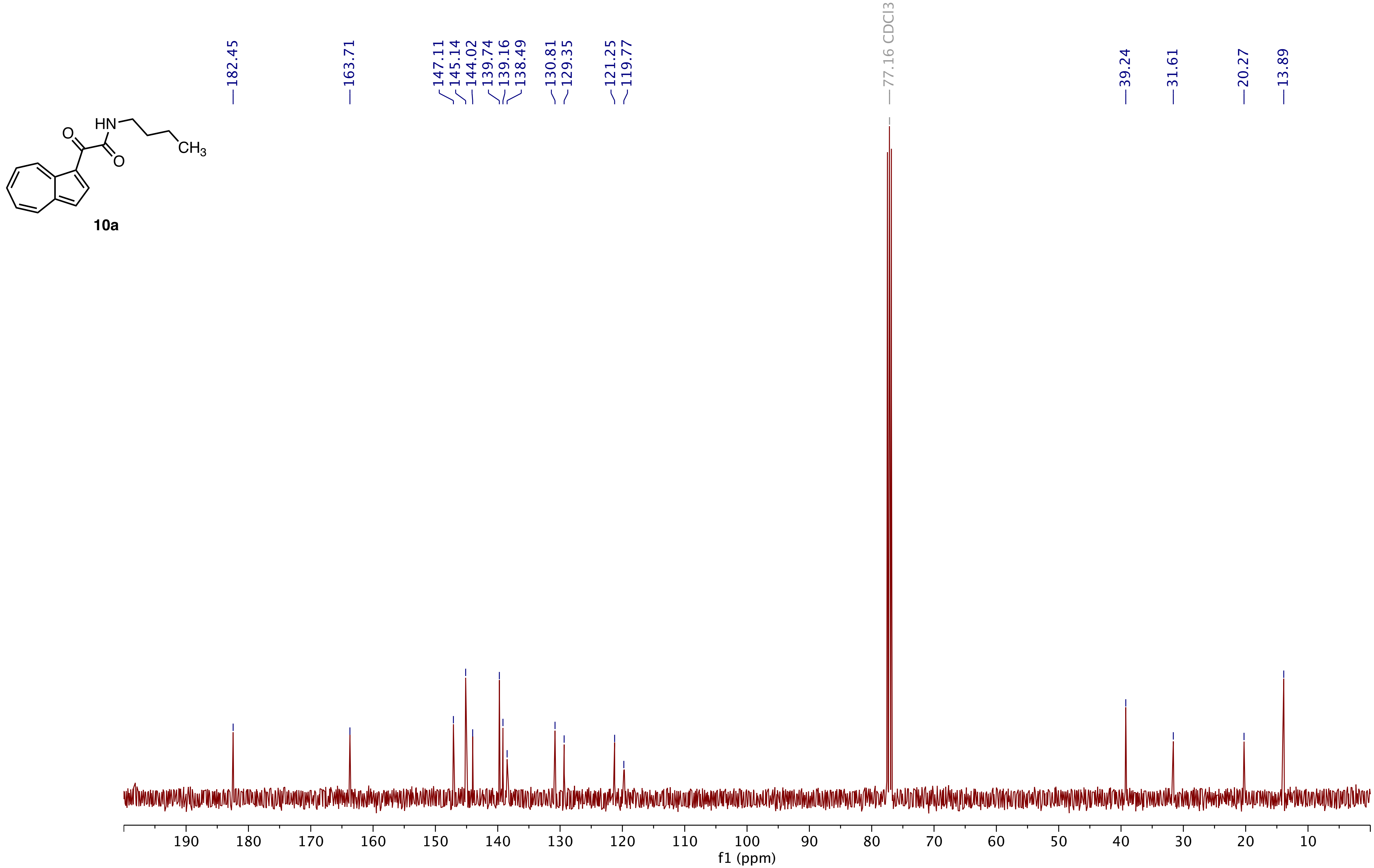

101 MHz $^{13}C\{^{1}H\}$-NMR spectrum of **10a** in $CDCl_3$

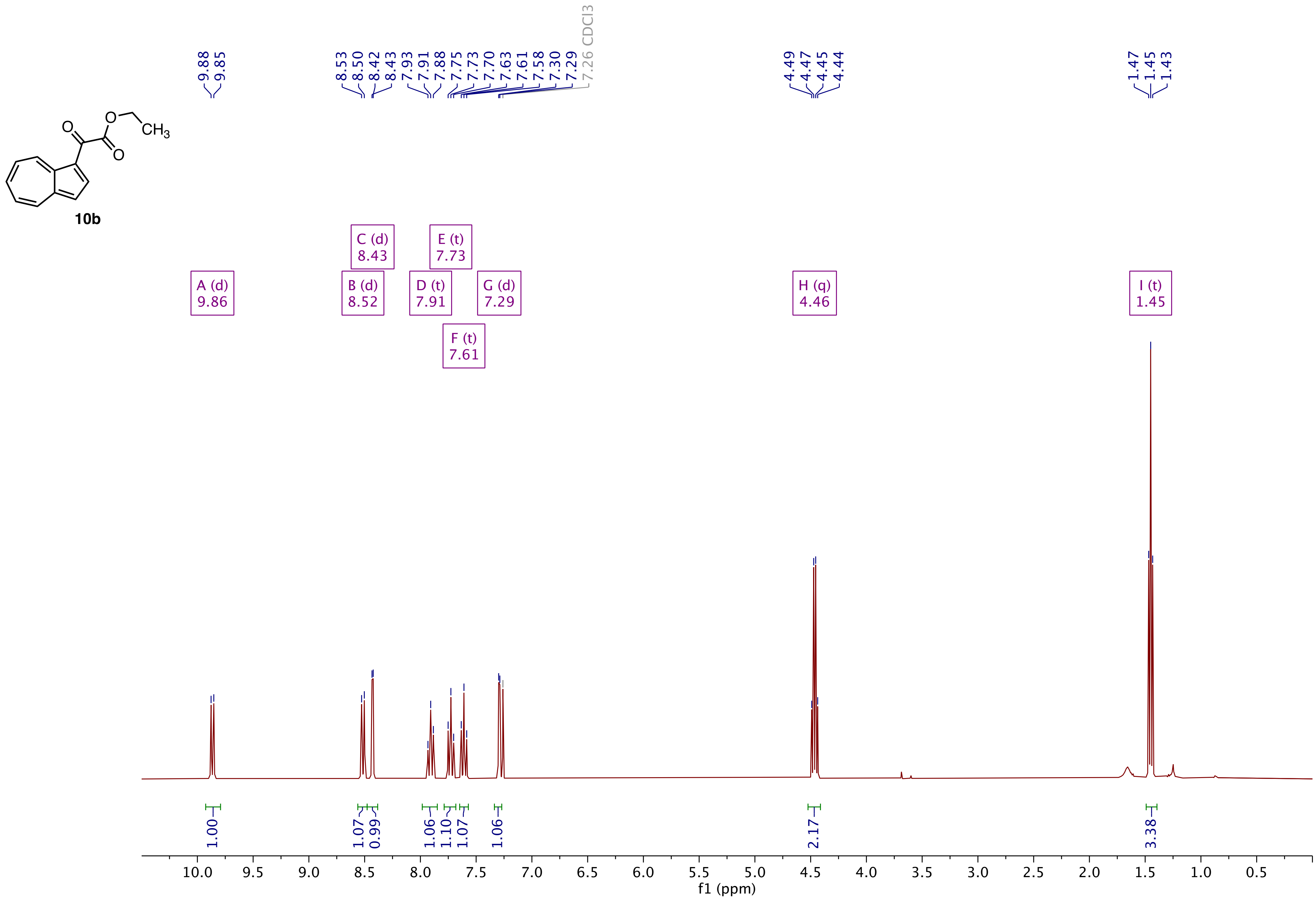

400 MHz ¹H-NMR spectrum of **10b** in $CDCl_3$

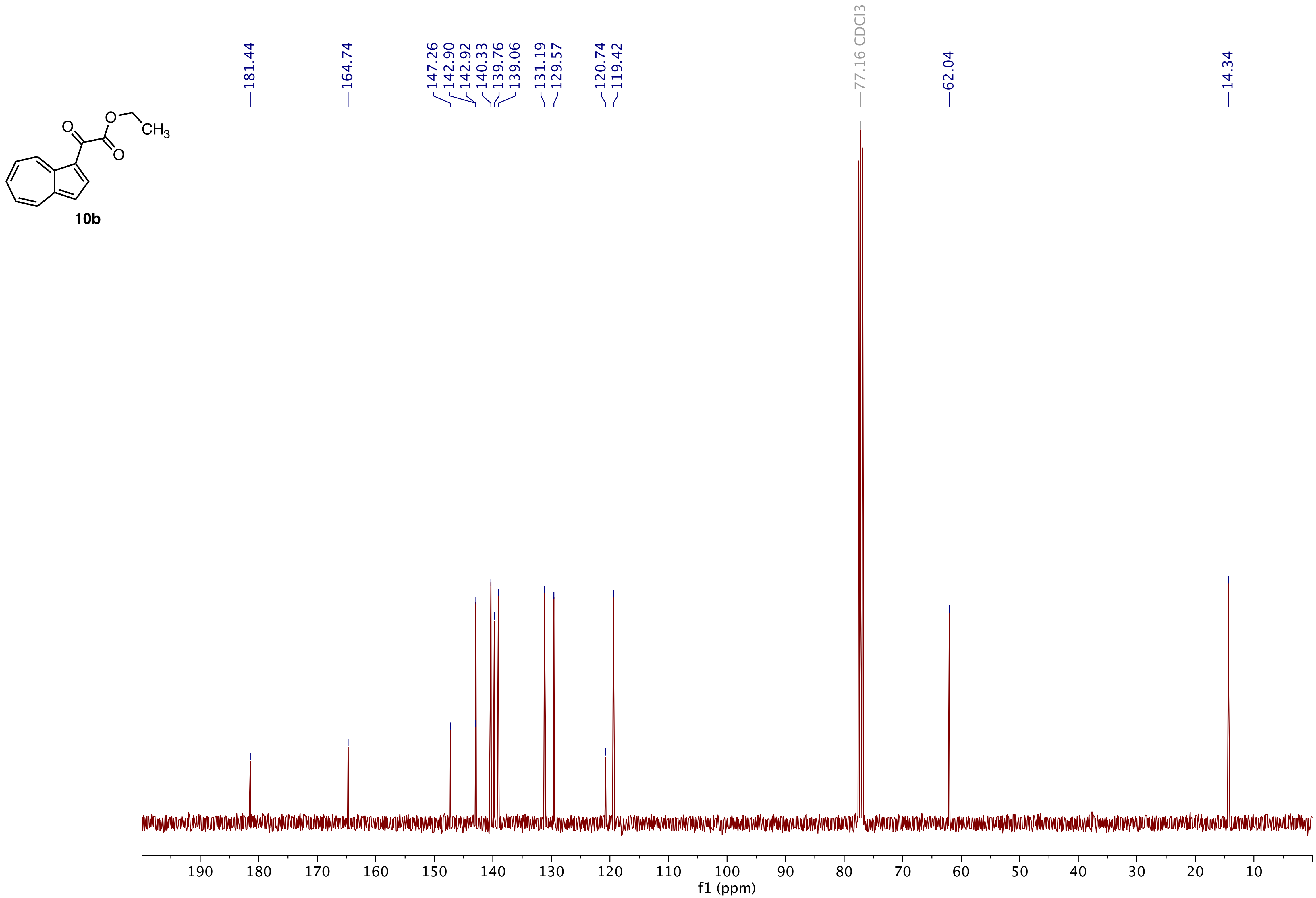

101 MHz $^{13}C\{^1H\}$-NMR spectrum of **10b** in $CDCl_3$

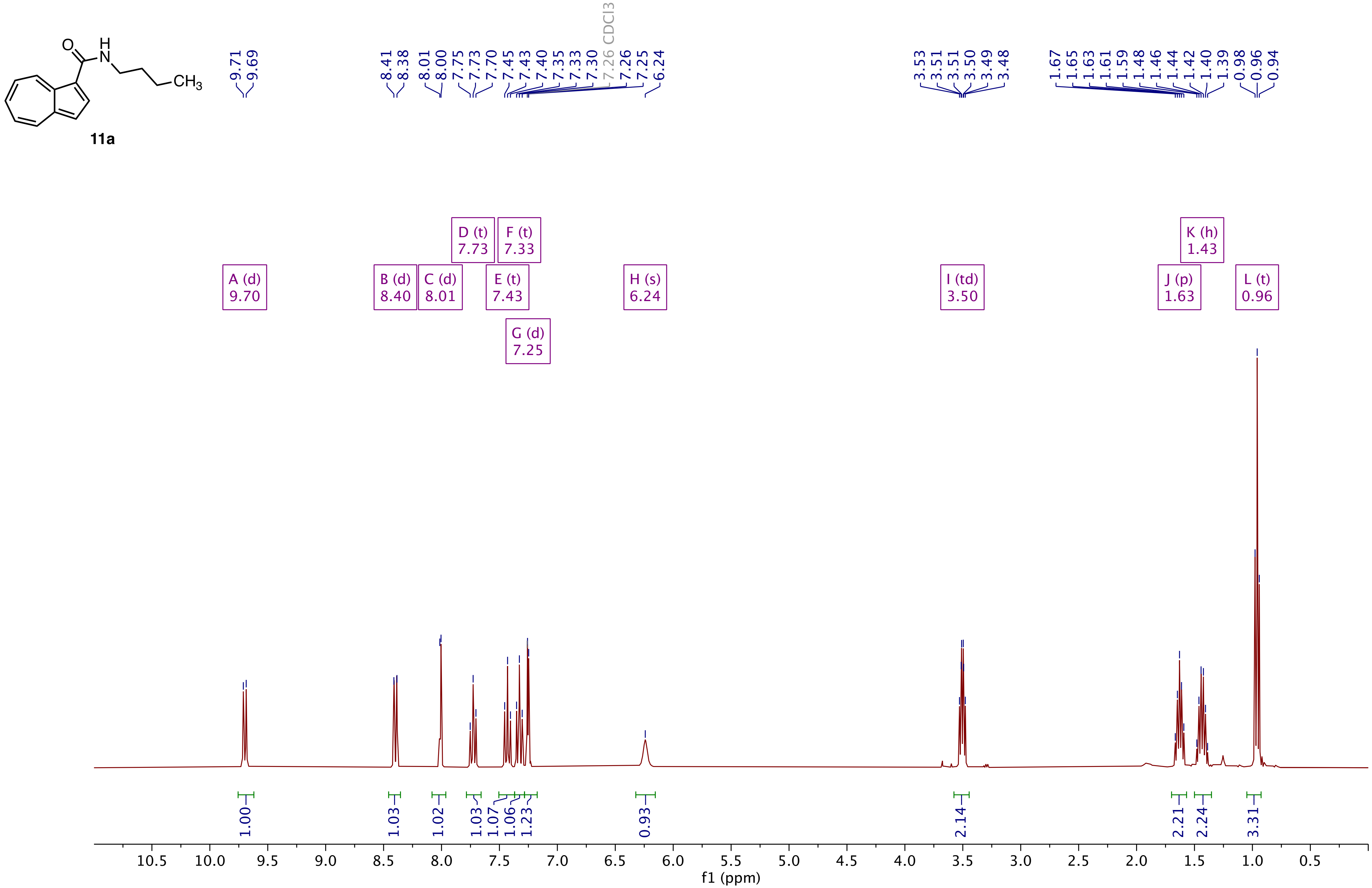

400 MHz ^{1}H-NMR spectrum of **11a** in $CDCl_3$

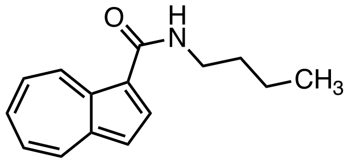

11a

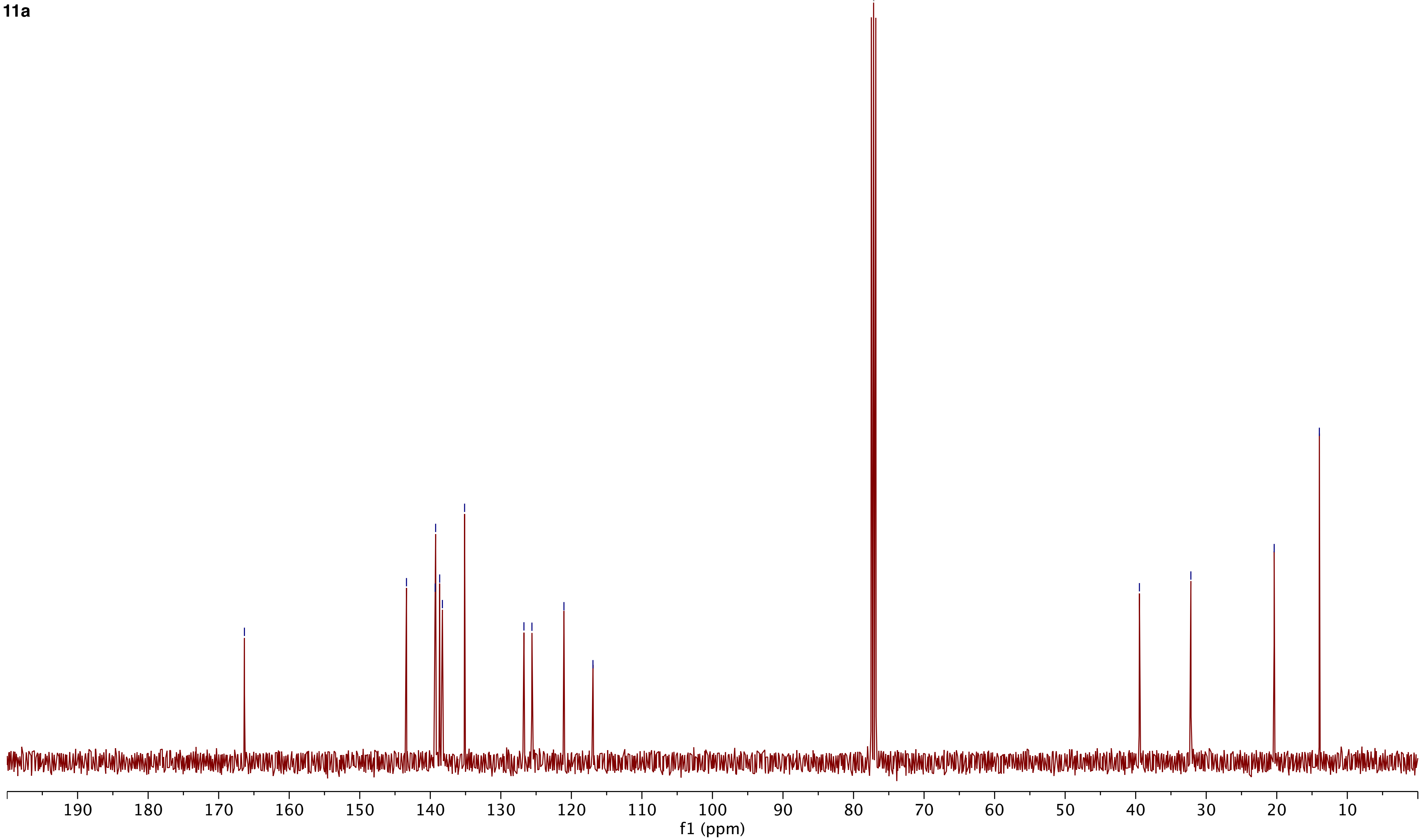

101 MHz $^{13}C\{^{1}H\}$-NMR spectrum of **11a** in $CDCl_3$

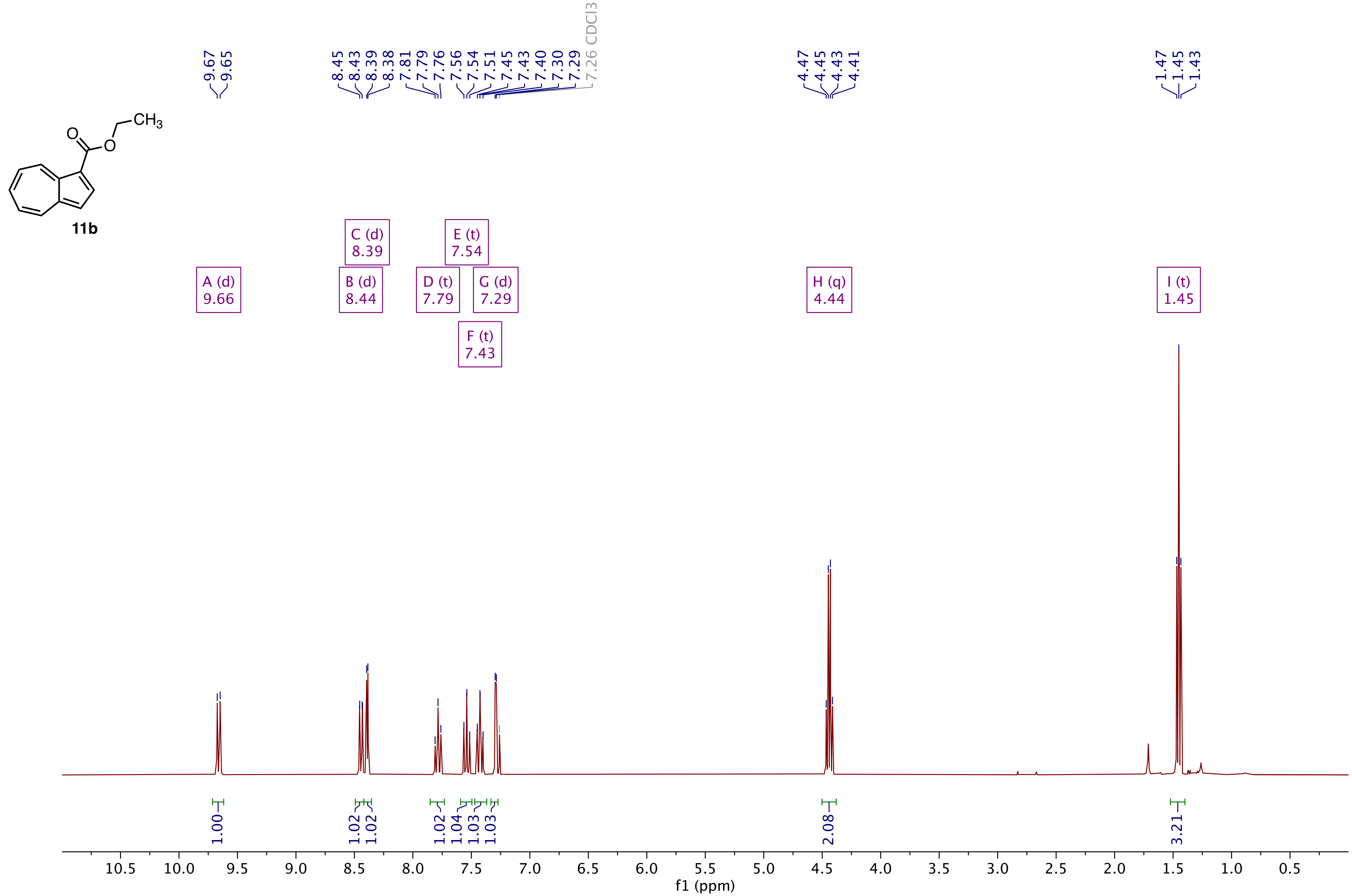

400 MHz ^{1}H-NMR spectrum of **11b** in $CDCl_3$

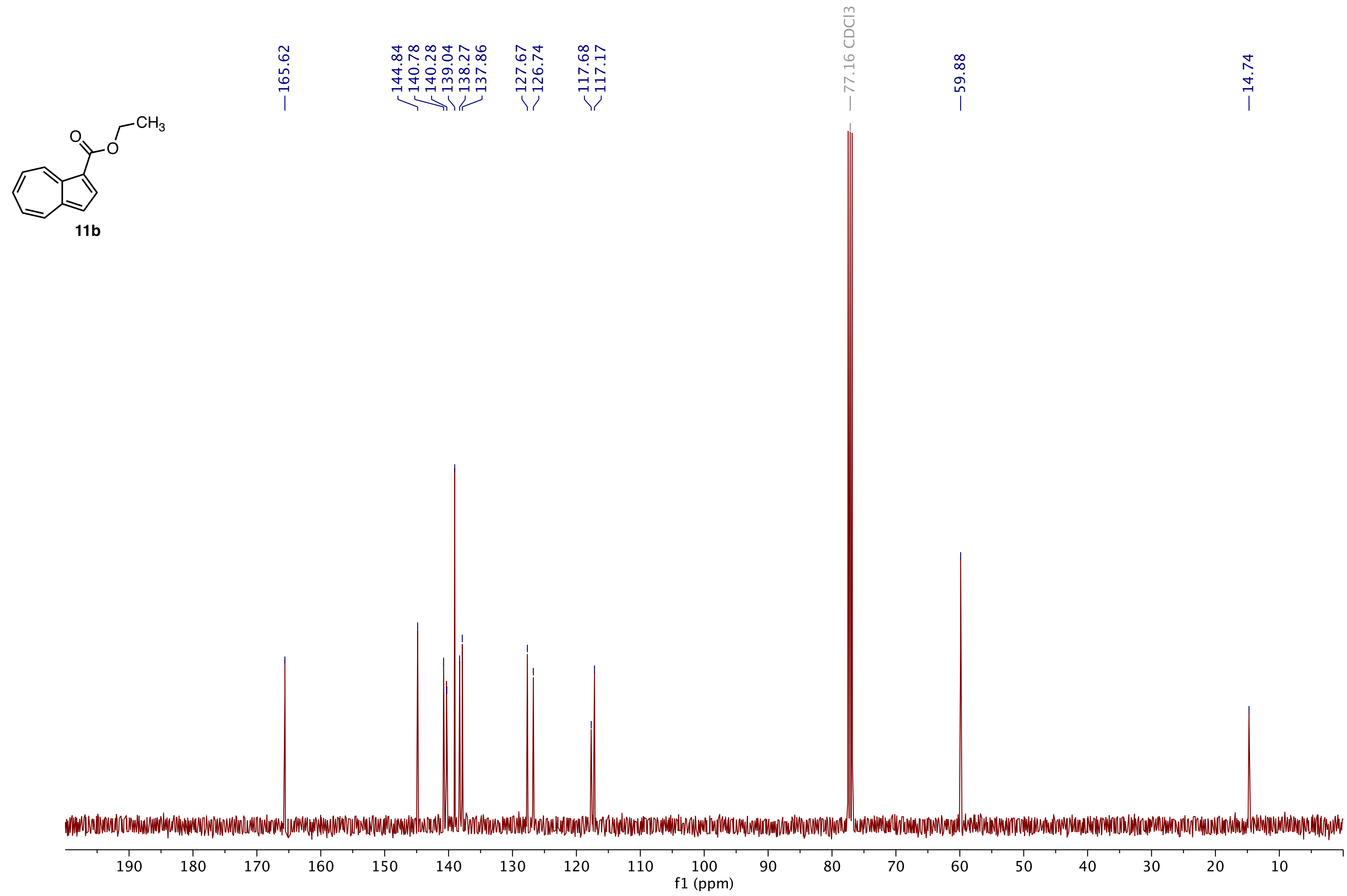

101 MHz $^{13}C\{^{1}H\}$-NMR spectrum of **11b** in $CDCl_3$
